# Accurate identification of *Helicoverpa armigera–Helicoverpa zea* hybrids using genome admixture analysis: implications for genomic surveillance

**DOI:** 10.3389/finsc.2024.1339143

**Published:** 2024-02-23

**Authors:** Dario Trujillo, Thiago Mastrangelo, Consuelo Estevez de Jensen, Jose Carlos Verle Rodrigues, Roger Lawrie, Steven E. Massey

**Affiliations:** ^1^ Department of Agro-Environmental Sciences, University of Puerto Rico - Mayaguez, Mayaguez, Puerto Rico; ^2^ Universidade de São Paulo, Centro de Energia Nuclear na Agricultura, Piracicaba, SP, Brazil; ^3^ USDA-APHIS-PPQ-S&T, Edinburg, TX, United States; ^4^ Center for Excellence in Quarantine and Invasive Species (CEQUIS), Estacion Experimental Agricola, San Juan, Puerto Rico; ^5^ Department of Biology, University of Puerto Rico - Rio Piedras, San Juan, Puerto Rico

**Keywords:** *Helicoverpa zea*, *Helicoverpa armigera*, hybrid, F1, admixture, genome, crop pest

## Abstract

*Helicoverpa armigera*, the cotton bollworm moth, is one of the world’s most important crop pests, and is spreading throughout the New World from its original range in the Old World. In Brazil, invasive *H. armigera* has been reported to hybridize with local populations of *Helicoverpa zea*. The correct identification of *H. armigera-H. zea* hybrids is important in understanding the origin, spread and future outlook for New World regions that are affected by outbreaks, given that hybridization can potentially facilitate *H. zea* pesticide resistance and host plant range via introgression of *H. armigera* genes. Here, we present a genome admixture analysis of high quality genome sequences generated from two *H. armigera-H. zea* F1 hybrids generated in two different labs. Our admixture pipeline predicts 48.8% and 48.9% *H. armigera* for the two F1 hybrids, confirming its accuracy. Genome sequences from five *H. zea* and one *H. armigera* that were generated as part of the study show no evidence of hybridization. Interestingly, we show that four *H. zea* genomes generated from a previous study are predicted to possess a proportion of *H. armigera* genetic material. Using unsupervised clustering to identify non-hybridized *H. armigera* and *H. zea* genomes, 8511 ancestry informative markers (AIMs) were identified. Their relative frequencies are consistent with a minor *H. armigera* component in the four genomes, however its origin remains to be established. We show that the size and quality of genomic reference datasets are critical for accurate hybridization prediction. Consequently, we discuss potential pitfalls in genome admixture analysis of *H. armigera-H. zea* hybrids, and suggest measures that will improve such analyses.

## Introduction

*Helicoverpa armigera*, the Old World cotton bollworm, is an Old World species of moth, and one of the world’s most important plant pests, whose larvae consume plants belonging to at least 68 plant families (1). In the New World, *H. armigera* was initially observed in Brazil in 2013 (2), and has subsequently spread throughout much of Latin America (3, 4), appearing to have undergone multiple introduction events into South America from the Old World (5, 6). There has not yet been a formal identification of *H. armigera* in North America, although it has been intercepted at several ports (7). The potential economic damage that *H. armigera* could cause in North America is large: $78 billion worth of crops in the United States were estimated to be susceptible to the pest in 2015 (7).

A closely related species, *Helicoverpa zea*, is native to the New World, and does not have such a wide host range, feeding off over 110 host plant species (8). *H. zea* does not possess such a high degree of resistance to common pesticides as that observed in *H. armigera* (9) [although resistance to Bt-proteins has been widely documented in *H. zea* (10)], implying it does not pose such an economic threat as *H. armigera*.

*H. armigera* and *H. zea* diverged approximately 1.5 million years ago (11), and are able to produce viable hybrids (12). *H. armigera-H. zea* hybrids have been reported from Brazil (13–15), but have yet to be identified from elsewhere. Adult *H. armigera* are difficult to distinguish from *H. zea* on the basis of morphology, requiring dissection of genitalia (16). Identifying hybrids using such methods is impossible, while larvae of the two species are likewise indistinguishable using morphology (17). In addition, such methods are inappropriate for screening large numbers of animals. While pure *H. armigera* and *H. zea* can be differentiated using species-specific PCR of the ITS1 region, this method does not work for hybrids (18). Hence, genomic methods have great potential utility for accurate species and hybrid identification.

The occurrence of *H. armigera-H. zea* hybrids in Brazil (13–15) has implications for pest management programs. Adaptive introgression of genes from invasive pest species into related local species poses a significant threat to global agriculture (17). A primary reason for studying *H. armigera-H. zea* hybrids in the field is to monitor the adaptive introgression of pesticide resistance genes to *H. zea* from *H. armigera*, which has been subject to intense selective pressure from synthetic pesticides (19, 20). For example, there is evidence that the *CYP337B3* gene, which confers resistance to pyrethroids, has already introgressed into *H. zea* populations in Brazil (15). The frequency of pesticide resistance genes in both *H. zea* and *H. armigera* populations has implications for the choice, duration and intensity of pesticide regimens dedicated to their control.

Genes in addition to those responsible for pesticide resistance may also have a propensity to introgress into local *H. zea* populations. For example, *H. zea* lacks genes for gustatory receptors and detoxification compared to *H. armigera*, which may help to explain its more limited range of host plant species (21). These genes may have the potential to introgress from *H. armigera* into *H. zea*, potentially increasing *H. zea’s* agricultural impact by increasing its range of host plants.

*H. armigera* has not yet been formally identified from North America, partly due to difficulties in distinguishing the species from *H. zea*. *H. armigera* was reported in Puerto Rico in 2014 and 2018, however since that time has not been reported again (22) The Caribbean represents a major transit route for pests and pathogens between North and South America (23), forming a ‘Caribbean corridor’, so Puerto Rico is a critical location for monitoring the potential spread of *H. armigera* from the South American continent into North America.

In this study, we implemented a bioinformatic pipeline to predict hybridization proportions by using whole genome sequences. We used the genomes of two lab generated *H. armigera-H. zea* F1 hybrids to confirm the accuracy of our admixture analysis procedure. We demonstrate that genomes from Puerto Rican and North American *H. zea* genomes generated as part of the study do not show evidence of hybridization with *H. armigera*. However, four attributed North American *H. zea* genomes from a previous study displayed potential evidence of hybridization, representing the potential early presence of *H. armigera* in North America. We show that high quality genome sequence data, reference genomic datasets and careful SNP filtration approaches are important for the accurate determination of hybridization proportions.

## Methods

### Collection and maintenance of parental species

Individual *H. zea* animals were collected by USDA APHIS collaborators (Todd Gilligan) and shipped to our lab in San Juan in ethanol from Colorado in 2015 (HzCol), Illinois in 2016 (HzIll), Maine in 2016 (HzMaine) and North Carolina in 2016 (HzNC). Species identifications were performed using species specific PCR of the ITS1 region, following the methods of (18).

All live *Helicoverpa* colonies were maintained under the following conditions: 25 ± 2°C, 57 ± 9% relative humidity, photoperiod of 15 hours of light and 9 hours of dark (15: 9 LD). Female pupae were placed in incubators at 22.7 ± 1.6°C, 82 ± 4% relative humidity, photoperiod of 15:9 LD, females were placed at a lower temperature to synchronize the emergence of adults with males (24). The larvae were fed with Gypsy Moth Diet (Frontier Agricultural Sciences, Product # F9630B, Newark DE): 140.2 g of dry mix, 20 g of fats and sugars, 1.6 g of vitamin mix, 0.8 g of aureomycin, 1000 ml of distilled water, with the addition of 12 ml of formaldehyde 1%, and 2.5 g of FABCO mold inhibitor (Frontier Agricultural Sciences, Product # F0018, Newark DE); the agar was dissolved, when the temperature was ~50°C the rest of the reagents were added. Each larva was maintained in transparent plastic cups of 30 ml containing diet. The pupae were maintained in the same cups.

Emerged adults and pupae near to emergence were placed in white plastic buckets of 18.9 l, the upper part of the buckets was covered with cheesecloth (DeRoyal, BIDF2012380-BX, Tennessee) for oviposition. Inside each bucket a Petri dish with autoclaved sand a potted tomato plant was placed to increase relative humidity. The adults received the following diet recipe modified from (25): 500 ml of distilled water, 50 ml of honey, 10 ml of solution 28% of Vanderzant vitamin mixture (Sigma, V1007, USA), 1 g of methyl-4-hydroxybenzoate (Sigma, H3647, USA), and 1 ml of ethanol 95%; methyl-4-hydroxybenzoate was dissolved in the 95% ethanol, then all the ingredients were mixed in the water and fed to adult moths using cotton wicks. The cheesecloth with the oviposited eggs was placed in Ziploc bags of 3.8 liters with fine strips of larval diet. Once larvae emerged, they were transferred to cups with diet. Prior to molecular work, all samples were stored in 90% ethanol in a –20 °C freezer until DNA extractions were performed.

In the Center for Excellence in Quarantine and Invasive Species (CEQUIS), separate colonies of *H. armigera* and *H. zea* were maintained. The colony of *H. armigera* was obtained from five larvae and 30 pupae from Brazil courtesy of Dr Thiago Mastrangelo, University of Sao Paolo. The insects were collected from Bahia (12°13’53’’S, 45°44’44’’W) in 2016 and were introduced to quarantine facilities of the CEQUIS on February 4, 2017, under Puerto Rico Department of Agriculture Permit number OV-1617-03 and USDA-APHIS Permit number P526P-15-04600 to Dr. José Carlos Verle Rodrigues. The initial colony of *H. zea* was obtained from larvae collected in Isabela, Puerto Rico, from pigeon peas on November 11, 2015. During the F9 generation, a reintroduction of insects was done, from larvae collected in Isabela in corn on November 22, 2016.

### Breeding of the hybrids

The first hybrid included in the study (PRh) was generated in our lab from a male *H. armigera* from Brazil, and a female *H. zea* from Puerto Rico. Using the same rearing methods described above, 15 *H. zea* female pupa and 15 *H. armigera* male pupa were placed into a white plastic bucket with cheesecloth lid and allowed to emerge, mate, and oviposit. All surviving F1 hybrids resulting from this cross were labeled and stored in a –20°C freezer. The F1 hybrid that was sequenced (PRh) was a female.

Genome sequences were generated from parental animals. A sequence from a male *H. armigera* from Brazil (HaM) was generated. This animal was an adult male *H. armigera* from the *H. armigera* colony initiated in CEQUIS, and was one of the parents for the F1 hybrids generated in the lab. A sequence from a female *H. zea* from Puerto Rico (HzF) was also generated. HzF was reared following the conditions described above, and was a parent for the *H. armigera*-*H. zea* F1 hybrids (PRh in this study) generated in the lab.

The second hybrid included in the study (MAh) was generated from a female *H. armigera* from Portugal and a male *H. zea* from the mainland USA by the USDA APHIS Otis Lab in Buzzards Bay, Massachusetts in 2017 by Dr. Hannah Nadel. The Portugese *H. armigera* mother was reared from pupae supplied by Dr. Delia Munoz, Public University of Navarra, Spain in 2016. The *H. zea* father used in the MAh cross were supplied by Benzon Research Inc. (Carlisle, PA, USA). This hybrid was reared under the same rearing conditions described previously. Of potential interest, an asymmetrical hybridization between the Brazilian lineages of *H. armigera* and *H. zea* has been observed, where male *H. zea* and female *H. armigera* have a higher probability of generating F_1_ offspring (26). Both MAh and PRh F1 hybrids were females.

### DNA extraction and sequencing

DNA samples were obtained from the animals using QIAGEN blood and tissue DNA extraction kits (QIAGEN INC., Cat No,/ID 69506) following the manufacturer’s protocol, with the exception of the Colorado, Illinois and North Carolina samples, which were extracted using the CTAB method (27). DNA quality was assessed using a NanoDrop 2000 (ThermoFisher Scientific, Waltham, MA) to assess DNA concentration (ng/uL) and absorbance (A260/280) and gel electrophoresis (1.5% agarose) to assess integrity and molecular weight. After checking DNA concentration and quality, the eight samples were shipped overnight on ice to the Rapid Genomics sequencing laboratory in Florida (www.rapid-genomics.com).

Paired end sequencing was conducted by Rapid Genomics on the Illumina HiSeq-X platform (sequencing statistics are displayed in **Supplementary Table 1**). The sequence data has been deposited in the National Center for Biotechnology Information (NCBI) Short Read Archive (SRA) under the Accession numbers SAMN35038651 (PRh), SAMN35038652 (MAh), SAMN35038653 (HzF), SAMN35038654 (HaM), SAMN35038647 (HzCol), SAMN35038648 (HzIll), SAMN35038649 (HzMaine), SAMN35038650 (HzNC). Additional genomic data was used in the analysis, consisting of 29 *H. armigera*, 9 *H. zea* and 9 *H. armigera*-*H. zea* hybrids, from (13) (**Table 1**). Raw sequence data for these animals were obtained from the Commonwealth Scientific and Industrial Research Organisation (CSIRO; https://data.csiro.au/collection/csiro:29053). Genome sequence analysis was performed on an Amazon Web Services c6g.4xlarge instance (comprising the AWS Graviton2 processor, 16 vCPUs, 32 Gb memory and Amazon Linux platform).

**Table 1:** Details of 47 additional *Helicoverpa* genomes used in the Admixture analysis.

Assigned species	Sample name	Sequence ID	Species assignment, (13) [in brackets from (15)] % *H. zea*	Species assignment from Admixture analysis (this work) % *H. zea*	Sample origin
*Helicoverpa zea*	70	Index_70_703_503_1	NA (98.0)	100	Brazil
*Helicoverpa zea*	73	Index_73_702_503_1	NA (97.3)	100	Brazil
*Helicoverpa zea*	132	Index_132_705_502_1	NA (99.9)	66.4	Brazil
*Helicoverpa zea*	133	133_N702_S502_CGTACTAG-CTCTCTAT_L002_R1_001	NA	44.8	Brazil
*Helicoverpa zea*	134	Index_134_705_503_1	NA (99.8)	76.9	Brazil
*Helicoverpa zea/hybrid*	TMG4	TMG4_N701_S501_TAAGGCGA-TAGATCGC_L002_R1_001	51.4 (47.4)	51.6	Brazil
*Helicoverpa zea*	HZRL10	Index_HZRL10_703_502_1	NA (100)	87.4	USA
*Helicoverpa zea*	HZRL12	Index_HZRL12_701_503_1	NA (100)	81.9	USA
*Helicoverpa zea*	HZRL17	Index_HZRL17_705_504_1	NA (100)	88.0	USA
*Helicoverpa zea*	HZRL20	Index_HZRL20_704_502_1	NA (100)	85.0	USA
*Helicoverpa armigera*	M0086	HaM0086_R1	NA	0	Australia
*Helicoverpa armigera*	M0118	HaM0118_R1	NA	0	Australia
*Helicoverpa armigera*	M0237	HaM0237_R1	NA	0	Australia
*Helicoverpa armigera*	M0260	HaM0260_R1	NA	0	Australia
*Helicoverpa armigera*	7	Index_7_703_504_1	NA	0	China
*Helicoverpa armigera*	8	Index_8_704_504_1	NA	0	China
*Helicoverpa armigera*	10	Index_10_705_501_1	NA	0	China
*Helicoverpa armigera*	12	Index_12_705_502_1	NA	0	China
*Helicoverpa armigera*	FMM1.3	Index_FFM1.3_706_502_1	NA	0	France
*Helicoverpa armigera*	FMM1.2	Index_FMM1.2_706_501_1	NA	0	France
*Helicoverpa armigera*	FMM1.4	Index_FMM1.4_706_503_1	NA	0	France
*Helicoverpa armigera*	738	Index_738_705_504_1	NA	0	India
*Helicoverpa armigera*	I3	Index_I3_701_502_1	NA	0	India
*Helicoverpa armigera*	ICY5L	Index_ICY5L_702_502_1	NA	0	India
*Helicoverpa armigera*	MAD13	Index_MAD13_702_501_1	NA	0	Madagascar
*Helicoverpa armigera*	MAD20	Index_MAD20_703_501_1	NA	0	Madagascar
*Helicoverpa armigera*	MAD3	Index_MAD3_701_503_1	NA	0	Madagascar
*Helicoverpa armigera*	MAD5	Index_MAD5_701_501_1	NA	0	Madagascar
*Helicoverpa armigera*	NZ24	Index_NZ24_701_501_1	NA	0	New Zealand
*Helicoverpa armigera*	NZ27	Index_NZ27_706_504_1	NA	0	New Zealand
*Helicoverpa armigera*	NZ29	Index_NZ29_702_501_1	NA	0	New Zealand
*Helicoverpa armigera*	SEN2	Index_SEN2_704_501_1	NA	0	Senegal
*Helicoverpa armigera*	SEN6	Index_SEN6_701_502_1	NA	0	Senegal
*Helicoverpa armigera*	SEN8	Index_SEN8_702_502_1	NA	0	Senegal
*Helicoverpa armigera*	S.5	Index_S.5_703_503_1	NA	0	Spain
*Helicoverpa armigera*	UG32L	Index_UG32L_705_503_1	NA	0	Uganda
*Helicoverpa armigera*	UG37L	Index_UG37L_701_503_1	NA	0	Uganda
*Helicoverpa armigera*	UG38L	Index_UG38L_702_503_1	NA	0	Uganda
*Helicoverpa armigera*	UG39L	Index_UG39L_703_503_1	NA	0	Uganda
*Helicoverpa armigera*/hybrid	110	110_N704_S504_TCCTGAGC-AGAGTAGA_L002_R1_001	8.9 (4.5)	5.5	Brazil
*Helicoverpa armigera*/hybrid	125	Index_125_702_501_1	3.2 (0)	5.6	Brazil
*Helicoverpa armigera*/hybrid	131	Index_131_704_501_1	2.4 (0)	0	Brazil
*Helicoverpa armigera*/hybrid	142	142_N703_S503_AGGCAGAA-TATCCTCT_L002_R1_001	7.9 (0.8)	4.6	Brazil
*Helicoverpa armigera*/hybrid	144	Index_144_706_501_1	2.8	0	Brazil
*Helicoverpa armigera/*hybrid	BRA2	Index_BRA2_704_503_1	3.2 (0.5)	0	Brazil
*Helicoverpa armigera/*hybrid	BRA4	Index_BRA4_701_504_1	4.6 (0.2)	2.0	Brazil
*Helicoverpa armigera/*hybrid	TPG2	Index_TPG2_701_501_1	2.1 (0)	0	Brazil

Genome data was obtained from (13). ‘NA’ means ‘not available’.

### Mapping and SNP calling procedure

Using fastp (28), sequences were removed if they did not fulfill the criteria of 95% nucleotides > Q20, 3’ trimming was conducted by quality, and polynucleotide runs (6 or more consecutive). Filtered and trimmed sequences were repaired using the repair.sh script of BBMap (v37.99) (sourceforge.net/projects/bbmap). They were then mapped to the *H. armigera* reference genome (21) (all-chr-r.fasta, obtained from CSIRO at https://data.csiro.au/collection/csiro:29053v1), using BBMap in paired-end mode. The resulting sam files were converted to bam files, and sorted using SAMtools (29).

BCFtools mpileup (29) was used for variant calling. Bam files for all *Helicoverpa* genomes in the study were processed together, to improve the accuracy of calls of SNPs shared across genomes. After SNP calling, the resulting vcf files were filtered using vcftools (30), removing those SNPs that possessed mean read depth (min-meanDP < 5), Q value (Q < 20) and minor allele frequency (MAF < 0.05).

### Admixture analysis

Admixture v1.3.0 (29) was used for genome admixture analysis, and can be used to infer global ancestry proportions (31). Admixture assumes that genetic loci are independent. While linkage disequilibrium (LD) is not explicitly modeled by Admixture, it provides a useful approximation of global ancestry (29). In order to account for potential effects of LD, LD pruning was conducted using plink and an r^2^ threshold of 0.95, resulting in the removal of 277381 SNPs. Sex chromosomes (chromosome 1) were excluded from the analysis. Plink (32) was used to convert the combined vcf file into bed format, which was used as input for the Admixture analysis, which was run using K=2. Admixture output was visualized using the R ggplot2 package (https://github.com/tidyverse/ggplot2).

### Identification of ancestry informative markers

34 *H. armigera* and 7 *H. zea* unhybridized genomes were identified using the unsupervised clustering approach of Admixture, described above. The genotype data from these genomes was then used to identify SNPs that possessed a minor allele count (MAC) of 7 for the *H. zea* genomes and 1 for *H. armigera* genomes, using vcftools. These were then pruned by removing all SNP positions where a SNP was completely absent (GT = 0/0) from one or more *H. zea* genome.

## Results and discussion

Genome sequencing results are shown in **Table 2**, and show that the quality of the raw sequences was high for all eight genomes. For consistency, SNP calling was jointly conducted on the *Helicoverpa* raw sequence reads generated by (13), and on the sequences generated as part of this study. Filtering resulted in the removal of a large proportion of SNPs (83%); this might be reduced in future by increasing sequence depth in the overall dataset.

**Table 2:** Mapping and SNP statistics, and hybridization proportions, of eight new genome sequences generated in the study.

Sample name	Average genome sequencing depth	Number of SNPs after filtering	Species assignment from Admixture analysis (this work) % *H. zea*	Geographic origin
PRh	31.4	9230976	51.2	F1 hybrid of male *H. armigera* from Brazil and a female *H. zea* from Puerto Rico
MAh	34.7	8990547	51.1	F1 hybrid of a female *H. armigera* from Spain and a male *H. zea* from USA
HaM	43.7	7189237	0	Brazil
HzF	37.4	5139536	100	Puerto Rico
HzCol	25.0	5185015	100	Colorado, USA
HzIll	25.0	5190809	100	Illinois, USA
HzMaine	25.3	5188817	100	Maine, USA
HzNC	26.0	5793330	100	North Carolina, USA

### Predicted hybridization proportion of the two lab-reared F1 hybrids

**Figure 1:**
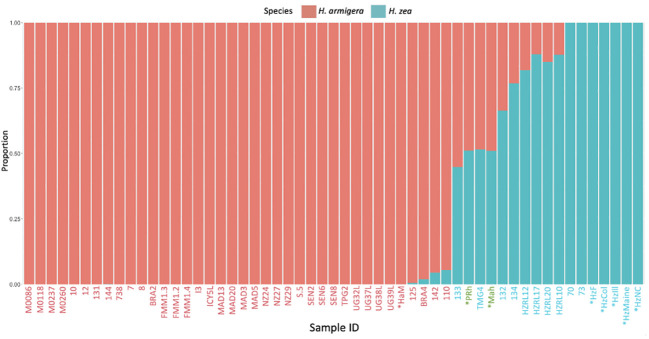
Admixture analysis of *Helicoverpa armigera* and *Helicoverpa zea* genomes. The bar plot shows the relative proportions of *H. armigera* and *H. zea* present in *Helicoverpa* genomes generated in this study (PRh, MAh, HaM, HzF, HzCol, HzIll, HzMaine, HzNC), and from (13). The Admixture analysis used K=2, and excluded sex chromosomes. The X-axis displays sample ID code, metadata for each sample is described in **Tables 1** and **2**. X-axis labels in red are *H. armigera* samples, labels in blue are *H. zea* samples, and labels in green are *Helicoverpa zea-armigera* F1 hybrids. X-axis labels with an asterisk are those that were sequenced during this study, the other samples are from previous studies. The Y-axis displays the proportion of SNPs specific to either *H. zea* (blue) or *H. armigera* (red).

In the hybrid animals, approximately equal proportions of the genome originate from both *Helicoverpa* species (51.1 % *H. zea*: 48.9% *H. armigera* in PRh and 51.2% *H. zea*: 48.8% *H. armigera* in MAh). These data are displayed on the Admixture plot (**Figure 1**). In both cases, the Admixture prediction is not exactly 50% *H. zea*: 50% *H. armigera* for either hybrid, even though in the case of PRh, genomes derived from the parental populations were 100% *H. zea* (HzF) and 100% *H. armigera* (HaM). This may be due to two reasons. Firstly, the (male) parental insect population may have possessed a degree of hybridization because they were collected originally from Brazil near where early hybrids have since been detected (15). However, it is notable that the MAh F1 hybrid also has a similar *H. armigera*: *H. zea* ratio (48.8%: 51.2%). This would mean that the *H. zea* from the USA, used to generate the hybrid would also have to have had a low level of *H. armigera* admixture; this seems more unlikely than for an *H. zea* insect from Brazil, where the presence of hybrids has been validated.

Secondly, the Admixture analysis may lack exact precision. This may be the result of a limited number of pure *H. zea* in the dataset (seven), which means that the genetic diversity of the species is not adequately represented. This is supported by the observation that the *H. armigera*: *H. zea* ratio is the same for both F1 hybrids: this indicates a systemic bias in admixture prediction.

Encouragingly, even though *H. armigera* and *H. zea* are closely related species, the Admixture analysis is capable of accurately identifying the relative proportions present in an F1 hybrid genome. In future, accuracy may be improved by refinements in SNP calling, increasing the sequencing depth in the overall dataset and adding additional genomes, particularly from *H. zea*. Admixture analysis may be affected by a small sample size of one or more of the reference populations (33). In the analysis, even after the addition of five *H. zea* genomes generated in this study, only seven non-hybridized *H. zea* genomes were apparent.

### Predicted hybridization proportions of other *Helicoverpa* spp. genomes

From the new genome data generated by the study, the analysis indicated that the animals identified as *H. armigera* (HaM), and *H. zea* (HzF, HzCol, HzIll, HzMaine, HzNC), were non-hybridized animals. All Old World *H. armigera* datasets from (13) were identified as non-hybridized, as expected.

The Admixture analysis reveals some discrepancies with those previously published for 47 previously sequenced *Helicoverpa* genomes (13), which were used as a reference dataset here and in other studies. Most of the animals previously identified as 100% *H. zea* (13, 15) are predicted in our analysis to have a *H. armigera* component (132, 133, 134, HZRL10, HZRL12, HZRL17, HZRL20), while several specimens previously identified as hybrids (13, 15) were identified here as 100% *H. armigera* (131, 144, BRA2, TPG2) (**Table 1**). TMG4, previously described as a *H. zea* hybrid (13, 15), is also predicted by our analysis as a hybrid and appears to be F1, given its predicted proportion of 48.9% *H. armigera*. Given that this animal was collected in August 2013, this implies that hybridization occurred one generation previous to the collection date.

A key difference between our study and (13, 15) is that our inclusion of two lab-reared F1 hybrids allows us to verify the accuracy of our analysis. Potential explanations for differences in predicted hybridization proportions reported in (13) may include lack of filtration after SNP calling, and the lower number of *H. zea* in the dataset (leading to a limited reference population for this species). In addition, in (13) SNPs were called on a dataset which included *Helicoverpa punctigera*, *Helicoverpa gelotopoeon*, *Helicoverpa hardwicki* and *Helicoverpa assulta*. In our method, our simultaneous SNP calling procedure only included *H. armigera* and *H. zea* datasets. In addition, in our analysis we chose not to include the Z sex chromosome (chromosome 1), focusing only on autosomes.

The reason for differences between our study and the predicted species proportions described in (15) is less clear, given that the authors used filtration criteria similar to our own, and only called SNPs against *H. armigera* and *H. zea* genomes, rather than including additional *Helicoverpa* spp. in their analyses. However, the ancestry proportions that they report in their Supplementary Table S4 are derived from ~1 million SNPs identified as segregating between the two species, whereas we base our ancestry proportions on Admixture analysis, consequently methodological differences may provide the source of the discrepancy.

### Potential *H. armigera* hybridization detected in North American *H. zea* from 2005

The identification in the reference dataset of potential *H. armigera*-*H. zea* hybrids from North America (HZRL10, HZRL12, HZRL17, HZRL20), with predicted *H. armigera* proportions of ​​12.6%, 18.1%, 12% and 15%, respectively (**Table 1**) is interesting, given that *H. armigera* has not been formally identified in the mainland US, and that *H. armigera* was first detected in the Americas in 2013 in Brazil (2). This may therefore represent an early presence of *H. armigera* in the Americas.

The samples were originally described in a 2007 study that constructed a phylogeny of *Helicoverpa* spp. using mitochondrial DNA (11), and their genome sequences, used in the study described here, were described in (13). The samples are recorded as having been collected from ‘Riverland, NY’ (13), however this location is unclear. Dr Daniel Gilrein supplied the *H. zea* samples (11), and is based at the Long Island Horticultural Research and Extension Center (LIHREC), Riverhead, NY. The origin of the samples is confirmed as Riverhead, NY (personal communication, Dr Dan Gilrein).

The four samples were collected in 2005, in September/October (personal communication, Dr Dan Gilrein). Significantly, this date predates the first reports of *H. armigera* in the New World in 2013 in Brazil (2). In order to confirm this result, 8511 AIMs were identified, as described in Methods. Unsupervised clustering allowed the *a priori* identification of 34 *H. armigera* and 7 *H. zea* non-hybridized genomes (**Table 1**). These were used to identify SNPs that preferentially segregate in one species or the other (AIMS) (**Supplementary Material**). The 8511 AIMS thus identified indicate a *H. armigera* component ranging from 25.8 to 31.1% in the four genomes (**Table 3**). The predicted presence of a *H. armigera* component is consistent with the results from the Admixture analysis.

**Table 3:** Proportion of *H. zea* AIMs present in different genomic datasets.

Species assignment	Sample	Proportion of *H. zea* specific AIMs (%)	Average sequence depth of the 8511 AIMS
*H. armigera*-*H. zea* F1 hybrid	PRh	65.2	102.3
*H. armigera*-*H. zea* F1 hybrid	MAh	61.1	107.2
*H. armigera*	HaM	0.6	98.8
*H. zea*	HzF	100	107.0
*H. zea*	HzCol	100	85.7
*H. zea*	HzIll	100	83.1
*H. zea*	HzMaine	100	86.3
*H.zee*	HzNC	100	25.7
*H. zea*	70	100	23.7
*H. zea*	73	100	29.1
*H. zea*	132	61.0	10.1
*H. zea*	133	52.2	8.7
*H. zea*	134	66.9	11.9
*H. zea/hybrid*	TMG4	66.0	76.1
*H. zea*	HZRL10	73.7	21.8
*H. zea*	HZRL12	69.9	14.9
*H. zea*	HZRL17	74.2	17.2
*H. zea*	HZRL20	70.9	21.9
*H. armigera*	M0086	0.6	39.7
*H. armigera*	M0118	0.6	26.1
*H. armigera*	M0237	0.6	30.7
*H. armigera*	M0260	0.6	34.7
*H. armigera*	7	0.6	14.5
*H. armigera*	8	0.6	18.1
*H. armigera*	10	0.6	12.2
*H. armigera*	12	0.6	16.4
*H. armigera*	FMM1.3	0.6	11.7
*H. armigera*	FMM1.2	0.6	9.0
*H. armigera*	FMM1.4	0.6	13.3
*H. armigera*	738	0.6	24.9
*H. armigera*	I3	0.6	16.2
*H. armigera*	ICY5L	0.6	14.2
*H. armigera*	MAD13	0.6	13.5
*H. armigera*	MAD20	0.6	14.1
*H. armigera*	MAD3	0.6	15.3
*H. armigera*	MAD5	0.6	9.9
*H. armigera*	NZ24	0.6	10.7
*H. armigera*	NZ27	0.6	28.0
*H. armigera*	NZ29	0.6	16.0
*H. armigera*	SEN2	0.6	12.7
*H. armigera*	SEN6	0.6	16.1
*H. armigera*	SEN8	0.6	22.5
*H. armigera*	S.5	0.6	15.2
*H. armigera*	UG32L	0.6	19.7
*H. armigera*	UG37L	0.6	13.8
*H. armigera*	UG38L	0.6	18.8
*H. armigera*	UG39L	0.6	15.3
*H. armigera*/hybrid	110	11.4	49.6
*H. armigera*/hybrid	125	3.4	11.8
*H. armigera*/hybrid	131	0.6	16.4
*H. armigera*/hybrid	142	11.1	33.5
*H. armigera*/hybrid	144	0.6	14.2
*H. armigera/*hybrid	BRA2	0.6	23.0
*H. armigera/*hybrid	BRA4	4.6	21.8
*H. armigera/*hybrid	TPG2	0.6	19.8

8511 AIMs were identified as described in Methods. The proportion of *H. armigera* - specific AIMs identified in the different genomes is listed. The proportions were determined by comparison with the filtered SNPs produced from the SNP calling procedure described in Methods.

Regarding the accuracy of this approach, using a reduced set of SNPs is not expected to give the same accuracy as the whole genome considerations utilized by Admixture, however the unsupervised clustering approach represents an independent manner of assessing a potential *H. armigera* contribution to *H. zea* genomic datasets. The *H. armigera* component is higher than predicted by the Admixture approach, which gives 12.6 to 18.1% *H. armigera*. Notably, the predicted *H. armigera* proportion for the two F1 hybrids is 34.8% (PRh) and 38.9% (MAh) (**Table 3**), which underestimates the true proportion of 50%. One potential source of error is uneven distribution of AIMs along the chromosomes. Another is that the *H. zea* dataset was limited in size, and so this reduces the accuracy in identifying species-specific AIMs. The low level (0.6%) of *H. zea* AIMs detected in most of the *H. armigera* genomes reflects the AIM selection approach: the *H. zea* AIMs were present in all 7 *H. zea* genomes, and were also found to be present in at most one *H. armigera* genome in the reference dataset.

Finally, it is possible that low sequencing depth may affect the predicted hybridization proportions. Given that the SNPs are called against a *H. armigera* reference genome, then if a SNP position has low or no read depth in a particular genome, the SNP calling software will call the *H. armigera* genotype at that position. This means a bias toward calling *H. armigera* AIMs when sequencing depth is low. For example, HZRL10 has 333 AIM positions where there is no sequence coverage, reflecting its low average sequencing depth of 21.8 for the AIM positions. In total there are 986 AIM positions where DP < 5, and so cannot be called with confidence; these constitute 11% of the total number of AIMs. The AIM positions where there is no sequence coverage are by default identified as *H. armigera* (reflecting the reference genome sequence at those positions). This therefore can account for a proportion of *H. armigera* AIMs in the HZRL10 genome sequence, but not all. This observation may also account for a proportion of the *H. armigera* ancestry in HZRL10 detected by the Admixture analysis.

Further work will be required to validate or discount these observations. In particular, the approaches described are not able to distinguish sample contamination from hybridization. Larger, high quality datasets will be necessary in order to distinguish these two alternative scenarios. Development of such fine-grained methods will have value in screening of historic samples and detection of contamination in hybridization studies. These are currently difficult to detect (a method developed by SEM for detecting contamination of NGS datasets, mitoscan https://github.com/semassey/Scanning-NGS-datasets-for-mitochondrial-and-coronavirus-contaminants/blob/main/mitoscan.sh, maps reads against all NCBI mitochondrial genomes, however it is not able to distinguish contamination by closely related species, due to cross-mapping between closely related mitochondria).

### The use of genome admixture analysis for the identification and control of *Helicoverpa* infestations

We have shown the efficacy of genome admixture analysis for verifying the identity of *Helicoverpa* hybrids, which are morphologically cryptic, and so recalcitrant to traditional identification methods, as is the identification of the two *Helicoverpa* species themselves. We found that increasing the number of *H. zea* genomes in the analysis improved the accuracy of admixture prediction, for the *H. zea* and *H. armigera* genomes, and the two F1 hybrid genomes generated in the study. Likewise, filtering based on sequencing depth also had a similar effect, although we were restricted in increasing filtering stringency, given limitations in sequencing depth in the dataset. Future improvements in accuracy will arise from greater average sequencing depth in the reference genomes used in admixture analyses. Finally, for accurate hybrid identification, whole genome approaches are most likely to yield the precision necessary for understanding the dynamics of *H. armigera* invasivity in the field.

In addition to the indirect detection of *H. armigera* in a region via identification of *H. armigera*-*H. zea* hybrids, determining the presence of the hybrids will have utility for monitoring the occurrence and spread of pesticide resistance. This is desirable because *H. armigera* populations in the Old World have typically been subjected to significant pesticide exposure, thus leading to the evolution of resistance (15). Hybridization with local *H. zea* populations is expected to lead to the introgression of pesticide resistance genes from the *H. armigera* genomic component (15). The phenomenon of rapid introgression of pesticide resistance genes between sister species has been observed in *Anopheles* spp. exposed to selection pressure from pesticide exposure (34). The evolutionary dynamics would be expected to be rather similar in crop pests such as *Helicoverpa* spp.

Host plant preference is another agriculturally relevant phenotype that may be influenced by hybridization and gene introgression is that of host plant preference. *H. armigera* has a considerably more extensive plant host range than *H. zea*, apparently partly due to its larger number of gustatory receptor and detoxification genes compared to *H. zea* (21). Adaptive introgression of these genes from *H. armigera* into local populations of *H. zea* may cause changes in the host plant preferences of *H. zea*, a process consistent with the ‘hybrid bridge’ hypothesis of host shifting of herbivorous insect pests (35). Furthermore, increasing ease of *H. armigera-H. zea* hybrid detection will allow for the collection of empirical evidence for whether hybridization will influence changes in pesticide susceptibility or feeding behavior. Currently, because hybrids are extremely difficult to identify, empirical data for these phenotypic changes are near impossible to collect.

Puerto Rico is a stepping stone between North and South America, given its geographic location and possession of a major port in San Juan, through which agricultural produce enters and exits the United States. This transit route for agricultural pests and pathogens comprises part of a ‘Caribbean corridor’. So far, there are no reports in the literature on sustained *H. armigera* populations in North America or Puerto Rico. One potential route for the spread of *H. armigera* into North America from South America may be through Puerto Rico.

The detection of *H. armigera-zea* hybrids can reveal aspects of the population dynamics of both species and help inform control strategies. The accurate determination of hybrid proportions can also indicate whether species boundaries are maintained, given that hybridization is often maladaptive.

Accurate admixture prediction methods for *Helicoverpa* species are essential for the design of accurate high throughput hybrid identification tools, and so the datasets generated as part of this study will be useful in the development of tools for the rapid, economical and accurate identification of pure species or hybrids. Future detection of hybrids from Puerto Rico and potentially North America will help inform control regimens, facilitated by the development of rapid molecular tests to accurately determine hybrids. In particular, if there is detection of *H. armigera* in North America, screening of local *H. zea* populations for hybridization could be used to assess whether breeding has occurred.

## Data availability statement

The datasets presented in this study can be found in online repositories. The names of the repository/repositories and accession number(s) can be found in the article/**Supplementary Material**.

## Ethics statement

The manuscript presents research on animals that do not require ethical approval for their study.

## Author contributions

DT: Investigation, Writing – review & editing. TM: Investigation, Resources, Writing – review & editing. CE: Investigation, Funding acquisition, Project administration, Writing – review & editing. JV: Funding acquisition, Investigation, Project administration, Writing – review & editing, Conceptualization, Resources, Methodology. RL: Writing – review & editing, Visualization, Validation. SM: Writing – review & editing, Conceptualization, Data curation, Formal Analysis, Funding acquisition, Investigation, Methodology, Project administration, Software, Supervision, Validation, Writing – original draft.
