# Supplementary material for: Accurate identification of Helicoverpa armigera–Helicoverpa zea hybrids using genome admixture analysis: implications for genomic surveillance
Source: Front Insect Sci. 2024 Feb 23;4:1339143. doi: 10.3389/finsc.2024.1339143 (PMC10926370; doi:10.3389/finsc.2024.1339143)
Supplement: Supplementary file 1 [file DataSheet_1.docx]

2 6479 . T C 999 . . GT:PL:DP 0/1:255,0,100:48 0/1:255,0,60:47 0/1:233,0,65:33 0/1:161,0,59:15 0/1:16,0,22:4 0/1:100,0,38:11 0/1:251,0,164:99

2 6481 . A C 999 . . GT:PL:DP 0/1:255,0,121:49 0/1:255,0,62:47 0/1:235,0,76:34 0/1:155,0,57:15 0/1:16,0,22:4 0/1:87,0,40:10 0/1:252,0,171:101

2 6975 . T G 999 . . GT:PL:DP 0/1:241,0,22:22 0/1:255,0,37:33 0/1:99,0,79:11 0/1:57,6,0:2 0/1:142,0,26:8 0/1:44,0,58:5 0/1:255,0,65:65

2 7519 . T C 999 . . GT:PL:DP 0/1:255,0,85:40 0/1:176,0,229:61 0/1:99,0,246:57 0/1:93,0,71:9 0/1:51,0,44:6 0/1:225,0,88:26 0/1:232,0,199:72

2 7538 . G C 999 . . GT:PL:DP 0/1:253,0,99:43 0/1:203,0,213:60 0/1:83,0,247:51 0/1:57,0,74:8 0/1:43,0,44:6 0/1:196,0,88:26 0/1:230,0,193:67

2 7740 . A C 999 . . GT:PL:DP 0/1:177,0,129:48 0/1:120,0,235:60 0/1:77,0,241:62 0/1:76,2,0:7 0/1:16,0,150:11 0/1:55,0,127:18 0/1:209,0,161:68

2 7803 . G A 999 . . GT:PL:DP 0/1:166,0,171:28 0/1:10,0,222:26 0/1:137,0,255:53 0/1:21,6,0:2 0/1:36,0,52:7 0/1:135,0,154:26 0/1:223,0,160:57

2 7852 . C A 999 . . GT:PL:DP 0/1:210,0,32:28 0/1:143,0,169:31 0/1:181,0,192:42 0/1:30,3,0:1 0/1:50,0,5:8 0/1:142,0,35:25 0/1:227,0,61:54

2 7874 . G C 999 . . GT:PL:DP 0/1:245,0,45:42 0/1:164,0,114:38 0/1:220,0,126:40 0/1:30,3,0:1 0/1:53,3,0:7 0/1:118,0,26:23 0/1:241,0,90:50

2 8097 . A G 999 . . GT:PL:DP 0/1:255,0,117:38 0/1:233,0,211:44 0/1:249,0,161:39 0/1:55,0,22:3 0/1:22,0,39:3 0/1:129,0,26:8 0/1:255,0,113:58

2 8359 . A G 999 . . GT:PL:DP 0/1:99,0,120:20 0/1:89,0,141:29 0/1:47,0,171:29 0/1:9,0,43:3 0/1:16,0,56:5 0/1:52,0,74:9 0/1:183,0,142:43

2 13514 . A G 999 . . GT:PL:DP 0/1:76,0,71:34 0/1:37,0,119:34 0/1:58,0,163:47 0/1:21,0,73:10 0/1:49,0,11:7 0/1:78,0,49:9 0/1:85,0,190:49

2 51418 . C A 999 . . GT:PL:DP 0/1:49,0,255:207 0/1:141,0,255:79 0/1:255,0,255:97 0/1:115,0,211:38 0/1:68,0,255:35 0/1:15,0,255:28 0/1:170,0,255:162

2 51486 . A G 999 . . GT:PL:DP 0/1:255,0,255:214 0/1:255,0,255:75 0/1:177,0,255:95 0/1:37,0,255:32 0/1:248,0,255:81 0/1:255,0,238:44 0/1:140,0,255:180

2 130014 . C T 999 . . GT:PL:DP 0/1:255,0,237:144 0/1:255,0,172:163 0/1:255,0,255:161 0/1:80,0,155:27 0/1:209,0,60:43 0/1:226,0,69:52 0/1:255,0,240:227

2 130029 . A T 999 . . GT:PL:DP 0/1:165,0,255:145 0/1:32,0,255:159 0/1:150,0,255:156 0/1:145,0,157:27 0/1:45,0,227:40 0/1:12,0,253:52 0/1:131,0,255:223

2 278695 . A G 999 . . GT:PL:DP 0/1:81,0,129:34 0/1:53,0,159:28 0/1:24,0,175:27 0/1:62,0,47:9 0/1:17,0,41:4 0/1:10,0,68:5 0/1:46,0,207:58

2 278718 . G A 999 . . GT:PL:DP 0/1:133,0,105:43 0/1:145,0,82:32 0/1:151,0,70:29 0/1:44,0,77:10 0/1:66,0,16:5 0/1:68,0,10:5 0/1:197,0,76:68

2 592080 . C T 999 . . GT:PL:DP 0/1:214,0,255:237 0/1:189,0,255:232 0/1:242,0,255:230 0/1:174,0,255:57 0/1:161,0,180:26 0/1:37,0,231:26 0/1:255,0,255:237

2 592259 . C T 999 . . GT:PL:DP 0/1:60,0,209:232 0/1:9,0,159:192 0/1:70,0,156:188 0/1:66,0,151:28 0/1:33,0,24:2 0/1:44,0,89:10 0/1:126,0,230:231

2 633923 . G C 999 . . GT:PL:DP 0/1:165,0,54:23 0/1:236,0,107:33 0/1:111,0,194:19 0/1:27,0,49:4 0/1:63,0,56:7 0/1:17,0,51:6 0/1:133,0,201:39

2 731847 . A G 999 . . GT:PL:DP 0/1:194,0,255:86 0/1:161,0,255:92 0/1:124,0,255:69 0/1:121,0,215:20 0/1:33,0,255:50 0/1:54,0,219:21 0/1:101,0,255:174

2 731919 . C A 999 . . GT:PL:DP 0/1:110,0,255:62 0/1:198,0,255:75 0/1:108,0,255:57 0/1:26,0,255:15 0/1:23,0,184:35 0/1:65,0,14:13 0/1:229,0,255:175

2 731923 . C T 999 . . GT:PL:DP 0/1:135,0,255:58 0/1:214,0,255:74 0/1:137,0,255:57 0/1:20,0,250:17 0/1:53,0,194:44 0/1:82,0,59:14 0/1:237,0,255:176

2 763261 . G A 999 . . GT:PL:DP 0/1:255,0,255:206 0/1:255,0,255:214 0/1:255,0,255:219 0/1:255,0,207:39 0/1:255,0,236:60 0/1:255,0,255:46 0/1:255,0,255:209

2 763515 . T C 999 . . GT:PL:DP 0/1:87,0,255:210 0/1:131,0,255:206 0/1:144,0,255:204 0/1:143,0,255:46 0/1:69,0,255:62 0/1:105,0,255:54 0/1:78,0,255:209

2 796005 . A G 999 . . GT:PL:DP 0/1:61,0,188:228 0/1:15,0,135:226 0/1:112,0,236:225 0/1:72,0,148:59 0/1:23,0,54:35 0/1:58,0,116:86 0/1:115,0,210:281

2 819912 . G A 999 . . GT:PL:DP 0/1:245,0,195:59 0/1:123,0,150:42 0/1:150,0,233:47 0/1:9,0,85:5 0/1:17,0,48:6 0/1:12,4,109:21 0/1:255,0,209:89

2 845089 . C T 999 . . GT:PL:DP 0/1:175,0,255:192 0/1:159,0,255:226 0/1:212,0,255:204 0/1:92,0,255:30 0/1:24,0,72:11 0/1:40,0,179:48 0/1:158,0,255:218

2 845996 . A G 999 . . GT:PL:DP 0/1:76,0,255:130 0/1:71,0,255:178 0/1:193,0,255:167 0/1:58,0,255:27 0/1:109,0,131:11 0/1:109,0,255:45 0/1:105,0,255:166

2 858904 . T A 999 . . GT:PL:DP 0/1:83,0,150:82 0/1:235,0,149:60 0/1:176,0,142:75 0/1:52,0,168:22 0/1:126,0,45:20 0/1:141,0,89:30 0/1:241,0,220:180

2 858917 . G A 999 . . GT:PL:DP 0/1:105,0,168:77 0/1:255,0,187:71 0/1:202,0,153:70 0/1:62,0,138:19 0/1:128,0,23:24 0/1:164,0,115:36 0/1:252,0,209:174

2 858993 . G A 999 . . GT:PL:DP 0/1:209,0,243:82 0/1:255,0,225:90 0/1:243,0,231:90 0/1:74,0,76:17 0/1:82,0,65:29 0/1:113,0,85:33 0/1:255,0,198:168

2 858994 . G T 999 . . GT:PL:DP 0/1:213,0,235:83 0/1:255,0,252:90 0/1:224,0,236:93 0/1:57,0,110:17 0/1:73,0,87:28 0/1:103,0,105:33 0/1:255,0,216:170

2 859011 . T A 999 . . GT:PL:DP 0/1:193,0,248:83 0/1:255,0,255:90 0/1:237,0,245:96 0/1:42,0,158:18 0/1:75,0,77:25 0/1:64,0,86:30 0/1:255,0,255:173

2 873940 . A T 999 . . GT:PL:DP 0/1:195,0,255:29 0/1:255,0,255:41 0/1:255,0,255:32 0/1:133,0,53:7 0/1:40,3,0:1 0/1:90,0,25:4 0/1:255,0,255:66

2 880232 . T A 999 . . GT:PL:DP 0/1:255,0,255:129 0/1:255,0,214:121 0/1:255,0,222:119 0/1:235,0,209:48 0/1:52,0,127:32 0/1:102,0,144:69 0/1:226,0,190:116

2 880235 . G C 999 . . GT:PL:DP 0/1:171,0,255:208 0/1:206,0,255:205 0/1:173,0,255:199 0/1:117,0,255:80 0/1:52,0,162:46 0/1:51,0,184:90 0/1:139,0,255:188

2 880320 . T A 999 . . GT:PL:DP 0/1:182,0,255:220 0/1:255,0,255:235 0/1:255,0,255:223 0/1:222,0,255:163 0/1:194,0,255:103 0/1:146,0,255:142 0/1:197,0,255:217

2 880361 . G A 999 . . GT:PL:DP 0/1:183,0,255:232 0/1:199,0,255:235 0/1:138,0,255:227 0/1:176,0,255:148 0/1:74,0,241:63 0/1:146,0,255:64 0/1:108,0,255:217

2 880622 . G A 999 . . GT:PL:DP 0/1:211,0,220:208 0/1:193,0,230:206 0/1:186,0,232:195 0/1:178,0,142:83 0/1:191,0,142:36 0/1:157,0,98:31 0/1:255,0,242:177

2 880667 . C T 999 . . GT:PL:DP 0/1:214,0,198:132 0/1:204,0,202:140 0/1:127,0,130:146 0/1:200,0,122:57 0/1:161,0,127:29 0/1:132,0,97:32 0/1:222,0,209:155

2 1088942 . A T 999 . . GT:PL:DP 0/1:186,0,253:231 0/1:247,0,218:240 0/1:216,0,255:224 0/1:255,0,255:139 0/1:223,0,217:86 0/1:199,0,153:99 0/1:255,0,255:219

2 1089037 . C T 999 . . GT:PL:DP 0/1:255,0,182:233 0/1:255,0,173:236 0/1:255,0,255:236 0/1:255,0,184:108 0/1:102,0,102:41 0/1:88,0,85:50 0/1:255,0,242:223

2 1089096 . C G 999 . . GT:PL:DP 0/1:255,0,172:211 0/1:255,0,204:221 0/1:255,0,255:196 0/1:255,0,144:79 0/1:41,0,153:22 0/1:15,0,64:13 0/1:255,0,255:209

2 1089209 . C T 999 . . GT:PL:DP 0/1:181,0,170:240 0/1:164,0,124:241 0/1:151,0,195:236 0/1:197,0,199:120 0/1:63,0,164:50 0/1:12,0,98:99 0/1:255,0,237:226

2 1209836 . A T 999 . . GT:PL:DP 0/1:160,0,227:50 0/1:214,0,208:38 0/1:227,0,147:42 0/1:21,0,140:15 0/1:98,0,12:13 0/1:99,0,41:21 0/1:159,0,232:59

2 1344962 . T A 999 . . GT:PL:DP 0/1:32,0,207:53 0/1:160,0,228:42 0/1:119,0,189:54 0/1:15,0,67:5 0/1:27,0,97:12 0/1:17,0,7:3 0/1:97,0,255:119

2 1344965 . A T 999 . . GT:PL:DP 0/1:53,0,209:54 0/1:164,0,221:42 0/1:122,0,194:54 0/1:15,0,76:5 0/1:27,0,97:12 0/1:33,3,0:3 0/1:97,0,255:122

2 1345058 . C G 999 . . GT:PL:DP 0/1:153,0,147:46 0/1:135,0,130:35 0/1:172,0,167:69 0/1:37,0,87:9 0/1:32,0,116:21 0/1:75,0,108:18 0/1:155,0,209:117

2 1520624 . C T 999 . . GT:PL:DP 0/1:83,0,173:43 0/1:175,0,236:63 0/1:101,0,205:59 0/1:102,0,216:17 0/1:14,7,0:10 0/1:56,0,18:13 0/1:118,0,251:70

2 1630956 . G C 999 . . GT:PL:DP 0/1:255,0,70:40 0/1:255,0,45:42 0/1:255,0,88:45 0/1:136,0,69:9 0/1:106,0,69:13 0/1:86,0,50:9 0/1:255,0,136:44

2 1631553 . A T 999 . . GT:PL:DP 0/1:193,0,123:28 0/1:226,0,113:40 0/1:214,0,139:65 0/1:153,0,38:12 0/1:40,6,0:2 0/1:77,6,0:6 0/1:227,0,127:45

2 1631869 . G A 999 . . GT:PL:DP 0/1:29,0,220:72 0/1:142,0,205:51 0/1:51,0,243:95 0/1:62,0,120:19 0/1:9,0,144:10 0/1:73,0,201:24 0/1:68,0,255:85

2 1643334 . T C 999 . . GT:PL:DP 0/1:113,0,234:58 0/1:95,0,187:36 0/1:99,0,225:28 0/1:42,0,80:8 0/1:40,0,120:16 0/1:48,0,53:9 0/1:116,0,223:50

2 1643336 . G C 999 . . GT:PL:DP 0/1:106,0,239:58 0/1:100,0,190:34 0/1:72,0,222:26 0/1:39,0,80:8 0/1:33,0,122:15 0/1:42,0,59:7 0/1:126,0,223:50

2 1645087 . T A 999 . . GT:PL:DP 0/1:100,0,72:21 0/1:97,0,69:19 0/1:149,0,50:18 0/1:39,6,0:2 0/1:94,0,38:9 0/1:93,0,29:7 0/1:137,0,61:27

2 1656849 . A C 999 . . GT:PL:DP 0/1:108,0,95:25 0/1:71,0,91:19 0/1:59,0,62:17 0/1:22,0,19:5 0/1:68,0,57:10 0/1:68,0,45:10 0/1:92,0,126:41

2 1656950 . T A 999 . . GT:PL:DP 0/1:135,0,155:24 0/1:103,0,45:12 0/1:109,0,81:24 0/1:28,0,18:3 0/1:103,0,32:16 0/1:55,0,138:14 0/1:153,0,172:50

2 1656998 . C G 999 . . GT:PL:DP 0/1:105,0,108:21 0/1:119,0,75:17 0/1:114,0,119:26 0/1:66,0,37:6 0/1:36,0,33:9 0/1:87,0,10:10 0/1:118,0,166:55

2 1657025 . C T 999 . . GT:PL:DP 0/1:71,0,49:18 0/1:141,0,31:19 0/1:108,0,52:22 0/1:52,0,27:6 0/1:28,0,12:8 0/1:12,0,15:8 0/1:123,0,82:41

2 1661900 . G T 999 . . GT:PL:DP 0/1:214,0,179:38 0/1:110,0,176:20 0/1:231,0,228:38 0/1:34,0,72:6 0/1:62,0,130:13 0/1:109,0,112:15 0/1:223,18,154:59

2 1661946 . T C 999 . . GT:PL:DP 0/1:163,0,208:34 0/1:128,0,138:20 0/1:158,0,237:36 0/1:22,0,44:6 0/1:37,0,129:12 0/1:45,0,95:9 0/1:142,0,221:55

2 1675893 . G A 999 . . GT:PL:DP 0/1:226,0,255:142 0/1:136,0,255:117 0/1:135,0,255:166 0/1:15,0,255:30 0/1:8,0,162:33 0/1:22,0,229:72 0/1:49,0,220:186

2 1692897 . G T 999 . . GT:PL:DP 0/1:89,0,58:25 0/1:118,0,102:37 0/1:122,0,10:20 0/1:51,0,10:5 0/1:36,0,5:5 0/1:19,0,6:4 0/1:158,0,5:35

2 1692945 . C G 999 . . GT:PL:DP 0/1:107,0,59:30 0/1:155,0,119:50 0/1:148,0,67:38 0/1:69,0,47:7 0/1:24,0,16:5 0/1:60,0,18:9 0/1:188,0,6:46

2 1692946 . A T 999 . . GT:PL:DP 0/1:107,0,59:30 0/1:157,0,118:51 0/1:148,0,68:38 0/1:72,0,47:7 0/1:23,0,14:6 0/1:60,0,18:9 0/1:191,0,22:47

2 1692952 . G A 999 . . GT:PL:DP 0/1:110,0,84:37 0/1:158,0,102:52 0/1:154,0,70:44 0/1:72,0,39:7 0/1:24,0,15:5 0/1:60,0,18:9 0/1:189,0,79:49

2 1692956 . C G 999 . . GT:PL:DP 0/1:112,0,69:46 0/1:165,0,110:59 0/1:148,0,74:50 0/1:86,0,42:9 0/1:44,0,11:7 0/1:68,0,12:11 0/1:178,0,84:60

2 1693009 . C G 999 . . GT:PL:DP 0/1:141,0,174:51 0/1:186,0,158:71 0/1:187,0,139:57 0/1:97,0,116:13 0/1:81,0,15:8 0/1:75,0,33:9 0/1:188,0,194:71

2 1693014 . G C 999 . . GT:PL:DP 0/1:138,0,179:53 0/1:184,0,172:73 0/1:196,0,143:59 0/1:111,0,114:14 0/1:79,0,19:9 0/1:75,0,33:9 0/1:197,0,217:78

2 1693390 . A G 999 . . GT:PL:DP 0/1:255,0,255:104 0/1:255,0,255:110 0/1:255,0,255:114 0/1:196,0,102:17 0/1:223,0,244:47 0/1:217,0,255:65 0/1:255,0,255:145

2 1699432 . C G 999 . . GT:PL:DP 0/1:87,0,255:37 0/1:99,0,180:23 0/1:92,0,182:28 0/1:19,0,123:6 0/1:41,0,73:7 0/1:71,0,195:14 0/1:174,0,255:52

2 1700208 . G T 999 . . GT:PL:DP 0/1:255,0,201:68 0/1:255,0,179:71 0/1:255,0,174:73 0/1:76,0,57:6 0/1:158,0,128:16 0/1:144,0,205:21 0/1:255,0,179:110

2 1700223 . G A 999 . . GT:PL:DP 0/1:255,0,200:72 0/1:255,0,187:71 0/1:255,0,151:80 0/1:115,0,62:9 0/1:174,0,163:19 0/1:131,0,251:26 0/1:255,0,186:109

2 1700258 . T C 999 . . GT:PL:DP 0/1:255,0,175:74 0/1:255,0,147:80 0/1:255,0,147:88 0/1:142,0,35:10 0/1:109,0,255:20 0/1:20,0,255:31 0/1:255,0,176:108

2 1704427 . A G 999 . . GT:PL:DP 0/1:255,0,255:36 0/1:213,0,255:37 0/1:236,0,220:32 0/1:36,0,171:11 0/1:148,0,19:8 0/1:32,0,137:8 0/1:255,0,255:65

2 1712434 . T G 999 . . GT:PL:DP 0/1:148,0,53:25 0/1:185,0,93:28 0/1:156,0,112:25 0/1:25,3,0:1 0/1:37,0,28:5 0/1:88,0,71:13 0/1:191,0,131:35

2 1762644 . A T 999 . . GT:PL:DP 0/1:106,0,255:40 0/1:129,0,255:47 0/1:101,0,255:55 0/1:31,0,99:9 0/1:89,0,22:4 0/1:51,0,26:3 0/1:243,0,255:49

2 1763382 . A G 999 . . GT:PL:DP 0/1:255,0,63:42 0/1:255,0,255:61 0/1:238,0,255:42 0/1:159,0,3:8 0/1:83,0,109:9 0/1:61,0,220:15 0/1:255,0,255:54

2 1763843 . A T 999 . . GT:PL:DP 0/1:132,0,255:78 0/1:208,0,255:46 0/1:45,0,255:64 0/1:28,0,182:16 0/1:32,0,126:11 0/1:78,0,150:13 0/1:205,0,255:84

2 1763997 . C T 999 . . GT:PL:DP 0/1:255,0,255:60 0/1:255,0,194:37 0/1:255,0,255:81 0/1:230,0,120:18 0/1:82,0,106:7 0/1:54,0,107:6 0/1:255,0,255:97

2 1764056 . A C 999 . . GT:PL:DP 0/1:255,0,255:67 0/1:255,0,138:50 0/1:255,0,255:59 0/1:182,0,119:14 0/1:117,0,47:6 0/1:24,0,119:5 0/1:255,0,255:92

2 1828984 . G C 999 . . GT:PL:DP 0/1:127,0,238:34 0/1:236,0,126:29 0/1:136,0,194:29 0/1:41,0,84:6 0/1:67,0,27:4 0/1:80,0,104:11 0/1:129,0,159:20

2 1888439 . C T 999 . . GT:PL:DP 0/1:235,0,255:199 0/1:125,0,255:206 0/1:73,0,238:183 0/1:144,0,255:59 0/1:67,0,113:10 0/1:82,0,199:35 0/1:113,0,255:203

2 1888752 . T C 999 . . GT:PL:DP 0/1:136,0,234:130 0/1:230,0,255:149 0/1:255,0,255:149 0/1:102,0,212:30 0/1:46,0,76:6 0/1:94,0,53:8 0/1:241,0,247:138

2 1888774 . G A 999 . . GT:PL:DP 0/1:110,0,218:148 0/1:234,0,255:175 0/1:255,0,255:161 0/1:93,0,130:21 0/1:38,0,113:9 0/1:99,0,55:11 0/1:218,0,243:175

2 1956256 . G T 999 . . GT:PL:DP 0/1:110,0,15:20 0/1:123,0,84:42 0/1:43,0,94:29 0/1:45,1,0:5 0/1:30,0,26:6 0/1:37,0,53:8 0/1:71,0,134:36

2 1956259 . G T 999 . . GT:PL:DP 0/1:112,0,15:20 0/1:106,0,93:35 0/1:44,0,89:29 0/1:13,1,7:3 0/1:30,0,26:6 0/1:37,0,53:8 0/1:57,0,131:34

2 2309219 . T A 999 . . GT:PL:DP 0/1:184,0,160:21 0/1:135,0,55:21 0/1:192,0,186:37 0/1:20,0,54:3 0/1:116,0,90:8 0/1:57,0,120:8 0/1:160,0,253:33

2 2313667 . T C 999 . . GT:PL:DP 0/1:159,0,255:215 0/1:215,0,255:243 0/1:169,0,255:226 0/1:173,0,242:47 0/1:116,0,253:55 0/1:23,0,255:38 0/1:137,0,255:241

2 2313692 . G A 999 . . GT:PL:DP 0/1:242,0,255:225 0/1:252,0,255:233 0/1:216,0,255:217 0/1:206,0,206:49 0/1:118,0,238:55 0/1:43,0,201:32 0/1:255,0,255:236

2 2313709 . G A 999 . . GT:PL:DP 0/1:255,0,255:222 0/1:255,0,255:233 0/1:251,0,255:218 0/1:215,0,211:53 0/1:89,0,255:68 0/1:78,0,234:42 0/1:255,0,255:241

2 2313719 . G T 999 . . GT:PL:DP 0/1:254,0,255:226 0/1:255,0,255:231 0/1:255,0,255:217 0/1:203,0,183:50 0/1:66,0,255:86 0/1:75,0,245:43 0/1:255,0,255:236

2 2313798 . T C 999 . . GT:PL:DP 0/1:230,0,255:216 0/1:247,0,255:240 0/1:231,0,255:216 0/1:230,0,255:74 0/1:67,0,255:85 0/1:116,0,252:40 0/1:245,0,255:237

2 2313929 . T A 999 . . GT:PL:DP 0/1:255,0,255:204 0/1:255,0,255:231 0/1:255,0,255:211 0/1:212,0,255:74 0/1:57,0,255:90 0/1:72,0,255:57 0/1:204,0,255:237

2 2313932 . T C 999 . . GT:PL:DP 0/1:255,0,255:210 0/1:255,0,255:237 0/1:255,0,255:214 0/1:233,0,255:74 0/1:92,0,255:91 0/1:79,0,255:57 0/1:206,0,255:241

2 2314267 . A G 999 . . GT:PL:DP 0/1:254,0,255:209 0/1:255,0,255:240 0/1:255,0,255:232 0/1:255,0,255:78 0/1:252,0,255:56 0/1:45,0,255:53 0/1:255,0,255:228

2 2415855 . T G 999 . . GT:PL:DP 0/1:169,1,0:20 0/1:125,0,45:16 0/1:42,0,34:8 0/1:30,3,0:1 0/1:11,0,56:7 0/1:76,0,77:17 0/1:133,0,43:28

2 2491989 . C A 999 . . GT:PL:DP 0/1:248,0,87:56 0/1:195,0,138:45 0/1:241,0,122:50 0/1:23,0,123:9 0/1:25,0,27:8 0/1:48,6,0:2 0/1:250,0,255:91

2 2558967 . C T 999 . . GT:PL:DP 0/1:185,0,255:70 0/1:144,0,255:93 0/1:196,0,249:80 0/1:33,0,97:25 0/1:85,0,236:30 0/1:138,0,214:31 0/1:169,0,255:59

2 2559152 . G T 999 . . GT:PL:DP 0/1:254,0,255:219 0/1:225,0,248:212 0/1:255,0,255:170 0/1:178,0,160:37 0/1:251,0,229:51 0/1:255,0,243:68 0/1:221,0,255:243

2 2559243 . G A 999 . . GT:PL:DP 0/1:197,0,203:113 0/1:143,0,59:114 0/1:198,0,90:114 0/1:120,0,149:27 0/1:193,0,60:80 0/1:222,0,85:77 0/1:182,0,255:165

2 2559263 . G T 999 . . GT:PL:DP 0/1:182,0,178:102 0/1:129,0,21:102 0/1:189,0,94:103 0/1:96,0,115:22 0/1:119,0,21:54 0/1:120,0,81:51 0/1:183,0,255:146

2 2559307 . A T 999 . . GT:PL:DP 0/1:196,0,175:73 0/1:200,0,146:72 0/1:212,0,152:66 0/1:79,0,67:10 0/1:85,0,6:23 0/1:77,0,40:26 0/1:194,0,255:105

2 2559429 . A G 999 . . GT:PL:DP 0/1:255,0,213:112 0/1:255,0,162:137 0/1:255,0,204:106 0/1:127,0,39:17 0/1:255,0,146:77 0/1:245,0,131:63 0/1:199,0,183:116

2 2559677 . T A 999 . . GT:PL:DP 0/1:255,0,225:62 0/1:255,0,199:42 0/1:255,0,52:39 0/1:31,0,32:3 0/1:32,4,0:4 0/1:17,1,0:14 0/1:255,0,223:44

2 2560111 . G C 999 . . GT:PL:DP 0/1:228,0,255:223 0/1:213,0,255:217 0/1:255,0,255:166 0/1:255,0,112:35 0/1:53,0,150:48 0/1:128,0,15:25 0/1:245,0,255:237

2 2641687 . G A 999 . . GT:PL:DP 0/1:68,0,255:27 0/1:85,0,255:24 0/1:232,0,255:45 0/1:114,0,58:7 0/1:32,0,131:11 0/1:65,7,0:7 0/1:255,0,231:82

2 2689995 . T A 999 . . GT:PL:DP 0/1:43,0,54:8 0/1:101,4,0:12 0/1:111,0,10:13 0/1:24,3,0:1 0/1:32,6,0:2 0/1:20,0,28:4 0/1:149,0,58:23

2 2754896 . T C 999 . . GT:PL:DP 0/1:203,0,255:155 0/1:122,0,218:131 0/1:170,0,255:157 0/1:44,0,242:24 0/1:51,0,174:42 0/1:70,0,193:48 0/1:135,0,255:210

2 2963531 . G A 999 . . GT:PL:DP 0/1:89,0,31:12 0/1:70,0,9:11 0/1:56,0,28:7 0/1:36,6,0:2 0/1:12,3,0:1 0/1:23,0,6:3 0/1:149,2,0:18

2 2963532 . A T 999 . . GT:PL:DP 0/1:89,0,41:12 0/1:70,0,37:12 0/1:56,0,28:7 0/1:36,6,0:2 0/1:12,3,0:1 0/1:23,0,10:3 0/1:148,0,7:17

2 2963533 . A G 999 . . GT:PL:DP 0/1:89,0,47:12 0/1:70,0,51:12 0/1:70,13,6:6 0/1:36,6,0:2 0/1:12,3,0:1 0/1:23,0,10:3 0/1:150,2,0:16

2 3044061 . G C 999 . . GT:PL:DP 0/1:191,0,255:45 0/1:243,0,255:73 0/1:216,0,255:77 0/1:77,0,66:7 0/1:169,0,90:17 0/1:47,0,113:15 0/1:193,0,255:91

2 3044089 . G A 999 . . GT:PL:DP 0/1:222,0,255:48 0/1:224,0,255:53 0/1:232,0,255:81 0/1:37,0,104:6 0/1:155,0,85:16 0/1:34,0,124:14 0/1:216,0,255:76

2 3048025 . T A 999 . . GT:PL:DP 0/1:58,6,0:2 0/1:83,0,68:13 0/1:151,0,24:14 0/1:75,0,29:7 0/1:36,0,16:5 0/1:25,0,53:9 0/1:18,0,34:5

2 3049278 . T A 999 . . GT:PL:DP 0/1:141,0,255:43 0/1:221,0,122:48 0/1:207,0,51:33 0/1:11,3,0:1 0/1:45,0,32:6 0/1:88,0,52:9 0/1:230,0,174:50

2 3077981 . T G 999 . . GT:PL:DP 0/1:19,0,144:19 0/1:35,0,179:17 0/1:76,0,147:20 0/1:51,0,63:5 0/1:30,3,0:1 0/1:107,0,74:16 0/1:128,0,255:38

2 3104084 . C A 999 . . GT:PL:DP 0/1:154,0,255:59 0/1:183,0,255:48 0/1:255,0,121:60 0/1:178,0,36:14 0/1:118,0,126:15 0/1:111,0,68:8 0/1:52,0,255:102

2 3111859 . A G 999 . . GT:PL:DP 0/1:255,0,197:73 0/1:166,0,231:72 0/1:255,0,93:42 0/1:28,0,96:9 0/1:77,0,66:8 0/1:73,0,32:7 0/1:66,0,255:47

2 3111899 . A C 999 . . GT:PL:DP 0/1:255,0,211:68 0/1:147,0,255:79 0/1:255,0,100:43 0/1:27,0,134:8 0/1:125,0,141:12 0/1:103,0,67:8 0/1:87,0,255:74

2 3216447 . G A 999 . . GT:PL:DP 0/1:89,0,87:17 0/1:108,0,9:19 0/1:176,0,8:22 0/1:60,0,32:6 0/1:28,0,73:7 0/1:44,0,17:8 0/1:130,0,60:31

2 3217519 . A G 999 . . GT:PL:DP 0/1:70,0,170:29 0/1:134,0,104:25 0/1:117,0,96:23 0/1:21,0,28:2 0/1:34,3,0:2 0/1:21,0,46:6 0/1:52,0,196:42

2 3244462 . G C 999 . . GT:PL:DP 0/1:255,0,212:242 0/1:255,0,255:208 0/1:255,0,255:230 0/1:178,0,255:49 0/1:103,0,192:42 0/1:153,0,141:35 0/1:255,0,255:239

2 3244508 . G T 999 . . GT:PL:DP 0/1:255,0,255:168 0/1:255,0,255:172 0/1:229,0,255:183 0/1:180,0,255:35 0/1:118,0,165:18 0/1:112,0,255:24 0/1:255,0,255:180

2 3244575 . C T 999 . . GT:PL:DP 0/1:255,0,255:172 0/1:98,0,255:190 0/1:34,0,255:201 0/1:51,0,255:41 0/1:42,0,200:21 0/1:60,0,212:27 0/1:192,0,255:204

2 3244816 . G A 999 . . GT:PL:DP 0/1:54,0,255:34 0/1:99,0,106:11 0/1:83,0,230:36 0/1:10,0,92:5 0/1:35,3,0:1 0/1:34,3,0:1 0/1:162,0,255:41

2 3254225 . C A 999 . . GT:PL:DP 0/1:68,0,27:11 0/1:86,0,44:6 0/1:43,0,87:7 0/1:64,0,45:5 0/1:65,2,0:6 0/1:24,0,0:6 0/1:98,0,160:20

2 3258485 . G A 999 . . GT:PL:DP 0/1:120,0,255:177 0/1:211,0,255:206 0/1:191,0,255:187 0/1:101,0,255:49 0/1:45,0,217:29 0/1:88,0,137:24 0/1:232,0,255:209

2 3396809 . G A 999 . . GT:PL:DP 0/1:153,0,255:166 0/1:143,0,255:139 0/1:135,0,226:140 0/1:118,0,254:43 0/1:119,0,255:76 0/1:16,0,141:158 0/1:135,0,255:177

2 3411745 . A G 97.4227 . . GT:PL:DP 0/1:27,6,0:2 0/1:21,3,0:1 0/1:37,6,0:2 0/1:22,3,0:1 0/1:14,3,0:1 0/1:33,9,0:3 0/1:20,3,0:1

2 3520015 . A G 999 . . GT:PL:DP 0/1:231,0,26:24 0/1:218,0,90:37 0/1:209,0,33:29 0/1:36,3,0:1 0/1:59,0,22:5 0/1:20,8,0:5 0/1:252,0,23:34

2 3520819 . T A 999 . . GT:PL:DP 0/1:29,0,196:294 0/1:40,0,210:302 0/1:94,0,241:293 0/1:46,0,234:72 0/1:45,0,69:85 0/1:29,0,166:83 0/1:24,0,173:285

2 3521206 . A G 999 . . GT:PL:DP 0/1:77,0,240:112 0/1:98,0,255:138 0/1:91,0,255:121 0/1:12,0,179:67 0/1:13,0,32:19 0/1:50,0,18:42 0/1:95,0,250:184

2 3763037 . G A 999 . . GT:PL:DP 0/1:188,0,255:182 0/1:255,0,255:97 0/1:54,0,255:195 0/1:103,0,255:34 0/1:209,0,229:25 0/1:143,0,173:14 0/1:42,0,255:223

2 3857456 . T C 999 . . GT:PL:DP 0/1:171,0,255:235 0/1:105,0,255:237 0/1:151,0,255:229 0/1:101,0,255:90 0/1:247,0,255:97 0/1:255,0,255:113 0/1:216,0,255:237

2 3970230 . A G 999 . . GT:PL:DP 0/1:255,0,144:65 0/1:255,0,110:60 0/1:255,0,75:65 0/1:130,0,8:8 0/1:206,0,51:14 0/1:255,0,10:20 0/1:255,0,105:84

2 3971160 . A C 999 . . GT:PL:DP 0/1:94,0,140:24 0/1:181,0,135:26 0/1:186,0,7:16 0/1:22,3,0:1 0/1:15,0,67:6 0/1:22,0,76:7 0/1:202,0,164:75

2 3971187 . A T 999 . . GT:PL:DP 0/1:160,0,141:26 0/1:255,0,73:28 0/1:200,0,19:18 0/1:43,6,0:2 0/1:53,6,0:2 0/1:73,0,35:7 0/1:255,0,125:75

2 4075057 . G C 999 . . GT:PL:DP 0/1:144,0,188:29 0/1:143,0,194:29 0/1:15,0,255:31 0/1:19,0,186:11 0/1:104,0,255:35 0/1:167,0,255:97 0/1:102,0,255:67

2 4208088 . A G 999 . . GT:PL:DP 0/1:73,0,81:19 0/1:113,0,107:21 0/1:116,0,91:16 0/1:63,0,6:5 0/1:33,0,78:9 0/1:45,0,55:6 0/1:76,0,181:38

2 4226709 . T A 999 . . GT:PL:DP 0/1:171,0,188:40 0/1:219,0,139:36 0/1:106,0,118:34 0/1:62,0,95:9 0/1:16,0,59:8 0/1:55,0,101:21 0/1:215,0,249:75

2 4249905 . C T 999 . . GT:PL:DP 0/1:138,0,67:87 0/1:226,0,7:96 0/1:111,0,81:104 0/1:79,0,20:38 0/1:24,2,0:48 0/1:161,0,31:87 0/1:184,0,66:96

2 4329179 . A C 999 . . GT:PL:DP 0/1:45,0,183:18 0/1:166,0,125:26 0/1:34,0,157:12 0/1:59,0,15:5 0/1:7,0,101:9 0/1:38,0,199:43 0/1:71,0,255:57

2 4335426 . T A 999 . . GT:PL:DP 0/1:160,0,56:49 0/1:172,0,190:39 0/1:174,0,35:40 0/1:71,0,38:7 0/1:62,0,64:8 0/1:19,0,83:7 0/1:189,0,117:46

2 4335533 . A C 999 . . GT:PL:DP 0/1:163,0,73:21 0/1:201,0,80:23 0/1:103,0,104:14 0/1:61,6,0:2 0/1:24,0,70:5 0/1:32,0,65:5 0/1:218,0,147:40

2 4343347 . G A 999 . . GT:PL:DP 0/1:255,0,24:32 0/1:173,0,49:28 0/1:255,0,97:34 0/1:74,21,17:6 0/1:31,0,32:8 0/1:28,0,44:8 0/1:255,0,142:62

2 4345241 . T G 999 . . GT:PL:DP 0/1:80,0,239:47 0/1:202,0,211:38 0/1:193,0,113:25 0/1:99,0,35:8 0/1:59,0,52:6 0/1:49,0,18:3 0/1:213,0,115:74

2 4367252 . G A 999 . . GT:PL:DP 0/1:232,0,77:29 0/1:120,0,32:15 0/1:113,0,69:17 0/1:40,0,82:6 0/1:14,0,47:5 0/1:73,0,39:5 0/1:213,0,130:30

2 4377976 . A T 999 . . GT:PL:DP 0/1:255,0,255:101 0/1:235,0,255:99 0/1:255,0,255:96 0/1:255,0,255:60 0/1:255,0,255:160 0/1:255,0,255:211 0/1:228,0,255:125

2 4405112 . T A 999 . . GT:PL:DP 0/1:128,0,184:38 0/1:56,0,24:6 0/1:198,0,103:38 0/1:6,0,75:10 0/1:154,0,61:12 0/1:177,0,153:17 0/1:241,0,97:36

2 4616702 . G A 999 . . GT:PL:DP 0/1:144,0,190:79 0/1:151,0,179:61 0/1:153,0,170:67 0/1:94,0,115:14 0/1:70,0,83:8 0/1:104,0,67:12 0/1:122,0,254:92

2 4755292 . C G 999 . . GT:PL:DP 0/1:235,0,236:126 0/1:184,0,226:95 0/1:255,0,222:120 0/1:61,0,106:18 0/1:9,0,11:13 0/1:70,0,87:29 0/1:197,0,255:116

2 4761531 . C T 999 . . GT:PL:DP 0/1:115,0,47:22 0/1:124,0,54:22 0/1:126,8,0:33 0/1:10,0,87:9 0/1:13,0,35:5 0/1:42,0,56:12 0/1:71,0,135:52

2 4796785 . C T 999 . . GT:PL:DP 0/1:103,0,248:18 0/1:128,0,255:34 0/1:187,0,188:21 0/1:25,0,66:4 0/1:12,0,115:9 0/1:21,0,98:7 0/1:255,0,229:34

2 4828682 . T G 999 . . GT:PL:DP 0/1:255,0,255:80 0/1:201,0,255:118 0/1:255,0,255:135 0/1:49,0,128:11 0/1:93,0,255:21 0/1:98,0,171:11 0/1:12,0,235:217

2 4828732 . G A 999 . . GT:PL:DP 0/1:255,0,255:89 0/1:255,0,255:121 0/1:255,6,255:138 0/1:82,53,221:14 0/1:62,0,247:26 0/1:142,0,186:17 0/1:78,0,255:201

2 4828780 . G C 999 . . GT:PL:DP 0/1:255,0,255:86 0/1:255,0,255:118 0/1:187,0,255:140 0/1:99,0,242:16 0/1:49,0,255:20 0/1:187,0,186:18 0/1:177,0,255:202

2 4829002 . C A 999 . . GT:PL:DP 0/1:255,0,229:53 0/1:188,0,255:80 0/1:255,0,255:114 0/1:62,0,128:8 0/1:28,0,202:13 0/1:195,0,88:15 0/1:229,0,255:186

2 5049317 . T C 999 . . GT:PL:DP 0/1:141,0,130:76 0/1:127,0,253:77 0/1:143,0,239:70 0/1:46,0,111:7 0/1:21,0,19:6 0/1:10,0,27:4 0/1:186,0,253:85

2 5049713 . C G 999 . . GT:PL:DP 0/1:121,0,91:40 0/1:180,0,224:58 0/1:150,0,108:43 0/1:60,0,20:10 0/1:17,8,0:5 0/1:23,0,40:11 0/1:87,0,255:68

2 5050112 . A C 999 . . GT:PL:DP 0/1:125,0,99:26 0/1:83,0,162:47 0/1:116,0,151:51 0/1:48,0,50:5 0/1:17,0,0:8 0/1:54,0,37:23 0/1:129,0,196:60

2 5050123 . C T 999 . . GT:PL:DP 0/1:129,0,129:33 0/1:67,0,166:52 0/1:123,0,148:48 0/1:46,0,50:6 0/1:17,0,1:8 0/1:38,0,34:22 0/1:126,0,197:62

2 5076259 . G C 999 . . GT:PL:DP 0/1:52,0,113:26 0/1:48,0,174:25 0/1:48,0,171:28 0/1:46,0,59:8 0/1:11,0,25:10 0/1:38,12,0:4 0/1:38,0,138:24

2 5124164 . A G 999 . . GT:PL:DP 0/1:243,0,79:27 0/1:156,0,13:23 0/1:219,0,29:22 0/1:115,0,4:7 0/1:40,0,32:7 0/1:33,0,120:19 0/1:156,0,212:44

2 5223065 . C G 999 . . GT:PL:DP 0/1:157,0,63:31 0/1:122,0,23:47 0/1:193,0,105:41 0/1:54,0,37:4 0/1:142,0,19:15 0/1:36,0,44:6 0/1:163,0,43:49

2 5241347 . C T 999 . . GT:PL:DP 0/1:56,0,137:28 0/1:65,0,115:31 0/1:76,0,83:15 0/1:24,0,8:2 0/1:54,0,37:8 0/1:36,0,62:7 0/1:145,0,188:48

2 5488345 . T C 999 . . GT:PL:DP 0/1:58,0,96:34 0/1:28,0,93:25 0/1:94,0,66:29 0/1:18,0,32:6 0/1:34,0,39:8 0/1:17,0,85:12 0/1:99,0,113:51

2 5567297 . A T 999 . . GT:PL:DP 0/1:255,0,255:212 0/1:255,0,255:207 0/1:219,0,255:211 0/1:90,0,255:54 0/1:13,0,226:50 0/1:38,0,255:77 0/1:255,0,255:229

2 5572115 . C T 999 . . GT:PL:DP 0/1:44,0,149:20 0/1:113,0,125:43 0/1:76,0,78:28 0/1:27,0,30:6 0/1:28,0,126:12 0/1:69,0,82:23 0/1:142,0,214:73

2 5629138 . T C 999 . . GT:PL:DP 0/1:255,0,231:61 0/1:97,0,255:50 0/1:255,0,161:60 0/1:119,0,172:15 0/1:121,0,46:10 0/1:79,0,36:6 0/1:255,0,255:69

2 5756707 . A C 999 . . GT:PL:DP 0/1:25,0,202:30 0/1:41,0,180:39 0/1:29,0,124:24 0/1:18,0,26:8 0/1:40,0,39:5 0/1:33,0,172:21 0/1:146,0,241:51

2 5756711 . G A 999 . . GT:PL:DP 0/1:191,0,51:31 0/1:148,0,82:40 0/1:128,0,33:24 0/1:61,0,8:10 0/1:39,0,40:5 0/1:120,0,78:20 0/1:188,0,177:48

2 5756724 . G A 999 . . GT:PL:DP 0/1:6,0,213:32 0/1:126,0,89:40 0/1:66,0,143:26 0/1:16,0,26:10 0/1:56,0,16:5 0/1:73,0,3:14 0/1:53,0,187:55

2 5982109 . C T 999 . . GT:PL:DP 0/1:82,0,37:12 0/1:80,7,0:5 0/1:90,0,94:18 0/1:27,3,0:1 0/1:46,6,0:2 0/1:60,9,0:3 0/1:18,0,132:19

2 5986925 . T C 999 . . GT:PL:DP 0/1:102,0,2:12 0/1:93,0,4:9 0/1:106,0,25:13 0/1:11,3,0:1 0/1:16,0,1:2 0/1:16,3,0:1 0/1:149,0,3:16

2 5986932 . T A 999 . . GT:PL:DP 0/1:108,0,28:14 0/1:93,0,15:9 0/1:106,0,79:15 0/1:29,3,0:1 0/1:39,1,0:3 0/1:10,0,2:2 0/1:149,0,8:15

2 5986933 . T C 999 . . GT:PL:DP 0/1:112,0,28:14 0/1:93,0,16:9 0/1:90,0,88:14 0/1:29,3,0:1 0/1:39,1,0:3 0/1:10,0,2:2 0/1:149,0,9:15

2 6005977 . T A 999 . . GT:PL:DP 0/1:63,0,71:18 0/1:44,0,80:18 0/1:45,0,95:24 0/1:20,0,15:4 0/1:40,3,0:3 0/1:83,0,32:11 0/1:58,0,105:31

2 6009661 . T A 999 . . GT:PL:DP 0/1:137,0,138:26 0/1:97,0,91:25 0/1:133,0,71:26 0/1:12,0,29:5 0/1:34,6,0:2 0/1:22,0,10:3 0/1:108,0,95:46

2 6055027 . G A 999 . . GT:PL:DP 0/1:170,0,84:20 0/1:110,0,235:36 0/1:64,0,171:31 0/1:13,0,31:2 0/1:11,0,67:4 0/1:10,0,33:2 0/1:52,0,255:37

2 6055035 . A G 999 . . GT:PL:DP 0/1:193,0,60:21 0/1:135,0,189:29 0/1:64,0,170:31 0/1:13,0,31:2 0/1:37,0,62:5 0/1:10,0,33:2 0/1:112,0,255:38

2 6082975 . T A 999 . . GT:PL:DP 0/1:76,0,195:42 0/1:45,0,184:37 0/1:87,0,119:48 0/1:37,0,80:12 0/1:17,6,0:2 0/1:16,6,0:2 0/1:22,0,240:152

2 6083693 . C T 999 . . GT:PL:DP 0/1:255,0,255:39 0/1:126,0,183:15 0/1:153,0,255:24 0/1:104,0,104:12 0/1:38,10,7:2 0/1:34,3,0:1 0/1:225,0,255:101

2 6085158 . T C 999 . . GT:PL:DP 0/1:255,0,215:77 0/1:146,0,255:40 0/1:143,0,255:65 0/1:63,0,206:14 0/1:61,0,174:13 0/1:56,0,132:10 0/1:7,0,255:183

2 6246629 . A T 999 . . GT:PL:DP 0/1:133,0,246:57 0/1:146,0,255:51 0/1:153,0,195:39 0/1:13,0,178:9 0/1:23,0,76:5 0/1:52,0,156:18 0/1:81,0,255:103

2 6246653 . G A 999 . . GT:PL:DP 0/1:191,0,185:60 0/1:119,0,105:71 0/1:132,0,127:59 0/1:103,0,86:14 0/1:30,0,28:3 0/1:88,0,74:15 0/1:255,0,168:120

2 6268623 . C T 999 . . GT:PL:DP 0/1:255,0,255:219 0/1:255,0,255:221 0/1:255,0,255:227 0/1:255,0,255:98 0/1:255,0,255:162 0/1:255,0,255:209 0/1:255,0,255:229

3 378865 . A G 999 . . GT:PL:DP 0/1:96,0,175:121 0/1:130,0,205:131 0/1:110,0,154:132 0/1:76,0,43:19 0/1:101,0,33:31 0/1:20,0,10:37 0/1:135,0,197:133

3 379368 . G A 999 . . GT:PL:DP 0/1:128,0,156:90 0/1:103,0,214:96 0/1:178,0,214:114 0/1:7,0,51:8 0/1:65,0,112:17 0/1:95,0,148:22 0/1:173,0,241:69

3 659890 . A C 999 . . GT:PL:DP 0/1:12,0,94:8 0/1:45,0,3:9 0/1:119,0,1:9 0/1:41,9,0:3 0/1:61,9,0:3 0/1:43,6,0:2 0/1:94,0,47:13

3 662698 . T A 999 . . GT:PL:DP 0/1:122,0,228:27 0/1:183,0,248:40 0/1:164,0,240:35 0/1:70,0,48:5 0/1:79,0,38:5 0/1:192,0,95:21 0/1:228,0,255:59

3 753882 . C A 999 . . GT:PL:DP 0/1:137,0,159:25 0/1:182,0,114:26 0/1:175,0,222:45 0/1:86,0,131:11 0/1:77,0,83:7 0/1:73,0,164:11 0/1:115,0,226:35

3 753974 . G A 999 . . GT:PL:DP 0/1:108,0,247:26 0/1:141,0,255:27 0/1:255,0,254:42 0/1:88,0,192:13 0/1:57,0,144:10 0/1:129,0,135:11 0/1:255,0,115:46

3 754002 . T A 999 . . GT:PL:DP 0/1:86,0,255:24 0/1:13,0,255:18 0/1:255,0,251:39 0/1:37,0,214:12 0/1:41,0,190:10 0/1:101,0,122:9 0/1:167,0,255:42

3 754167 . C A 999 . . GT:PL:DP 0/1:49,0,255:40 0/1:134,0,144:19 0/1:93,0,161:23 0/1:71,0,43:5 0/1:34,0,33:2 0/1:55,0,96:7 0/1:87,0,255:41

3 754168 . G A 999 . . GT:PL:DP 0/1:206,0,255:39 0/1:132,0,127:19 0/1:148,0,93:23 0/1:22,0,92:5 0/1:33,0,34:2 0/1:88,0,59:7 0/1:146,0,255:42

3 787168 . T C 999 . . GT:PL:DP 0/1:201,0,255:168 0/1:251,0,255:158 0/1:211,0,255:194 0/1:128,0,231:28 0/1:89,0,141:15 0/1:140,0,146:25 0/1:152,0,255:205

3 841550 . T A 999 . . GT:PL:DP 0/1:94,0,255:31 0/1:255,0,255:53 0/1:255,0,219:42 0/1:23,0,208:13 0/1:111,0,144:11 0/1:58,0,54:4 0/1:255,0,255:124

3 841620 . C A 999 . . GT:PL:DP 0/1:229,0,150:26 0/1:126,0,200:47 0/1:98,0,208:36 0/1:173,0,73:14 0/1:111,0,0:5 0/1:82,9,0:3 0/1:180,0,255:96

3 941132 . C G 999 . . GT:PL:DP 0/1:140,0,30:10 0/1:140,0,238:36 0/1:230,0,82:26 0/1:58,0,3:3 0/1:34,3,0:1 0/1:71,9,0:3 0/1:255,0,168:39

3 1033258 . G A 999 . . GT:PL:DP 0/1:105,0,149:23 0/1:92,0,132:14 0/1:255,4,0:32 0/1:37,0,59:6 0/1:39,0,124:6 0/1:122,0,82:9 0/1:221,0,86:37

3 1382363 . C T 999 . . GT:PL:DP 0/1:36,0,108:13 0/1:75,5,0:13 0/1:59,0,57:16 0/1:7,0,95:5 0/1:17,0,98:7 0/1:21,3,0:1 0/1:61,0,231:44

3 1817215 . G A 999 . . GT:PL:DP 0/1:190,0,40:16 0/1:131,0,92:23 0/1:97,0,124:16 0/1:86,9,0:3 0/1:15,0,26:3 0/1:35,3,0:1 0/1:163,0,73:18

3 2085404 . C T 999 . . GT:PL:DP 0/1:199,0,74:32 0/1:255,0,147:41 0/1:151,0,108:23 0/1:52,0,37:6 0/1:79,0,15:9 0/1:137,0,27:20 0/1:222,0,225:76

3 2086391 . C T 999 . . GT:PL:DP 0/1:162,0,152:58 0/1:147,0,155:35 0/1:85,0,156:40 0/1:64,0,29:6 0/1:36,0,28:2 0/1:73,0,52:17 0/1:172,0,115:33

3 2086613 . C T 999 . . GT:PL:DP 0/1:255,0,255:75 0/1:255,0,255:55 0/1:234,0,255:72 0/1:146,0,86:12 0/1:108,0,182:20 0/1:152,0,255:60 0/1:255,0,255:92

3 2086766 . A G 999 . . GT:PL:DP 0/1:255,0,255:201 0/1:255,0,255:201 0/1:255,0,255:202 0/1:198,0,255:35 0/1:155,0,255:39 0/1:128,0,255:97 0/1:255,0,255:160

3 2086772 . A G 999 . . GT:PL:DP 0/1:255,0,255:200 0/1:255,0,255:205 0/1:255,0,255:202 0/1:213,0,255:38 0/1:155,0,255:40 0/1:132,0,255:107 0/1:255,0,255:161

3 2086877 . A G 999 . . GT:PL:DP 0/1:255,0,255:198 0/1:255,0,255:191 0/1:209,0,255:198 0/1:250,0,255:38 0/1:84,0,244:52 0/1:222,0,255:147 0/1:255,0,255:164

3 2086889 . A G 999 . . GT:PL:DP 0/1:255,0,255:198 0/1:255,0,255:180 0/1:172,0,255:199 0/1:255,0,255:36 0/1:63,0,178:46 0/1:126,0,255:139 0/1:255,0,255:160

3 2087074 . G A 999 . . GT:PL:DP 0/1:255,0,255:89 0/1:192,0,255:67 0/1:255,0,255:71 0/1:16,0,88:7 0/1:22,0,108:13 0/1:22,0,76:31 0/1:188,0,255:81

3 2087090 . G C 999 . . GT:PL:DP 0/1:255,0,255:129 0/1:236,0,255:98 0/1:255,0,255:106 0/1:47,0,142:9 0/1:61,0,137:25 0/1:53,0,70:63 0/1:247,0,255:113

3 2087124 . C T 999 . . GT:PL:DP 0/1:90,0,255:195 0/1:90,0,255:157 0/1:147,0,255:181 0/1:113,0,225:21 0/1:64,0,251:46 0/1:103,0,255:106 0/1:255,0,255:165

3 2087504 . A C 999 . . GT:PL:DP 0/1:58,0,188:57 0/1:10,0,142:32 0/1:15,0,152:30 0/1:24,0,62:4 0/1:41,0,90:9 0/1:26,0,18:12 0/1:66,0,143:21

3 2119567 . T A 999 . . GT:PL:DP 0/1:134,0,90:27 0/1:140,0,50:19 0/1:93,0,29:13 0/1:69,0,26:9 0/1:15,0,33:3 0/1:13,0,26:3 0/1:14,0,137:21

3 2242479 . T C 999 . . GT:PL:DP 0/1:178,0,9:22 0/1:150,0,88:21 0/1:178,0,81:33 0/1:38,0,30:4 0/1:43,0,32:5 0/1:52,0,40:5 0/1:140,0,25:24

3 2252835 . T A 999 . . GT:PL:DP 0/1:143,0,165:39 0/1:160,0,196:51 0/1:196,0,92:33 0/1:76,0,83:11 0/1:51,5,0:4 0/1:30,6,0:2 0/1:149,0,199:38

3 2252854 . T A 999 . . GT:PL:DP 0/1:148,0,155:40 0/1:116,0,161:40 0/1:187,0,58:26 0/1:40,0,94:9 0/1:14,1,0:4 0/1:30,6,0:2 0/1:121,0,147:27

3 2253550 . C A 999 . . GT:PL:DP 0/1:83,0,48:21 0/1:110,0,24:34 0/1:91,0,75:23 0/1:26,0,5:3 0/1:44,0,46:7 0/1:26,0,27:5 0/1:135,0,66:39

3 2335549 . T G 999 . . GT:PL:DP 0/1:73,0,125:17 0/1:64,0,155:19 0/1:5,0,153:14 0/1:33,6,0:2 0/1:75,0,66:9 0/1:23,6,0:2 0/1:98,0,108:25

3 2363128 . T G 999 . . GT:PL:DP 0/1:90,0,89:21 0/1:103,0,99:21 0/1:126,0,54:33 0/1:39,0,13:4 0/1:17,0,33:6 0/1:22,0,20:7 0/1:107,0,112:37

3 2456581 . T C 999 . . GT:PL:DP 0/1:156,0,255:234 0/1:56,0,255:237 0/1:34,0,249:215 0/1:156,0,255:89 0/1:198,0,255:75 0/1:180,0,255:163 0/1:120,0,255:211

3 2600300 . C T 999 . . GT:PL:DP 0/1:168,0,255:231 0/1:173,0,255:228 0/1:185,0,255:240 0/1:170,0,255:251 0/1:107,0,255:224 0/1:75,0,223:237 0/1:148,0,255:235

3 2600320 . A G 999 . . GT:PL:DP 0/1:255,0,255:224 0/1:255,0,255:233 0/1:255,0,255:242 0/1:255,0,255:246 0/1:255,0,255:229 0/1:255,0,243:242 0/1:255,0,255:233

3 2702988 . T C 999 . . GT:PL:DP 0/1:89,0,125:27 0/1:97,0,228:29 0/1:79,0,102:19 0/1:12,0,19:2 0/1:53,0,31:6 0/1:10,0,33:2 0/1:63,0,184:31

3 2704823 . T A 999 . . GT:PL:DP 0/1:235,0,169:132 0/1:255,0,152:156 0/1:237,0,181:129 0/1:110,0,24:15 0/1:88,0,2:8 0/1:97,0,39:8 0/1:255,0,74:223

3 2726414 . G A 999 . . GT:PL:DP 0/1:53,0,96:12 0/1:59,0,58:11 0/1:121,0,74:15 0/1:25,3,0:1 0/1:19,0,0:2 0/1:16,0,3:3 0/1:85,0,68:11

3 2726418 . T G 999 . . GT:PL:DP 0/1:63,0,68:11 0/1:108,0,36:12 0/1:96,0,80:16 0/1:25,3,0:1 0/1:44,3,0:3 0/1:16,0,3:3 0/1:150,0,43:14

3 2726421 . T C 999 . . GT:PL:DP 0/1:79,0,68:11 0/1:115,0,36:12 0/1:107,0,80:16 0/1:25,3,0:1 0/1:44,3,0:3 0/1:16,0,3:3 0/1:129,0,61:14

3 2726422 . C A 999 . . GT:PL:DP 0/1:88,0,53:10 0/1:115,0,36:12 0/1:131,0,72:16 0/1:25,3,0:1 0/1:44,3,0:3 0/1:16,0,3:3 0/1:168,0,43:14

3 2776789 . G A 999 . . GT:PL:DP 0/1:255,0,255:219 0/1:173,0,255:223 0/1:255,0,255:216 0/1:239,0,255:37 0/1:191,0,255:79 0/1:255,0,255:153 0/1:255,0,255:221

3 2777035 . G A 999 . . GT:PL:DP 0/1:255,0,255:244 0/1:255,0,172:240 0/1:255,0,255:239 0/1:255,0,255:80 0/1:255,0,255:83 0/1:255,0,255:177 0/1:255,0,255:241

3 2820377 . C A 999 . . GT:PL:DP 0/1:112,0,255:236 0/1:34,0,235:241 0/1:105,0,249:225 0/1:181,0,255:162 0/1:239,0,255:179 0/1:255,0,255:182 0/1:255,0,255:239

3 2820495 . G A 999 . . GT:PL:DP 0/1:105,0,255:243 0/1:210,0,255:240 0/1:255,0,255:241 0/1:232,0,255:185 0/1:191,0,255:178 0/1:255,0,255:204 0/1:255,0,255:248

3 2820529 . A T 999 . . GT:PL:DP 0/1:161,0,255:241 0/1:202,0,255:239 0/1:255,0,255:228 0/1:249,0,255:195 0/1:215,0,255:193 0/1:255,0,255:185 0/1:255,0,255:232

3 2820610 . G A 999 . . GT:PL:DP 0/1:233,0,255:239 0/1:140,0,255:233 0/1:255,0,255:228 0/1:211,0,255:151 0/1:192,0,255:197 0/1:255,0,255:189 0/1:249,0,255:237

3 3055310 . G A 999 . . GT:PL:DP 0/1:177,0,240:201 0/1:122,0,197:189 0/1:125,0,255:153 0/1:116,0,84:35 0/1:22,0,25:72 0/1:28,0,0:95 0/1:162,0,157:241

3 3074379 . A C 999 . . GT:PL:DP 0/1:197,0,244:29 0/1:213,0,209:23 0/1:153,0,255:23 0/1:31,0,31:2 0/1:52,0,127:6 0/1:37,3,0:1 0/1:255,0,253:40

3 3074460 . C T 999 . . GT:PL:DP 0/1:198,0,223:32 0/1:101,0,208:20 0/1:125,0,160:17 0/1:53,0,28:3 0/1:67,0,14:5 0/1:38,6,0:2 0/1:202,0,255:46

3 3075667 . C A 999 . . GT:PL:DP 0/1:167,0,255:93 0/1:83,0,255:78 0/1:84,0,255:105 0/1:49,0,162:13 0/1:46,0,138:13 0/1:145,0,150:61 0/1:249,0,255:165

3 3141992 . G A 999 . . GT:PL:DP 0/1:133,0,209:27 0/1:170,0,201:34 0/1:162,0,203:27 0/1:55,0,42:6 0/1:34,0,67:6 0/1:53,6,0:2 0/1:99,0,255:37

3 3142030 . G A 999 . . GT:PL:DP 0/1:143,0,164:44 0/1:202,0,160:42 0/1:136,0,160:31 0/1:61,0,54:9 0/1:10,0,65:5 0/1:27,3,0:1 0/1:70,0,197:32

3 3142033 . A G 999 . . GT:PL:DP 0/1:153,0,161:45 0/1:197,0,163:42 0/1:139,0,165:31 0/1:65,0,51:10 0/1:7,0,66:6 0/1:40,6,0:2 0/1:64,0,200:34

3 3183524 . C A 999 . . GT:PL:DP 0/1:255,0,247:35 0/1:255,0,139:31 0/1:238,0,124:21 0/1:85,9,0:3 0/1:29,3,0:1 0/1:66,6,0:2 0/1:255,0,218:39

3 3189535 . T G 999 . . GT:PL:DP 0/1:136,0,208:68 0/1:107,0,201:69 0/1:111,0,174:121 0/1:117,0,128:24 0/1:73,0,205:37 0/1:107,0,114:20 0/1:255,0,178:205

3 3190403 . C T 999 . . GT:PL:DP 0/1:239,0,244:88 0/1:183,0,255:73 0/1:161,0,208:49 0/1:87,0,61:9 0/1:22,0,82:9 0/1:39,0,20:16 0/1:127,0,255:124

3 3204572 . C G 999 . . GT:PL:DP 0/1:144,0,161:115 0/1:113,0,192:126 0/1:144,0,206:127 0/1:85,0,144:26 0/1:78,0,129:28 0/1:51,0,21:17 0/1:19,0,222:192

3 3475547 . T C 999 . . GT:PL:DP 0/1:205,0,148:43 0/1:35,0,181:27 0/1:96,0,180:21 0/1:13,0,38:3 0/1:21,0,48:4 0/1:17,3,0:1 0/1:121,0,245:39

3 3500598 . T G 999 . . GT:PL:DP 0/1:83,0,84:23 0/1:139,0,31:17 0/1:152,0,58:19 0/1:81,0,4:6 0/1:17,0,7:3 0/1:15,0,4:3 0/1:175,0,108:44

3 3500705 . C A 999 . . GT:PL:DP 0/1:176,0,152:24 0/1:218,0,51:21 0/1:215,0,115:21 0/1:53,0,13:3 0/1:78,0,24:6 0/1:25,0,28:2 0/1:232,0,161:45

3 3500707 . G C 999 . . GT:PL:DP 0/1:198,0,121:26 0/1:211,0,53:22 0/1:214,0,108:21 0/1:53,0,13:3 0/1:78,0,24:6 0/1:31,3,0:1 0/1:237,0,109:45

3 3639336 . G A 999 . . GT:PL:DP 0/1:99,0,70:17 0/1:103,0,58:12 0/1:51,0,47:9 0/1:68,0,2:5 0/1:12,3,0:1 0/1:39,6,0:2 0/1:155,0,101:27

3 3704007 . T G 999 . . GT:PL:DP 0/1:221,0,191:43 0/1:206,0,208:33 0/1:156,0,218:29 0/1:57,0,128:11 0/1:59,0,72:8 0/1:67,9,0:3 0/1:247,0,189:46

3 3728901 . G A 999 . . GT:PL:DP 0/1:255,0,238:152 0/1:255,0,239:207 0/1:255,0,192:99 0/1:255,0,251:33 0/1:255,0,95:31 0/1:183,0,226:22 0/1:255,0,198:227

3 3729007 . A G 999 . . GT:PL:DP 0/1:255,0,255:178 0/1:255,0,255:219 0/1:255,0,255:113 0/1:255,0,255:34 0/1:255,0,124:37 0/1:255,0,201:30 0/1:255,0,232:221

3 3732000 . G A 999 . . GT:PL:DP 0/1:147,0,16:16 0/1:132,0,43:19 0/1:158,0,8:23 0/1:35,0,17:4 0/1:16,0,0:3 0/1:34,3,0:1 0/1:192,0,21:44

3 3803658 . G A 999 . . GT:PL:DP 0/1:136,0,140:21 0/1:107,0,138:16 0/1:28,0,115:9 0/1:38,0,110:8 0/1:46,0,15:3 0/1:32,3,0:1 0/1:144,0,137:26

3 3864737 . T C 999 . . GT:PL:DP 0/1:235,0,255:38 0/1:255,0,221:34 0/1:174,0,255:39 0/1:108,0,82:8 0/1:107,0,72:7 0/1:75,9,0:3 0/1:255,0,255:43

3 3943543 . A C 999 . . GT:PL:DP 0/1:223,0,54:21 0/1:79,0,227:29 0/1:207,9,0:16 0/1:57,6,0:2 0/1:27,3,0:1 0/1:24,3,0:1 0/1:244,0,10:30

3 3976184 . A C 999 . . GT:PL:DP 0/1:206,0,41:35 0/1:131,2,0:19 0/1:173,0,77:21 0/1:41,0,65:9 0/1:18,0,53:8 0/1:35,0,57:9 0/1:195,3,0:80

3 4051512 . T C 999 . . GT:PL:DP 0/1:134,0,35:11 0/1:81,0,84:20 0/1:185,0,89:28 0/1:22,0,2:2 0/1:24,3,0:1 0/1:52,6,0:2 0/1:169,0,0:20

3 4309062 . C T 999 . . GT:PL:DP 0/1:115,0,182:38 0/1:38,0,165:25 0/1:177,0,150:44 0/1:17,0,67:7 0/1:23,0,61:8 0/1:15,0,92:5 0/1:117,0,185:71

3 4336599 . T C 999 . . GT:PL:DP 0/1:21,3,0:1 0/1:64,0,131:9 0/1:127,0,19:6 0/1:30,0,19:2 0/1:35,3,0:1 0/1:77,0,11:5 0/1:35,0,208:31

3 4363070 . G A 999 . . GT:PL:DP 0/1:255,0,255:86 0/1:255,0,249:95 0/1:255,0,255:73 0/1:96,0,178:15 0/1:199,0,116:19 0/1:185,0,255:75 0/1:255,0,255:168

3 4363126 . C T 999 . . GT:PL:DP 0/1:161,0,161:74 0/1:231,0,240:71 0/1:200,0,182:68 0/1:39,0,90:10 0/1:64,0,110:16 0/1:80,0,234:49 0/1:218,0,240:152

3 4391562 . G C 999 . . GT:PL:DP 0/1:203,0,174:28 0/1:156,0,82:22 0/1:214,0,143:24 0/1:5,0,7:2 0/1:59,6,0:2 0/1:70,9,0:3 0/1:220,0,207:38

3 4423879 . G A 999 . . GT:PL:DP 0/1:116,0,41:20 0/1:101,0,76:14 0/1:140,0,170:30 0/1:45,0,82:7 0/1:68,0,45:5 0/1:52,6,0:2 0/1:149,0,59:41

3 4506168 . C T 999 . . GT:PL:DP 0/1:33,0,4:13 0/1:24,0,105:22 0/1:85,0,36:13 0/1:12,0,4:3 0/1:15,6,0:2 0/1:14,1,0:2 0/1:51,0,7:16

3 4507483 . A T 999 . . GT:PL:DP 0/1:230,0,3:17 0/1:170,0,51:23 0/1:241,7,0:26 0/1:11,0,12:2 0/1:43,9,0:3 0/1:67,9,0:3 0/1:255,0,42:55

3 4548618 . G C 999 . . GT:PL:DP 0/1:29,0,191:19 0/1:219,0,165:27 0/1:248,0,115:27 0/1:69,0,43:7 0/1:31,0,29:2 0/1:55,6,0:2 0/1:255,0,197:42

3 4552375 . A G 999 . . GT:PL:DP 0/1:255,0,247:176 0/1:255,0,240:148 0/1:255,0,248:171 0/1:255,0,70:27 0/1:57,0,120:21 0/1:89,0,88:18 0/1:255,0,255:188

3 4563551 . T G 999 . . GT:PL:DP 0/1:106,0,45:7 0/1:22,0,59:3 0/1:75,0,68:8 0/1:28,3,0:1 0/1:23,3,0:1 0/1:21,3,0:1 0/1:124,0,13:17

3 4566014 . A G 999 . . GT:PL:DP 0/1:58,0,77:22 0/1:148,0,16:16 0/1:139,0,80:19 0/1:17,3,0:1 0/1:70,0,10:6 0/1:38,6,0:2 0/1:153,3,0:23

3 4612338 . A T 999 . . GT:PL:DP 0/1:86,0,62:9 0/1:19,0,22:6 0/1:125,0,135:20 0/1:6,0,28:2 0/1:71,0,17:4 0/1:16,0,23:2 0/1:28,0,88:10

3 4612340 . A T 999 . . GT:PL:DP 0/1:66,0,51:9 0/1:19,0,22:6 0/1:125,0,135:20 0/1:6,0,19:2 0/1:71,0,17:4 0/1:16,0,23:2 0/1:28,0,88:10

3 4612344 . A C 999 . . GT:PL:DP 0/1:64,0,84:10 0/1:19,0,22:6 0/1:89,0,138:18 0/1:6,0,28:2 0/1:42,0,20:3 0/1:16,0,23:2 0/1:26,0,79:10

3 4626421 . T C 999 . . GT:PL:DP 0/1:255,0,255:36 0/1:194,0,255:34 0/1:255,0,255:35 0/1:38,0,144:7 0/1:81,0,166:11 0/1:70,6,0:2 0/1:255,0,255:59

3 4778680 . G A 999 . . GT:PL:DP 0/1:133,0,17:23 0/1:199,0,76:29 0/1:13,0,55:20 0/1:14,0,53:9 0/1:90,0,43:20 0/1:136,0,35:52 0/1:154,0,170:76

3 4921193 . T C 999 . . GT:PL:DP 0/1:255,0,250:168 0/1:255,0,255:145 0/1:255,0,255:169 0/1:195,0,133:36 0/1:214,0,214:98 0/1:254,0,201:118 0/1:255,0,255:167

3 4974775 . G C 999 . . GT:PL:DP 0/1:192,0,255:98 0/1:183,0,255:124 0/1:183,0,255:103 0/1:170,0,197:45 0/1:26,3,0:8 0/1:9,0,47:23 0/1:255,0,255:142

3 5053642 . G T 999 . . GT:PL:DP 0/1:197,0,154:115 0/1:196,0,72:139 0/1:250,0,207:107 0/1:100,0,47:19 0/1:28,0,47:13 0/1:74,0,13:10 0/1:142,0,91:189

3 5061014 . C A 999 . . GT:PL:DP 0/1:76,0,113:11 0/1:76,5,0:6 0/1:176,7,0:12 0/1:55,3,0:3 0/1:44,6,0:2 0/1:37,0,68:7 0/1:247,0,0:22

3 5076395 . A G 999 . . GT:PL:DP 0/1:81,9,0:3 0/1:33,3,0:1 0/1:53,6,0:2 0/1:11,3,0:1 0/1:61,6,0:2 0/1:50,6,0:2 0/1:46,0,20:3

3 5078157 . A T 999 . . GT:PL:DP 0/1:134,0,255:109 0/1:105,0,255:99 0/1:149,0,255:117 0/1:42,0,183:28 0/1:15,0,120:24 0/1:14,0,132:20 0/1:235,0,255:149

3 5078159 . T C 999 . . GT:PL:DP 0/1:151,0,255:115 0/1:122,0,255:99 0/1:164,0,255:118 0/1:28,0,172:27 0/1:17,0,121:24 0/1:14,0,132:20 0/1:242,0,255:152

3 5078254 . T A 999 . . GT:PL:DP 0/1:140,0,255:158 0/1:107,0,255:135 0/1:168,0,255:150 0/1:68,0,226:34 0/1:204,0,255:106 0/1:241,0,255:77 0/1:238,0,255:195

3 5079360 . G A 999 . . GT:PL:DP 0/1:255,0,162:59 0/1:255,0,175:60 0/1:255,0,231:61 0/1:58,0,29:5 0/1:145,0,64:12 0/1:192,0,86:23 0/1:255,0,180:94

3 5080654 . T A 999 . . GT:PL:DP 0/1:155,0,171:60 0/1:112,0,90:64 0/1:188,0,128:83 0/1:48,0,47:12 0/1:25,11,102:14 0/1:55,0,115:17 0/1:155,0,139:84

3 5110043 . T A 999 . . GT:PL:DP 0/1:249,0,233:209 0/1:168,0,204:149 0/1:143,0,209:205 0/1:203,0,194:49 0/1:124,0,178:56 0/1:43,0,86:15 0/1:95,0,230:235

3 5110535 . G T 999 . . GT:PL:DP 0/1:184,0,255:226 0/1:142,0,255:224 0/1:101,0,232:204 0/1:167,0,255:64 0/1:188,0,255:68 0/1:197,0,255:62 0/1:38,0,242:225

3 5147142 . A G 999 . . GT:PL:DP 0/1:65,0,27:11 0/1:111,0,16:12 0/1:48,18,24:6 0/1:19,3,0:1 0/1:21,0,7:6 0/1:25,0,18:3 0/1:115,0,22:15

3 5147146 . C A 999 . . GT:PL:DP 0/1:50,0,78:15 0/1:111,0,63:13 0/1:24,0,64:9 0/1:13,0,18:2 0/1:25,0,9:4 0/1:26,0,55:5 0/1:105,0,108:20

3 5214601 . T A 999 . . GT:PL:DP 0/1:255,0,255:34 0/1:255,0,255:35 0/1:206,0,250:26 0/1:103,0,81:8 0/1:78,0,53:5 0/1:66,6,0:2 0/1:255,0,255:33

3 5216346 . C T 999 . . GT:PL:DP 0/1:133,0,204:112 0/1:197,0,199:89 0/1:216,0,191:100 0/1:99,0,109:17 0/1:66,0,83:8 0/1:19,0,51:6 0/1:189,0,195:76

3 5225914 . A G 999 . . GT:PL:DP 0/1:35,3,0:1 0/1:63,6,0:2 0/1:100,12,0:4 0/1:34,3,0:1 0/1:30,3,0:1 0/1:33,3,0:1 0/1:59,6,0:2

3 5227137 . T C 999 . . GT:PL:DP 0/1:248,0,177:35 0/1:224,0,209:33 0/1:165,0,221:36 0/1:13,0,59:4 0/1:23,3,0:1 0/1:67,6,0:2 0/1:211,0,186:39

3 5330789 . G A 999 . . GT:PL:DP 0/1:182,0,255:34 0/1:255,0,255:52 0/1:255,0,252:36 0/1:93,0,87:6 0/1:92,0,104:9 0/1:79,6,0:2 0/1:255,0,255:59

3 5357515 . G A 999 . . GT:PL:DP 0/1:190,0,222:64 0/1:226,0,144:69 0/1:255,0,243:90 0/1:52,0,131:8 0/1:127,0,96:20 0/1:31,0,119:18 0/1:255,0,255:120

3 5357706 . T A 999 . . GT:PL:DP 0/1:101,0,185:238 0/1:60,0,164:229 0/1:112,0,227:227 0/1:91,0,127:151 0/1:106,0,70:56 0/1:117,0,65:96 0/1:148,0,166:234

3 5358882 . G A 999 . . GT:PL:DP 0/1:255,0,255:233 0/1:255,0,230:231 0/1:255,0,255:235 0/1:255,0,255:135 0/1:121,0,255:146 0/1:166,0,255:187 0/1:255,0,251:228

3 5358897 . A T 999 . . GT:PL:DP 0/1:255,0,255:229 0/1:216,0,175:232 0/1:255,0,255:230 0/1:255,0,255:110 0/1:124,0,255:121 0/1:169,0,255:140 0/1:237,0,190:225

3 5359047 . G T 999 . . GT:PL:DP 0/1:130,0,59:224 0/1:124,0,42:214 0/1:173,0,126:232 0/1:173,0,165:95 0/1:237,0,235:154 0/1:238,0,255:105 0/1:247,0,171:217

3 5359048 . G T 999 . . GT:PL:DP 0/1:121,0,48:223 0/1:121,0,33:214 0/1:166,0,127:232 0/1:170,0,160:95 0/1:238,0,234:154 0/1:222,0,255:104 0/1:247,0,180:217

3 5492732 . C T 999 . . GT:PL:DP 0/1:255,0,246:240 0/1:255,0,255:240 0/1:225,6,0:242 0/1:255,0,255:159 0/1:247,0,129:121 0/1:170,0,29:210 0/1:255,0,144:246

3 5492873 . T G 999 . . GT:PL:DP 0/1:255,0,235:229 0/1:255,0,166:230 0/1:239,0,105:224 0/1:255,0,172:155 0/1:255,0,240:103 0/1:209,0,75:177 0/1:255,0,165:240

3 5492893 . A G 999 . . GT:PL:DP 0/1:255,0,236:221 0/1:255,0,158:232 0/1:255,0,168:227 0/1:255,0,171:141 0/1:255,0,252:102 0/1:230,0,119:160 0/1:246,0,111:245

3 5619080 . T A 999 . . GT:PL:DP 0/1:92,0,119:30 0/1:77,0,105:22 0/1:63,0,41:10 0/1:46,0,11:5 0/1:14,0,2:2 0/1:15,3,0:1 0/1:97,0,67:29

3 5924202 . T C 999 . . GT:PL:DP 0/1:63,0,139:93 0/1:75,0,151:100 0/1:28,0,161:84 0/1:30,0,83:27 0/1:17,0,14:17 0/1:7,0,16:12 0/1:107,0,159:159

3 5924586 . G A 999 . . GT:PL:DP 0/1:240,0,215:68 0/1:131,0,215:70 0/1:18,0,207:81 0/1:42,0,131:15 0/1:31,0,46:13 0/1:60,0,17:12 0/1:70,0,178:136

3 5924587 . C T 999 . . GT:PL:DP 0/1:243,0,212:68 0/1:165,0,231:71 0/1:66,0,228:80 0/1:67,0,122:15 0/1:35,0,45:13 0/1:60,0,17:12 0/1:160,0,230:137

3 5924662 . C A 999 . . GT:PL:DP 0/1:238,0,164:73 0/1:98,0,242:66 0/1:47,0,255:102 0/1:23,0,175:13 0/1:92,0,68:17 0/1:44,0,130:19 0/1:53,0,215:122

3 5937153 . G A 999 . . GT:PL:DP 0/1:255,0,255:222 0/1:255,0,201:214 0/1:255,0,255:221 0/1:255,0,196:172 0/1:160,0,99:111 0/1:192,0,142:88 0/1:255,0,255:229

3 5972808 . T C 999 . . GT:PL:DP 0/1:130,0,255:43 0/1:243,0,178:38 0/1:143,0,208:30 0/1:95,0,18:6 0/1:37,18,9:4 0/1:47,6,0:2 0/1:255,0,116:49

3 6119219 . A G 999 . . GT:PL:DP 0/1:103,0,255:32 0/1:79,0,237:23 0/1:20,0,212:17 0/1:38,0,167:11 0/1:23,0,55:7 0/1:52,0,255:33 0/1:180,0,255:46

3 6121236 . A T 999 . . GT:PL:DP 0/1:29,0,250:31 0/1:149,0,255:45 0/1:83,0,195:19 0/1:43,0,82:6 0/1:21,3,0:1 0/1:61,9,0:3 0/1:62,0,251:34

3 6121277 . A G 999 . . GT:PL:DP 0/1:134,0,255:38 0/1:196,0,219:42 0/1:87,0,251:26 0/1:22,0,108:6 0/1:32,18,15:2 0/1:38,16,10:3 0/1:107,0,255:43

3 6134211 . C G 999 . . GT:PL:DP 0/1:255,0,255:167 0/1:255,0,222:151 0/1:255,0,255:152 0/1:122,0,133:31 0/1:255,0,130:34 0/1:221,0,168:51 0/1:255,0,255:216

3 6134296 . G A 999 . . GT:PL:DP 0/1:255,0,255:105 0/1:255,0,252:96 0/1:255,0,255:104 0/1:142,0,151:20 0/1:57,0,118:27 0/1:33,0,76:33 0/1:255,0,255:107

3 6134305 . G C 999 . . GT:PL:DP 0/1:255,0,234:87 0/1:255,0,240:85 0/1:255,0,255:100 0/1:136,0,153:19 0/1:49,0,97:24 0/1:37,0,29:27 0/1:255,0,255:88

3 6134811 . A T 999 . . GT:PL:DP 0/1:184,0,154:143 0/1:182,0,109:112 0/1:214,0,134:98 0/1:94,0,177:18 0/1:61,0,12:12 0/1:59,0,23:20 0/1:255,0,147:238

3 6135046 . T G 999 . . GT:PL:DP 0/1:180,0,228:120 0/1:154,0,228:85 0/1:129,0,255:90 0/1:123,0,167:24 0/1:38,0,164:13 0/1:61,0,124:13 0/1:173,0,255:223

3 6182318 . G A 999 . . GT:PL:DP 0/1:218,0,228:72 0/1:206,0,255:116 0/1:255,0,255:81 0/1:157,0,116:17 0/1:20,0,38:21 0/1:24,0,48:11 0/1:247,0,231:210

3 6182414 . G T 999 . . GT:PL:DP 0/1:255,0,216:235 0/1:255,0,175:228 0/1:255,0,137:218 0/1:255,0,217:71 0/1:124,0,202:74 0/1:53,0,78:62 0/1:255,0,164:245

3 6182519 . A C 999 . . GT:PL:DP 0/1:255,0,226:152 0/1:255,0,255:185 0/1:255,0,255:173 0/1:255,0,233:63 0/1:255,0,252:96 0/1:255,0,255:97 0/1:255,0,255:205

3 6182579 . A G 999 . . GT:PL:DP 0/1:255,0,172:199 0/1:255,0,223:199 0/1:255,0,185:198 0/1:255,0,165:64 0/1:82,0,156:30 0/1:144,0,56:43 0/1:255,0,176:201

3 6182588 . A G 999 . . GT:PL:DP 0/1:255,0,146:205 0/1:255,0,217:199 0/1:255,0,196:197 0/1:255,0,157:61 0/1:52,0,146:31 0/1:94,0,53:39 0/1:255,0,182:202

3 6182784 . C T 999 . . GT:PL:DP 0/1:255,0,131:49 0/1:255,0,255:68 0/1:255,0,255:51 0/1:147,0,82:10 0/1:128,0,180:20 0/1:120,0,155:17 0/1:255,0,217:143

3 6182787 . A G 999 . . GT:PL:DP 0/1:255,0,130:49 0/1:255,0,255:68 0/1:255,0,255:52 0/1:147,0,82:10 0/1:128,0,179:20 0/1:119,0,177:17 0/1:255,0,211:145

3 6182817 . A T 999 . . GT:PL:DP 0/1:255,0,176:50 0/1:239,0,255:74 0/1:255,0,255:51 0/1:138,0,75:10 0/1:123,0,202:22 0/1:166,0,176:20 0/1:255,0,202:145

3 6182851 . T C 999 . . GT:PL:DP 0/1:255,0,169:52 0/1:255,0,255:71 0/1:255,0,255:47 0/1:162,0,16:9 0/1:193,0,114:18 0/1:86,0,155:17 0/1:252,0,138:143

3 6182878 . A T 999 . . GT:PL:DP 0/1:255,0,193:48 0/1:255,0,255:64 0/1:255,0,255:46 0/1:160,0,16:9 0/1:106,0,92:16 0/1:146,0,139:26 0/1:255,0,127:127

3 6182883 . T A 999 . . GT:PL:DP 0/1:255,0,182:50 0/1:255,0,247:61 0/1:255,0,255:46 0/1:151,0,16:9 0/1:106,0,72:14 0/1:136,0,129:23 0/1:255,0,91:123

3 6182957 . T A 999 . . GT:PL:DP 0/1:137,0,139:29 0/1:84,0,160:21 0/1:129,0,120:18 0/1:118,10,0:6 0/1:46,0,100:7 0/1:64,0,4:8 0/1:125,0,62:53

3 6230983 . T C 999 . . GT:PL:DP 0/1:149,0,163:122 0/1:124,0,194:140 0/1:93,0,146:219 0/1:53,0,56:10 0/1:159,0,189:58 0/1:138,0,226:88 0/1:183,0,223:208

3 6231401 . A C 999 . . GT:PL:DP 0/1:84,0,255:241 0/1:110,0,255:224 0/1:251,0,255:235 0/1:214,0,255:100 0/1:132,0,255:111 0/1:129,0,255:122 0/1:248,0,255:231

3 6231405 . T G 999 . . GT:PL:DP 0/1:90,0,255:242 0/1:34,0,255:227 0/1:255,0,255:233 0/1:243,0,255:104 0/1:152,0,255:109 0/1:140,0,255:122 0/1:249,0,255:233

3 6231424 . G A 999 . . GT:PL:DP 0/1:231,0,255:220 0/1:101,0,255:208 0/1:255,0,255:216 0/1:222,0,255:102 0/1:192,0,255:129 0/1:195,0,255:124 0/1:255,0,255:225

3 6231528 . C T 999 . . GT:PL:DP 0/1:255,0,255:235 0/1:255,0,255:212 0/1:255,0,255:228 0/1:255,0,177:87 0/1:255,0,221:56 0/1:255,0,171:66 0/1:255,0,255:229

3 6234280 . C A 999 . . GT:PL:DP 0/1:230,0,255:95 0/1:255,0,231:59 0/1:150,0,236:89 0/1:36,0,176:15 0/1:22,0,110:14 0/1:64,0,18:7 0/1:10,0,188:172

3 6234362 . T C 999 . . GT:PL:DP 0/1:255,0,255:160 0/1:255,0,255:96 0/1:255,0,255:158 0/1:144,0,206:24 0/1:153,0,202:48 0/1:228,0,233:60 0/1:255,0,255:203

3 6235957 . C A 999 . . GT:PL:DP 0/1:245,0,15:23 0/1:199,0,82:19 0/1:215,0,0:21 0/1:75,0,72:7 0/1:22,0,25:2 0/1:33,0,43:4 0/1:189,0,52:23

3 6235961 . T A 999 . . GT:PL:DP 0/1:229,0,21:21 0/1:193,0,81:18 0/1:210,0,0:21 0/1:75,0,72:7 0/1:22,0,25:2 0/1:12,0,43:3 0/1:201,0,52:23

3 6277746 . A G 999 . . GT:PL:DP 0/1:181,0,255:31 0/1:163,0,255:31 0/1:221,0,243:28 0/1:18,0,124:6 0/1:97,9,0:3 0/1:126,2,0:8 0/1:199,0,247:29

3 6355745 . C G 999 . . GT:PL:DP 0/1:242,0,255:112 0/1:255,0,143:120 0/1:255,0,180:95 0/1:87,0,58:10 0/1:103,0,15:9 0/1:104,0,71:12 0/1:255,0,255:119

3 6355814 . C T 999 . . GT:PL:DP 0/1:255,0,255:70 0/1:255,0,168:61 0/1:255,0,203:58 0/1:39,0,96:7 0/1:16,0,61:17 0/1:48,0,122:16 0/1:255,0,255:82

3 6356213 . T A 999 . . GT:PL:DP 0/1:115,0,255:267 0/1:228,0,255:267 0/1:37,0,204:267 0/1:156,0,255:129 0/1:80,0,255:63 0/1:175,0,255:122 0/1:170,0,255:262

3 6402091 . G A 999 . . GT:PL:DP 0/1:134,0,174:18 0/1:113,0,113:13 0/1:158,0,69:16 0/1:20,0,43:3 0/1:43,6,0:2 0/1:25,0,28:2 0/1:220,0,100:28

3 6408322 . C A 999 . . GT:PL:DP 0/1:255,0,255:217 0/1:236,0,255:224 0/1:255,0,255:210 0/1:247,0,255:116 0/1:124,0,237:154 0/1:133,0,255:109 0/1:212,0,255:230

3 6408348 . A T 999 . . GT:PL:DP 0/1:255,0,255:239 0/1:251,0,255:236 0/1:255,0,255:231 0/1:255,0,255:120 0/1:129,0,186:142 0/1:147,0,255:107 0/1:255,0,255:239

3 6408408 . A T 999 . . GT:PL:DP 0/1:226,0,255:149 0/1:222,0,255:122 0/1:255,0,255:161 0/1:146,0,255:71 0/1:62,0,149:19 0/1:82,0,226:27 0/1:191,0,255:161

3 6408470 . G A 999 . . GT:PL:DP 0/1:202,0,255:221 0/1:100,0,233:229 0/1:15,0,207:224 0/1:112,0,255:74 0/1:37,0,186:37 0/1:47,0,255:50 0/1:20,0,227:234

3 6408722 . G T 999 . . GT:PL:DP 0/1:26,0,135:49 0/1:89,0,167:45 0/1:60,0,195:42 0/1:55,0,129:16 0/1:53,0,63:7 0/1:24,0,176:16 0/1:82,0,211:65

3 6520052 . C T 999 . . GT:PL:DP 0/1:91,0,132:14 0/1:48,0,76:7 0/1:95,0,133:19 0/1:53,0,76:5 0/1:50,0,70:9 0/1:69,0,37:11 0/1:111,0,199:21

3 6668878 . T A 999 . . GT:PL:DP 0/1:230,0,166:22 0/1:251,0,251:33 0/1:255,0,255:34 0/1:107,0,62:8 0/1:82,0,24:5 0/1:53,0,42:4 0/1:255,0,255:43

3 6680586 . G A 223.525 . . GT:PL:DP 0/1:39,9,0:3 0/1:19,6,0:2 0/1:33,6,0:2 0/1:34,6,0:2 0/1:9,3,0:1 0/1:14,3,0:1 0/1:35,3,0:4

3 6716990 . C T 999 . . GT:PL:DP 0/1:153,0,202:38 0/1:202,0,255:57 0/1:61,0,212:27 0/1:101,0,30:10 0/1:49,0,99:6 0/1:96,0,34:6 0/1:179,0,190:34

3 6758418 . C T 999 . . GT:PL:DP 0/1:255,0,255:114 0/1:255,0,255:219 0/1:255,0,0:79 0/1:255,0,210:38 0/1:243,0,173:25 0/1:255,0,92:24 0/1:255,0,205:138

3 6812776 . A C 999 . . GT:PL:DP 0/1:105,0,255:60 0/1:206,0,255:66 0/1:121,0,255:62 0/1:98,0,167:24 0/1:76,0,70:12 0/1:97,0,195:28 0/1:255,0,255:108

3 6813118 . C A 999 . . GT:PL:DP 0/1:115,0,223:50 0/1:20,0,240:27 0/1:115,0,226:37 0/1:32,0,85:9 0/1:40,0,39:8 0/1:14,0,51:3 0/1:110,0,182:95

3 6813119 . A T 999 . . GT:PL:DP 0/1:121,0,184:53 0/1:148,0,160:28 0/1:79,0,223:37 0/1:34,0,113:11 0/1:39,0,40:8 0/1:10,0,47:3 0/1:68,0,178:95

3 6819581 . T C 999 . . GT:PL:DP 0/1:228,0,255:49 0/1:255,0,255:85 0/1:193,0,255:131 0/1:149,0,96:13 0/1:24,0,82:9 0/1:71,0,35:12 0/1:197,0,255:118

3 6823050 . G A 999 . . GT:PL:DP 0/1:210,0,255:147 0/1:255,0,255:108 0/1:134,0,255:168 0/1:165,0,255:39 0/1:86,0,255:24 0/1:125,0,255:43 0/1:255,0,255:182

3 6823113 . A G 999 . . GT:PL:DP 0/1:197,0,255:154 0/1:255,0,255:115 0/1:178,0,255:161 0/1:121,0,255:33 0/1:91,0,194:13 0/1:99,0,255:46 0/1:255,0,255:175

3 6823155 . A G 999 . . GT:PL:DP 0/1:200,0,255:155 0/1:255,0,255:114 0/1:173,0,255:168 0/1:130,0,255:30 0/1:79,0,232:18 0/1:220,0,255:51 0/1:255,0,255:181

3 6823375 . G A 999 . . GT:PL:DP 0/1:63,0,255:193 0/1:255,0,255:97 0/1:143,0,255:168 0/1:82,0,255:37 0/1:48,0,255:30 0/1:66,0,255:61 0/1:255,0,255:171

3 6888085 . C T 999 . . GT:PL:DP 0/1:139,0,213:36 0/1:211,0,163:35 0/1:151,0,199:28 0/1:38,6,0:2 0/1:37,0,12:3 0/1:16,0,78:7 0/1:192,0,230:49

3 6891734 . A C 999 . . GT:PL:DP 0/1:70,0,68:10 0/1:184,0,1:10 0/1:175,0,60:12 0/1:58,6,0:2 0/1:47,6,0:2 0/1:40,2,0:4 0/1:148,0,68:13

3 6935731 . A C 999 . . GT:PL:DP 0/1:178,0,199:30 0/1:22,0,118:14 0/1:175,0,188:28 0/1:9,0,36:3 0/1:34,0,27:4 0/1:85,0,24:4 0/1:168,0,155:35

3 7127187 . G A 999 . . GT:PL:DP 0/1:81,0,97:11 0/1:37,3,0:1 0/1:179,0,58:23 0/1:28,0,22:3 0/1:69,0,28:5 0/1:50,0,129:8 0/1:180,0,255:90

3 7283733 . A T 999 . . GT:PL:DP 0/1:195,0,255:96 0/1:255,0,255:95 0/1:255,0,255:69 0/1:31,0,255:26 0/1:73,0,177:13 0/1:88,0,227:27 0/1:131,0,255:201

3 7350755 . A G 999 . . GT:PL:DP 0/1:48,0,30:4 0/1:46,0,183:26 0/1:46,0,124:18 0/1:14,0,56:5 0/1:21,0,31:3 0/1:15,0,44:4 0/1:155,0,140:24

3 7443508 . T C 999 . . GT:PL:DP 0/1:228,0,117:40 0/1:142,0,211:38 0/1:207,0,152:41 0/1:103,0,77:11 0/1:235,0,0:33 0/1:255,0,3:23 0/1:255,0,255:107

3 7462707 . T C 999 . . GT:PL:DP 0/1:213,0,255:71 0/1:104,0,193:36 0/1:243,0,202:46 0/1:17,0,161:11 0/1:14,0,187:28 0/1:55,0,107:15 0/1:52,0,249:84

3 7567401 . G C 999 . . GT:PL:DP 0/1:94,0,155:39 0/1:105,0,71:20 0/1:59,0,126:22 0/1:25,0,16:7 0/1:51,0,15:4 0/1:24,0,46:5 0/1:55,0,217:22

3 7575220 . G A 999 . . GT:PL:DP 0/1:198,0,255:239 0/1:150,0,255:227 0/1:148,0,255:225 0/1:200,0,255:58 0/1:10,0,255:38 0/1:26,0,255:63 0/1:255,0,255:237

3 7639161 . C A 999 . . GT:PL:DP 0/1:255,0,131:194 0/1:255,0,66:183 0/1:255,0,204:199 0/1:255,0,179:72 0/1:196,0,164:56 0/1:165,0,101:47 0/1:221,0,180:194

3 7645815 . T C 999 . . GT:PL:DP 0/1:243,0,206:38 0/1:179,0,255:40 0/1:223,0,255:40 0/1:19,0,31:2 0/1:52,6,0:2 0/1:53,6,0:2 0/1:255,0,255:67

3 7699863 . T G 999 . . GT:PL:DP 0/1:119,0,255:124 0/1:96,0,255:108 0/1:64,0,255:116 0/1:41,0,181:23 0/1:86,0,59:26 0/1:195,0,95:23 0/1:78,0,255:169

3 7864136 . T A 999 . . GT:PL:DP 0/1:25,0,74:29 0/1:34,0,64:24 0/1:41,0,52:28 0/1:9,0,39:10 0/1:36,0,13:12 0/1:22,1,0:2 0/1:92,0,42:83

3 7926304 . G A 999 . . GT:PL:DP 0/1:255,0,255:57 0/1:128,0,255:79 0/1:255,0,255:65 0/1:60,0,203:12 0/1:102,0,34:8 0/1:117,0,254:20 0/1:251,0,255:78

3 7926355 . G C 999 . . GT:PL:DP 0/1:255,0,255:57 0/1:227,0,255:101 0/1:255,0,255:75 0/1:81,0,212:13 0/1:103,0,99:8 0/1:39,0,249:21 0/1:255,0,255:97

3 7926440 . T A 999 . . GT:PL:DP 0/1:202,0,255:34 0/1:255,0,255:97 0/1:255,0,255:77 0/1:82,0,115:8 0/1:22,0,255:17 0/1:97,0,205:22 0/1:255,0,255:74

3 7984047 . G C 999 . . GT:PL:DP 0/1:195,0,139:201 0/1:212,0,182:202 0/1:197,0,255:204 0/1:130,0,103:86 0/1:104,10,0:32 0/1:26,0,0:60 0/1:201,0,255:190

3 8042897 . C A 999 . . GT:PL:DP 0/1:67,0,104:45 0/1:59,0,33:39 0/1:124,0,47:49 0/1:50,0,75:11 0/1:12,0,95:7 0/1:7,0,65:11 0/1:71,5,0:20

3 8043391 . T A 999 . . GT:PL:DP 0/1:255,0,255:184 0/1:255,0,255:182 0/1:255,0,255:189 0/1:255,0,235:42 0/1:154,0,255:49 0/1:179,0,255:50 0/1:255,0,255:194

3 8166559 . A G 999 . . GT:PL:DP 0/1:243,0,242:34 0/1:221,0,191:31 0/1:250,0,172:39 0/1:119,0,70:9 0/1:76,0,109:9 0/1:100,0,115:10 0/1:228,0,255:39

3 8166569 . G T 999 . . GT:PL:DP 0/1:232,0,252:33 0/1:225,0,227:35 0/1:234,0,172:39 0/1:131,0,83:10 0/1:76,0,102:9 0/1:81,0,116:9 0/1:241,0,255:41

3 8411324 . T G 999 . . GT:PL:DP 0/1:255,0,255:213 0/1:229,0,245:196 0/1:229,0,255:190 0/1:140,0,255:92 0/1:44,0,104:15 0/1:53,0,129:33 0/1:249,0,255:221

3 8413388 . G T 999 . . GT:PL:DP 0/1:64,0,142:22 0/1:67,0,59:16 0/1:43,0,81:17 0/1:37,0,38:6 0/1:39,0,84:7 0/1:59,0,152:9 0/1:80,0,161:23

3 8413399 . A G 999 . . GT:PL:DP 0/1:68,0,142:22 0/1:83,0,29:17 0/1:66,0,77:16 0/1:33,0,41:5 0/1:41,0,51:6 0/1:59,0,145:9 0/1:89,0,150:23

3 8425907 . G A 999 . . GT:PL:DP 0/1:255,0,245:48 0/1:6,0,255:22 0/1:127,0,255:73 0/1:25,0,70:4 0/1:121,0,54:10 0/1:255,0,108:26 0/1:255,0,255:83

3 8523574 . C G 999 . . GT:PL:DP 0/1:128,0,61:33 0/1:142,0,122:51 0/1:169,0,125:51 0/1:22,0,104:11 0/1:57,8,0:5 0/1:124,0,21:10 0/1:157,0,155:102

3 8523591 . C T 999 . . GT:PL:DP 0/1:113,0,112:40 0/1:100,0,123:62 0/1:51,0,158:65 0/1:35,0,78:10 0/1:19,0,24:7 0/1:109,0,55:10 0/1:112,0,161:92

3 8680395 . A T 999 . . GT:PL:DP 0/1:137,0,55:23 0/1:121,0,11:12 0/1:151,0,5:23 0/1:21,0,28:5 0/1:37,0,44:7 0/1:27,0,57:10 0/1:171,0,11:35

3 8680417 . A G 999 . . GT:PL:DP 0/1:113,0,64:22 0/1:42,0,14:6 0/1:118,0,55:20 0/1:21,0,28:5 0/1:24,0,29:6 0/1:8,0,46:9 0/1:97,0,57:25

3 8879551 . A G 999 . . GT:PL:DP 0/1:255,0,255:221 0/1:255,0,255:218 0/1:255,0,255:224 0/1:255,0,255:151 0/1:112,0,255:64 0/1:202,0,194:75 0/1:255,0,255:213

3 8879632 . A G 999 . . GT:PL:DP 0/1:255,0,255:225 0/1:221,0,255:222 0/1:255,0,255:219 0/1:121,0,255:83 0/1:126,0,214:49 0/1:135,0,161:35 0/1:255,0,255:213

3 8901966 . T G 999 . . GT:PL:DP 0/1:132,0,255:42 0/1:130,0,255:36 0/1:95,0,255:43 0/1:19,0,114:7 0/1:50,3,0:5 0/1:78,4,0:9 0/1:171,0,255:64

3 8922480 . C G 999 . . GT:PL:DP 0/1:227,0,109:24 0/1:212,0,188:36 0/1:218,0,177:42 0/1:11,0,45:3 0/1:11,0,50:3 0/1:27,3,0:1 0/1:187,0,255:51

3 8932433 . T A 999 . . GT:PL:DP 0/1:112,0,161:15 0/1:47,0,144:8 0/1:43,0,197:15 0/1:36,3,0:1 0/1:90,0,51:5 0/1:33,3,0:1 0/1:224,0,230:32

3 8932544 . C T 999 . . GT:PL:DP 0/1:44,0,125:14 0/1:106,0,128:16 0/1:133,0,143:22 0/1:33,6,0:2 0/1:8,0,75:4 0/1:27,0,35:2 0/1:106,0,130:30

3 8932595 . C A 999 . . GT:PL:DP 0/1:14,0,118:9 0/1:125,0,125:17 0/1:123,0,127:19 0/1:57,9,0:3 0/1:29,3,0:1 0/1:20,0,28:3 0/1:145,0,10:16

3 8981138 . C T 999 . . GT:PL:DP 0/1:223,0,112:34 0/1:157,0,197:28 0/1:206,0,114:22 0/1:24,0,53:3 0/1:59,0,26:6 0/1:104,0,8:7 0/1:203,0,177:40

3 8994420 . T A 999 . . GT:PL:DP 0/1:61,0,255:122 0/1:255,0,255:128 0/1:255,0,255:88 0/1:105,0,255:20 0/1:153,0,141:15 0/1:28,0,197:11 0/1:255,0,255:88

3 8994486 . C T 999 . . GT:PL:DP 0/1:104,0,255:122 0/1:255,0,255:130 0/1:255,0,255:91 0/1:123,0,249:18 0/1:161,0,128:15 0/1:88,0,234:16 0/1:255,0,255:93

3 9011645 . A G 999 . . GT:PL:DP 0/1:255,0,230:72 0/1:255,0,255:95 0/1:255,0,246:72 0/1:157,0,50:15 0/1:141,0,119:22 0/1:192,0,57:40 0/1:255,0,255:157

3 9015247 . A C 999 . . GT:PL:DP 0/1:66,0,96:33 0/1:133,0,145:71 0/1:133,52,132:46 0/1:121,70,73:7 0/1:121,0,108:20 0/1:84,0,16:7 0/1:117,0,95:92

3 9015512 . C A 999 . . GT:PL:DP 0/1:255,0,255:195 0/1:255,0,202:188 0/1:255,0,255:201 0/1:167,0,161:72 0/1:177,0,182:210 0/1:156,0,152:202 0/1:255,0,255:185

3 9058447 . G T 999 . . GT:PL:DP 0/1:255,0,255:127 0/1:136,0,255:88 0/1:255,0,255:59 0/1:70,0,197:17 0/1:126,0,217:17 0/1:255,0,255:35 0/1:255,0,255:69

3 9096529 . C T 999 . . GT:PL:DP 0/1:87,0,175:42 0/1:158,0,115:22 0/1:117,0,114:43 0/1:67,0,97:13 0/1:11,0,14:19 0/1:7,2,0:20 0/1:252,0,218:141

3 9096658 . G A 999 . . GT:PL:DP 0/1:236,0,205:47 0/1:176,0,119:26 0/1:179,0,162:36 0/1:86,0,19:9 0/1:46,0,109:11 0/1:62,0,179:32 0/1:255,0,212:138

3 9167424 . T C 999 . . GT:PL:DP 0/1:228,0,155:22 0/1:217,0,215:22 0/1:117,0,238:20 0/1:91,9,0:3 0/1:70,6,0:2 0/1:112,9,0:3 0/1:255,0,255:56

3 9198171 . T C 999 . . GT:PL:DP 0/1:255,0,255:112 0/1:143,0,255:211 0/1:255,0,255:166 0/1:89,0,255:31 0/1:121,0,242:22 0/1:218,0,255:38 0/1:231,0,255:228

3 9202404 . T A 999 . . GT:PL:DP 0/1:143,6,0:14 0/1:124,0,60:14 0/1:146,0,75:24 0/1:41,0,21:3 0/1:7,0,24:5 0/1:9,3,0:1 0/1:177,0,73:43

3 9282658 . T A 999 . . GT:PL:DP 0/1:33,0,162:12 0/1:21,0,221:23 0/1:102,0,146:21 0/1:48,0,117:9 0/1:32,3,0:1 0/1:25,0,26:2 0/1:7,0,166:19

3 9335891 . G A 999 . . GT:PL:DP 0/1:255,0,120:42 0/1:255,0,142:41 0/1:255,0,63:33 0/1:95,0,9:7 0/1:114,0,20:8 0/1:159,0,90:16 0/1:255,0,244:68

3 9335985 . C T 999 . . GT:PL:DP 0/1:152,0,67:23 0/1:156,0,167:33 0/1:28,0,190:15 0/1:34,0,44:4 0/1:62,6,0:2 0/1:141,0,15:8 0/1:156,0,255:42

3 9377427 . T C 999 . . GT:PL:DP 0/1:109,0,145:63 0/1:112,0,237:64 0/1:134,0,200:74 0/1:210,0,254:147 0/1:64,0,111:32 0/1:70,0,66:26 0/1:139,0,220:70

3 9445672 . C T 999 . . GT:PL:DP 0/1:111,0,143:25 0/1:24,0,99:22 0/1:40,0,108:26 0/1:27,3,0:1 0/1:27,0,53:4 0/1:119,2,0:9 0/1:127,0,49:43

3 9465440 . C A 999 . . GT:PL:DP 0/1:46,0,255:37 0/1:73,0,201:31 0/1:96,0,255:32 0/1:34,0,143:10 0/1:10,0,43:7 0/1:10,0,151:10 0/1:96,0,255:72

3 9486991 . C T 999 . . GT:PL:DP 0/1:86,0,93:144 0/1:232,0,50:111 0/1:240,0,92:143 0/1:53,1,0:15 0/1:137,0,43:14 0/1:99,0,56:15 0/1:249,0,224:124

3 9505029 . A G 999 . . GT:PL:DP 0/1:20,0,96:10 0/1:7,0,134:13 0/1:109,0,60:14 0/1:19,3,0:1 0/1:19,0,33:2 0/1:44,6,0:2 0/1:80,0,158:21

3 9505030 . C G 999 . . GT:PL:DP 0/1:20,0,96:10 0/1:7,0,134:13 0/1:107,0,61:14 0/1:19,3,0:1 0/1:19,0,35:2 0/1:44,6,0:2 0/1:93,0,162:21

3 9505033 . C G 999 . . GT:PL:DP 0/1:23,0,67:9 0/1:13,0,106:11 0/1:105,0,59:15 0/1:19,3,0:1 0/1:19,0,33:2 0/1:61,9,0:3 0/1:93,0,159:21

3 9505034 . T C 999 . . GT:PL:DP 0/1:23,0,67:9 0/1:44,0,86:12 0/1:105,0,49:15 0/1:19,3,0:1 0/1:19,0,33:2 0/1:59,9,0:3 0/1:108,0,162:23

3 9505035 . G C 999 . . GT:PL:DP 0/1:23,0,67:9 0/1:65,0,104:13 0/1:105,0,59:15 0/1:19,3,0:1 0/1:19,0,33:2 0/1:65,9,0:3 0/1:110,0,161:22

3 9505036 . T C 999 . . GT:PL:DP 0/1:23,0,67:9 0/1:52,0,104:13 0/1:105,0,59:15 0/1:19,3,0:1 0/1:19,0,31:2 0/1:68,9,0:3 0/1:119,0,161:22

3 9505037 . C T 999 . . GT:PL:DP 0/1:23,0,67:9 0/1:84,0,102:14 0/1:105,0,59:15 0/1:19,3,0:1 0/1:19,0,31:2 0/1:65,9,0:3 0/1:119,0,161:22

3 9515640 . A T 999 . . GT:PL:DP 0/1:182,0,240:31 0/1:91,0,255:34 0/1:255,0,120:32 0/1:53,0,102:9 0/1:28,0,44:4 0/1:56,0,50:5 0/1:255,1,0:38

3 9521064 . T C 999 . . GT:PL:DP 0/1:99,0,39:35 0/1:105,0,17:24 0/1:171,0,84:50 0/1:77,0,5:7 0/1:32,0,17:5 0/1:38,4,0:4 0/1:184,0,97:55

3 9573244 . A T 999 . . GT:PL:DP 0/1:91,6,0:11 0/1:109,0,79:34 0/1:28,0,95:12 0/1:8,0,9:2 0/1:10,0,10:2 0/1:14,4,85:7 0/1:81,0,30:41

3 9577376 . C A 999 . . GT:PL:DP 0/1:69,0,68:27 0/1:43,0,54:23 0/1:58,0,18:28 0/1:21,0,1:4 0/1:31,0,17:6 0/1:7,0,46:7 0/1:50,0,13:17

3 9582230 . G A 999 . . GT:PL:DP 0/1:178,0,133:16 0/1:98,0,200:16 0/1:180,0,151:16 0/1:49,0,25:3 0/1:35,3,0:1 0/1:55,6,0:2 0/1:208,0,201:30

3 9668340 . T C 999 . . GT:PL:DP 0/1:36,0,117:17 0/1:42,0,109:16 0/1:76,0,92:14 0/1:19,0,44:5 0/1:16,0,9:3 0/1:31,0,23:5 0/1:113,0,113:24

3 9716905 . A C 999 . . GT:PL:DP 0/1:136,0,255:87 0/1:132,0,255:54 0/1:113,0,255:104 0/1:49,0,255:15 0/1:19,0,53:4 0/1:61,0,154:18 0/1:173,0,255:111

3 9777501 . T C 999 . . GT:PL:DP 0/1:39,0,169:15 0/1:43,0,70:10 0/1:27,0,201:22 0/1:22,0,29:4 0/1:26,0,30:3 0/1:7,0,86:6 0/1:109,0,176:57

3 9777529 . A C 999 . . GT:PL:DP 0/1:137,0,184:28 0/1:154,0,160:27 0/1:82,0,239:33 0/1:121,0,54:11 0/1:146,0,67:22 0/1:82,0,70:17 0/1:216,0,255:121

3 10000158 . C G 999 . . GT:PL:DP 0/1:156,0,255:72 0/1:255,0,97:48 0/1:117,0,255:39 0/1:77,0,255:23 0/1:41,0,147:11 0/1:127,0,140:12 0/1:255,0,255:89

3 10001738 . G A 999 . . GT:PL:DP 0/1:194,0,61:42 0/1:65,0,168:34 0/1:199,0,67:53 0/1:89,0,166:15 0/1:90,0,9:11 0/1:52,0,61:12 0/1:96,0,255:132

4 1372 . C T 999 . . GT:PL:DP 0/1:255,0,65:44 0/1:255,0,200:58 0/1:255,0,138:56 0/1:131,0,67:12 0/1:155,0,148:21 0/1:131,0,95:14 0/1:255,0,244:99

4 1694 . G C 999 . . GT:PL:DP 0/1:45,0,255:62 0/1:18,0,255:40 0/1:72,0,255:54 0/1:7,0,154:9 0/1:53,0,168:9 0/1:14,0,163:11 0/1:194,0,242:45

4 84235 . G T 999 . . GT:PL:DP 0/1:255,0,255:201 0/1:255,0,255:211 0/1:255,0,255:205 0/1:255,0,255:99 0/1:255,0,255:98 0/1:255,0,255:95 0/1:255,0,140:187

4 84243 . A G 999 . . GT:PL:DP 0/1:195,0,255:197 0/1:185,0,255:207 0/1:216,0,255:202 0/1:156,0,255:96 0/1:239,0,255:96 0/1:213,0,255:93 0/1:37,0,255:188

4 109611 . A G 999 . . GT:PL:DP 0/1:234,0,152:86 0/1:255,0,175:75 0/1:246,0,255:110 0/1:60,0,78:10 0/1:173,0,8:33 0/1:172,0,112:87 0/1:255,0,228:143

4 113764 . G A 80.4593 . . GT:PL:DP 0/1:24,3,0:1 0/1:34,6,0:2 0/1:62,9,0:3 0/1:19,3,0:1 0/1:11,3,0:1 0/1:13,3,0:1 0/1:12,0,9:2

4 258180 . T A 999 . . GT:PL:DP 0/1:98,0,22:21 0/1:79,0,144:51 0/1:63,0,137:18 0/1:31,0,92:11 0/1:12,0,51:4 0/1:21,0,19:3 0/1:49,0,235:59

4 258836 . G A 999 . . GT:PL:DP 0/1:246,0,255:48 0/1:7,0,255:57 0/1:230,0,255:64 0/1:52,0,255:20 0/1:36,0,156:11 0/1:203,0,89:13 0/1:255,0,255:99

4 334421 . T C 999 . . GT:PL:DP 0/1:133,105,99:17 0/1:56,47,162:19 0/1:119,76,67:10 0/1:61,37,28:6 0/1:34,0,13:4 0/1:56,11,2:4 0/1:171,112,121:21

4 402406 . C T 999 . . GT:PL:DP 0/1:171,0,236:101 0/1:204,0,255:106 0/1:53,0,230:202 0/1:123,0,18:7 0/1:163,0,157:37 0/1:181,0,141:39 0/1:255,0,244:167

4 490116 . G C 999 . . GT:PL:DP 0/1:255,0,255:74 0/1:204,0,255:75 0/1:255,0,255:74 0/1:124,0,169:14 0/1:65,0,255:18 0/1:49,0,162:10 0/1:222,0,255:71

4 490117 . A G 999 . . GT:PL:DP 0/1:255,0,255:80 0/1:206,0,255:74 0/1:255,0,255:77 0/1:114,0,169:14 0/1:62,0,255:19 0/1:46,0,194:11 0/1:222,0,255:71

4 491800 . A C 999 . . GT:PL:DP 0/1:144,0,118:27 0/1:65,0,178:15 0/1:124,0,92:15 0/1:67,6,0:2 0/1:53,0,79:5 0/1:101,0,24:5 0/1:252,0,124:42

4 538061 . G A 999 . . GT:PL:DP 0/1:163,0,234:278 0/1:118,0,222:283 0/1:23,0,71:297 0/1:167,0,207:95 0/1:179,0,255:185 0/1:158,0,255:229 0/1:112,0,193:288

4 538358 . G A 999 . . GT:PL:DP 0/1:255,0,255:215 0/1:184,0,255:216 0/1:204,0,255:220 0/1:172,0,252:118 0/1:213,0,255:118 0/1:116,0,228:123 0/1:56,0,194:225

4 538570 . A G 999 . . GT:PL:DP 0/1:208,0,255:249 0/1:202,0,255:245 0/1:197,0,255:240 0/1:200,0,245:111 0/1:193,0,217:91 0/1:175,0,177:48 0/1:182,0,254:231

4 538854 . C G 999 . . GT:PL:DP 0/1:255,0,255:239 0/1:255,0,255:245 0/1:255,0,255:245 0/1:255,0,255:149 0/1:147,0,136:73 0/1:157,0,125:47 0/1:255,0,243:236

4 585552 . T C 999 . . GT:PL:DP 0/1:136,0,217:31 0/1:96,0,175:16 0/1:82,0,235:31 0/1:25,0,58:3 0/1:59,6,0:2 0/1:12,0,33:2 0/1:210,0,140:55

4 644967 . T G 999 . . GT:PL:DP 0/1:100,0,174:58 0/1:130,0,200:38 0/1:62,0,192:54 0/1:20,0,148:16 0/1:8,0,132:10 0/1:48,0,73:12 0/1:135,0,221:77

4 645101 . C T 999 . . GT:PL:DP 0/1:207,0,255:93 0/1:255,0,255:88 0/1:145,0,255:99 0/1:133,0,232:19 0/1:65,0,255:22 0/1:72,0,255:27 0/1:205,0,255:160

4 645333 . T A 999 . . GT:PL:DP 0/1:255,0,255:77 0/1:255,0,255:63 0/1:255,0,255:59 0/1:127,0,7:6 0/1:185,0,132:20 0/1:160,0,82:19 0/1:255,0,255:147

4 645358 . C T 999 . . GT:PL:DP 0/1:222,0,255:78 0/1:177,0,255:59 0/1:142,0,255:60 0/1:39,0,112:7 0/1:67,0,237:24 0/1:109,0,79:22 0/1:125,0,255:147

4 645365 . G A 999 . . GT:PL:DP 0/1:116,0,255:78 0/1:104,0,255:57 0/1:119,0,255:57 0/1:41,0,80:6 0/1:41,0,237:22 0/1:40,0,110:22 0/1:15,0,255:146

4 645373 . G A 999 . . GT:PL:DP 0/1:65,0,255:71 0/1:95,0,255:53 0/1:61,0,255:53 0/1:32,0,80:6 0/1:47,0,205:22 0/1:16,0,112:21 0/1:20,0,255:151

4 856859 . A T 999 . . GT:PL:DP 0/1:206,0,255:105 0/1:222,0,255:81 0/1:226,0,255:93 0/1:103,0,242:21 0/1:57,0,255:38 0/1:160,0,255:55 0/1:37,0,251:184

4 856963 . A T 999 . . GT:PL:DP 0/1:116,0,189:60 0/1:81,0,224:48 0/1:122,0,159:58 0/1:70,0,158:17 0/1:8,0,126:19 0/1:13,0,108:25 0/1:109,0,255:91

4 864321 . T C 999 . . GT:PL:DP 0/1:255,0,149:39 0/1:255,0,134:57 0/1:233,0,146:47 0/1:124,0,132:15 0/1:176,0,63:13 0/1:143,0,93:12 0/1:255,0,150:71

4 864380 . T C 999 . . GT:PL:DP 0/1:115,0,213:36 0/1:112,0,255:43 0/1:140,0,161:41 0/1:124,0,105:12 0/1:32,0,126:8 0/1:95,0,109:10 0/1:26,0,255:68

4 885746 . A G 999 . . GT:PL:DP 0/1:50,0,27:14 0/1:81,0,53:16 0/1:57,1,0:11 0/1:16,3,0:1 0/1:10,0,26:2 0/1:51,9,0:3 0/1:132,0,77:24

4 901675 . A T 999 . . GT:PL:DP 0/1:194,0,93:42 0/1:186,0,185:49 0/1:134,0,224:71 0/1:18,0,61:6 0/1:56,0,36:12 0/1:51,0,82:11 0/1:154,0,207:96

4 901685 . A G 999 . . GT:PL:DP 0/1:211,0,67:54 0/1:214,0,190:60 0/1:200,0,233:83 0/1:77,0,49:9 0/1:53,0,19:12 0/1:57,0,69:12 0/1:217,0,205:116

4 901961 . A T 999 . . GT:PL:DP 0/1:243,0,255:217 0/1:142,0,255:230 0/1:255,0,255:223 0/1:222,0,245:43 0/1:140,0,218:33 0/1:86,0,209:30 0/1:187,0,255:256

4 901976 . C T 999 . . GT:PL:DP 0/1:236,0,159:211 0/1:255,0,247:213 0/1:255,0,255:218 0/1:244,0,243:45 0/1:251,0,99:29 0/1:164,0,227:30 0/1:255,0,255:236

4 902104 . C T 999 . . GT:PL:DP 0/1:133,0,227:64 0/1:195,0,247:119 0/1:70,0,251:108 0/1:125,0,207:28 0/1:28,0,65:6 0/1:28,0,10:7 0/1:168,0,255:156

4 902110 . A G 999 . . GT:PL:DP 0/1:138,0,200:61 0/1:199,0,237:126 0/1:106,0,244:113 0/1:132,0,192:28 0/1:21,0,28:4 0/1:50,10,0:6 0/1:183,0,255:160

4 902139 . T C 999 . . GT:PL:DP 0/1:111,0,217:54 0/1:151,0,238:121 0/1:163,0,253:96 0/1:20,0,233:27 0/1:19,0,74:7 0/1:5,0,49:8 0/1:182,0,255:196

4 903150 . C T 999 . . GT:PL:DP 0/1:161,0,255:167 0/1:196,0,255:161 0/1:50,0,205:174 0/1:124,0,237:56 0/1:106,0,232:36 0/1:91,0,192:27 0/1:73,0,239:173

4 903446 . C A 999 . . GT:PL:DP 0/1:35,0,236:156 0/1:227,0,255:209 0/1:195,0,255:190 0/1:105,0,245:48 0/1:9,0,199:22 0/1:113,0,213:30 0/1:185,0,255:236

4 903538 . C A 999 . . GT:PL:DP 0/1:160,0,255:128 0/1:92,0,255:178 0/1:126,0,242:197 0/1:74,0,199:42 0/1:88,0,237:17 0/1:78,0,255:41 0/1:120,0,255:197

4 1432136 . G C 999 . . GT:PL:DP 0/1:253,0,197:61 0/1:234,0,255:115 0/1:97,0,231:51 0/1:37,0,116:11 0/1:156,0,69:43 0/1:139,0,108:54 0/1:240,0,196:120

4 1451530 . T C 999 . . GT:PL:DP 0/1:254,0,59:24 0/1:255,0,255:77 0/1:255,0,255:64 0/1:92,9,0:3 0/1:129,0,117:13 0/1:99,0,4:5 0/1:255,0,255:85

4 1454074 . A G 999 . . GT:PL:DP 0/1:192,0,250:32 0/1:255,0,83:51 0/1:255,0,129:53 0/1:21,0,73:4 0/1:76,0,22:4 0/1:155,0,18:10 0/1:255,0,219:71

4 1532104 . C T 999 . . GT:PL:DP 0/1:46,6,0:2 0/1:83,0,94:12 0/1:119,0,10:11 0/1:22,3,0:1 0/1:44,6,0:2 0/1:41,0,12:3 0/1:37,0,95:9

4 1532105 . T A 999 . . GT:PL:DP 0/1:46,6,0:2 0/1:83,0,94:12 0/1:119,0,10:11 0/1:22,3,0:1 0/1:44,6,0:2 0/1:41,0,11:3 0/1:34,0,101:10

4 1532107 . T A 999 . . GT:PL:DP 0/1:46,6,0:2 0/1:83,0,56:11 0/1:119,0,10:11 0/1:22,3,0:1 0/1:44,6,0:2 0/1:50,6,0:2 0/1:60,0,90:10

4 1581092 . T C 50.0291 . . GT:PL:DP 0/1:16,0,30:4 0/1:11,0,14:4 0/1:33,0,42:5 0/1:20,3,0:1 0/1:14,0,17:3 0/1:28,4,0:4 0/1:11,0,31:5

4 1674384 . A G 999 . . GT:PL:DP 0/1:114,0,210:119 0/1:158,0,229:111 0/1:95,0,182:128 0/1:71,0,113:16 0/1:24,5,0:4 0/1:23,0,63:12 0/1:61,0,255:208

4 1839566 . C T 999 . . GT:PL:DP 0/1:249,0,176:210 0/1:192,0,47:170 0/1:255,0,240:193 0/1:251,7,0:50 0/1:102,0,57:57 0/1:193,0,233:60 0/1:252,0,215:223

4 1855491 . G C 999 . . GT:PL:DP 0/1:40,0,1:3 0/1:73,0,39:10 0/1:34,0,75:6 0/1:88,0,37:6 0/1:31,3,0:1 0/1:30,3,0:1 0/1:168,0,91:18

4 1855496 . G C 999 . . GT:PL:DP 0/1:48,6,0:2 0/1:121,0,13:11 0/1:43,0,17:3 0/1:84,0,10:5 0/1:35,3,0:1 0/1:32,3,0:1 0/1:233,0,22:22

4 1864949 . A G 999 . . GT:PL:DP 0/1:136,0,35:64 0/1:115,0,138:85 0/1:96,0,84:56 0/1:39,0,33:13 0/1:8,0,100:14 0/1:20,0,94:30 0/1:127,0,101:110

4 1924808 . C A 999 . . GT:PL:DP 0/1:175,0,167:104 0/1:95,0,162:65 0/1:73,0,171:60 0/1:64,0,66:9 0/1:17,0,53:4 0/1:19,0,51:4 0/1:110,0,196:70

4 1924809 . C A 999 . . GT:PL:DP 0/1:187,0,183:106 0/1:92,0,161:65 0/1:82,0,171:60 0/1:64,0,69:9 0/1:17,0,53:4 0/1:19,0,51:4 0/1:115,0,197:69

4 2087447 . C A 999 . . GT:PL:DP 0/1:139,0,108:23 0/1:185,0,165:27 0/1:245,0,34:25 0/1:91,0,9:5 0/1:57,0,47:5 0/1:98,0,185:17 0/1:251,0,105:36

4 2087473 . A G 999 . . GT:PL:DP 0/1:107,0,82:22 0/1:48,0,217:22 0/1:191,0,20:23 0/1:68,0,12:4 0/1:46,0,19:4 0/1:115,0,123:12 0/1:168,0,98:33

4 2134332 . T G 999 . . GT:PL:DP 0/1:135,0,215:16 0/1:10,0,248:20 0/1:25,0,255:27 0/1:25,3,0:1 0/1:12,0,19:2 0/1:48,9,0:3 0/1:255,0,193:41

4 2142211 . T C 999 . . GT:PL:DP 0/1:95,1,0:7 0/1:34,0,37:9 0/1:41,0,56:15 0/1:37,0,12:3 0/1:18,0,22:5 0/1:27,0,7:7 0/1:93,0,28:14

4 2438093 . T C 999 . . GT:PL:DP 0/1:9,0,59:7 0/1:36,0,10:4 0/1:55,0,65:10 0/1:23,0,15:3 0/1:17,3,0:1 0/1:31,6,0:2 0/1:77,0,36:13

4 2438095 . C A 999 . . GT:PL:DP 0/1:9,0,59:7 0/1:36,0,10:4 0/1:52,0,82:11 0/1:23,0,15:3 0/1:17,3,0:1 0/1:31,6,0:2 0/1:81,0,40:13

4 2445965 . T A 999 . . GT:PL:DP 0/1:89,5,0:12 0/1:100,0,1:20 0/1:54,0,13:16 0/1:40,6,0:2 0/1:28,0,34:7 0/1:30,11,7:6 0/1:104,0,30:25

4 2485437 . C A 999 . . GT:PL:DP 0/1:244,0,229:86 0/1:127,0,116:48 0/1:188,0,245:80 0/1:86,0,100:12 0/1:15,0,50:19 0/1:10,0,88:22 0/1:56,0,255:143

4 2485634 . T A 999 . . GT:PL:DP 0/1:193,0,255:101 0/1:225,0,255:118 0/1:174,0,255:103 0/1:117,0,142:17 0/1:42,0,43:14 0/1:45,0,54:8 0/1:89,0,255:123

4 2493099 . C T 999 . . GT:PL:DP 0/1:80,0,163:53 0/1:132,0,153:97 0/1:104,0,150:118 0/1:74,0,115:17 0/1:24,0,12:3 0/1:14,3,0:10 0/1:128,0,127:69

4 2508517 . G A 999 . . GT:PL:DP 0/1:255,0,255:246 0/1:255,0,255:245 0/1:255,0,255:240 0/1:152,0,255:87 0/1:130,0,199:53 0/1:137,0,171:40 0/1:255,0,228:244

4 2605928 . A G 999 . . GT:PL:DP 0/1:222,0,227:172 0/1:255,0,221:169 0/1:255,0,255:189 0/1:214,0,75:46 0/1:140,0,110:68 0/1:136,0,38:89 0/1:231,0,211:277

4 2605934 . T G 999 . . GT:PL:DP 0/1:243,0,221:172 0/1:255,0,229:167 0/1:255,0,255:188 0/1:239,0,130:46 0/1:143,0,118:64 0/1:124,0,37:87 0/1:235,0,223:267

4 2605980 . C T 999 . . GT:PL:DP 0/1:186,0,75:124 0/1:161,0,186:132 0/1:190,0,180:132 0/1:134,0,99:41 0/1:67,0,60:50 0/1:52,0,37:63 0/1:85,0,119:130

4 2801444 . C T 167.582 . . GT:PL:DP 0/1:46,0,66:12 0/1:42,0,96:22 0/1:14,0,167:32 0/1:15,0,18:3 0/1:19,9,0:3 0/1:6,0,23:2 0/1:30,0,161:29

4 2823642 . T A 999 . . GT:PL:DP 0/1:255,0,255:256 0/1:255,0,255:256 0/1:255,0,255:223 0/1:255,0,160:34 0/1:255,0,255:90 0/1:255,0,255:137 0/1:255,0,255:260

4 2823754 . A G 999 . . GT:PL:DP 0/1:74,0,255:55 0/1:215,0,255:51 0/1:28,0,242:34 0/1:13,0,238:19 0/1:175,0,145:34 0/1:0,0,129:23 0/1:123,0,255:62

4 2823876 . C G 999 . . GT:PL:DP 0/1:94,0,255:50 0/1:87,0,172:33 0/1:117,0,242:33 0/1:14,0,104:7 0/1:11,0,229:21 0/1:52,0,183:17 0/1:120,0,255:40

4 2823950 . T C 999 . . GT:PL:DP 0/1:86,0,255:118 0/1:107,0,223:87 0/1:130,0,255:91 0/1:124,0,167:22 0/1:175,0,253:34 0/1:131,0,255:33 0/1:70,0,255:82

4 2824202 . G A 999 . . GT:PL:DP 0/1:207,0,255:65 0/1:117,0,255:48 0/1:176,0,233:52 0/1:131,0,30:9 0/1:22,0,152:14 0/1:146,0,37:13 0/1:171,0,255:112

4 2889820 . A T 999 . . GT:PL:DP 0/1:135,0,255:25 0/1:136,0,221:22 0/1:136,0,221:26 0/1:51,6,0:2 0/1:23,0,21:2 0/1:39,3,0:1 0/1:178,0,255:34

4 2908573 . T C 999 . . GT:PL:DP 0/1:251,0,255:32 0/1:255,0,227:37 0/1:255,0,147:25 0/1:93,0,134:10 0/1:59,0,110:7 0/1:110,9,0:3 0/1:255,0,255:56

4 3183150 . C G 999 . . GT:PL:DP 0/1:103,0,200:151 0/1:202,0,237:162 0/1:82,0,197:180 0/1:14,0,184:53 0/1:104,0,156:62 0/1:38,0,185:36 0/1:63,0,236:172

4 3280628 . T A 999 . . GT:PL:DP 0/1:197,0,110:87 0/1:237,0,155:102 0/1:255,0,158:96 0/1:142,0,159:26 0/1:70,0,51:24 0/1:23,0,63:19 0/1:253,0,118:174

4 3359911 . T G 999 . . GT:PL:DP 0/1:130,0,255:88 0/1:255,0,255:105 0/1:223,0,255:95 0/1:188,0,209:35 0/1:123,0,112:45 0/1:155,0,156:79 0/1:255,0,255:233

4 3363244 . T A 999 . . GT:PL:DP 0/1:201,0,255:227 0/1:69,0,255:224 0/1:94,0,255:236 0/1:235,0,255:157 0/1:99,0,249:208 0/1:155,0,255:191 0/1:131,0,255:220

4 3456511 . T G 999 . . GT:PL:DP 0/1:215,0,255:46 0/1:108,0,231:27 0/1:137,0,210:20 0/1:25,0,52:4 0/1:49,6,0:2 0/1:104,0,58:7 0/1:255,0,73:38

4 3594299 . T C 999 . . GT:PL:DP 0/1:255,0,195:128 0/1:255,0,70:187 0/1:255,0,80:155 0/1:255,0,132:48 0/1:249,0,162:25 0/1:255,0,35:30 0/1:255,0,255:183

4 3674003 . A G 999 . . GT:PL:DP 0/1:255,0,255:38 0/1:237,0,255:32 0/1:255,0,207:33 0/1:63,0,207:11 0/1:68,0,26:3 0/1:95,9,0:3 0/1:255,0,255:51

4 3855650 . T G 999 . . GT:PL:DP 0/1:58,0,80:12 0/1:58,0,98:11 0/1:129,0,32:13 0/1:22,0,37:4 0/1:63,6,0:2 0/1:26,3,0:1 0/1:153,0,59:15

4 4280753 . C T 999 . . GT:PL:DP 0/1:70,0,17:7 0/1:133,8,15:19 0/1:131,0,2:13 0/1:81,9,0:3 0/1:59,6,0:2 0/1:53,0,5:3 0/1:121,0,28:28

4 4444745 . A T 999 . . GT:PL:DP 0/1:25,3,0:1 0/1:73,6,0:2 0/1:92,9,0:3 0/1:37,3,0:1 0/1:35,3,0:1 0/1:64,6,0:2 0/1:40,3,0:1

4 4482061 . T C 999 . . GT:PL:DP 0/1:163,0,13:22 0/1:195,0,42:31 0/1:153,0,24:17 0/1:70,9,0:3 0/1:58,0,119:9 0/1:8,0,65:5 0/1:10,0,179:15

4 4535051 . C A 999 . . GT:PL:DP 0/1:156,0,121:27 0/1:47,0,206:27 0/1:80,0,167:24 0/1:45,9,0:3 0/1:61,9,0:3 0/1:20,3,0:1 0/1:104,0,211:44

4 4561273 . A C 999 . . GT:PL:DP 0/1:97,0,164:21 0/1:47,0,233:21 0/1:165,0,173:23 0/1:19,0,24:2 0/1:54,9,0:3 0/1:95,0,51:11 0/1:152,0,202:35

4 4576920 . A T 999 . . GT:PL:DP 0/1:255,0,255:223 0/1:255,0,255:226 0/1:255,0,255:224 0/1:224,0,255:39 0/1:255,0,255:60 0/1:255,0,213:71 0/1:255,0,255:207

4 4577210 . C T 999 . . GT:PL:DP 0/1:255,0,255:202 0/1:255,0,255:214 0/1:255,0,255:225 0/1:207,0,255:34 0/1:94,0,141:51 0/1:109,0,117:86 0/1:255,0,255:234

4 4577294 . A C 999 . . GT:PL:DP 0/1:179,0,255:193 0/1:255,0,255:210 0/1:255,0,255:230 0/1:31,0,254:31 0/1:22,0,94:5 0/1:130,0,60:12 0/1:175,0,255:223

4 4577307 . C A 999 . . GT:PL:DP 0/1:105,0,255:184 0/1:162,0,255:199 0/1:203,0,255:232 0/1:35,0,235:31 0/1:17,0,17:3 0/1:112,0,77:13 0/1:145,0,255:217

4 4591807 . C T 999 . . GT:PL:DP 0/1:100,0,96:25 0/1:46,0,188:31 0/1:133,0,102:22 0/1:34,0,33:4 0/1:39,3,0:4 0/1:8,0,31:4 0/1:148,0,100:32

4 4610407 . A C 999 . . GT:PL:DP 0/1:167,0,163:32 0/1:150,0,137:23 0/1:161,0,117:28 0/1:34,0,65:5 0/1:25,0,24:2 0/1:15,0,62:3 0/1:175,0,192:42

4 4611018 . G A 999 . . GT:PL:DP 0/1:165,0,213:27 0/1:238,0,190:28 0/1:187,0,255:29 0/1:10,0,114:5 0/1:27,0,56:4 0/1:87,0,103:9 0/1:129,0,255:44

4 4825535 . C T 999 . . GT:PL:DP 0/1:179,0,223:25 0/1:201,0,233:31 0/1:248,0,241:37 0/1:65,0,68:6 0/1:82,0,22:4 0/1:166,0,11:7 0/1:199,0,255:46

4 4828426 . T G 999 . . GT:PL:DP 0/1:133,14,91:47 0/1:121,0,107:31 0/1:113,21,91:32 0/1:52,2,24:7 0/1:11,0,64:9 0/1:69,0,23:7 0/1:72,0,147:79

4 4828429 . T G 999 . . GT:PL:DP 0/1:127,10,59:57 0/1:101,26,89:46 0/1:89,0,98:39 0/1:53,4,38:9 0/1:11,0,26:8 0/1:61,0,14:12 0/1:46,0,124:107

4 4828604 . A T 999 . . GT:PL:DP 0/1:212,0,255:190 0/1:86,0,255:177 0/1:182,0,255:159 0/1:189,0,255:55 0/1:7,0,159:24 0/1:61,0,205:47 0/1:91,0,254:172

4 4828629 . A G 999 . . GT:PL:DP 0/1:230,0,255:194 0/1:144,0,255:185 0/1:165,0,255:172 0/1:131,0,255:63 0/1:24,0,164:27 0/1:95,0,251:59 0/1:166,0,255:174

4 4845508 . G A 999 . . GT:PL:DP 0/1:255,0,229:40 0/1:243,0,255:43 0/1:216,0,255:32 0/1:26,0,113:6 0/1:64,0,96:7 0/1:68,6,0:2 0/1:255,0,255:51

4 4846012 . A G 999 . . GT:PL:DP 0/1:94,0,253:37 0/1:210,0,215:45 0/1:190,0,232:34 0/1:16,0,18:2 0/1:36,0,112:7 0/1:11,0,66:6 0/1:187,0,235:40

4 5133076 . C T 999 . . GT:PL:DP 0/1:255,0,255:38 0/1:255,0,255:35 0/1:255,0,255:37 0/1:65,0,87:6 0/1:75,0,23:4 0/1:91,0,82:6 0/1:255,0,255:59

4 5376083 . A T 999 . . GT:PL:DP 0/1:138,0,135:57 0/1:52,0,234:53 0/1:95,0,201:60 0/1:73,0,109:17 0/1:43,3,0:17 0/1:43,1,0:9 0/1:123,0,145:65

4 5376088 . G C 999 . . GT:PL:DP 0/1:113,0,105:55 0/1:71,0,240:49 0/1:116,0,182:57 0/1:92,0,79:15 0/1:39,3,0:16 0/1:50,7,0:9 0/1:122,0,128:60

4 5376099 . A T 999 . . GT:PL:DP 0/1:109,0,104:51 0/1:56,0,172:35 0/1:110,0,170:44 0/1:92,0,79:15 0/1:58,6,0:10 0/1:29,5,0:4 0/1:121,0,104:55

4 5418384 . G C 999 . . GT:PL:DP 0/1:108,0,250:74 0/1:4,0,255:58 0/1:65,0,255:98 0/1:12,0,117:9 0/1:53,0,124:30 0/1:63,0,131:22 0/1:124,0,255:98

4 5447212 . C G 999 . . GT:PL:DP 0/1:122,0,34:13 0/1:65,0,36:15 0/1:164,0,23:15 0/1:30,0,11:2 0/1:42,6,0:2 0/1:44,6,0:2 0/1:103,0,126:23

4 5447215 . T G 999 . . GT:PL:DP 0/1:122,0,34:13 0/1:79,0,45:15 0/1:133,0,27:12 0/1:30,0,11:2 0/1:42,6,0:2 0/1:44,6,0:2 0/1:50,0,135:21

4 5471396 . T C 999 . . GT:PL:DP 0/1:147,0,227:44 0/1:132,0,129:26 0/1:119,0,177:25 0/1:48,6,0:2 0/1:56,0,93:10 0/1:20,3,0:1 0/1:149,0,221:50

4 5478864 . G A 999 . . GT:PL:DP 0/1:219,0,255:28 0/1:197,0,255:31 0/1:197,0,255:34 0/1:31,0,31:2 0/1:56,0,60:4 0/1:31,0,33:2 0/1:255,0,255:51

4 5491593 . A T 999 . . GT:PL:DP 0/1:214,0,255:42 0/1:108,0,255:27 0/1:255,0,255:70 0/1:98,9,0:3 0/1:10,0,184:8 0/1:105,0,50:6 0/1:255,0,255:66

4 5543314 . A G 999 . . GT:PL:DP 0/1:180,0,255:198 0/1:186,0,255:171 0/1:242,0,255:202 0/1:122,0,152:30 0/1:229,0,179:51 0/1:233,0,147:69 0/1:170,0,255:204

4 5543368 . A G 999 . . GT:PL:DP 0/1:181,0,255:207 0/1:195,0,255:183 0/1:228,0,255:217 0/1:150,0,166:36 0/1:191,0,131:40 0/1:231,0,58:50 0/1:189,0,255:202

4 5615116 . A C 999 . . GT:PL:DP 0/1:255,0,255:42 0/1:255,0,255:44 0/1:212,0,255:33 0/1:107,0,107:10 0/1:55,0,81:5 0/1:43,0,89:6 0/1:255,0,255:50

4 5615715 . A G 999 . . GT:PL:DP 0/1:98,0,138:18 0/1:181,0,102:26 0/1:206,0,212:31 0/1:49,6,0:2 0/1:25,0,26:2 0/1:26,3,0:1 0/1:255,0,223:48

4 5616769 . C T 999 . . GT:PL:DP 0/1:248,0,238:36 0/1:224,0,255:27 0/1:190,0,255:34 0/1:84,0,25:4 0/1:24,0,34:2 0/1:66,0,30:5 0/1:255,0,255:54

4 5638330 . A T 999 . . GT:PL:DP 0/1:63,0,141:11 0/1:171,0,243:21 0/1:212,0,196:18 0/1:74,6,0:2 0/1:36,3,0:1 0/1:31,0,24:2 0/1:255,0,255:38

4 5641166 . T G 999 . . GT:PL:DP 0/1:255,0,255:45 0/1:243,0,255:36 0/1:245,0,255:36 0/1:87,0,20:5 0/1:79,0,78:7 0/1:37,3,0:1 0/1:255,0,255:46

4 5657522 . T C 999 . . GT:PL:DP 0/1:154,0,47:25 0/1:180,0,18:17 0/1:222,0,132:38 0/1:51,0,54:7 0/1:24,0,25:3 0/1:8,0,78:6 0/1:242,0,80:29

4 5698361 . A G 999 . . GT:PL:DP 0/1:243,0,241:26 0/1:155,0,255:38 0/1:255,0,255:34 0/1:50,0,28:3 0/1:17,0,74:3 0/1:86,0,94:7 0/1:214,0,255:34

4 5698404 . G C 999 . . GT:PL:DP 0/1:255,0,233:33 0/1:255,0,160:34 0/1:255,0,255:45 0/1:6,0,76:5 0/1:69,0,27:3 0/1:36,0,82:5 0/1:255,0,182:32

4 5698408 . G A 999 . . GT:PL:DP 0/1:227,0,255:33 0/1:151,0,255:35 0/1:251,0,255:41 0/1:55,0,47:5 0/1:25,0,68:3 0/1:83,0,56:6 0/1:156,0,255:28

4 5698409 . T A 999 . . GT:PL:DP 0/1:224,0,255:32 0/1:148,0,255:34 0/1:255,0,255:37 0/1:55,0,43:4 0/1:21,0,70:3 0/1:82,0,43:6 0/1:156,0,255:28

4 5748971 . G A 999 . . GT:PL:DP 0/1:247,0,236:35 0/1:237,0,236:30 0/1:214,0,255:41 0/1:122,0,19:6 0/1:73,0,123:9 0/1:118,0,56:7 0/1:255,0,244:38

4 5874626 . A C 999 . . GT:PL:DP 0/1:207,0,217:34 0/1:255,0,188:39 0/1:208,0,255:39 0/1:62,0,102:7 0/1:33,0,27:2 0/1:30,0,63:3 0/1:244,0,228:41

4 5875849 . C T 999 . . GT:PL:DP 0/1:126,0,182:21 0/1:35,0,252:19 0/1:67,0,255:18 0/1:18,0,83:6 0/1:58,0,19:5 0/1:47,0,127:12 0/1:140,0,255:43

4 5875850 . A T 999 . . GT:PL:DP 0/1:120,0,179:21 0/1:18,0,255:19 0/1:75,0,250:18 0/1:14,0,99:6 0/1:55,0,19:5 0/1:38,0,127:12 0/1:127,0,255:43

4 5876195 . C T 999 . . GT:PL:DP 0/1:103,0,209:31 0/1:161,0,143:27 0/1:124,0,203:28 0/1:74,0,86:8 0/1:31,0,52:5 0/1:17,0,37:3 0/1:186,0,218:51

4 5876236 . T G 999 . . GT:PL:DP 0/1:161,0,102:32 0/1:114,0,178:28 0/1:180,0,61:20 0/1:32,0,71:6 0/1:20,0,64:6 0/1:20,0,15:3 0/1:142,0,183:46

4 5876334 . T G 999 . . GT:PL:DP 0/1:199,0,156:30 0/1:171,0,178:32 0/1:187,0,124:26 0/1:33,0,124:8 0/1:65,9,0:3 0/1:77,0,41:6 0/1:171,0,178:40

4 5993146 . T A 999 . . GT:PL:DP 0/1:140,0,81:23 0/1:192,0,110:33 0/1:179,0,112:26 0/1:14,0,26:2 0/1:41,0,45:5 0/1:27,0,54:4 0/1:230,0,160:47

4 6004716 . T C 999 . . GT:PL:DP 0/1:255,0,255:209 0/1:255,0,255:206 0/1:255,0,238:208 0/1:255,0,255:193 0/1:239,0,251:52 0/1:136,0,255:47 0/1:255,0,255:199

4 6042595 . C A 999 . . GT:PL:DP 0/1:166,0,255:126 0/1:37,0,255:57 0/1:45,0,242:97 0/1:13,0,167:16 0/1:56,0,196:18 0/1:11,0,211:38 0/1:28,0,214:163

4 6042820 . C T 999 . . GT:PL:DP 0/1:66,52,108:20 0/1:124,29,40:28 0/1:102,0,1:27 0/1:22,0,26:3 0/1:9,3,0:2 0/1:18,3,0:2 0/1:146,13,9:24

4 6055758 . A G 999 . . GT:PL:DP 0/1:41,0,53:7 0/1:146,0,94:12 0/1:23,0,45:4 0/1:39,0,32:6 0/1:55,31,29:4 0/1:42,0,40:4 0/1:179,0,57:25

4 6109234 . T A 999 . . GT:PL:DP 0/1:138,0,156:39 0/1:99,0,146:40 0/1:102,0,167:79 0/1:17,0,108:6 0/1:31,0,33:2 0/1:37,3,0:1 0/1:134,0,129:33

4 6110206 . C T 999 . . GT:PL:DP 0/1:255,0,255:164 0/1:212,0,251:76 0/1:255,0,255:184 0/1:26,0,155:36 0/1:143,0,190:39 0/1:183,0,138:34 0/1:231,0,250:135

4 6110240 . C A 999 . . GT:PL:DP 0/1:255,0,201:106 0/1:181,0,100:43 0/1:255,0,255:127 0/1:51,0,68:20 0/1:90,0,71:14 0/1:106,0,33:13 0/1:159,0,124:90

4 6112219 . G A 999 . . GT:PL:DP 0/1:255,0,255:220 0/1:255,0,255:162 0/1:255,0,255:235 0/1:112,0,255:61 0/1:255,0,255:85 0/1:247,0,255:158 0/1:221,0,255:203

4 6113228 . A G 999 . . GT:PL:DP 0/1:255,0,205:149 0/1:175,0,192:88 0/1:255,0,255:156 0/1:108,0,88:13 0/1:121,0,168:17 0/1:213,0,166:25 0/1:96,0,255:112

4 6113280 . T G 999 . . GT:PL:DP 0/1:120,0,237:86 0/1:40,0,161:48 0/1:138,0,247:87 0/1:10,0,54:5 0/1:37,0,104:12 0/1:56,0,192:22 0/1:51,0,172:64

4 6113297 . T A 999 . . GT:PL:DP 0/1:134,0,191:62 0/1:111,0,131:31 0/1:141,0,223:61 0/1:20,0,27:4 0/1:52,0,113:12 0/1:93,0,154:21 0/1:86,0,179:47

4 6116281 . A G 999 . . GT:PL:DP 0/1:117,0,102:16 0/1:123,0,6:10 0/1:126,0,31:15 0/1:52,6,0:2 0/1:27,1,0:2 0/1:82,0,18:7 0/1:114,0,155:33

4 6116368 . C G 999 . . GT:PL:DP 0/1:115,0,164:26 0/1:133,0,98:23 0/1:157,0,139:27 0/1:60,0,47:6 0/1:54,0,86:10 0/1:72,0,119:10 0/1:199,0,213:51

4 6116407 . C T 999 . . GT:PL:DP 0/1:80,0,158:16 0/1:99,0,96:17 0/1:133,0,111:24 0/1:21,0,52:4 0/1:58,0,78:8 0/1:35,0,124:9 0/1:187,0,218:39

4 6117066 . T C 999 . . GT:PL:DP 0/1:255,0,71:29 0/1:253,0,135:26 0/1:255,0,214:31 0/1:99,0,57:8 0/1:146,0,13:9 0/1:149,0,102:13 0/1:255,0,204:60

4 6122907 . T C 999 . . GT:PL:DP 0/1:139,0,239:18 0/1:176,0,227:21 0/1:248,0,216:30 0/1:10,5,2:2 0/1:25,0,87:4 0/1:76,6,0:2 0/1:231,0,255:32

4 6125296 . G A 999 . . GT:PL:DP 0/1:71,0,66:10 0/1:20,0,219:36 0/1:77,0,190:34 0/1:81,0,12:6 0/1:24,0,8:2 0/1:55,6,0:2 0/1:96,0,204:47

4 6125456 . C A 999 . . GT:PL:DP 0/1:201,0,180:28 0/1:34,0,255:19 0/1:142,0,178:23 0/1:98,0,54:6 0/1:28,3,0:1 0/1:95,9,0:3 0/1:242,0,255:52

4 6135844 . A T 999 . . GT:PL:DP 0/1:234,0,197:31 0/1:255,0,5:31 0/1:200,0,110:19 0/1:118,0,30:6 0/1:49,0,105:10 0/1:108,0,72:10 0/1:255,0,152:33

4 6181100 . A G 999 . . GT:PL:DP 0/1:152,0,255:68 0/1:96,0,221:58 0/1:147,0,255:82 0/1:76,0,97:8 0/1:40,0,139:8 0/1:35,0,184:16 0/1:235,0,247:68

4 6181354 . T G 999 . . GT:PL:DP 0/1:255,0,255:57 0/1:255,0,255:82 0/1:166,0,255:86 0/1:97,0,213:17 0/1:33,0,255:26 0/1:55,0,199:17 0/1:220,0,255:139

4 6181355 . C T 999 . . GT:PL:DP 0/1:186,0,255:57 0/1:169,0,255:82 0/1:255,0,255:87 0/1:144,0,191:17 0/1:140,0,239:26 0/1:102,0,168:17 0/1:255,0,255:137

4 6181442 . C T 999 . . GT:PL:DP 0/1:191,0,255:70 0/1:248,0,255:98 0/1:121,0,255:94 0/1:63,0,205:15 0/1:38,0,165:9 0/1:120,0,202:15 0/1:255,0,255:162

4 6181446 . A T 999 . . GT:PL:DP 0/1:205,0,255:68 0/1:193,0,255:96 0/1:255,0,255:89 0/1:102,0,199:14 0/1:37,0,117:9 0/1:46,0,244:16 0/1:186,0,255:162

4 6181501 . T G 999 . . GT:PL:DP 0/1:124,0,255:66 0/1:196,0,255:83 0/1:106,0,255:97 0/1:70,0,248:17 0/1:144,0,190:16 0/1:34,0,177:13 0/1:255,0,255:156

4 6181508 . C T 999 . . GT:PL:DP 0/1:117,0,255:65 0/1:211,0,255:79 0/1:103,0,255:95 0/1:70,0,237:17 0/1:142,0,202:17 0/1:55,0,177:13 0/1:255,0,255:152

4 6181919 . G A 999 . . GT:PL:DP 0/1:255,0,209:62 0/1:33,0,255:42 0/1:255,0,255:67 0/1:176,0,143:15 0/1:96,0,193:17 0/1:113,14,74:11 0/1:255,0,255:114

4 6181948 . G C 999 . . GT:PL:DP 0/1:255,0,255:58 0/1:77,0,255:46 0/1:255,0,255:74 0/1:157,0,70:10 0/1:62,0,252:17 0/1:78,0,121:8 0/1:255,0,255:113

4 6181992 . T A 999 . . GT:PL:DP 0/1:255,0,244:46 0/1:56,0,255:52 0/1:255,0,255:75 0/1:123,0,43:7 0/1:41,0,164:8 0/1:122,0,55:7 0/1:255,0,255:115

4 6182013 . A G 999 . . GT:PL:DP 0/1:255,0,255:50 0/1:47,0,255:47 0/1:255,0,255:74 0/1:124,0,62:9 0/1:38,0,169:9 0/1:128,0,17:6 0/1:255,0,255:107

4 6182025 . G A 999 . . GT:PL:DP 0/1:255,0,255:48 0/1:56,0,255:43 0/1:255,0,255:74 0/1:75,0,73:7 0/1:44,0,133:7 0/1:134,0,17:6 0/1:255,0,255:109

4 6182052 . G T 999 . . GT:PL:DP 0/1:255,0,240:55 0/1:96,0,255:43 0/1:255,0,255:65 0/1:90,0,96:8 0/1:53,0,94:7 0/1:132,0,58:9 0/1:255,0,255:112

4 6182142 . A G 999 . . GT:PL:DP 0/1:215,0,255:58 0/1:181,0,138:24 0/1:189,0,255:65 0/1:104,0,202:17 0/1:91,0,78:12 0/1:44,0,128:11 0/1:255,0,255:105

4 6182160 . T G 999 . . GT:PL:DP 0/1:255,0,247:56 0/1:130,0,193:29 0/1:255,0,189:63 0/1:193,0,106:16 0/1:85,0,72:11 0/1:120,0,77:10 0/1:255,0,255:93

4 6182251 . T A 999 . . GT:PL:DP 0/1:255,0,192:49 0/1:214,0,193:35 0/1:255,0,168:56 0/1:85,0,79:7 0/1:161,0,52:11 0/1:9,0,90:6 0/1:255,0,255:66

4 6182252 . T A 999 . . GT:PL:DP 0/1:255,0,205:49 0/1:218,0,197:35 0/1:255,0,163:58 0/1:110,0,79:7 0/1:162,0,39:11 0/1:9,0,90:6 0/1:255,0,255:66

4 6402691 . G A 999 . . GT:PL:DP 0/1:34,0,115:20 0/1:54,0,125:38 0/1:56,0,128:33 0/1:9,0,101:7 0/1:60,0,56:8 0/1:24,0,81:9 0/1:80,0,89:16

4 6402698 . T C 999 . . GT:PL:DP 0/1:26,0,120:17 0/1:68,0,125:36 0/1:26,0,132:30 0/1:7,0,106:6 0/1:32,0,60:6 0/1:25,0,88:8 0/1:85,0,87:16

4 6457138 . T A 999 . . GT:PL:DP 0/1:188,0,235:24 0/1:147,0,174:18 0/1:244,0,255:55 0/1:82,9,0:3 0/1:43,0,47:4 0/1:94,0,85:7 0/1:255,0,255:47

4 6574796 . G A 999 . . GT:PL:DP 0/1:255,0,252:36 0/1:255,0,255:45 0/1:255,0,255:49 0/1:203,0,58:12 0/1:147,0,41:7 0/1:57,0,88:5 0/1:255,0,255:64

4 6803001 . C A 999 . . GT:PL:DP 0/1:255,0,88:51 0/1:255,0,59:52 0/1:255,0,49:26 0/1:68,0,26:5 0/1:58,0,0:3 0/1:61,40,66:6 0/1:255,0,116:70

4 6805135 . T G 999 . . GT:PL:DP 0/1:150,0,118:18 0/1:105,0,129:19 0/1:46,0,110:14 0/1:23,3,0:1 0/1:57,0,15:5 0/1:28,0,22:4 0/1:136,0,93:19

4 6805136 . C G 999 . . GT:PL:DP 0/1:143,0,99:17 0/1:90,0,136:18 0/1:46,0,110:14 0/1:23,3,0:1 0/1:57,0,15:5 0/1:28,0,22:4 0/1:137,0,98:18

4 6807241 . C G 999 . . GT:PL:DP 0/1:59,0,137:76 0/1:68,0,137:62 0/1:169,0,10:55 0/1:90,0,93:19 0/1:42,0,63:27 0/1:51,0,74:30 0/1:104,0,157:84

4 6929099 . C A 999 . . GT:PL:DP 0/1:46,0,148:16 0/1:120,0,133:29 0/1:135,0,186:39 0/1:27,3,0:1 0/1:30,0,34:4 0/1:48,0,2:5 0/1:191,0,135:34

4 7017536 . T C 999 . . GT:PL:DP 0/1:212,0,112:27 0/1:44,0,141:16 0/1:191,0,255:50 0/1:85,12,0:4 0/1:48,0,58:6 0/1:71,0,24:6 0/1:147,0,255:44

4 7028855 . T C 999 . . GT:PL:DP 0/1:255,0,255:34 0/1:255,0,255:50 0/1:255,0,255:50 0/1:90,0,33:5 0/1:104,0,118:8 0/1:64,0,25:3 0/1:255,0,255:48

4 7028901 . T C 999 . . GT:PL:DP 0/1:255,0,255:39 0/1:255,0,255:51 0/1:255,0,255:50 0/1:142,0,48:7 0/1:100,0,124:8 0/1:58,0,30:3 0/1:255,0,255:48

4 7250829 . T A 999 . . GT:PL:DP 0/1:155,0,23:24 0/1:80,0,31:14 0/1:106,0,25:14 0/1:62,9,0:3 0/1:7,0,19:6 0/1:7,0,0:3 0/1:115,0,65:28

4 7250834 . A T 999 . . GT:PL:DP 0/1:155,0,39:25 0/1:80,0,35:14 0/1:105,0,13:14 0/1:62,9,0:3 0/1:7,0,19:6 0/1:7,0,0:3 0/1:112,0,79:30

4 7250835 . T G 999 . . GT:PL:DP 0/1:155,0,5:25 0/1:80,0,2:14 0/1:106,0,13:14 0/1:45,0,47:6 0/1:11,1,0:5 0/1:10,3,0:3 0/1:108,0,48:32

4 7271198 . T A 999 . . GT:PL:DP 0/1:144,0,2:12 0/1:70,0,76:9 0/1:94,0,144:20 0/1:17,0,16:2 0/1:23,3,0:1 0/1:18,0,33:3 0/1:190,0,72:33

4 7295271 . T C 999 . . GT:PL:DP 0/1:255,0,230:142 0/1:232,0,229:137 0/1:226,0,150:127 0/1:133,0,179:27 0/1:88,0,220:44 0/1:135,0,69:32 0/1:208,0,206:149

4 7295275 . C G 999 . . GT:PL:DP 0/1:253,0,229:141 0/1:212,0,205:133 0/1:229,0,150:135 0/1:135,0,156:27 0/1:76,0,225:43 0/1:136,0,68:32 0/1:203,0,134:148

4 7295296 . C T 999 . . GT:PL:DP 0/1:230,0,182:168 0/1:203,0,214:189 0/1:190,0,90:182 0/1:71,0,175:29 0/1:55,0,157:38 0/1:102,0,22:31 0/1:195,0,208:168

4 7295901 . T C 999 . . GT:PL:DP 0/1:150,0,255:146 0/1:146,0,255:158 0/1:164,0,255:138 0/1:74,0,149:14 0/1:70,0,123:20 0/1:146,0,129:41 0/1:200,0,255:124

4 7296869 . T C 999 . . GT:PL:DP 0/1:177,0,72:34 0/1:173,0,196:38 0/1:102,0,200:40 0/1:54,0,27:6 0/1:29,2,0:6 0/1:42,0,24:14 0/1:195,0,204:53

4 7317141 . C G 999 . . GT:PL:DP 0/1:11,1,233:14 0/1:22,0,225:20 0/1:53,0,255:46 0/1:33,1,0:2 0/1:78,0,19:5 0/1:20,0,52:5 0/1:23,0,255:69

4 7323642 . G A 999 . . GT:PL:DP 0/1:175,0,168:153 0/1:197,0,101:162 0/1:151,0,212:160 0/1:170,0,91:41 0/1:124,0,66:16 0/1:73,0,13:11 0/1:217,0,255:160

4 7324226 . A G 999 . . GT:PL:DP 0/1:219,0,244:64 0/1:186,0,255:83 0/1:200,0,255:100 0/1:255,0,194:43 0/1:51,0,6:12 0/1:119,0,23:19 0/1:229,0,255:86

4 7324373 . G A 999 . . GT:PL:DP 0/1:255,0,255:232 0/1:210,0,255:248 0/1:233,0,255:217 0/1:255,0,173:104 0/1:142,0,126:59 0/1:133,0,168:43 0/1:230,0,255:213

4 7324411 . G C 999 . . GT:PL:DP 0/1:214,0,255:262 0/1:210,0,255:272 0/1:212,0,255:248 0/1:255,0,218:161 0/1:239,0,138:113 0/1:211,0,156:55 0/1:193,0,255:247

4 7371184 . G T 999 . . GT:PL:DP 0/1:47,0,176:24 0/1:106,0,145:27 0/1:118,0,141:29 0/1:28,0,35:4 0/1:40,0,38:5 0/1:35,0,14:3 0/1:74,0,166:27

4 7444669 . C T 999 . . GT:PL:DP 0/1:225,0,99:188 0/1:218,0,31:206 0/1:248,0,162:169 0/1:149,0,53:46 0/1:167,0,52:29 0/1:150,0,17:31 0/1:167,0,19:226

4 7445056 . G A 999 . . GT:PL:DP 0/1:116,0,255:67 0/1:172,0,222:58 0/1:83,0,255:73 0/1:125,0,112:24 0/1:85,0,50:13 0/1:69,0,74:14 0/1:99,0,227:58

4 7472508 . T C 999 . . GT:PL:DP 0/1:200,0,255:115 0/1:223,0,255:76 0/1:169,0,255:126 0/1:202,0,125:20 0/1:112,0,106:8 0/1:97,0,183:18 0/1:255,0,255:149

4 7522340 . C T 999 . . GT:PL:DP 0/1:213,0,245:23 0/1:255,0,224:33 0/1:255,0,255:40 0/1:126,0,148:13 0/1:32,0,97:7 0/1:90,0,67:6 0/1:255,0,255:53

4 7528217 . A C 999 . . GT:PL:DP 0/1:234,0,159:29 0/1:251,0,240:42 0/1:80,0,233:25 0/1:9,0,98:5 0/1:19,0,109:5 0/1:72,6,0:2 0/1:235,0,255:52

4 7601693 . G T 999 . . GT:PL:DP 0/1:41,0,240:31 0/1:138,0,193:41 0/1:161,0,161:51 0/1:68,0,118:11 0/1:24,0,51:5 0/1:21,0,92:5 0/1:211,0,255:67

4 7601695 . T A 999 . . GT:PL:DP 0/1:58,0,239:31 0/1:136,0,191:41 0/1:166,0,148:51 0/1:68,0,109:11 0/1:24,0,59:5 0/1:21,0,101:5 0/1:213,0,255:67

4 7646948 . G C 999 . . GT:PL:DP 0/1:229,0,255:49 0/1:157,0,245:31 0/1:111,0,224:23 0/1:66,9,0:3 0/1:35,3,0:1 0/1:66,9,0:3 0/1:212,0,227:47

4 7691655 . G A 999 . . GT:PL:DP 0/1:148,0,249:30 0/1:113,0,242:32 0/1:92,0,187:27 0/1:15,0,123:9 0/1:75,0,14:5 0/1:59,3,0:5 0/1:228,0,103:32

4 7704904 . G C 999 . . GT:PL:DP 0/1:134,0,233:159 0/1:151,0,211:105 0/1:202,0,192:126 0/1:124,0,138:29 0/1:11,0,62:13 0/1:49,3,0:13 0/1:104,0,255:147

4 7705225 . A G 999 . . GT:PL:DP 0/1:214,0,255:174 0/1:235,0,255:109 0/1:130,0,255:133 0/1:206,0,217:41 0/1:132,0,184:46 0/1:161,0,255:115 0/1:255,0,255:192

4 7841495 . G T 999 . . GT:PL:DP 0/1:255,0,203:24 0/1:177,0,255:26 0/1:255,0,255:45 0/1:77,0,156:9 0/1:52,0,46:4 0/1:59,0,139:9 0/1:255,0,255:64

4 7880648 . G A 999 . . GT:PL:DP 0/1:250,0,255:43 0/1:255,0,255:50 0/1:248,0,255:33 0/1:39,0,28:3 0/1:8,0,115:6 0/1:28,0,66:4 0/1:255,0,248:40

4 7931493 . T G 999 . . GT:PL:DP 0/1:188,0,255:216 0/1:202,0,255:186 0/1:251,0,255:186 0/1:70,0,117:23 0/1:28,0,49:38 0/1:78,0,98:53 0/1:177,0,236:237

4 7960892 . G T 999 . . GT:PL:DP 0/1:63,0,38:10 0/1:121,0,3:12 0/1:52,0,38:8 0/1:45,0,9:5 0/1:17,0,28:5 0/1:37,0,13:4 0/1:89,0,55:14

4 7960894 . C G 999 . . GT:PL:DP 0/1:63,0,38:10 0/1:121,0,3:12 0/1:52,0,38:8 0/1:45,0,15:5 0/1:17,0,28:5 0/1:37,0,13:4 0/1:89,0,55:14

4 7960897 . T C 999 . . GT:PL:DP 0/1:63,0,38:10 0/1:119,0,13:13 0/1:52,0,38:8 0/1:45,0,15:5 0/1:17,0,28:5 0/1:37,0,13:4 0/1:86,0,64:16

4 7975316 . A C 999 . . GT:PL:DP 0/1:19,0,69:4 0/1:158,0,59:11 0/1:38,0,66:7 0/1:64,6,0:2 0/1:28,3,0:1 0/1:18,1,0:2 0/1:15,0,152:18

4 8059568 . T G 999 . . GT:PL:DP 0/1:127,0,96:26 0/1:132,0,159:41 0/1:45,0,94:25 0/1:40,6,0:2 0/1:94,0,10:12 0/1:29,0,17:7 0/1:119,0,189:36

4 8120046 . A G 999 . . GT:PL:DP 0/1:130,0,255:55 0/1:226,0,255:49 0/1:255,0,255:78 0/1:84,0,157:10 0/1:135,0,95:9 0/1:95,0,233:16 0/1:42,0,255:85

4 8130197 . C A 999 . . GT:PL:DP 0/1:204,0,141:30 0/1:91,0,143:14 0/1:226,0,215:44 0/1:43,0,119:11 0/1:30,3,0:1 0/1:53,6,0:2 0/1:207,0,186:45

4 8198462 . G A 999 . . GT:PL:DP 0/1:255,0,139:110 0/1:157,0,213:187 0/1:148,0,126:104 0/1:34,0,80:21 0/1:75,0,128:26 0/1:37,0,68:23 0/1:255,0,255:167

4 8346125 . C G 999 . . GT:PL:DP 0/1:116,0,255:160 0/1:255,0,255:138 0/1:142,0,255:145 0/1:66,0,255:33 0/1:21,0,175:56 0/1:230,0,255:116 0/1:152,0,255:153

4 8358591 . G A 999 . . GT:PL:DP 0/1:41,0,142:188 0/1:17,0,136:205 0/1:84,0,189:193 0/1:56,0,203:134 0/1:29,0,204:113 0/1:40,0,207:93 0/1:21,0,133:196

4 8408526 . G A 999 . . GT:PL:DP 0/1:255,0,252:54 0/1:185,0,59:17 0/1:25,0,191:29 0/1:74,0,70:7 0/1:53,0,17:8 0/1:18,2,0:11 0/1:108,0,255:65

4 8508747 . G A 999 . . GT:PL:DP 0/1:255,0,173:31 0/1:255,0,225:32 0/1:183,0,255:31 0/1:159,0,45:8 0/1:51,0,2:3 0/1:109,0,87:8 0/1:255,0,255:46

4 8628994 . C T 999 . . GT:PL:DP 0/1:255,0,255:38 0/1:255,0,255:40 0/1:229,0,255:29 0/1:188,0,166:16 0/1:128,0,89:8 0/1:133,0,10:6 0/1:150,0,255:47

4 8637782 . T C 999 . . GT:PL:DP 0/1:255,0,250:35 0/1:248,0,255:42 0/1:255,0,255:45 0/1:29,0,29:3 0/1:65,0,26:3 0/1:20,0,117:5 0/1:255,0,255:60

4 8657002 . G C 999 . . GT:PL:DP 0/1:133,0,75:14 0/1:128,0,126:14 0/1:140,0,120:18 0/1:13,0,75:4 0/1:38,0,21:3 0/1:33,0,15:3 0/1:134,0,245:31

4 8743399 . T C 999 . . GT:PL:DP 0/1:106,0,255:203 0/1:255,0,255:196 0/1:255,0,255:205 0/1:242,0,255:40 0/1:163,0,255:38 0/1:250,0,255:68 0/1:255,0,255:218

4 8743429 . C T 999 . . GT:PL:DP 0/1:155,0,255:192 0/1:255,0,255:198 0/1:255,0,255:208 0/1:238,0,247:41 0/1:89,0,255:37 0/1:182,0,255:83 0/1:255,0,255:220

4 8743579 . C T 999 . . GT:PL:DP 0/1:153,0,160:153 0/1:162,0,139:127 0/1:183,0,170:152 0/1:129,0,13:20 0/1:62,0,120:29 0/1:58,0,62:73 0/1:186,0,132:198

4 8743633 . A G 999 . . GT:PL:DP 0/1:144,0,148:103 0/1:158,0,138:105 0/1:173,0,160:137 0/1:132,0,21:21 0/1:57,0,111:20 0/1:17,0,41:37 0/1:151,0,131:144

4 8826625 . G A 999 . . GT:PL:DP 0/1:255,0,255:216 0/1:255,0,255:207 0/1:255,0,254:212 0/1:255,0,255:60 0/1:255,0,243:45 0/1:255,0,255:184 0/1:255,0,255:212

4 8826712 . G A 999 . . GT:PL:DP 0/1:255,0,255:202 0/1:231,0,255:194 0/1:255,0,255:195 0/1:255,0,255:49 0/1:242,0,255:75 0/1:255,0,255:173 0/1:234,0,255:211

4 8826844 . T C 999 . . GT:PL:DP 0/1:255,0,254:171 0/1:241,0,63:197 0/1:255,0,109:185 0/1:149,0,10:38 0/1:255,0,169:62 0/1:255,0,85:138 0/1:255,0,255:179

4 8826857 . C T 999 . . GT:PL:DP 0/1:207,0,255:167 0/1:143,0,213:194 0/1:255,0,255:162 0/1:95,0,181:36 0/1:165,0,249:62 0/1:183,0,255:130 0/1:69,0,251:173

4 8844374 . A C 999 . . GT:PL:DP 0/1:193,0,255:105 0/1:183,0,255:102 0/1:255,0,255:106 0/1:155,0,127:24 0/1:44,0,117:27 0/1:53,0,95:27 0/1:255,0,255:118

4 8844388 . T C 999 . . GT:PL:DP 0/1:241,0,255:83 0/1:246,0,255:94 0/1:111,0,255:91 0/1:82,0,176:17 0/1:70,0,102:22 0/1:17,0,116:17 0/1:112,0,255:103

4 8844601 . A T 999 . . GT:PL:DP 0/1:108,0,255:73 0/1:50,0,255:55 0/1:141,0,255:80 0/1:58,0,42:6 0/1:18,0,24:5 0/1:29,0,25:2 0/1:25,0,160:24

4 8844678 . A G 999 . . GT:PL:DP 0/1:255,0,255:178 0/1:255,0,255:177 0/1:253,0,139:187 0/1:170,0,100:23 0/1:125,0,35:15 0/1:23,0,1:6 0/1:255,0,193:97

4 8844743 . C T 999 . . GT:PL:DP 0/1:255,0,255:190 0/1:255,0,255:208 0/1:255,0,216:168 0/1:229,0,84:26 0/1:82,0,71:15 0/1:42,4,0:13 0/1:255,0,255:142

4 8845129 . G A 999 . . GT:PL:DP 0/1:255,0,255:193 0/1:253,0,255:189 0/1:255,0,255:154 0/1:255,0,253:57 0/1:12,0,51:13 0/1:50,0,128:15 0/1:255,0,255:170

4 8845382 . C G 999 . . GT:PL:DP 0/1:43,0,62:54 0/1:51,0,127:61 0/1:15,0,82:47 0/1:15,0,26:8 0/1:6,0,36:4 0/1:10,0,10:5 0/1:63,0,87:88

4 8845399 . C T 999 . . GT:PL:DP 0/1:57,0,50:43 0/1:51,0,153:51 0/1:30,0,86:43 0/1:15,0,34:8 0/1:18,0,33:4 0/1:19,0,11:7 0/1:44,0,88:68

4 8845466 . T C 88.4289 . . GT:PL:DP 0/1:25,0,40:11 0/1:34,0,155:26 0/1:32,0,20:24 0/1:23,0,1:3 0/1:14,3,0:1 0/1:8,1,0:2 0/1:31,0,49:26

4 8846039 . G A 999 . . GT:PL:DP 0/1:255,0,173:76 0/1:255,0,142:70 0/1:255,0,255:53 0/1:167,0,13:8 0/1:6,0,63:10 0/1:69,0,104:13 0/1:255,0,225:92

4 8847009 . C T 999 . . GT:PL:DP 0/1:255,0,144:64 0/1:253,0,154:81 0/1:246,0,175:68 0/1:129,0,15:19 0/1:128,0,127:21 0/1:87,0,137:15 0/1:227,0,36:100

4 8879562 . T G 999 . . GT:PL:DP 0/1:123,0,93:16 0/1:42,0,128:9 0/1:73,0,147:17 0/1:15,3,0:1 0/1:26,0,25:3 0/1:28,0,20:3 0/1:76,0,146:23

4 8918314 . G T 999 . . GT:PL:DP 0/1:69,0,254:130 0/1:106,0,225:100 0/1:24,0,191:71 0/1:86,0,222:39 0/1:67,0,183:27 0/1:12,0,96:23 0/1:164,0,255:96

4 8919210 . C T 999 . . GT:PL:DP 0/1:115,0,133:44 0/1:74,0,171:56 0/1:81,0,151:68 0/1:48,0,63:7 0/1:31,6,0:2 0/1:38,0,60:8 0/1:150,0,236:78

4 8933551 . G A 999 . . GT:PL:DP 0/1:195,0,246:58 0/1:188,0,166:46 0/1:154,0,241:54 0/1:57,0,100:9 0/1:42,9,0:3 0/1:36,8,0:5 0/1:247,0,255:102

4 8941875 . C A 999 . . GT:PL:DP 0/1:168,0,189:87 0/1:142,0,221:91 0/1:151,0,206:86 0/1:17,0,164:21 0/1:114,0,1:13 0/1:110,0,12:9 0/1:175,0,255:177

4 9059687 . A G 999 . . GT:PL:DP 0/1:255,0,255:192 0/1:255,0,255:160 0/1:255,0,255:183 0/1:24,0,255:42 0/1:21,0,141:21 0/1:34,0,59:28 0/1:124,0,252:200

4 9059704 . T A 999 . . GT:PL:DP 0/1:255,0,255:206 0/1:255,0,255:182 0/1:255,0,255:194 0/1:123,0,255:45 0/1:121,0,95:25 0/1:107,0,134:29 0/1:255,0,255:202

4 9086527 . T A 999 . . GT:PL:DP 0/1:147,0,255:31 0/1:255,0,116:32 0/1:255,0,183:30 0/1:41,3,0:1 0/1:18,0,106:7 0/1:27,0,30:2 0/1:255,0,255:52

4 9228660 . C A 999 . . GT:PL:DP 0/1:255,0,179:39 0/1:255,0,255:39 0/1:164,0,255:21 0/1:79,0,77:6 0/1:57,0,22:3 0/1:99,0,90:8 0/1:255,0,255:46

4 9345838 . A G 999 . . GT:PL:DP 0/1:255,0,110:31 0/1:205,0,207:30 0/1:255,0,174:38 0/1:18,9,0:3 0/1:23,0,70:4 0/1:40,0,73:7 0/1:199,0,255:47

4 9500086 . T G 999 . . GT:PL:DP 0/1:242,0,255:233 0/1:236,0,255:244 0/1:86,0,255:225 0/1:97,0,247:19 0/1:16,0,219:30 0/1:98,0,255:47 0/1:255,0,255:234

4 9500512 . G A 999 . . GT:PL:DP 0/1:229,0,255:176 0/1:179,0,255:202 0/1:217,0,255:176 0/1:83,0,255:42 0/1:255,0,255:184 0/1:153,0,255:186 0/1:148,0,255:161

4 9525695 . T A 999 . . GT:PL:DP 0/1:158,4,0:21 0/1:70,0,2:20 0/1:122,0,44:33 0/1:45,0,20:3 0/1:21,1,0:2 0/1:62,0,38:7 0/1:126,0,20:23

4 9603616 . T C 999 . . GT:PL:DP 0/1:211,0,219:258 0/1:255,0,184:238 0/1:255,0,107:255 0/1:172,0,213:130 0/1:151,0,196:134 0/1:136,0,255:190 0/1:255,0,251:221

4 9603653 . C T 999 . . GT:PL:DP 0/1:88,0,30:200 0/1:110,0,86:202 0/1:81,0,51:215 0/1:81,0,63:126 0/1:95,0,54:131 0/1:255,0,255:177 0/1:244,0,217:179

4 9603704 . T C 999 . . GT:PL:DP 0/1:77,0,69:194 0/1:125,0,104:184 0/1:60,0,25:211 0/1:127,0,125:137 0/1:129,0,161:109 0/1:255,0,255:136 0/1:255,0,255:182

4 9603886 . C T 999 . . GT:PL:DP 0/1:255,0,225:194 0/1:223,0,98:184 0/1:241,0,188:208 0/1:255,0,255:195 0/1:255,0,255:183 0/1:255,0,190:161 0/1:255,0,186:185

4 9604313 . A G 999 . . GT:PL:DP 0/1:255,0,255:214 0/1:255,0,255:223 0/1:255,0,255:219 0/1:255,0,255:227 0/1:255,0,255:210 0/1:226,0,255:219 0/1:255,0,243:209

4 9604331 . C G 999 . . GT:PL:DP 0/1:255,0,196:205 0/1:255,0,221:222 0/1:255,0,215:220 0/1:255,0,239:225 0/1:255,0,200:196 0/1:253,0,243:207 0/1:255,0,192:204

4 9604334 . C G 999 . . GT:PL:DP 0/1:255,0,209:211 0/1:255,0,255:220 0/1:255,0,255:220 0/1:255,0,255:223 0/1:255,0,249:203 0/1:255,0,255:209 0/1:255,0,215:205

4 9731361 . A G 999 . . GT:PL:DP 0/1:41,6,0:2 0/1:21,3,0:1 0/1:77,0,160:14 0/1:12,0,56:4 0/1:26,6,0:2 0/1:16,3,0:1 0/1:32,0,77:8

4 9770657 . C T 999 . . GT:PL:DP 0/1:51,0,81:21 0/1:92,0,37:27 0/1:116,0,110:34 0/1:36,9,0:3 0/1:48,0,20:5 0/1:6,0,6:2 0/1:76,0,162:56

4 9779852 . T C 999 . . GT:PL:DP 0/1:118,0,255:39 0/1:93,0,255:51 0/1:41,0,255:41 0/1:38,0,29:4 0/1:18,0,94:23 0/1:55,0,16:7 0/1:115,0,255:116

4 9779862 . G A 999 . . GT:PL:DP 0/1:117,0,255:39 0/1:90,0,255:51 0/1:67,0,255:41 0/1:38,0,29:4 0/1:17,0,95:21 0/1:56,0,22:6 0/1:58,0,255:117

4 9779868 . T C 999 . . GT:PL:DP 0/1:106,0,255:38 0/1:96,0,255:51 0/1:41,0,255:41 0/1:49,0,29:4 0/1:10,0,77:21 0/1:51,0,46:7 0/1:53,0,255:121

4 9864846 . T C 999 . . GT:PL:DP 0/1:255,0,240:122 0/1:255,0,214:113 0/1:191,0,255:95 0/1:107,0,169:21 0/1:19,3,0:22 0/1:63,0,74:83 0/1:255,0,255:127

4 9869424 . G A 999 . . GT:PL:DP 0/1:255,0,236:77 0/1:255,0,255:49 0/1:255,0,255:81 0/1:128,0,38:9 0/1:25,0,88:13 0/1:24,0,72:7 0/1:255,0,167:144

4 9869425 . G T 999 . . GT:PL:DP 0/1:255,0,241:78 0/1:255,0,255:49 0/1:255,0,255:81 0/1:128,0,32:9 0/1:25,0,87:13 0/1:24,0,72:7 0/1:255,0,175:142

4 10025888 . C T 999 . . GT:PL:DP 0/1:139,0,255:120 0/1:231,0,255:82 0/1:143,0,255:131 0/1:34,0,179:13 0/1:67,0,180:16 0/1:11,0,255:34 0/1:226,0,255:180

4 10215205 . T G 999 . . GT:PL:DP 0/1:62,0,22:18 0/1:100,0,0:10 0/1:166,8,0:14 0/1:24,0,12:3 0/1:43,6,0:3 0/1:88,9,0:3 0/1:172,0,2:22

4 10262340 . C T 999 . . GT:PL:DP 0/1:85,0,248:40 0/1:41,0,211:39 0/1:11,0,236:25 0/1:9,0,34:7 0/1:112,0,30:18 0/1:189,0,185:64 0/1:84,0,255:169

4 10262415 . C T 999 . . GT:PL:DP 0/1:91,0,178:29 0/1:39,0,138:23 0/1:36,0,115:12 0/1:16,3,0:1 0/1:50,0,14:16 0/1:117,0,115:38 0/1:132,0,255:73

4 10270871 . G A 999 . . GT:PL:DP 0/1:225,0,165:25 0/1:139,0,224:22 0/1:193,0,232:36 0/1:37,3,0:1 0/1:80,9,0:3 0/1:67,0,42:5 0/1:255,0,138:32

4 10397458 . T C 999 . . GT:PL:DP 0/1:73,0,86:13 0/1:97,0,23:25 0/1:110,2,0:20 0/1:13,0,13:2 0/1:20,0,52:4 0/1:21,3,0:1 0/1:56,0,11:13

4 10576930 . A C 999 . . GT:PL:DP 0/1:111,0,27:16 0/1:43,0,11:3 0/1:10,3,0:1 0/1:43,6,0:2 0/1:29,3,0:3 0/1:30,5,0:4 0/1:80,0,24:11

4 10576933 . C A 999 . . GT:PL:DP 0/1:111,0,30:16 0/1:43,0,9:3 0/1:10,3,0:1 0/1:38,6,0:2 0/1:36,9,0:3 0/1:36,9,0:3 0/1:35,0,37:9

4 10634435 . A T 999 . . GT:PL:DP 0/1:110,0,110:59 0/1:60,0,255:58 0/1:128,0,219:86 0/1:47,0,16:9 0/1:69,0,159:38 0/1:21,0,48:9 0/1:149,0,255:123

4 10634436 . C T 999 . . GT:PL:DP 0/1:107,0,120:59 0/1:64,0,255:56 0/1:133,0,217:83 0/1:51,0,21:7 0/1:68,0,161:38 0/1:24,0,50:9 0/1:153,0,255:121

4 10663227 . A T 999 . . GT:PL:DP 0/1:72,0,54:9 0/1:69,0,33:10 0/1:94,0,18:14 0/1:31,6,0:2 0/1:74,8,0:5 0/1:21,0,36:9 0/1:69,0,60:11

4 10704462 . T C 999 . . GT:PL:DP 0/1:100,0,60:42 0/1:100,0,105:20 0/1:195,0,22:31 0/1:39,0,24:5 0/1:43,2,0:4 0/1:71,0,14:13 0/1:168,0,103:72

4 10704476 . C A 999 . . GT:PL:DP 0/1:70,0,56:41 0/1:93,0,107:19 0/1:167,0,25:30 0/1:39,0,24:5 0/1:49,5,0:4 0/1:34,0,7:16 0/1:142,0,96:76

4 10704507 . G A 999 . . GT:PL:DP 0/1:56,0,95:43 0/1:91,0,107:20 0/1:127,0,18:27 0/1:39,0,24:5 0/1:25,5,0:4 0/1:29,0,17:17 0/1:107,0,117:75

4 10704510 . T C 999 . . GT:PL:DP 0/1:56,0,86:42 0/1:79,0,110:21 0/1:133,0,18:27 0/1:39,0,32:5 0/1:25,5,0:4 0/1:29,0,18:17 0/1:101,0,116:75

4 10704514 . T A 999 . . GT:PL:DP 0/1:93,0,82:40 0/1:73,0,103:23 0/1:121,0,27:28 0/1:41,0,28:4 0/1:23,5,0:4 0/1:41,0,8:16 0/1:109,0,114:77

4 10750572 . G A 999 . . GT:PL:DP 0/1:179,0,255:94 0/1:250,0,255:157 0/1:255,0,255:102 0/1:132,0,79:14 0/1:166,0,121:26 0/1:194,0,189:40 0/1:255,0,149:207

4 10750887 . G T 999 . . GT:PL:DP 0/1:255,0,255:132 0/1:232,0,255:185 0/1:255,0,255:127 0/1:223,0,160:23 0/1:110,0,196:22 0/1:157,0,255:31 0/1:255,0,255:228

4 10751259 . C T 999 . . GT:PL:DP 0/1:255,0,143:52 0/1:255,0,72:86 0/1:255,0,23:50 0/1:123,0,30:9 0/1:98,1,0:7 0/1:62,0,73:15 0/1:255,0,41:93

4 10755188 . G A 999 . . GT:PL:DP 0/1:64,0,247:16 0/1:172,0,215:27 0/1:233,0,112:20 0/1:86,0,73:6 0/1:37,3,0:1 0/1:129,12,0:4 0/1:109,0,255:29

4 10775774 . A C 999 . . GT:PL:DP 0/1:154,0,248:27 0/1:198,0,123:20 0/1:120,0,223:16 0/1:95,9,0:3 0/1:53,0,96:6 0/1:35,3,0:1 0/1:241,0,255:38

4 10827799 . C A 999 . . GT:PL:DP 0/1:97,0,255:25 0/1:199,0,255:46 0/1:178,0,255:46 0/1:51,0,219:13 0/1:11,0,22:2 0/1:24,0,159:9 0/1:246,0,255:43

4 10827813 . C T 999 . . GT:PL:DP 0/1:158,0,255:21 0/1:255,0,255:48 0/1:255,0,255:52 0/1:192,0,70:11 0/1:22,0,11:2 0/1:70,0,75:8 0/1:255,0,255:46

4 10827823 . G A 999 . . GT:PL:DP 0/1:255,0,207:26 0/1:255,0,251:44 0/1:255,0,255:52 0/1:181,0,46:9 0/1:28,3,0:1 0/1:113,0,40:8 0/1:255,0,255:50

4 10828078 . G A 999 . . GT:PL:DP 0/1:255,0,196:46 0/1:255,0,255:69 0/1:255,0,255:49 0/1:182,0,43:9 0/1:185,0,161:17 0/1:67,0,206:13 0/1:255,0,255:88

4 10828333 . T C 999 . . GT:PL:DP 0/1:199,0,255:56 0/1:255,0,255:49 0/1:133,0,255:46 0/1:59,0,157:9 0/1:175,0,72:14 0/1:133,0,70:9 0/1:187,0,255:76

4 10828366 . A T 999 . . GT:PL:DP 0/1:165,0,255:53 0/1:205,0,255:54 0/1:24,0,255:44 0/1:53,0,182:11 0/1:202,0,163:21 0/1:183,0,39:11 0/1:23,0,255:77

4 10828453 . G A 999 . . GT:PL:DP 0/1:14,0,255:43 0/1:134,0,255:48 0/1:78,0,255:54 0/1:39,0,177:11 0/1:80,0,216:13 0/1:95,0,179:12 0/1:145,0,255:67

4 10875098 . C T 146.055 . . GT:PL:DP 0/1:53,9,0:3 0/1:56,6,0:2 0/1:42,6,0:2 0/1:17,3,0:1 0/1:10,0,19:2 0/1:7,0,17:2 0/1:44,9,0:3

4 11023785 . T C 999 . . GT:PL:DP 0/1:78,0,82:10 0/1:12,0,199:16 0/1:111,0,20:18 0/1:90,9,0:3 0/1:22,0,84:4 0/1:20,0,64:4 0/1:163,0,113:35

4 11098378 . G A 999 . . GT:PL:DP 0/1:156,0,42:28 0/1:177,0,111:68 0/1:173,0,139:47 0/1:72,0,30:7 0/1:85,0,13:8 0/1:74,0,23:6 0/1:123,0,183:53

4 11148471 . T C 999 . . GT:PL:DP 0/1:255,0,255:93 0/1:255,0,255:78 0/1:255,0,255:107 0/1:154,0,197:23 0/1:159,0,58:17 0/1:139,0,181:16 0/1:32,0,255:66

4 11148505 . A G 999 . . GT:PL:DP 0/1:255,0,255:110 0/1:255,0,255:86 0/1:255,0,255:114 0/1:173,0,226:25 0/1:182,0,59:16 0/1:219,0,185:22 0/1:101,0,255:80

4 11148562 . A G 999 . . GT:PL:DP 0/1:67,0,255:123 0/1:74,0,255:96 0/1:191,0,255:111 0/1:24,0,255:26 0/1:13,0,255:14 0/1:147,0,255:32 0/1:171,0,255:117

4 11148613 . A T 999 . . GT:PL:DP 0/1:255,0,255:100 0/1:148,0,255:77 0/1:220,0,255:75 0/1:71,0,228:16 0/1:16,0,199:12 0/1:211,0,255:43 0/1:255,0,255:97

4 11162580 . C A 999 . . GT:PL:DP 0/1:190,0,137:33 0/1:147,0,17:16 0/1:208,0,74:33 0/1:46,0,13:4 0/1:37,3,0:3 0/1:27,0,112:8 0/1:179,0,223:57

4 11224665 . C T 999 . . GT:PL:DP 0/1:216,0,226:56 0/1:255,0,76:32 0/1:173,0,171:24 0/1:68,0,47:11 0/1:57,6,0:2 0/1:91,0,23:7 0/1:255,0,45:92

4 11250162 . C G 999 . . GT:PL:DP 0/1:183,0,96:28 0/1:99,0,107:14 0/1:151,0,100:20 0/1:39,0,24:3 0/1:20,3,0:1 0/1:18,3,0:1 0/1:98,0,138:26

4 11256784 . G A 999 . . GT:PL:DP 0/1:255,0,221:42 0/1:255,0,255:47 0/1:255,0,123:32 0/1:77,0,77:6 0/1:56,0,66:4 0/1:62,0,24:3 0/1:255,0,255:60

4 11256908 . A C 999 . . GT:PL:DP 0/1:169,0,255:45 0/1:208,0,252:31 0/1:249,0,152:37 0/1:47,0,54:5 0/1:44,0,27:3 0/1:19,0,23:2 0/1:228,0,249:46

4 11366206 . A C 999 . . GT:PL:DP 0/1:138,0,83:21 0/1:105,0,93:23 0/1:171,0,117:31 0/1:7,0,3:2 0/1:28,3,0:4 0/1:41,0,13:4 0/1:141,0,112:20

4 11366208 . T G 999 . . GT:PL:DP 0/1:134,0,131:20 0/1:92,0,110:24 0/1:177,0,122:25 0/1:13,3,0:1 0/1:28,3,0:4 0/1:41,0,13:4 0/1:107,0,148:21

4 11366210 . T C 999 . . GT:PL:DP 0/1:121,0,134:19 0/1:98,0,110:24 0/1:177,0,128:25 0/1:13,3,0:1 0/1:28,3,0:4 0/1:13,0,15:3 0/1:120,0,148:21

4 11366211 . A G 999 . . GT:PL:DP 0/1:88,0,134:17 0/1:50,0,111:21 0/1:177,0,128:25 0/1:13,3,0:1 0/1:28,3,0:4 0/1:13,0,15:3 0/1:112,0,148:21

4 11366213 . A G 999 . . GT:PL:DP 0/1:70,0,132:16 0/1:50,0,102:21 0/1:139,0,131:23 0/1:13,3,0:1 0/1:28,3,0:4 0/1:13,0,15:3 0/1:86,0,163:22

4 11366215 . T G 999 . . GT:PL:DP 0/1:71,0,131:16 0/1:50,0,111:21 0/1:29,0,164:22 0/1:13,3,0:1 0/1:28,3,0:4 0/1:13,0,15:3 0/1:47,0,167:21

4 11366219 . A T 89.288 . . GT:PL:DP 0/1:25,0,155:17 0/1:55,0,103:19 0/1:45,0,133:20 0/1:13,3,0:1 0/1:9,0,2:2 0/1:13,0,15:3 0/1:20,0,179:20

4 11366221 . T G 71.6144 . . GT:PL:DP 0/1:41,0,158:18 0/1:43,0,113:19 0/1:39,0,153:21 0/1:13,3,0:1 0/1:9,0,2:2 0/1:13,0,15:3 0/1:21,0,173:20

4 11542447 . G C 999 . . GT:PL:DP 0/1:51,0,143:19 0/1:45,0,85:12 0/1:130,0,50:22 0/1:36,6,0:2 0/1:26,0,72:5 0/1:11,0,27:2 0/1:188,0,42:21

4 11561823 . A C 999 . . GT:PL:DP 0/1:255,0,255:132 0/1:255,0,255:106 0/1:255,0,255:179 0/1:255,0,162:30 0/1:235,0,156:24 0/1:255,0,246:45 0/1:41,0,219:178

4 11576645 . A G 999 . . GT:PL:DP 0/1:155,0,153:32 0/1:82,0,255:36 0/1:136,0,120:18 0/1:6,0,50:4 0/1:105,0,95:9 0/1:83,0,102:7 0/1:159,0,244:34

4 11576659 . T G 999 . . GT:PL:DP 0/1:110,0,170:28 0/1:132,0,225:37 0/1:69,0,156:18 0/1:13,0,50:4 0/1:73,0,106:8 0/1:57,0,85:6 0/1:159,0,171:28

4 11608680 . C T 999 . . GT:PL:DP 0/1:243,0,255:203 0/1:160,0,255:197 0/1:240,0,255:199 0/1:66,0,255:63 0/1:216,0,215:58 0/1:122,0,255:77 0/1:236,0,255:205

4 11608886 . C A 999 . . GT:PL:DP 0/1:255,0,255:220 0/1:255,0,255:220 0/1:255,0,255:231 0/1:231,0,255:73 0/1:173,0,145:48 0/1:36,0,221:56 0/1:255,0,255:202

4 11608944 . G A 999 . . GT:PL:DP 0/1:255,0,255:227 0/1:255,0,255:221 0/1:255,0,255:228 0/1:255,0,255:77 0/1:190,0,173:53 0/1:95,0,255:89 0/1:255,0,255:217

4 11625754 . A G 999 . . GT:PL:DP 0/1:52,0,179:12 0/1:32,0,175:18 0/1:35,0,186:16 0/1:37,3,0:1 0/1:52,6,0:2 0/1:7,0,55:3 0/1:132,0,11:9

4 11701503 . C A 999 . . GT:PL:DP 0/1:72,0,73:17 0/1:103,0,62:18 0/1:78,0,44:10 0/1:13,0,12:2 0/1:13,3,0:1 0/1:13,0,2:2 0/1:90,0,76:17

4 11701504 . T A 999 . . GT:PL:DP 0/1:77,0,44:17 0/1:108,0,36:18 0/1:78,0,20:10 0/1:13,0,6:2 0/1:13,3,0:1 0/1:13,0,9:2 0/1:96,0,57:18

4 11763064 . G A 999 . . GT:PL:DP 0/1:119,0,146:30 0/1:83,0,141:32 0/1:18,0,163:37 0/1:31,0,36:10 0/1:52,2,0:8 0/1:50,0,17:18 0/1:36,0,144:49

4 11790213 . C T 999 . . GT:PL:DP 0/1:203,0,255:63 0/1:227,0,240:102 0/1:218,0,191:64 0/1:21,0,191:10 0/1:85,0,11:6 0/1:109,0,73:15 0/1:37,0,229:114

4 12080634 . C T 999 . . GT:PL:DP 0/1:146,0,70:17 0/1:147,0,90:18 0/1:5,0,64:5 0/1:34,0,8:5 0/1:64,6,0:2 0/1:61,9,0:3 0/1:157,0,112:27

4 12080639 . C A 999 . . GT:PL:DP 0/1:104,0,76:15 0/1:48,0,110:9 0/1:16,0,38:5 0/1:22,0,17:2 0/1:64,6,0:2 0/1:34,3,0:1 0/1:115,0,130:18

4 12081391 . T G 999 . . GT:PL:DP 0/1:106,0,212:20 0/1:25,0,235:15 0/1:75,0,164:18 0/1:55,0,124:8 0/1:26,0,10:2 0/1:10,0,28:3 0/1:97,0,255:26

4 12081507 . G A 999 . . GT:PL:DP 0/1:180,0,143:29 0/1:73,0,255:35 0/1:150,0,182:30 0/1:53,0,72:7 0/1:24,3,0:1 0/1:23,3,0:1 0/1:138,0,255:38

4 12081558 . T A 999 . . GT:PL:DP 0/1:246,0,178:33 0/1:156,0,255:40 0/1:139,0,255:29 0/1:83,0,73:7 0/1:31,3,0:1 0/1:58,0,23:4 0/1:255,0,255:46

4 12094040 . T C 999 . . GT:PL:DP 0/1:95,0,149:25 0/1:125,0,139:28 0/1:197,0,200:31 0/1:30,0,53:5 0/1:19,0,89:6 0/1:54,9,0:3 0/1:91,0,255:44

4 12185400 . G T 999 . . GT:PL:DP 0/1:142,0,122:15 0/1:184,0,125:15 0/1:145,0,200:24 0/1:83,9,0:3 0/1:141,0,13:6 0/1:66,6,0:2 0/1:255,0,253:39

4 12220792 . A G 999 . . GT:PL:DP 0/1:168,0,46:21 0/1:11,0,88:5 0/1:162,0,119:26 0/1:33,6,0:2 0/1:55,28,19:4 0/1:28,3,0:1 0/1:146,0,0:26

4 12258882 . A G 999 . . GT:PL:DP 0/1:217,0,255:36 0/1:221,0,202:23 0/1:255,0,255:36 0/1:91,0,109:9 0/1:74,0,28:4 0/1:42,0,115:7 0/1:254,0,255:45

4 12320302 . A T 999 . . GT:PL:DP 0/1:48,0,215:54 0/1:72,0,255:53 0/1:21,0,184:60 0/1:44,0,73:13 0/1:11,0,89:8 0/1:10,0,26:7 0/1:74,0,255:109

4 12320793 . A G 999 . . GT:PL:DP 0/1:255,0,105:71 0/1:209,0,201:54 0/1:255,0,153:66 0/1:19,0,57:5 0/1:11,0,17:10 0/1:59,0,37:6 0/1:253,0,201:114

4 12320824 . T G 999 . . GT:PL:DP 0/1:206,0,255:61 0/1:151,0,188:54 0/1:238,0,176:51 0/1:16,0,80:6 0/1:15,0,35:6 0/1:128,0,69:16 0/1:168,0,170:96

4 12340808 . T C 999 . . GT:PL:DP 0/1:255,0,255:40 0/1:255,0,255:110 0/1:255,0,255:85 0/1:185,0,206:19 0/1:49,0,169:9 0/1:115,0,255:20 0/1:255,0,255:87

4 12341074 . T A 999 . . GT:PL:DP 0/1:255,0,132:27 0/1:255,0,255:126 0/1:255,0,255:84 0/1:112,0,226:20 0/1:46,0,126:8 0/1:153,0,255:24 0/1:208,0,255:157

4 12341082 . G A 999 . . GT:PL:DP 0/1:155,0,255:28 0/1:255,0,255:127 0/1:118,0,255:85 0/1:226,0,146:19 0/1:87,0,80:8 0/1:96,0,255:24 0/1:252,0,255:152

4 12341097 . T C 999 . . GT:PL:DP 0/1:166,0,255:32 0/1:255,0,255:120 0/1:137,0,255:81 0/1:214,0,170:21 0/1:73,0,79:7 0/1:90,0,255:21 0/1:255,0,255:148

4 12404857 . C T 999 . . GT:PL:DP 0/1:19,0,255:72 0/1:146,0,255:58 0/1:235,0,255:156 0/1:37,0,213:12 0/1:82,0,255:35 0/1:216,0,255:29 0/1:47,0,255:174

4 12404912 . C T 999 . . GT:PL:DP 0/1:170,0,255:69 0/1:189,0,255:51 0/1:211,0,255:146 0/1:70,0,231:17 0/1:153,0,255:31 0/1:203,0,241:26 0/1:136,0,255:150

4 12405631 . A G 999 . . GT:PL:DP 0/1:255,0,255:183 0/1:255,0,255:194 0/1:255,0,255:200 0/1:217,0,198:35 0/1:192,0,181:31 0/1:255,0,255:65 0/1:223,0,255:193

4 12497038 . C G 999 . . GT:PL:DP 0/1:155,0,197:29 0/1:188,0,156:30 0/1:188,0,172:35 0/1:52,6,0:2 0/1:72,0,19:6 0/1:75,0,34:7 0/1:230,0,113:45

4 12509976 . T A 999 . . GT:PL:DP 0/1:219,0,209:24 0/1:225,0,233:36 0/1:243,0,195:40 0/1:17,0,127:8 0/1:20,0,110:7 0/1:45,0,13:4 0/1:240,0,255:57

4 12624936 . T C 999 . . GT:PL:DP 0/1:220,0,204:64 0/1:46,0,255:50 0/1:204,0,157:53 0/1:16,0,122:7 0/1:18,0,142:10 0/1:72,0,140:19 0/1:140,0,255:86

4 12700844 . C T 999 . . GT:PL:DP 0/1:140,0,200:26 0/1:73,0,255:31 0/1:153,0,112:28 0/1:59,0,6:6 0/1:38,6,0:2 0/1:28,0,30:4 0/1:136,0,152:39

4 12705089 . T C 999 . . GT:PL:DP 0/1:130,0,255:185 0/1:111,0,255:199 0/1:138,0,255:175 0/1:72,0,241:29 0/1:33,0,104:13 0/1:42,0,227:60 0/1:105,0,255:196

4 12705349 . A G 999 . . GT:PL:DP 0/1:119,0,105:65 0/1:145,0,61:69 0/1:141,0,102:63 0/1:33,0,68:8 0/1:23,4,0:4 0/1:5,0,56:5 0/1:144,0,10:63

4 12762152 . A G 999 . . GT:PL:DP 0/1:220,0,250:36 0/1:150,0,255:72 0/1:89,0,255:110 0/1:66,0,211:16 0/1:15,0,255:17 0/1:119,0,255:28 0/1:161,0,255:127

4 12762339 . C T 999 . . GT:PL:DP 0/1:255,0,255:77 0/1:184,0,255:83 0/1:215,0,255:98 0/1:192,0,88:12 0/1:82,0,255:25 0/1:68,0,255:27 0/1:255,0,255:117

4 12762361 . C T 999 . . GT:PL:DP 0/1:255,0,255:81 0/1:134,0,255:84 0/1:225,0,255:104 0/1:150,0,129:12 0/1:74,0,255:28 0/1:70,0,255:29 0/1:255,0,255:111

4 12762512 . A C 999 . . GT:PL:DP 0/1:255,0,255:59 0/1:255,0,249:89 0/1:255,0,255:135 0/1:66,0,143:14 0/1:219,0,142:19 0/1:232,0,54:19 0/1:237,0,255:142

4 12762549 . C T 999 . . GT:PL:DP 0/1:255,0,255:71 0/1:255,0,255:93 0/1:255,0,213:137 0/1:101,0,141:14 0/1:213,0,171:21 0/1:210,0,86:17 0/1:223,0,255:133

4 12762621 . T C 999 . . GT:PL:DP 0/1:255,0,224:56 0/1:255,0,247:93 0/1:255,0,180:130 0/1:117,0,38:8 0/1:139,0,255:29 0/1:255,0,185:35 0/1:255,0,255:121

4 12926453 . G A 999 . . GT:PL:DP 0/1:250,0,161:43 0/1:176,0,254:31 0/1:181,0,236:28 0/1:53,0,8:3 0/1:23,3,0:1 0/1:115,0,7:9 0/1:237,0,255:58

4 12960887 . C T 999 . . GT:PL:DP 0/1:173,0,255:46 0/1:255,0,255:79 0/1:255,0,255:54 0/1:93,0,146:12 0/1:58,0,86:6 0/1:102,0,195:16 0/1:255,0,255:129

4 12960905 . T C 999 . . GT:PL:DP 0/1:255,0,157:46 0/1:255,0,255:66 0/1:255,0,248:53 0/1:126,0,83:11 0/1:88,0,22:5 0/1:157,0,135:15 0/1:232,0,255:122

4 12961351 . T C 999 . . GT:PL:DP 0/1:165,0,255:47 0/1:255,0,255:108 0/1:255,0,255:97 0/1:123,0,102:10 0/1:95,0,157:13 0/1:142,0,213:18 0/1:250,0,240:181

4 12961605 . C T 999 . . GT:PL:DP 0/1:255,0,46:38 0/1:255,0,255:93 0/1:255,0,255:108 0/1:161,0,160:16 0/1:100,0,87:8 0/1:131,0,255:32 0/1:72,0,195:178

4 12961673 . A G 999 . . GT:PL:DP 0/1:85,0,255:40 0/1:214,0,255:93 0/1:230,0,255:101 0/1:101,0,140:15 0/1:19,0,133:10 0/1:195,0,68:19 0/1:156,0,187:190

4 12961815 . C G 999 . . GT:PL:DP 0/1:255,0,249:53 0/1:255,0,255:82 0/1:208,0,255:127 0/1:170,0,189:25 0/1:143,0,72:9 0/1:100,0,239:17 0/1:61,0,212:199

4 12961826 . G A 999 . . GT:PL:DP 0/1:255,0,242:54 0/1:255,0,255:78 0/1:222,0,255:130 0/1:167,0,183:25 0/1:146,0,95:11 0/1:88,0,253:18 0/1:63,0,221:189

4 12961916 . A C 999 . . GT:PL:DP 0/1:255,0,194:45 0/1:137,0,255:67 0/1:189,0,255:152 0/1:166,0,200:19 0/1:42,0,102:7 0/1:56,0,226:16 0/1:19,0,203:192

4 12962155 . A G 999 . . GT:PL:DP 0/1:243,0,160:42 0/1:99,0,235:73 0/1:192,0,255:108 0/1:104,0,53:12 0/1:98,0,49:10 0/1:134,0,120:20 0/1:99,0,209:180

4 12962181 . C T 999 . . GT:PL:DP 0/1:254,0,211:46 0/1:122,0,255:62 0/1:207,0,255:106 0/1:75,0,145:16 0/1:131,0,57:10 0/1:129,0,191:19 0/1:145,0,255:160

4 12962323 . C T 999 . . GT:PL:DP 0/1:178,0,116:34 0/1:120,0,184:54 0/1:176,0,215:97 0/1:22,0,119:13 0/1:50,0,68:7 0/1:31,0,74:8 0/1:147,0,236:112

4 12974499 . C T 999 . . GT:PL:DP 0/1:87,0,255:235 0/1:91,0,255:249 0/1:64,0,255:223 0/1:73,0,176:37 0/1:123,0,207:75 0/1:105,0,166:27 0/1:92,0,255:246

4 12975067 . C A 999 . . GT:PL:DP 0/1:90,56,255:206 0/1:146,0,255:193 0/1:164,0,255:217 0/1:127,0,248:56 0/1:34,0,174:51 0/1:102,0,211:50 0/1:68,0,238:201

4 12975281 . G T 999 . . GT:PL:DP 0/1:159,0,85:93 0/1:206,0,148:126 0/1:162,0,231:135 0/1:58,0,44:6 0/1:74,0,134:16 0/1:35,5,0:6 0/1:149,0,149:73

4 12975628 . G C 999 . . GT:PL:DP 0/1:208,0,255:162 0/1:255,0,255:200 0/1:72,0,219:184 0/1:147,0,203:19 0/1:101,0,83:15 0/1:142,0,79:20 0/1:162,0,255:212

5 90838 . G C 999 . . GT:PL:DP 0/1:131,0,156:14 0/1:173,0,117:18 0/1:135,0,81:15 0/1:85,5,0:4 0/1:53,6,0:2 0/1:85,0,22:4 0/1:179,0,108:21

5 90841 . C G 999 . . GT:PL:DP 0/1:133,0,156:14 0/1:178,0,93:17 0/1:130,0,81:15 0/1:76,5,0:4 0/1:55,6,0:2 0/1:113,31,22:4 0/1:188,0,93:20

5 127377 . C T 999 . . GT:PL:DP 0/1:255,0,190:151 0/1:255,0,106:141 0/1:255,0,255:81 0/1:255,0,110:41 0/1:255,5,0:35 0/1:234,0,54:26 0/1:255,0,197:173

5 142238 . C A 999 . . GT:PL:DP 0/1:65,0,110:19 0/1:10,0,134:9 0/1:57,0,61:9 0/1:26,3,0:1 0/1:7,0,14:7 0/1:63,8,0:6 0/1:42,0,149:28

5 142239 . G T 999 . . GT:PL:DP 0/1:77,0,115:19 0/1:10,0,91:9 0/1:57,0,61:9 0/1:26,3,0:1 0/1:8,0,12:7 0/1:63,8,0:6 0/1:39,0,155:28

5 142240 . T C 999 . . GT:PL:DP 0/1:72,0,124:19 0/1:10,0,134:9 0/1:36,0,55:8 0/1:26,3,0:1 0/1:7,0,14:7 0/1:49,8,0:6 0/1:39,0,172:28

5 240826 . A T 999 . . GT:PL:DP 0/1:194,0,153:33 0/1:102,0,255:58 0/1:192,0,255:49 0/1:39,0,141:10 0/1:48,0,139:7 0/1:83,0,85:8 0/1:250,0,3:42

5 240857 . C T 999 . . GT:PL:DP 0/1:221,0,198:43 0/1:121,0,255:62 0/1:203,0,255:54 0/1:119,76,106:9 0/1:45,0,146:10 0/1:64,0,106:11 0/1:255,0,153:60

5 378261 . T G 999 . . GT:PL:DP 0/1:32,0,115:10 0/1:94,0,72:16 0/1:40,0,155:21 0/1:50,6,0:2 0/1:32,0,43:5 0/1:45,9,0:3 0/1:88,0,22:11

5 396968 . G T 999 . . GT:PL:DP 0/1:128,0,179:22 0/1:136,0,123:31 0/1:172,0,181:35 0/1:46,0,56:4 0/1:110,0,69:16 0/1:94,0,110:17 0/1:195,0,64:33

5 396989 . T G 999 . . GT:PL:DP 0/1:122,0,166:20 0/1:114,0,126:30 0/1:156,0,179:36 0/1:23,0,59:3 0/1:77,0,78:16 0/1:57,0,102:19 0/1:194,0,62:34

5 617631 . G A 999 . . GT:PL:DP 0/1:124,0,166:32 0/1:103,0,159:29 0/1:138,0,81:11 0/1:52,6,0:2 0/1:24,3,0:1 0/1:29,3,0:1 0/1:79,0,193:16

5 617805 . T G 999 . . GT:PL:DP 0/1:104,0,255:66 0/1:38,0,255:63 0/1:159,0,238:38 0/1:46,0,68:6 0/1:21,0,73:3 0/1:14,0,107:4 0/1:142,0,255:30

5 617806 . C T 999 . . GT:PL:DP 0/1:106,0,255:66 0/1:38,0,255:63 0/1:161,0,238:38 0/1:29,0,97:6 0/1:21,0,73:3 0/1:14,0,107:4 0/1:139,0,255:30

5 617827 . G C 999 . . GT:PL:DP 0/1:166,0,255:69 0/1:179,0,255:71 0/1:174,0,255:46 0/1:45,0,82:6 0/1:56,0,64:4 0/1:31,0,123:7 0/1:162,0,255:40

5 617828 . G T 999 . . GT:PL:DP 0/1:159,0,255:69 0/1:172,0,255:73 0/1:163,0,255:46 0/1:42,0,130:7 0/1:56,0,63:4 0/1:31,0,123:7 0/1:162,0,255:40

5 617943 . A G 999 . . GT:PL:DP 0/1:208,0,255:68 0/1:136,0,255:102 0/1:214,0,255:60 0/1:42,0,167:9 0/1:120,0,7:7 0/1:165,0,61:10 0/1:239,0,255:54

5 617981 . G A 999 . . GT:PL:DP 0/1:168,0,255:75 0/1:117,0,255:96 0/1:182,0,255:59 0/1:29,0,189:10 0/1:100,0,10:6 0/1:139,0,40:8 0/1:189,0,255:53

5 743934 . T C 999 . . GT:PL:DP 0/1:102,0,236:21 0/1:117,0,202:18 0/1:166,0,156:16 0/1:74,9,0:3 0/1:25,0,64:3 0/1:106,9,0:3 0/1:218,0,225:24

5 941037 . G C 999 . . GT:PL:DP 0/1:201,0,237:81 0/1:210,0,189:45 0/1:187,0,237:63 0/1:97,0,34:10 0/1:41,0,81:9 0/1:73,0,48:7 0/1:255,0,255:95

5 941059 . C T 999 . . GT:PL:DP 0/1:183,0,219:76 0/1:198,0,212:47 0/1:134,0,224:59 0/1:80,0,58:9 0/1:13,0,99:8 0/1:11,0,99:8 0/1:205,0,255:106

5 941082 . T G 999 . . GT:PL:DP 0/1:201,0,221:79 0/1:225,0,192:50 0/1:141,0,208:59 0/1:96,0,44:9 0/1:39,0,92:10 0/1:42,0,21:10 0/1:255,0,255:103

5 1031642 . T C 999 . . GT:PL:DP 0/1:154,0,93:11 0/1:55,0,255:21 0/1:255,0,120:35 0/1:19,0,95:6 0/1:35,0,73:6 0/1:73,9,0:3 0/1:255,0,255:49

5 1160137 . T A 999 . . GT:PL:DP 0/1:85,0,255:100 0/1:11,0,189:56 0/1:163,0,255:111 0/1:13,0,22:5 0/1:16,3,0:1 0/1:10,0,72:3 0/1:6,0,217:93

5 1160138 . G T 999 . . GT:PL:DP 0/1:85,0,255:100 0/1:34,0,185:57 0/1:165,0,254:111 0/1:10,0,28:5 0/1:16,3,0:1 0/1:10,0,72:3 0/1:4,0,217:93

5 1160140 . A T 999 . . GT:PL:DP 0/1:102,0,255:102 0/1:62,0,195:60 0/1:175,0,246:116 0/1:10,0,22:5 0/1:16,3,0:1 0/1:10,0,72:3 0/1:8,0,226:92

5 1160144 . T G 999 . . GT:PL:DP 0/1:208,0,255:64 0/1:151,0,195:30 0/1:245,0,255:64 0/1:28,3,0:1 0/1:16,3,0:1 0/1:10,0,72:3 0/1:200,0,255:50

5 1160145 . A T 999 . . GT:PL:DP 0/1:203,0,255:65 0/1:151,0,197:30 0/1:249,0,255:65 0/1:28,3,0:1 0/1:16,3,0:1 0/1:32,0,69:4 0/1:208,0,255:51

5 1160146 . A C 999 . . GT:PL:DP 0/1:209,0,255:65 0/1:151,0,197:30 0/1:249,0,255:65 0/1:28,3,0:1 0/1:16,3,0:1 0/1:32,0,69:4 0/1:207,0,255:51

5 1160147 . C A 999 . . GT:PL:DP 0/1:217,0,255:69 0/1:173,0,195:32 0/1:253,0,250:66 0/1:5,0,16:2 0/1:16,3,0:1 0/1:32,0,69:4 0/1:201,0,255:55

5 1160148 . A C 999 . . GT:PL:DP 0/1:225,0,255:73 0/1:179,0,196:33 0/1:255,0,255:66 0/1:22,0,17:2 0/1:16,3,0:1 0/1:32,0,69:4 0/1:204,0,255:55

5 1160154 . T G 999 . . GT:PL:DP 0/1:255,0,255:79 0/1:246,0,183:37 0/1:255,0,245:68 0/1:28,3,0:1 0/1:16,3,0:1 0/1:32,0,69:4 0/1:228,0,255:54

5 1160155 . G A 999 . . GT:PL:DP 0/1:255,0,255:77 0/1:246,0,181:37 0/1:255,0,249:68 0/1:28,3,0:1 0/1:16,3,0:1 0/1:32,0,69:4 0/1:226,0,255:53

5 1160159 . A G 999 . . GT:PL:DP 0/1:255,0,255:81 0/1:255,0,182:43 0/1:255,0,234:68 0/1:22,0,8:2 0/1:16,3,0:1 0/1:32,0,69:4 0/1:235,0,255:56

5 1256426 . A T 999 . . GT:PL:DP 0/1:37,0,121:15 0/1:116,0,35:16 0/1:66,0,146:19 0/1:31,3,0:1 0/1:28,3,0:1 0/1:28,7,1:3 0/1:82,0,132:25

5 1256428 . T G 999 . . GT:PL:DP 0/1:35,0,135:16 0/1:116,0,32:15 0/1:69,0,177:21 0/1:31,3,0:1 0/1:28,3,0:1 0/1:28,7,1:3 0/1:79,0,132:25

5 1320848 . T A 999 . . GT:PL:DP 0/1:37,3,0:1 0/1:105,9,0:3 0/1:74,6,0:2 0/1:37,3,0:1 0/1:34,3,0:1 0/1:35,3,0:1 0/1:38,3,0:1

5 1320849 . A C 999 . . GT:PL:DP 0/1:37,3,0:1 0/1:67,6,0:2 0/1:74,6,0:2 0/1:37,3,0:1 0/1:33,3,0:1 0/1:35,3,0:1 0/1:38,3,0:1

5 1426848 . G A 999 . . GT:PL:DP 0/1:55,0,146:14 0/1:154,0,11:17 0/1:176,0,135:22 0/1:105,0,43:8 0/1:122,0,76:17 0/1:12,0,104:6 0/1:225,0,255:54

5 1449479 . T C 999 . . GT:PL:DP 0/1:228,0,7:16 0/1:186,0,12:17 0/1:102,0,46:11 0/1:37,3,0:1 0/1:58,0,22:5 0/1:31,3,0:1 0/1:203,0,9:18

5 1653950 . T C 999 . . GT:PL:DP 0/1:168,0,95:25 0/1:147,0,114:22 0/1:168,0,174:29 0/1:32,0,66:5 0/1:29,0,19:2 0/1:35,6,0:2 0/1:147,0,251:43

5 1746860 . G A 999 . . GT:PL:DP 0/1:177,0,255:40 0/1:241,0,255:40 0/1:231,0,255:36 0/1:110,0,20:5 0/1:42,6,0:2 0/1:114,0,198:16 0/1:255,0,255:63

5 1747322 . G A 999 . . GT:PL:DP 0/1:245,0,255:34 0/1:218,0,255:33 0/1:255,0,255:42 0/1:53,0,113:7 0/1:56,0,64:4 0/1:255,0,200:26 0/1:255,0,87:50

5 1782503 . A T 999 . . GT:PL:DP 0/1:34,6,0:8 0/1:33,0,17:11 0/1:35,1,0:7 0/1:17,9,0:3 0/1:13,6,0:2 0/1:33,6,0:2 0/1:163,0,11:22

5 2064500 . T C 999 . . GT:PL:DP 0/1:94,0,255:93 0/1:60,0,255:64 0/1:104,0,255:80 0/1:19,0,80:6 0/1:21,0,42:10 0/1:91,0,33:19 0/1:92,0,255:112

5 2090775 . A G 999 . . GT:PL:DP 0/1:245,0,255:125 0/1:174,0,255:150 0/1:229,0,255:125 0/1:78,0,170:30 0/1:26,0,203:29 0/1:25,0,127:32 0/1:237,0,255:172

5 2090801 . C G 999 . . GT:PL:DP 0/1:221,0,85:92 0/1:188,0,72:102 0/1:191,0,74:95 0/1:88,0,128:27 0/1:119,0,29:16 0/1:59,0,25:23 0/1:234,0,146:118

5 2090856 . C T 999 . . GT:PL:DP 0/1:167,0,29:45 0/1:185,0,95:50 0/1:172,0,93:51 0/1:101,0,105:19 0/1:23,0,42:8 0/1:17,0,0:8 0/1:182,0,113:78

5 2238772 . C G 999 . . GT:PL:DP 0/1:225,0,255:26 0/1:243,0,127:17 0/1:255,0,255:40 0/1:81,0,25:4 0/1:100,9,0:3 0/1:166,7,0:7 0/1:255,0,255:68

5 2329170 . T C 999 . . GT:PL:DP 0/1:255,0,185:29 0/1:255,0,255:45 0/1:255,0,255:33 0/1:53,0,90:5 0/1:67,6,0:2 0/1:99,9,0:4 0/1:255,0,255:40

5 2394744 . G T 999 . . GT:PL:DP 0/1:229,0,201:29 0/1:226,0,242:32 0/1:255,0,66:33 0/1:13,0,79:4 0/1:81,9,0:3 0/1:74,5,0:4 0/1:233,0,255:48

5 2411923 . A T 999 . . GT:PL:DP 0/1:112,0,94:21 0/1:73,0,157:23 0/1:56,0,107:21 0/1:16,0,24:3 0/1:17,0,70:4 0/1:55,6,0:2 0/1:114,0,231:36

5 2525356 . T G 999 . . GT:PL:DP 0/1:189,0,126:19 0/1:215,0,38:16 0/1:255,0,59:39 0/1:191,9,0:8 0/1:137,0,67:23 0/1:194,0,40:31 0/1:255,0,134:50

5 2526945 . T G 999 . . GT:PL:DP 0/1:187,0,255:128 0/1:134,0,255:101 0/1:108,0,255:114 0/1:63,0,211:21 0/1:78,0,129:45 0/1:152,0,109:57 0/1:76,0,243:185

5 2527148 . T A 999 . . GT:PL:DP 0/1:125,0,220:195 0/1:124,0,226:189 0/1:75,0,177:177 0/1:133,0,138:28 0/1:132,0,255:92 0/1:214,0,255:141 0/1:112,0,255:216

5 2527166 . A G 999 . . GT:PL:DP 0/1:109,0,210:181 0/1:114,0,209:186 0/1:67,0,170:177 0/1:120,0,139:26 0/1:128,0,255:90 0/1:222,0,255:141 0/1:95,0,247:198

5 2527174 . T C 999 . . GT:PL:DP 0/1:101,0,210:179 0/1:101,0,206:175 0/1:77,0,178:170 0/1:104,0,150:24 0/1:133,0,255:85 0/1:210,0,255:139 0/1:131,0,250:186

5 2536495 . C T 999 . . GT:PL:DP 0/1:97,0,245:41 0/1:132,0,151:45 0/1:116,0,177:26 0/1:38,0,16:3 0/1:51,6,0:2 0/1:22,0,103:7 0/1:120,0,198:38

5 2577685 . C A 999 . . GT:PL:DP 0/1:32,0,19:9 0/1:60,5,0:12 0/1:71,0,4:12 0/1:34,9,0:3 0/1:17,6,0:2 0/1:55,0,11:5 0/1:31,0,227:40

5 2633286 . A G 999 . . GT:PL:DP 0/1:53,0,214:64 0/1:170,0,200:57 0/1:236,0,190:73 0/1:48,0,98:9 0/1:67,0,145:14 0/1:66,0,74:14 0/1:255,0,246:57

5 2711217 . A C 999 . . GT:PL:DP 0/1:244,0,201:43 0/1:178,0,255:51 0/1:253,0,255:59 0/1:66,0,55:10 0/1:11,0,56:10 0/1:27,0,43:7 0/1:120,0,255:38

5 2711237 . C T 999 . . GT:PL:DP 0/1:135,0,255:42 0/1:168,0,255:50 0/1:85,0,255:54 0/1:36,0,76:11 0/1:12,0,62:9 0/1:29,0,43:6 0/1:119,0,255:39

5 2842674 . C A 999 . . GT:PL:DP 0/1:51,0,118:7 0/1:102,0,97:9 0/1:108,0,55:6 0/1:119,0,22:5 0/1:56,6,0:2 0/1:96,9,0:3 0/1:140,0,141:12

5 2915986 . G A 999 . . GT:PL:DP 0/1:255,0,255:39 0/1:255,0,255:33 0/1:241,0,245:32 0/1:73,0,109:7 0/1:82,9,0:3 0/1:58,11,5:3 0/1:255,0,255:46

5 2953562 . C T 999 . . GT:PL:DP 0/1:27,0,157:25 0/1:71,0,36:14 0/1:48,0,151:35 0/1:44,0,3:6 0/1:20,0,15:3 0/1:23,6,0:2 0/1:21,0,142:41

5 2953745 . A T 999 . . GT:PL:DP 0/1:196,0,238:36 0/1:180,0,251:35 0/1:238,0,208:42 0/1:91,0,62:9 0/1:108,0,15:8 0/1:88,9,0:3 0/1:165,0,217:45

5 3152024 . G A 999 . . GT:PL:DP 0/1:195,0,204:29 0/1:214,0,180:33 0/1:152,0,192:24 0/1:12,0,151:7 0/1:45,0,62:4 0/1:56,6,0:2 0/1:235,0,255:43

5 3176505 . T C 999 . . GT:PL:DP 0/1:130,0,42:61 0/1:148,0,92:48 0/1:129,0,136:71 0/1:116,0,22:15 0/1:33,6,0:2 0/1:59,4,0:13 0/1:163,0,255:74

5 3176534 . T A 999 . . GT:PL:DP 0/1:214,0,138:82 0/1:143,0,116:59 0/1:169,0,178:81 0/1:141,0,20:18 0/1:38,3,0:1 0/1:73,3,0:12 0/1:175,0,255:86

5 3368206 . A G 999 . . GT:PL:DP 0/1:120,53,44:6 0/1:151,128,125:8 0/1:194,132,123:10 0/1:65,39,36:3 0/1:51,6,0:2 0/1:55,0,4:5 0/1:196,164,158:13

5 3421122 . C T 999 . . GT:PL:DP 0/1:255,0,255:244 0/1:226,0,255:253 0/1:249,0,255:254 0/1:255,0,154:61 0/1:226,0,215:250 0/1:194,0,252:245 0/1:234,0,255:261

5 3450705 . C T 999 . . GT:PL:DP 0/1:117,0,143:25 0/1:68,0,161:27 0/1:123,0,102:19 0/1:14,0,8:2 0/1:80,9,0:3 0/1:28,3,0:1 0/1:151,0,198:36

5 3512298 . G A 999 . . GT:PL:DP 0/1:175,0,213:118 0/1:167,0,221:148 0/1:255,0,255:135 0/1:104,0,190:35 0/1:12,0,103:10 0/1:53,0,66:15 0/1:127,0,208:190

5 3524337 . G A 999 . . GT:PL:DP 0/1:255,0,255:216 0/1:147,0,255:205 0/1:255,0,255:213 0/1:80,0,255:51 0/1:57,0,226:60 0/1:26,0,222:53 0/1:174,0,255:210

5 3524492 . T C 999 . . GT:PL:DP 0/1:255,0,255:201 0/1:107,0,255:144 0/1:208,0,255:165 0/1:44,0,255:31 0/1:98,0,231:40 0/1:136,0,170:48 0/1:141,0,255:161

5 3703694 . C T 999 . . GT:PL:DP 0/1:154,0,131:19 0/1:39,0,65:8 0/1:167,0,16:13 0/1:54,0,47:5 0/1:24,0,65:3 0/1:77,0,51:9 0/1:237,0,90:21

5 3767050 . C T 999 . . GT:PL:DP 0/1:255,0,88:25 0/1:242,0,11:25 0/1:180,3,0:11 0/1:87,0,18:4 0/1:47,0,20:4 0/1:68,13,7:3 0/1:255,0,14:29

5 3829249 . C G 90.4478 . . GT:PL:DP 0/1:35,6,0:2 0/1:14,3,0:1 0/1:31,0,166:21 0/1:28,6,0:2 0/1:20,3,0:1 0/1:13,3,0:1 0/1:68,7,0:6

5 3873405 . G C 999 . . GT:PL:DP 0/1:147,0,219:48 0/1:167,0,105:27 0/1:137,0,109:24 0/1:23,0,49:5 0/1:46,0,25:5 0/1:10,0,27:2 0/1:213,0,112:35

5 3917259 . T A 999 . . GT:PL:DP 0/1:17,0,17:3 0/1:152,0,20:13 0/1:147,0,2:16 0/1:59,6,0:2 0/1:22,0,8:2 0/1:51,6,0:2 0/1:254,9,0:22

5 3917261 . A G 999 . . GT:PL:DP 0/1:17,0,17:3 0/1:170,0,23:14 0/1:140,0,25:16 0/1:59,6,0:2 0/1:22,0,8:2 0/1:23,3,0:1 0/1:200,0,89:20

5 4176735 . C A 999 . . GT:PL:DP 0/1:223,0,161:51 0/1:143,0,196:32 0/1:140,0,120:37 0/1:86,0,84:9 0/1:56,0,91:6 0/1:12,0,143:16 0/1:254,0,232:112

5 4176777 . T A 999 . . GT:PL:DP 0/1:255,0,230:74 0/1:255,0,242:55 0/1:255,0,197:49 0/1:120,0,101:15 0/1:158,0,134:12 0/1:118,0,135:23 0/1:255,0,255:149

5 4176824 . G A 999 . . GT:PL:DP 0/1:230,0,255:84 0/1:255,0,255:80 0/1:222,0,255:74 0/1:146,0,135:19 0/1:134,0,136:11 0/1:74,0,255:34 0/1:255,0,255:156

5 4176868 . G T 999 . . GT:PL:DP 0/1:255,0,255:108 0/1:255,0,255:91 0/1:255,0,255:85 0/1:98,0,255:24 0/1:30,0,83:8 0/1:255,0,144:35 0/1:255,0,255:157

5 4377858 . T A 999 . . GT:PL:DP 0/1:255,0,115:28 0/1:195,0,255:31 0/1:255,0,255:43 0/1:81,0,77:7 0/1:20,0,123:5 0/1:70,6,0:2 0/1:255,0,255:64

5 4468528 . C T 999 . . GT:PL:DP 0/1:62,3,0:21 0/1:67,0,1:21 0/1:116,0,4:23 0/1:38,3,8:8 0/1:40,6,0:2 0/1:34,3,0:3 0/1:121,0,14:45

5 4542329 . C A 999 . . GT:PL:DP 0/1:250,0,53:37 0/1:213,0,32:30 0/1:234,0,182:38 0/1:47,0,95:10 0/1:72,0,67:7 0/1:7,0,23:2 0/1:255,0,233:104

5 4558412 . G C 999 . . GT:PL:DP 0/1:255,0,255:44 0/1:196,0,222:29 0/1:255,0,255:39 0/1:119,0,140:11 0/1:97,0,54:6 0/1:72,6,0:2 0/1:255,0,255:75

5 4624833 . A G 999 . . GT:PL:DP 0/1:81,0,152:210 0/1:162,0,255:206 0/1:167,0,255:230 0/1:156,0,255:83 0/1:79,0,255:70 0/1:109,0,255:89 0/1:110,0,255:194

5 4624845 . T A 999 . . GT:PL:DP 0/1:102,0,147:214 0/1:176,0,255:207 0/1:196,0,255:232 0/1:164,0,255:82 0/1:101,0,255:70 0/1:130,0,255:86 0/1:150,0,255:193

5 4624880 . A T 999 . . GT:PL:DP 0/1:173,0,126:216 0/1:215,0,255:206 0/1:255,0,255:232 0/1:255,0,255:96 0/1:173,0,255:57 0/1:194,0,255:71 0/1:235,0,255:190

5 4625075 . C T 999 . . GT:PL:DP 0/1:242,0,80:212 0/1:255,0,255:212 0/1:255,0,255:231 0/1:255,0,255:105 0/1:255,0,255:128 0/1:255,0,255:141 0/1:255,0,255:190

5 4625100 . G A 999 . . GT:PL:DP 0/1:225,0,114:198 0/1:255,0,255:210 0/1:255,0,255:229 0/1:255,0,255:105 0/1:232,0,255:96 0/1:246,0,255:108 0/1:226,0,255:188

5 4625196 . A G 999 . . GT:PL:DP 0/1:132,0,13:213 0/1:254,0,235:212 0/1:255,0,255:233 0/1:253,0,255:123 0/1:255,0,255:128 0/1:244,0,255:168 0/1:228,0,255:188

5 4625994 . A C 999 . . GT:PL:DP 0/1:160,0,4:196 0/1:255,0,255:199 0/1:255,0,255:208 0/1:255,0,255:83 0/1:255,0,189:96 0/1:255,0,177:164 0/1:255,0,255:192

5 4626561 . C A 999 . . GT:PL:DP 0/1:170,0,57:205 0/1:255,0,255:209 0/1:255,0,255:227 0/1:255,0,255:92 0/1:255,0,223:62 0/1:255,0,191:55 0/1:255,0,255:189

5 4626661 . A G 999 . . GT:PL:DP 0/1:128,1,0:197 0/1:255,0,255:205 0/1:255,0,255:223 0/1:255,0,255:86 0/1:255,0,255:61 0/1:255,0,255:58 0/1:255,0,255:188

5 4626681 . C T 999 . . GT:PL:DP 0/1:129,0,34:201 0/1:252,0,227:206 0/1:255,0,255:222 0/1:255,0,255:84 0/1:252,0,255:57 0/1:245,0,254:69 0/1:255,0,255:190

5 4627161 . A G 999 . . GT:PL:DP 0/1:236,0,117:193 0/1:255,0,255:193 0/1:255,0,255:228 0/1:255,0,228:70 0/1:255,0,255:94 0/1:255,0,143:78 0/1:255,0,255:183

5 4627166 . G C 999 . . GT:PL:DP 0/1:239,0,132:198 0/1:255,0,255:196 0/1:255,0,255:224 0/1:255,0,230:70 0/1:255,0,255:90 0/1:252,0,139:76 0/1:255,0,255:185

5 4627251 . A G 999 . . GT:PL:DP 0/1:192,0,166:190 0/1:247,0,255:197 0/1:255,0,255:217 0/1:242,0,210:77 0/1:220,0,255:58 0/1:200,0,37:80 0/1:210,0,255:187

5 4627260 . A G 999 . . GT:PL:DP 0/1:180,0,168:193 0/1:220,0,244:195 0/1:254,0,255:219 0/1:248,0,255:78 0/1:237,0,255:58 0/1:189,0,43:89 0/1:211,0,255:187

5 4627266 . A T 999 . . GT:PL:DP 0/1:185,0,168:199 0/1:219,0,237:196 0/1:254,0,255:217 0/1:245,0,255:77 0/1:250,0,255:61 0/1:176,0,41:93 0/1:208,0,255:186

5 4627641 . A C 999 . . GT:PL:DP 0/1:255,0,255:164 0/1:255,0,255:160 0/1:255,0,255:226 0/1:157,0,255:34 0/1:157,0,230:41 0/1:255,0,255:63 0/1:34,0,255:189

5 4628067 . A G 999 . . GT:PL:DP 0/1:108,0,255:164 0/1:132,0,255:171 0/1:83,0,255:200 0/1:99,0,242:27 0/1:96,0,255:28 0/1:132,0,255:38 0/1:83,0,255:185

5 4628068 . C T 999 . . GT:PL:DP 0/1:106,0,255:162 0/1:134,0,255:171 0/1:83,0,255:200 0/1:99,0,229:27 0/1:85,0,255:28 0/1:134,0,255:37 0/1:82,0,255:185

5 4628078 . T C 999 . . GT:PL:DP 0/1:95,0,250:165 0/1:131,0,255:172 0/1:91,0,255:211 0/1:83,0,229:27 0/1:89,0,255:28 0/1:117,0,255:40 0/1:114,0,255:184

5 4628135 . A T 999 . . GT:PL:DP 0/1:91,0,247:185 0/1:102,0,255:166 0/1:90,0,255:205 0/1:89,0,255:36 0/1:23,0,207:32 0/1:32,0,255:46 0/1:108,0,255:177

5 4628143 . T A 999 . . GT:PL:DP 0/1:106,0,243:180 0/1:113,0,255:166 0/1:107,0,255:201 0/1:88,0,255:35 0/1:9,0,214:35 0/1:28,0,255:51 0/1:110,0,255:180

5 4628148 . G A 999 . . GT:PL:DP 0/1:115,0,217:182 0/1:141,0,255:163 0/1:116,0,255:199 0/1:90,0,255:35 0/1:13,0,221:33 0/1:42,0,255:49 0/1:121,0,255:177

5 4628338 . T G 999 . . GT:PL:DP 0/1:166,0,155:82 0/1:219,0,166:109 0/1:190,0,200:100 0/1:149,0,104:14 0/1:144,0,52:11 0/1:221,0,89:19 0/1:246,0,255:127

5 4641811 . G A 999 . . GT:PL:DP 0/1:255,0,255:39 0/1:181,0,255:46 0/1:255,0,255:52 0/1:133,0,123:10 0/1:98,0,81:8 0/1:81,0,127:8 0/1:255,0,255:69

5 4641989 . C T 999 . . GT:PL:DP 0/1:255,0,255:52 0/1:255,0,255:66 0/1:209,0,255:54 0/1:121,0,163:14 0/1:224,0,68:14 0/1:117,0,121:9 0/1:255,0,255:79

5 4643909 . A G 999 . . GT:PL:DP 0/1:184,0,255:46 0/1:138,0,255:54 0/1:75,0,255:58 0/1:23,0,243:13 0/1:112,0,113:8 0/1:64,0,153:9 0/1:235,0,255:70

5 4643969 . A G 999 . . GT:PL:DP 0/1:255,0,248:42 0/1:255,0,57:39 0/1:255,0,129:45 0/1:255,4,0:14 0/1:133,0,80:9 0/1:64,0,39:4 0/1:255,0,255:64

5 4644842 . C T 999 . . GT:PL:DP 0/1:62,0,246:28 0/1:231,0,134:22 0/1:239,0,239:35 0/1:25,0,134:8 0/1:138,0,17:6 0/1:64,0,17:5 0/1:135,0,255:79

5 4644860 . T C 999 . . GT:PL:DP 0/1:243,0,100:33 0/1:193,0,136:22 0/1:230,0,255:42 0/1:77,0,115:9 0/1:14,0,163:7 0/1:19,0,134:9 0/1:237,0,255:72

5 4644914 . A T 999 . . GT:PL:DP 0/1:235,0,89:29 0/1:192,0,121:24 0/1:245,0,255:48 0/1:125,0,36:9 0/1:20,0,177:9 0/1:57,0,150:10 0/1:255,0,255:83

5 4645272 . A C 999 . . GT:PL:DP 0/1:255,0,243:39 0/1:255,0,41:19 0/1:167,0,225:48 0/1:22,0,96:5 0/1:162,0,67:10 0/1:114,0,187:18 0/1:255,0,255:74

5 4646093 . G A 999 . . GT:PL:DP 0/1:189,0,255:50 0/1:176,0,255:62 0/1:255,0,255:80 0/1:213,0,57:16 0/1:53,0,255:22 0/1:104,0,255:25 0/1:90,0,255:120

5 4646096 . A T 999 . . GT:PL:DP 0/1:224,0,255:48 0/1:176,0,255:63 0/1:255,0,255:79 0/1:215,0,57:16 0/1:52,0,255:23 0/1:88,0,255:25 0/1:87,0,255:119

5 4646134 . A G 999 . . GT:PL:DP 0/1:255,0,255:55 0/1:211,0,255:68 0/1:255,0,255:70 0/1:204,0,81:16 0/1:111,0,255:18 0/1:203,0,255:27 0/1:159,0,255:116

5 4646228 . T C 999 . . GT:PL:DP 0/1:255,0,216:50 0/1:255,0,255:72 0/1:255,0,255:64 0/1:235,0,50:17 0/1:16,0,233:16 0/1:143,0,255:29 0/1:255,0,255:121

5 4646300 . G C 999 . . GT:PL:DP 0/1:255,0,170:38 0/1:255,0,255:66 0/1:255,0,219:65 0/1:236,0,20:18 0/1:93,0,187:14 0/1:37,0,255:20 0/1:248,0,255:93

5 4647716 . C T 999 . . GT:PL:DP 0/1:255,0,255:48 0/1:255,0,174:47 0/1:255,0,254:50 0/1:181,0,71:12 0/1:52,0,27:4 0/1:27,0,47:5 0/1:255,0,255:82

5 4648478 . G A 999 . . GT:PL:DP 0/1:255,0,137:29 0/1:255,0,183:29 0/1:255,0,25:27 0/1:107,0,127:11 0/1:82,0,92:11 0/1:107,0,53:6 0/1:255,0,65:43

5 4648571 . C T 999 . . GT:PL:DP 0/1:255,0,42:21 0/1:201,0,129:28 0/1:255,0,29:23 0/1:53,0,74:5 0/1:88,0,82:6 0/1:151,0,16:7 0/1:255,0,57:50

5 4871887 . T C 999 . . GT:PL:DP 0/1:109,0,3:19 0/1:95,0,37:12 0/1:125,0,32:20 0/1:70,0,14:6 0/1:54,0,69:12 0/1:10,0,53:6 0/1:144,0,4:28

5 4871917 . C A 999 . . GT:PL:DP 0/1:118,0,10:20 0/1:54,0,70:13 0/1:121,0,49:22 0/1:39,0,17:5 0/1:29,0,71:8 0/1:16,0,24:6 0/1:142,0,16:23

5 4871918 . A C 999 . . GT:PL:DP 0/1:109,0,8:18 0/1:54,0,70:13 0/1:120,0,66:23 0/1:52,0,17:5 0/1:29,0,78:8 0/1:16,0,24:6 0/1:134,0,13:24

5 5055690 . T C 999 . . GT:PL:DP 0/1:130,0,143:19 0/1:45,0,184:26 0/1:139,0,190:30 0/1:43,0,34:4 0/1:5,0,13:3 0/1:71,9,0:3 0/1:213,0,30:55

5 5164531 . T C 999 . . GT:PL:DP 0/1:255,0,255:216 0/1:204,0,255:136 0/1:138,0,255:225 0/1:230,0,203:36 0/1:78,0,140:13 0/1:172,0,176:24 0/1:172,0,255:216

5 5359582 . G A 999 . . GT:PL:DP 0/1:134,0,85:16 0/1:166,0,195:30 0/1:208,0,165:34 0/1:7,0,41:3 0/1:36,3,0:1 0/1:62,6,0:2 0/1:137,0,208:36

5 5472320 . C T 999 . . GT:PL:DP 0/1:255,0,255:30 0/1:255,0,255:43 0/1:255,0,255:41 0/1:71,0,129:8 0/1:62,0,111:7 0/1:74,0,15:3 0/1:255,0,255:49

5 5472454 . C G 999 . . GT:PL:DP 0/1:239,0,255:31 0/1:193,0,255:29 0/1:255,0,255:45 0/1:77,0,82:6 0/1:127,0,58:6 0/1:111,9,0:3 0/1:255,0,255:48

5 5476758 . T C 999 . . GT:PL:DP 0/1:255,0,244:36 0/1:158,0,184:23 0/1:222,0,255:37 0/1:93,0,25:4 0/1:106,0,56:6 0/1:65,9,0:3 0/1:255,0,255:48

5 5499282 . T C 999 . . GT:PL:DP 0/1:255,0,255:131 0/1:255,0,255:153 0/1:255,0,227:78 0/1:255,0,216:28 0/1:29,0,207:11 0/1:130,0,193:13 0/1:150,0,255:112

5 5499372 . T C 999 . . GT:PL:DP 0/1:255,0,255:114 0/1:255,0,255:144 0/1:255,0,200:82 0/1:255,0,215:28 0/1:17,0,255:18 0/1:92,0,255:22 0/1:255,0,255:129

5 5509394 . C T 999 . . GT:PL:DP 0/1:255,0,255:37 0/1:255,0,255:30 0/1:255,0,255:42 0/1:94,0,95:6 0/1:73,6,0:2 0/1:79,6,0:2 0/1:255,0,222:43

5 5771755 . A T 999 . . GT:PL:DP 0/1:162,0,147:18 0/1:242,0,130:32 0/1:76,0,255:35 0/1:96,0,52:7 0/1:63,0,97:8 0/1:191,0,14:13 0/1:189,41,135:27

5 5878122 . C A 999 . . GT:PL:DP 0/1:74,0,77:10 0/1:98,0,33:12 0/1:89,0,34:11 0/1:66,0,17:4 0/1:20,0,44:4 0/1:22,0,7:3 0/1:75,0,36:12

5 5889881 . T A 999 . . GT:PL:DP 0/1:175,0,176:64 0/1:116,0,179:70 0/1:165,0,192:100 0/1:61,17,101:10 0/1:24,2,0:6 0/1:8,0,26:9 0/1:144,0,255:177

5 5890711 . G A 999 . . GT:PL:DP 0/1:136,0,134:62 0/1:228,0,144:45 0/1:145,0,159:59 0/1:97,0,48:11 0/1:5,0,45:10 0/1:79,2,0:11 0/1:187,0,255:98

5 5890795 . G T 999 . . GT:PL:DP 0/1:129,0,73:57 0/1:45,0,161:43 0/1:132,0,119:61 0/1:72,0,13:9 0/1:86,0,36:17 0/1:159,95,90:20 0/1:149,0,223:78

5 5918777 . C T 999 . . GT:PL:DP 0/1:255,0,49:46 0/1:255,3,0:41 0/1:254,1,0:35 0/1:31,0,0:2 0/1:75,0,44:12 0/1:45,0,86:36 0/1:255,0,69:78

5 6071868 . G C 999 . . GT:PL:DP 0/1:166,0,255:68 0/1:131,0,255:43 0/1:202,0,255:55 0/1:50,0,120:9 0/1:63,0,228:21 0/1:144,0,8:13 0/1:12,0,255:150

5 6072083 . T A 999 . . GT:PL:DP 0/1:173,0,255:53 0/1:123,0,255:46 0/1:159,0,166:29 0/1:70,0,72:7 0/1:30,0,169:15 0/1:58,0,81:9 0/1:82,0,255:129

5 6072198 . T C 999 . . GT:PL:DP 0/1:146,0,255:60 0/1:165,0,246:54 0/1:186,0,136:45 0/1:70,0,50:6 0/1:13,0,24:2 0/1:14,0,27:4 0/1:140,0,255:116

5 6085533 . A T 999 . . GT:PL:DP 0/1:67,0,44:17 0/1:118,0,5:15 0/1:145,0,24:35 0/1:38,9,0:3 0/1:94,7,0:7 0/1:41,9,0:3 0/1:114,0,63:29

5 6085540 . T C 999 . . GT:PL:DP 0/1:83,0,41:18 0/1:123,0,5:15 0/1:151,0,39:36 0/1:44,9,0:3 0/1:51,3,0:5 0/1:40,9,0:3 0/1:133,0,51:31

5 6128180 . T A 999 . . GT:PL:DP 0/1:185,0,230:81 0/1:159,0,211:68 0/1:186,0,157:52 0/1:112,9,0:8 0/1:59,0,131:9 0/1:37,3,0:1 0/1:170,0,223:102

5 6164333 . T G 999 . . GT:PL:DP 0/1:149,0,255:110 0/1:192,0,97:98 0/1:148,0,113:103 0/1:119,0,32:26 0/1:28,0,144:18 0/1:36,0,165:31 0/1:168,0,177:186

5 6164455 . G T 999 . . GT:PL:DP 0/1:113,0,126:199 0/1:33,0,4:173 0/1:38,0,39:180 0/1:140,0,13:38 0/1:27,0,195:28 0/1:73,0,255:42 0/1:102,0,102:176

5 6164690 . A T 999 . . GT:PL:DP 0/1:196,0,242:138 0/1:192,0,159:90 0/1:116,0,162:95 0/1:109,0,67:21 0/1:56,6,0:2 0/1:81,0,99:11 0/1:167,0,255:200

5 6164866 . C G 999 . . GT:PL:DP 0/1:138,0,119:62 0/1:122,0,68:52 0/1:102,0,30:65 0/1:62,0,16:10 0/1:50,0,28:5 0/1:22,0,0:4 0/1:101,0,149:55

5 6187151 . T G 999 . . GT:PL:DP 0/1:255,0,255:47 0/1:255,0,254:29 0/1:197,0,255:31 0/1:153,0,186:13 0/1:237,2,0:17 0/1:99,9,0:3 0/1:255,0,255:63

5 6216229 . A G 999 . . GT:PL:DP 0/1:238,0,30:24 0/1:54,0,23:3 0/1:205,0,18:15 0/1:49,0,25:3 0/1:29,3,0:1 0/1:54,0,4:3 0/1:217,0,24:13

5 6318201 . T G 999 . . GT:PL:DP 0/1:93,0,175:21 0/1:154,0,72:13 0/1:195,0,255:39 0/1:37,3,0:1 0/1:13,0,34:2 0/1:76,6,0:2 0/1:76,0,173:20

5 6396159 . C A 999 . . GT:PL:DP 0/1:247,6,0:22 0/1:229,0,176:36 0/1:166,0,239:34 0/1:29,3,0:1 0/1:72,9,0:3 0/1:27,3,0:1 0/1:148,0,252:43

5 6454631 . A G 999 . . GT:PL:DP 0/1:255,0,255:115 0/1:251,0,255:137 0/1:228,0,255:128 0/1:93,0,74:12 0/1:94,0,104:26 0/1:193,0,178:91 0/1:214,0,255:201

5 6454673 . G T 999 . . GT:PL:DP 0/1:255,0,255:116 0/1:152,0,255:123 0/1:212,0,255:127 0/1:93,0,169:16 0/1:131,0,191:22 0/1:236,0,255:109 0/1:245,0,255:190

5 6454739 . A G 999 . . GT:PL:DP 0/1:255,0,255:136 0/1:175,0,255:135 0/1:255,0,255:129 0/1:132,0,143:14 0/1:93,0,174:25 0/1:229,0,222:108 0/1:230,0,255:176

5 6489676 . A G 999 . . GT:PL:DP 0/1:160,0,104:25 0/1:137,0,190:29 0/1:162,0,166:28 0/1:19,0,31:2 0/1:43,1,0:5 0/1:53,5,0:6 0/1:182,0,214:46

5 6500792 . G A 999 . . GT:PL:DP 0/1:255,0,16:32 0/1:255,0,17:36 0/1:255,3,0:51 0/1:93,0,17:4 0/1:57,3,0:3 0/1:110,0,13:21 0/1:255,0,64:45

5 6500803 . G A 999 . . GT:PL:DP 0/1:255,0,29:27 0/1:255,0,17:36 0/1:255,1,0:50 0/1:93,0,17:4 0/1:48,1,0:4 0/1:100,0,12:23 0/1:255,0,77:42

5 6500805 . C G 999 . . GT:PL:DP 0/1:255,0,29:27 0/1:255,0,17:36 0/1:255,1,0:50 0/1:65,0,20:3 0/1:57,1,0:4 0/1:97,0,12:23 0/1:255,0,74:42

5 6501000 . A T 999 . . GT:PL:DP 0/1:93,0,5:8 0/1:51,1,0:3 0/1:14,0,11:6 0/1:32,3,0:1 0/1:62,6,0:2 0/1:81,9,0:3 0/1:41,0,12:8

5 6533011 . A T 999 . . GT:PL:DP 0/1:207,0,202:39 0/1:200,0,110:40 0/1:150,0,200:33 0/1:25,0,79:4 0/1:35,0,185:12 0/1:27,0,245:28 0/1:240,0,205:48

5 6533368 . G A 999 . . GT:PL:DP 0/1:217,0,255:57 0/1:244,0,195:70 0/1:198,0,243:70 0/1:123,0,162:14 0/1:142,0,126:15 0/1:105,0,101:19 0/1:129,0,255:124

5 6565490 . T C 999 . . GT:PL:DP 0/1:206,0,191:27 0/1:190,0,62:13 0/1:155,0,241:34 0/1:81,6,0:6 0/1:36,3,0:1 0/1:74,9,0:3 0/1:123,0,226:24

5 6622650 . G T 999 . . GT:PL:DP 0/1:115,0,66:19 0/1:169,0,107:26 0/1:94,0,79:29 0/1:25,0,29:4 0/1:37,6,0:2 0/1:46,0,45:9 0/1:174,0,129:44

5 6627103 . G T 999 . . GT:PL:DP 0/1:255,0,17:31 0/1:229,0,79:30 0/1:255,0,65:34 0/1:129,0,66:11 0/1:39,0,46:6 0/1:60,0,71:11 0/1:255,0,223:64

5 6648887 . T A 999 . . GT:PL:DP 0/1:94,0,69:17 0/1:103,0,60:16 0/1:103,0,74:14 0/1:17,3,0:1 0/1:73,0,15:7 0/1:91,0,32:8 0/1:114,0,55:16

5 6929983 . G A 999 . . GT:PL:DP 0/1:255,0,255:139 0/1:255,0,255:157 0/1:255,0,243:158 0/1:134,0,149:28 0/1:12,0,22:98 0/1:11,0,2:164 0/1:255,0,255:194

5 7023751 . T A 999 . . GT:PL:DP 0/1:255,2,0:31 0/1:114,0,3:8 0/1:122,6,0:6 0/1:60,0,25:4 0/1:17,0,27:2 0/1:18,0,29:2 0/1:255,0,81:63

5 7037271 . T A 999 . . GT:PL:DP 0/1:195,0,255:56 0/1:226,0,255:62 0/1:255,0,158:33 0/1:118,0,72:8 0/1:92,0,123:9 0/1:49,0,190:11 0/1:255,0,255:128

5 7039880 . G A 999 . . GT:PL:DP 0/1:175,0,100:61 0/1:92,0,226:50 0/1:204,0,118:33 0/1:40,0,24:5 0/1:87,0,47:6 0/1:132,0,89:15 0/1:123,0,82:136

5 7111633 . T A 999 . . GT:PL:DP 0/1:255,0,10:36 0/1:255,0,66:51 0/1:255,0,70:42 0/1:67,6,0:2 0/1:97,0,12:5 0/1:224,0,45:15 0/1:255,0,213:76

5 7130969 . G A 999 . . GT:PL:DP 0/1:46,0,19:16 0/1:83,0,19:14 0/1:45,0,34:16 0/1:23,3,0:1 0/1:15,6,0:2 0/1:11,3,0:1 0/1:100,0,64:26

5 7130972 . C G 999 . . GT:PL:DP 0/1:59,0,11:14 0/1:84,0,4:13 0/1:48,0,27:13 0/1:23,3,0:1 0/1:15,6,0:2 0/1:11,3,0:1 0/1:106,0,64:26

5 7144949 . T A 999 . . GT:PL:DP 0/1:34,0,238:306 0/1:140,0,255:295 0/1:39,0,233:300 0/1:175,0,236:138 0/1:255,0,255:184 0/1:230,0,255:194 0/1:64,0,237:334

5 7272538 . G A 999 . . GT:PL:DP 0/1:255,0,225:45 0/1:255,0,255:65 0/1:225,0,255:53 0/1:120,0,193:16 0/1:37,0,136:9 0/1:147,0,99:13 0/1:255,0,255:76

5 7413723 . C A 999 . . GT:PL:DP 0/1:255,0,221:55 0/1:196,0,255:37 0/1:170,0,173:25 0/1:116,0,5:6 0/1:42,0,105:6 0/1:91,9,0:3 0/1:255,0,255:54

5 7475220 . T C 999 . . GT:PL:DP 0/1:148,0,255:55 0/1:255,0,66:28 0/1:255,0,210:52 0/1:238,0,76:18 0/1:64,0,121:8 0/1:38,3,0:3 0/1:255,0,186:77

5 7559956 . C A 999 . . GT:PL:DP 0/1:202,0,255:27 0/1:191,0,255:25 0/1:255,0,243:50 0/1:104,9,0:3 0/1:106,0,142:14 0/1:151,0,4:7 0/1:255,0,132:34

5 7604122 . G A 999 . . GT:PL:DP 0/1:165,2,0:12 0/1:151,0,183:24 0/1:189,0,123:21 0/1:12,0,122:8 0/1:38,6,0:2 0/1:25,0,5:2 0/1:227,0,204:24

5 7609220 . C G 999 . . GT:PL:DP 0/1:70,0,234:35 0/1:118,0,246:34 0/1:229,0,135:27 0/1:23,0,40:5 0/1:22,0,0:3 0/1:51,0,69:11 0/1:255,0,233:73

5 7609229 . C A 999 . . GT:PL:DP 0/1:32,0,222:32 0/1:74,0,224:31 0/1:191,0,152:26 0/1:24,0,43:4 0/1:7,0,51:8 0/1:45,0,117:16 0/1:255,0,255:76

5 7881686 . C T 999 . . GT:PL:DP 0/1:131,0,156:37 0/1:35,0,158:17 0/1:77,0,157:19 0/1:43,0,25:4 0/1:46,0,26:5 0/1:54,0,33:6 0/1:107,0,184:40

5 7881923 . A C 999 . . GT:PL:DP 0/1:233,0,255:78 0/1:161,0,255:45 0/1:253,0,255:45 0/1:109,0,141:13 0/1:139,0,56:7 0/1:108,0,107:9 0/1:171,0,255:176

5 7881955 . G A 999 . . GT:PL:DP 0/1:255,0,255:102 0/1:255,0,255:59 0/1:255,0,255:52 0/1:144,0,217:16 0/1:103,0,140:10 0/1:111,0,240:18 0/1:205,0,255:184

5 7901113 . G C 999 . . GT:PL:DP 0/1:248,0,255:220 0/1:204,0,255:228 0/1:255,0,255:210 0/1:174,0,242:91 0/1:148,0,255:86 0/1:187,0,255:96 0/1:205,0,255:216

5 7901216 . G C 999 . . GT:PL:DP 0/1:229,0,255:211 0/1:226,0,255:226 0/1:226,0,255:169 0/1:255,0,255:77 0/1:151,0,240:79 0/1:173,0,110:59 0/1:255,0,255:216

5 7901477 . A G 999 . . GT:PL:DP 0/1:134,0,255:199 0/1:242,0,255:218 0/1:184,0,255:227 0/1:95,0,234:58 0/1:198,0,255:78 0/1:89,0,255:64 0/1:120,0,255:211

5 8107498 . G C 999 . . GT:PL:DP 0/1:134,0,252:225 0/1:77,0,252:242 0/1:223,0,221:225 0/1:149,0,233:53 0/1:21,0,180:56 0/1:31,0,204:127 0/1:19,0,173:266

5 8107548 . G T 999 . . GT:PL:DP 0/1:194,0,255:213 0/1:185,0,255:213 0/1:255,0,255:208 0/1:232,0,255:61 0/1:56,0,255:74 0/1:27,0,255:122 0/1:187,0,255:206

5 8107580 . A G 999 . . GT:PL:DP 0/1:138,0,255:148 0/1:150,0,255:126 0/1:249,0,255:136 0/1:190,0,255:52 0/1:76,0,255:67 0/1:98,0,255:123 0/1:60,0,255:137

5 8107598 . C A 999 . . GT:PL:DP 0/1:143,0,255:104 0/1:163,0,248:103 0/1:214,0,255:105 0/1:177,0,240:51 0/1:104,0,255:60 0/1:104,0,249:88 0/1:44,0,255:129

5 8107881 . A G 999 . . GT:PL:DP 0/1:255,0,255:262 0/1:208,0,255:258 0/1:149,0,255:260 0/1:165,0,254:62 0/1:146,0,254:34 0/1:29,0,255:46 0/1:104,0,251:254

5 8108156 . T C 999 . . GT:PL:DP 0/1:203,0,255:227 0/1:13,0,226:221 0/1:149,0,255:232 0/1:255,0,255:114 0/1:29,0,222:117 0/1:154,0,255:181 0/1:151,0,255:233

5 8139145 . A T 999 . . GT:PL:DP 0/1:221,0,134:135 0/1:214,0,232:97 0/1:255,0,255:116 0/1:146,0,114:21 0/1:110,0,109:23 0/1:113,0,112:23 0/1:208,0,97:145

5 8139158 . A G 999 . . GT:PL:DP 0/1:252,0,215:146 0/1:227,0,255:102 0/1:235,0,255:118 0/1:141,0,165:24 0/1:133,0,138:23 0/1:106,0,131:20 0/1:252,0,178:144

5 8139216 . A T 999 . . GT:PL:DP 0/1:184,0,184:85 0/1:136,0,218:56 0/1:197,0,233:72 0/1:95,0,89:15 0/1:28,1,0:2 0/1:59,6,0:2 0/1:181,0,243:98

5 8193472 . A T 999 . . GT:PL:DP 0/1:124,0,169:26 0/1:81,0,224:22 0/1:166,0,255:42 0/1:64,0,22:6 0/1:85,0,13:10 0/1:11,0,102:8 0/1:71,0,105:10

5 8193482 . A T 999 . . GT:PL:DP 0/1:97,0,172:25 0/1:49,0,224:22 0/1:115,0,255:42 0/1:66,0,22:6 0/1:77,0,16:9 0/1:11,0,96:8 0/1:63,0,91:13

5 8213814 . C T 999 . . GT:PL:DP 0/1:255,0,255:115 0/1:255,0,255:142 0/1:255,0,255:114 0/1:150,0,170:33 0/1:25,0,93:14 0/1:19,0,56:5 0/1:255,0,255:205

5 8222781 . T C 999 . . GT:PL:DP 0/1:27,0,137:30 0/1:47,0,180:39 0/1:88,0,233:41 0/1:10,0,79:4 0/1:24,1,0:2 0/1:40,0,18:5 0/1:52,0,102:16

5 8283660 . C G 999 . . GT:PL:DP 0/1:137,0,156:30 0/1:137,0,109:28 0/1:215,0,114:48 0/1:44,0,58:9 0/1:29,0,19:2 0/1:44,5,0:4 0/1:185,0,139:39

5 8322225 . T A 999 . . GT:PL:DP 0/1:153,0,160:43 0/1:55,0,255:39 0/1:168,0,239:38 0/1:14,0,118:8 0/1:50,0,35:7 0/1:97,0,95:7 0/1:80,0,255:42

5 8459063 . T A 999 . . GT:PL:DP 0/1:47,0,48:34 0/1:56,0,134:36 0/1:37,0,85:27 0/1:32,0,20:4 0/1:9,0,16:2 0/1:15,0,9:6 0/1:75,0,164:41

5 8550860 . G A 999 . . GT:PL:DP 0/1:255,0,57:107 0/1:149,0,255:90 0/1:255,0,238:151 0/1:103,0,151:15 0/1:185,0,178:20 0/1:136,0,199:19 0/1:255,0,255:136

5 8551287 . G A 999 . . GT:PL:DP 0/1:255,0,255:167 0/1:255,0,255:113 0/1:243,0,255:212 0/1:238,0,174:20 0/1:216,0,174:21 0/1:197,0,255:27 0/1:255,0,255:223

5 8607227 . C A 999 . . GT:PL:DP 0/1:139,0,188:21 0/1:182,0,202:21 0/1:182,0,255:42 0/1:97,0,25:4 0/1:17,3,0:1 0/1:28,0,115:6 0/1:255,0,255:56

5 8607316 . T A 999 . . GT:PL:DP 0/1:192,0,227:31 0/1:255,0,255:39 0/1:210,0,255:40 0/1:121,0,22:5 0/1:33,0,35:2 0/1:89,0,100:6 0/1:255,0,255:57

5 8607320 . T A 999 . . GT:PL:DP 0/1:198,0,173:28 0/1:255,0,255:40 0/1:245,0,255:44 0/1:121,0,22:5 0/1:33,0,32:2 0/1:58,0,103:5 0/1:255,0,255:57

5 8619174 . G T 999 . . GT:PL:DP 0/1:210,0,249:41 0/1:206,0,174:29 0/1:192,0,255:38 0/1:125,9,0:7 0/1:107,0,25:6 0/1:19,0,103:6 0/1:255,0,233:39

5 8687535 . T C 999 . . GT:PL:DP 0/1:65,0,92:23 0/1:47,0,112:33 0/1:43,0,162:44 0/1:25,0,62:4 0/1:7,0,10:2 0/1:42,0,44:17 0/1:49,0,147:50

5 8721587 . C A 999 . . GT:PL:DP 0/1:122,0,255:154 0/1:251,0,255:177 0/1:250,0,255:162 0/1:104,0,163:22 0/1:106,0,158:27 0/1:64,0,149:27 0/1:207,0,255:136

5 8721650 . C T 999 . . GT:PL:DP 0/1:215,0,255:183 0/1:234,0,255:210 0/1:235,0,216:187 0/1:138,0,119:26 0/1:127,0,104:24 0/1:5,0,99:11 0/1:174,0,252:190

5 8722223 . T C 999 . . GT:PL:DP 0/1:232,0,255:207 0/1:225,0,255:220 0/1:255,0,255:220 0/1:191,0,255:94 0/1:235,0,255:146 0/1:196,0,255:209 0/1:165,0,255:210

5 8757170 . G C 999 . . GT:PL:DP 0/1:202,0,151:94 0/1:161,3,0:59 0/1:136,0,55:71 0/1:93,0,95:14 0/1:138,0,120:15 0/1:152,0,6:11 0/1:187,0,82:110

5 8821639 . G A 999 . . GT:PL:DP 0/1:176,0,255:29 0/1:255,0,245:43 0/1:229,0,255:36 0/1:59,0,100:5 0/1:74,0,152:10 0/1:25,0,87:4 0/1:255,0,255:50

5 8854676 . T C 999 . . GT:PL:DP 0/1:161,0,118:14 0/1:132,0,94:11 0/1:119,0,229:17 0/1:34,3,0:1 0/1:16,0,99:5 0/1:52,0,49:5 0/1:187,0,143:13

5 8858575 . C T 999 . . GT:PL:DP 0/1:255,0,199:28 0/1:206,0,255:32 0/1:255,0,192:32 0/1:31,0,31:2 0/1:38,3,0:1 0/1:24,0,104:5 0/1:255,0,255:55

5 8870210 . C T 999 . . GT:PL:DP 0/1:101,0,104:29 0/1:85,0,59:20 0/1:92,0,93:20 0/1:31,0,4:4 0/1:36,0,19:4 0/1:11,0,30:5 0/1:89,0,117:34

5 8876308 . A T 999 . . GT:PL:DP 0/1:163,0,234:23 0/1:107,0,177:20 0/1:134,0,255:40 0/1:7,0,100:5 0/1:30,0,84:5 0/1:54,0,54:5 0/1:135,0,255:39

5 8876472 . A G 999 . . GT:PL:DP 0/1:166,0,88:22 0/1:103,0,193:28 0/1:168,0,216:38 0/1:14,0,46:3 0/1:101,0,32:8 0/1:29,0,33:4 0/1:102,0,210:39

5 9062946 . T G 999 . . GT:PL:DP 0/1:255,0,255:42 0/1:255,0,216:31 0/1:221,0,255:33 0/1:16,0,59:3 0/1:165,0,152:13 0/1:148,0,18:7 0/1:255,0,255:80

5 9064281 . A G 999 . . GT:PL:DP 0/1:255,0,239:62 0/1:233,0,255:40 0/1:119,0,255:57 0/1:27,0,31:2 0/1:163,0,255:21 0/1:235,0,82:18 0/1:255,0,255:101

5 9064284 . C T 999 . . GT:PL:DP 0/1:255,0,255:61 0/1:236,0,255:40 0/1:145,0,255:59 0/1:56,0,28:3 0/1:177,0,255:22 0/1:237,0,90:18 0/1:255,0,255:103

5 9064404 . T A 999 . . GT:PL:DP 0/1:255,0,218:60 0/1:255,0,255:35 0/1:235,0,255:71 0/1:98,0,51:6 0/1:92,0,255:19 0/1:152,0,144:14 0/1:255,0,255:111

5 9064510 . C A 999 . . GT:PL:DP 0/1:255,0,255:87 0/1:255,0,255:42 0/1:255,0,255:76 0/1:25,0,81:4 0/1:160,0,255:24 0/1:129,0,135:10 0/1:207,0,255:151

5 9064628 . C T 999 . . GT:PL:DP 0/1:255,0,255:70 0/1:245,0,255:63 0/1:223,0,255:77 0/1:40,0,114:6 0/1:25,0,242:18 0/1:238,0,150:17 0/1:213,0,255:118

5 9214030 . G A 999 . . GT:PL:DP 0/1:255,0,255:198 0/1:255,0,255:88 0/1:255,0,255:219 0/1:154,0,225:18 0/1:78,0,190:17 0/1:121,0,255:53 0/1:255,0,255:220

5 9298112 . A T 999 . . GT:PL:DP 0/1:181,0,133:83 0/1:226,0,133:116 0/1:255,0,153:119 0/1:88,0,55:14 0/1:126,0,88:41 0/1:68,0,2:28 0/1:247,0,213:98

5 9302456 . G A 999 . . GT:PL:DP 0/1:125,0,194:36 0/1:249,0,159:40 0/1:249,0,195:45 0/1:26,0,49:9 0/1:83,0,41:9 0/1:125,0,118:12 0/1:255,0,237:89

5 9359684 . T G 999 . . GT:PL:DP 0/1:255,0,176:80 0/1:255,0,91:137 0/1:255,0,255:58 0/1:216,0,70:12 0/1:255,0,165:27 0/1:255,0,184:47 0/1:255,0,112:202

5 9389213 . G A 999 . . GT:PL:DP 0/1:255,0,58:56 0/1:255,0,77:65 0/1:255,0,221:60 0/1:47,1,0:5 0/1:63,2,0:8 0/1:96,0,32:23 0/1:255,0,113:122

5 9500178 . T A 999 . . GT:PL:DP 0/1:155,0,209:52 0/1:231,0,35:25 0/1:245,0,171:42 0/1:98,0,61:11 0/1:160,0,30:16 0/1:119,0,29:12 0/1:241,0,116:62

5 10115415 . G C 999 . . GT:PL:DP 0/1:171,0,92:18 0/1:89,0,147:17 0/1:190,0,10:19 0/1:54,0,52:6 0/1:23,6,0:2 0/1:26,0,33:5 0/1:197,0,2:21

5 10221853 . G A 999 . . GT:PL:DP 0/1:155,0,255:28 0/1:224,0,255:39 0/1:154,0,255:31 0/1:21,0,27:3 0/1:53,9,0:3 0/1:7,0,129:5 0/1:164,0,255:40

5 10274676 . C T 999 . . GT:PL:DP 0/1:61,0,74:55 0/1:49,0,115:49 0/1:73,0,49:55 0/1:21,0,32:7 0/1:6,1,0:2 0/1:11,3,0:1 0/1:47,0,101:81

5 10274793 . C T 999 . . GT:PL:DP 0/1:111,0,240:182 0/1:175,0,255:155 0/1:22,0,209:177 0/1:41,0,225:30 0/1:139,0,204:28 0/1:134,0,235:34 0/1:177,0,255:250

5 10274857 . G A 999 . . GT:PL:DP 0/1:255,0,255:172 0/1:255,0,255:147 0/1:241,0,255:181 0/1:168,0,226:35 0/1:213,0,31:28 0/1:232,0,76:54 0/1:255,0,255:160

5 10274858 . G A 999 . . GT:PL:DP 0/1:255,0,255:175 0/1:255,0,255:147 0/1:213,0,255:183 0/1:149,0,237:35 0/1:213,0,56:28 0/1:247,0,88:52 0/1:255,0,255:157

5 10274869 . A C 999 . . GT:PL:DP 0/1:255,0,255:168 0/1:255,0,255:145 0/1:255,0,255:171 0/1:160,0,219:28 0/1:173,0,70:23 0/1:216,0,45:46 0/1:255,0,255:177

5 10297448 . C A 999 . . GT:PL:DP 0/1:99,0,36:9 0/1:49,0,10:7 0/1:60,0,21:15 0/1:13,3,0:1 0/1:16,0,28:5 0/1:42,0,0:4 0/1:40,0,21:8

5 10423780 . T A 999 . . GT:PL:DP 0/1:160,0,221:45 0/1:143,0,223:47 0/1:243,0,177:53 0/1:58,0,25:8 0/1:9,0,96:8 0/1:28,0,101:8 0/1:202,0,213:46

5 10535074 . T A 999 . . GT:PL:DP 0/1:193,0,255:120 0/1:160,0,255:108 0/1:254,0,255:106 0/1:6,0,185:15 0/1:33,0,112:15 0/1:16,0,149:12 0/1:149,0,255:168

5 10535325 . T C 999 . . GT:PL:DP 0/1:128,0,79:64 0/1:222,0,72:58 0/1:138,0,130:67 0/1:122,0,80:12 0/1:26,0,134:17 0/1:19,0,57:4 0/1:62,0,188:131

5 10613925 . A T 999 . . GT:PL:DP 0/1:84,0,79:11 0/1:102,0,56:9 0/1:106,0,114:14 0/1:51,6,0:2 0/1:42,6,0:2 0/1:44,6,0:2 0/1:22,0,103:11

5 10617612 . T G 999 . . GT:PL:DP 0/1:49,0,232:28 0/1:14,0,136:16 0/1:129,0,47:14 0/1:23,0,37:4 0/1:52,12,0:4 0/1:33,3,0:1 0/1:159,0,213:35

5 10667697 . T G 999 . . GT:PL:DP 0/1:64,0,131:29 0/1:218,0,116:30 0/1:228,0,91:32 0/1:19,0,45:6 0/1:84,0,14:9 0/1:90,7,0:7 0/1:255,0,172:100

5 10737642 . T A 999 . . GT:PL:DP 0/1:198,0,2:85 0/1:255,0,160:98 0/1:255,0,115:75 0/1:164,0,59:18 0/1:102,0,58:10 0/1:67,6,0:2 0/1:255,0,231:107

5 10912445 . T C 999 . . GT:PL:DP 0/1:88,0,208:24 0/1:39,0,255:35 0/1:82,0,227:35 0/1:44,0,117:10 0/1:56,0,46:8 0/1:54,0,13:9 0/1:84,0,255:48

5 10982356 . G A 999 . . GT:PL:DP 0/1:29,0,172:13 0/1:176,0,192:31 0/1:96,0,89:13 0/1:49,0,36:3 0/1:32,0,26:4 0/1:71,6,0:2 0/1:191,0,168:21

5 10993368 . T C 999 . . GT:PL:DP 0/1:63,0,16:8 0/1:25,0,99:24 0/1:69,0,23:13 0/1:18,0,21:2 0/1:77,0,114:15 0/1:30,0,109:15 0/1:135,0,60:21

5 10993380 . A G 999 . . GT:PL:DP 0/1:63,0,16:8 0/1:25,0,107:24 0/1:68,0,30:14 0/1:18,0,21:2 0/1:78,0,113:15 0/1:31,0,116:16 0/1:136,0,49:25

5 10993389 . C A 999 . . GT:PL:DP 0/1:81,0,6:12 0/1:33,0,112:24 0/1:62,0,61:18 0/1:18,0,21:2 0/1:77,0,97:14 0/1:30,0,116:15 0/1:143,0,88:28

5 11145949 . A G 999 . . GT:PL:DP 0/1:198,0,206:35 0/1:159,0,205:40 0/1:203,0,156:38 0/1:25,0,55:5 0/1:20,3,0:1 0/1:39,0,96:8 0/1:199,0,253:63

5 11236890 . T C 999 . . GT:PL:DP 0/1:216,0,247:54 0/1:184,0,148:35 0/1:225,0,199:52 0/1:105,0,34:6 0/1:65,0,58:7 0/1:124,0,0:12 0/1:255,0,225:64

5 11400725 . A G 999 . . GT:PL:DP 0/1:255,0,132:45 0/1:255,0,148:34 0/1:255,0,255:59 0/1:78,0,52:5 0/1:126,0,17:7 0/1:243,0,99:18 0/1:255,0,255:111

5 11448984 . A C 999 . . GT:PL:DP 0/1:239,0,255:96 0/1:255,0,255:105 0/1:129,0,255:108 0/1:255,0,254:45 0/1:233,0,194:68 0/1:251,0,255:75 0/1:255,0,255:95

5 11449343 . G A 999 . . GT:PL:DP 0/1:255,0,255:246 0/1:198,0,255:247 0/1:255,0,255:246 0/1:255,0,255:143 0/1:250,0,255:95 0/1:90,0,255:157 0/1:188,0,255:238

5 11641572 . A G 999 . . GT:PL:DP 0/1:40,0,53:16 0/1:65,0,41:14 0/1:18,0,140:19 0/1:42,9,0:3 0/1:32,6,0:2 0/1:21,2,0:3 0/1:44,0,145:37

5 11641936 . C T 999 . . GT:PL:DP 0/1:201,0,208:31 0/1:85,0,219:29 0/1:56,0,255:27 0/1:23,0,48:3 0/1:60,6,0:2 0/1:86,0,43:6 0/1:195,0,252:65

5 11641958 . A C 999 . . GT:PL:DP 0/1:212,0,178:39 0/1:136,0,210:32 0/1:114,0,247:37 0/1:6,0,73:4 0/1:55,1,0:5 0/1:93,0,39:7 0/1:186,0,235:83

5 11772717 . A T 999 . . GT:PL:DP 0/1:255,0,255:184 0/1:255,0,172:208 0/1:255,0,255:221 0/1:255,0,17:48 0/1:255,0,153:36 0/1:255,0,218:42 0/1:255,0,255:188

5 11773080 . C T 999 . . GT:PL:DP 0/1:255,0,255:182 0/1:255,0,43:217 0/1:255,0,255:216 0/1:255,0,249:60 0/1:255,0,130:35 0/1:255,0,255:55 0/1:255,0,255:192

5 11827247 . G C 999 . . GT:PL:DP 0/1:255,0,63:29 0/1:211,0,255:44 0/1:172,0,255:38 0/1:56,0,46:4 0/1:69,0,138:8 0/1:130,0,195:14 0/1:255,0,255:69

5 11827292 . A C 999 . . GT:PL:DP 0/1:17,0,255:30 0/1:159,0,255:43 0/1:193,0,255:34 0/1:68,0,54:5 0/1:113,0,22:5 0/1:235,0,31:13 0/1:255,0,255:74

5 11827539 . A T 999 . . GT:PL:DP 0/1:62,0,179:24 0/1:38,0,111:18 0/1:59,0,99:24 0/1:18,0,43:4 0/1:55,0,18:3 0/1:84,0,42:5 0/1:171,0,92:42

5 11883602 . A T 999 . . GT:PL:DP 0/1:218,0,201:41 0/1:255,0,255:38 0/1:189,0,177:20 0/1:31,0,31:2 0/1:76,6,0:2 0/1:112,0,96:9 0/1:187,0,255:32

5 12036738 . C T 999 . . GT:PL:DP 0/1:132,0,238:145 0/1:201,0,255:155 0/1:161,0,255:164 0/1:42,0,236:27 0/1:146,0,156:78 0/1:145,0,229:56 0/1:180,0,255:164

5 12036806 . T C 999 . . GT:PL:DP 0/1:188,0,255:101 0/1:201,0,255:123 0/1:191,0,255:136 0/1:72,0,172:21 0/1:63,0,121:17 0/1:83,0,117:20 0/1:159,0,255:111

5 12081692 . C A 999 . . GT:PL:DP 0/1:19,9,0:3 0/1:32,0,92:29 0/1:77,0,87:29 0/1:10,0,85:8 0/1:81,9,0:3 0/1:22,0,49:3 0/1:48,0,132:55

5 12315908 . A C 999 . . GT:PL:DP 0/1:228,0,210:227 0/1:255,0,175:214 0/1:181,0,177:208 0/1:98,0,172:35 0/1:90,0,232:105 0/1:146,0,255:185 0/1:201,0,172:231

5 12316890 . G A 999 . . GT:PL:DP 0/1:88,42,33:6 0/1:64,0,53:8 0/1:108,75,69:10 0/1:11,3,0:1 0/1:11,3,0:3 0/1:41,1,0:7 0/1:158,73,68:17

5 12320875 . T C 999 . . GT:PL:DP 0/1:111,0,234:47 0/1:126,0,157:31 0/1:107,0,182:42 0/1:76,0,12:8 0/1:101,0,138:19 0/1:147,6,0:26 0/1:255,0,190:98

5 12330921 . G A 999 . . GT:PL:DP 0/1:53,0,112:25 0/1:15,0,119:31 0/1:62,0,122:21 0/1:42,0,52:5 0/1:11,0,75:6 0/1:13,0,80:6 0/1:130,0,171:42

5 12330950 . G A 999 . . GT:PL:DP 0/1:135,0,143:30 0/1:112,0,126:36 0/1:148,0,131:25 0/1:47,0,62:5 0/1:29,0,104:9 0/1:76,0,116:13 0/1:219,0,132:51

5 12341274 . G A 999 . . GT:PL:DP 0/1:170,0,188:35 0/1:87,0,213:29 0/1:170,0,77:20 0/1:56,0,41:5 0/1:41,0,116:6 0/1:59,0,27:8 0/1:10,0,213:40

5 12341304 . G A 999 . . GT:PL:DP 0/1:191,0,255:32 0/1:128,0,255:35 0/1:255,0,65:24 0/1:56,0,50:5 0/1:75,0,150:10 0/1:63,0,23:8 0/1:65,0,255:42

5 12341471 . T A 999 . . GT:PL:DP 0/1:68,0,160:21 0/1:108,0,180:35 0/1:190,0,59:20 0/1:28,0,33:3 0/1:112,0,50:8 0/1:82,0,19:9 0/1:141,0,214:45

5 12341499 . T A 999 . . GT:PL:DP 0/1:60,0,140:17 0/1:48,0,139:25 0/1:89,2,0:13 0/1:31,0,27:2 0/1:78,0,52:8 0/1:30,0,9:8 0/1:51,0,206:36

5 12351844 . T A 999 . . GT:PL:DP 0/1:255,0,238:237 0/1:255,0,255:249 0/1:255,0,241:231 0/1:255,0,174:159 0/1:23,0,137:75 0/1:88,0,115:62 0/1:255,0,218:219

5 12351862 . A G 999 . . GT:PL:DP 0/1:255,0,237:231 0/1:255,0,255:225 0/1:255,0,244:219 0/1:255,0,209:204 0/1:30,0,96:79 0/1:107,0,105:68 0/1:255,0,238:218

5 12352051 . T C 999 . . GT:PL:DP 0/1:178,0,236:216 0/1:185,0,228:244 0/1:128,0,185:239 0/1:232,0,255:128 0/1:139,0,202:56 0/1:85,0,123:58 0/1:218,0,255:217

5 12352456 . T A 999 . . GT:PL:DP 0/1:237,0,255:217 0/1:234,0,255:221 0/1:250,0,255:211 0/1:191,0,255:157 0/1:200,0,188:90 0/1:165,0,202:60 0/1:127,0,255:201

5 12352986 . C G 999 . . GT:PL:DP 0/1:172,0,255:134 0/1:126,0,236:102 0/1:90,0,210:120 0/1:102,0,143:23 0/1:65,0,20:6 0/1:151,0,196:27 0/1:235,0,251:146

6 57940 . A G 999 . . GT:PL:DP 0/1:255,0,255:219 0/1:239,0,255:231 0/1:166,0,255:235 0/1:179,0,255:89 0/1:222,0,255:86 0/1:183,0,255:63 0/1:228,0,255:231

6 58495 . T A 999 . . GT:PL:DP 0/1:255,0,255:233 0/1:255,0,255:227 0/1:255,0,255:227 0/1:255,0,255:103 0/1:233,0,255:46 0/1:227,0,255:51 0/1:255,0,255:213

6 75561 . A T 112.378 . . GT:PL:DP 0/1:47,0,69:13 0/1:70,18,28:11 0/1:50,0,53:17 0/1:27,3,0:1 0/1:29,18,15:2 0/1:11,0,6:2 0/1:165,145,171:20

6 92450 . T A 999 . . GT:PL:DP 0/1:123,0,84:28 0/1:162,0,88:31 0/1:161,0,124:28 0/1:77,9,0:3 0/1:63,9,0:3 0/1:62,0,23:5 0/1:112,0,85:32

6 98683 . A T 999 . . GT:PL:DP 0/1:142,0,150:22 0/1:77,0,70:11 0/1:16,0,221:25 0/1:73,0,13:5 0/1:20,0,46:3 0/1:54,0,62:8 0/1:106,0,142:23

6 98684 . C A 999 . . GT:PL:DP 0/1:134,0,151:21 0/1:82,0,62:11 0/1:11,0,218:25 0/1:73,0,17:5 0/1:20,0,48:3 0/1:69,0,45:8 0/1:108,0,139:23

6 99262 . A G 999 . . GT:PL:DP 0/1:107,0,126:38 0/1:79,0,106:43 0/1:117,0,100:47 0/1:16,0,32:5 0/1:34,0,0:4 0/1:84,0,94:15 0/1:113,0,81:41

6 105519 . C T 999 . . GT:PL:DP 0/1:175,0,219:147 0/1:145,0,189:133 0/1:213,0,224:132 0/1:67,0,80:19 0/1:161,0,227:48 0/1:246,0,255:137 0/1:156,0,230:148

6 106307 . T A 999 . . GT:PL:DP 0/1:255,0,123:70 0/1:255,0,134:68 0/1:255,0,129:67 0/1:175,0,23:15 0/1:99,0,139:19 0/1:172,0,148:46 0/1:255,0,132:87

6 115579 . C T 999 . . GT:PL:DP 0/1:97,0,69:16 0/1:121,0,70:17 0/1:166,0,2:26 0/1:5,0,20:2 0/1:25,0,10:4 0/1:47,3,0:4 0/1:152,0,85:23

6 117578 . C A 999 . . GT:PL:DP 0/1:73,0,68:28 0/1:71,0,32:10 0/1:89,0,56:18 0/1:105,5,0:6 0/1:27,0,12:2 0/1:25,0,12:2 0/1:56,0,99:25

6 128714 . A T 999 . . GT:PL:DP 0/1:120,2,0:16 0/1:178,0,58:27 0/1:124,0,62:14 0/1:48,9,0:3 0/1:47,6,0:2 0/1:15,0,14:2 0/1:159,11,0:19

6 128974 . A C 999 . . GT:PL:DP 0/1:94,40,32:28 0/1:139,4,0:29 0/1:77,2,0:20 0/1:52,2,0:4 0/1:40,3,0:3 0/1:32,3,0:3 0/1:63,0,19:24

6 128975 . G A 999 . . GT:PL:DP 0/1:98,0,7:29 0/1:117,0,6:33 0/1:62,0,3:22 0/1:50,1,0:4 0/1:35,0,0:4 0/1:30,3,0:3 0/1:66,0,8:24

6 128976 . T C 999 . . GT:PL:DP 0/1:96,0,100:30 0/1:109,0,142:34 0/1:66,0,121:23 0/1:50,0,31:4 0/1:34,0,2:5 0/1:28,2,0:3 0/1:91,0,130:24

6 141272 . T G 999 . . GT:PL:DP 0/1:72,0,255:103 0/1:56,0,255:120 0/1:115,0,255:92 0/1:73,0,191:22 0/1:48,0,137:11 0/1:19,0,152:12 0/1:66,0,255:134

6 141366 . C T 999 . . GT:PL:DP 0/1:92,0,246:139 0/1:90,0,255:145 0/1:112,0,239:150 0/1:59,0,192:28 0/1:119,0,200:32 0/1:91,0,161:32 0/1:41,0,255:198

6 145125 . T C 999 . . GT:PL:DP 0/1:76,0,255:42 0/1:77,0,255:40 0/1:131,0,218:36 0/1:16,0,187:12 0/1:46,0,72:10 0/1:53,0,110:12 0/1:139,0,255:97

6 145236 . T C 999 . . GT:PL:DP 0/1:164,0,255:70 0/1:187,0,255:70 0/1:236,0,205:69 0/1:30,0,150:9 0/1:195,0,106:18 0/1:164,0,148:19 0/1:236,0,255:121

6 145297 . G A 999 . . GT:PL:DP 0/1:186,0,255:75 0/1:216,0,255:58 0/1:216,0,243:71 0/1:44,0,169:12 0/1:124,0,149:31 0/1:106,0,109:13 0/1:245,0,255:132

6 146018 . T A 999 . . GT:PL:DP 0/1:255,0,255:64 0/1:255,0,255:72 0/1:255,0,255:51 0/1:7,0,161:10 0/1:44,0,41:17 0/1:75,0,44:13 0/1:195,0,255:120

6 146991 . G A 999 . . GT:PL:DP 0/1:139,0,255:164 0/1:225,0,216:145 0/1:159,0,255:140 0/1:36,0,127:34 0/1:21,0,57:57 0/1:7,0,100:89 0/1:63,0,221:142

6 164887 . G A 999 . . GT:PL:DP 0/1:82,26,255:36 0/1:62,12,169:47 0/1:115,0,220:49 0/1:48,0,42:7 0/1:13,0,67:17 0/1:66,0,132:29 0/1:99,3,255:142

6 206366 . G A 999 . . GT:PL:DP 0/1:255,0,247:105 0/1:179,0,213:80 0/1:255,0,249:84 0/1:165,0,100:16 0/1:79,0,72:10 0/1:189,0,140:19 0/1:204,0,165:112

6 206398 . A G 999 . . GT:PL:DP 0/1:201,0,255:88 0/1:140,0,255:77 0/1:218,0,255:88 0/1:34,0,150:13 0/1:55,0,90:9 0/1:32,0,223:18 0/1:182,0,255:116

6 213280 . C G 999 . . GT:PL:DP 0/1:255,0,255:96 0/1:242,0,255:57 0/1:166,0,255:65 0/1:9,2,117:10 0/1:70,0,173:16 0/1:40,0,219:19 0/1:253,0,255:122

6 220219 . G C 999 . . GT:PL:DP 0/1:249,0,192:89 0/1:241,0,189:67 0/1:240,0,248:86 0/1:59,0,110:8 0/1:10,0,91:4 0/1:70,0,178:15 0/1:255,0,255:118

6 221354 . A G 999 . . GT:PL:DP 0/1:25,3,0:1 0/1:31,3,0:1 0/1:81,9,0:3 0/1:37,3,0:1 0/1:23,3,0:1 0/1:61,9,0:3 0/1:79,9,0:3

6 225865 . G A 999 . . GT:PL:DP 0/1:32,3,0:1 0/1:64,6,0:2 0/1:65,6,0:2 0/1:37,3,0:1 0/1:34,3,0:1 0/1:31,3,0:1 0/1:15,6,0:2

6 377039 . G A 999 . . GT:PL:DP 0/1:209,0,157:49 0/1:143,0,186:42 0/1:235,0,84:33 0/1:112,0,60:12 0/1:54,6,0:2 0/1:86,0,8:5 0/1:212,0,166:49

6 377118 . T G 999 . . GT:PL:DP 0/1:95,0,255:92 0/1:28,0,243:79 0/1:59,0,241:65 0/1:39,0,120:13 0/1:40,0,133:16 0/1:22,0,143:15 0/1:129,0,255:108

6 377122 . A T 999 . . GT:PL:DP 0/1:115,0,255:93 0/1:71,0,255:79 0/1:90,0,249:66 0/1:42,0,119:13 0/1:57,0,125:16 0/1:31,0,151:16 0/1:132,0,255:108

6 377152 . A G 999 . . GT:PL:DP 0/1:183,0,207:88 0/1:161,0,255:62 0/1:154,0,236:52 0/1:65,0,38:12 0/1:32,0,110:10 0/1:74,0,108:14 0/1:172,0,229:94

6 377172 . C T 999 . . GT:PL:DP 0/1:255,0,133:78 0/1:255,0,89:53 0/1:255,0,102:53 0/1:75,0,23:9 0/1:122,0,21:15 0/1:178,0,26:13 0/1:255,0,164:99

6 377231 . T C 999 . . GT:PL:DP 0/1:129,0,255:73 0/1:75,0,255:56 0/1:123,0,255:58 0/1:112,0,103:15 0/1:74,0,180:20 0/1:49,0,187:14 0/1:170,0,255:108

6 377259 . T C 999 . . GT:PL:DP 0/1:109,0,255:53 0/1:55,0,255:37 0/1:129,0,255:46 0/1:49,0,97:10 0/1:55,0,74:10 0/1:51,0,165:13 0/1:120,0,255:55

6 377444 . C T 999 . . GT:PL:DP 0/1:255,0,224:60 0/1:255,0,217:39 0/1:9,0,255:32 0/1:15,0,96:5 0/1:24,0,100:6 0/1:30,0,56:5 0/1:255,0,255:86

6 377475 . A T 999 . . GT:PL:DP 0/1:180,0,255:59 0/1:208,0,255:47 0/1:255,0,136:38 0/1:21,0,79:5 0/1:98,0,80:10 0/1:55,0,128:10 0/1:217,0,255:88

6 377490 . A T 999 . . GT:PL:DP 0/1:162,0,255:62 0/1:206,0,255:44 0/1:252,0,90:36 0/1:22,0,47:5 0/1:104,0,98:12 0/1:58,0,133:10 0/1:213,0,255:82

6 377497 . A G 999 . . GT:PL:DP 0/1:152,0,255:59 0/1:208,0,255:43 0/1:252,0,150:35 0/1:32,0,50:5 0/1:95,0,108:12 0/1:59,0,149:10 0/1:233,0,255:78

6 388176 . T G 999 . . GT:PL:DP 0/1:175,0,252:53 0/1:229,0,193:41 0/1:59,0,238:43 0/1:22,0,171:12 0/1:13,0,189:16 0/1:87,0,44:7 0/1:222,0,191:59

6 388725 . T C 999 . . GT:PL:DP 0/1:212,0,170:62 0/1:65,0,220:68 0/1:212,0,197:54 0/1:72,0,146:18 0/1:81,0,37:10 0/1:29,0,24:5 0/1:32,0,216:101

6 388781 . G C 999 . . GT:PL:DP 0/1:161,32,206:63 0/1:77,0,208:70 0/1:120,0,203:55 0/1:110,84,151:15 0/1:78,0,54:12 0/1:67,9,0:3 0/1:144,0,224:133

6 388792 . G A 999 . . GT:PL:DP 0/1:140,0,212:45 0/1:38,0,203:66 0/1:110,0,216:49 0/1:23,0,130:11 0/1:60,0,77:11 0/1:67,9,0:3 0/1:163,0,233:123

6 390069 . T C 999 . . GT:PL:DP 0/1:238,0,213:34 0/1:174,0,128:26 0/1:224,0,30:25 0/1:49,0,28:3 0/1:70,0,23:4 0/1:37,8,0:5 0/1:223,0,104:33

6 505236 . A G 999 . . GT:PL:DP 0/1:255,0,255:77 0/1:255,0,234:53 0/1:170,0,255:49 0/1:147,0,118:15 0/1:40,0,188:11 0/1:192,0,175:17 0/1:255,0,255:145

6 539117 . C A 999 . . GT:PL:DP 0/1:180,0,255:218 0/1:180,0,255:227 0/1:219,0,255:224 0/1:131,0,255:56 0/1:180,0,255:76 0/1:144,0,255:146 0/1:147,0,255:238

6 539132 . T C 999 . . GT:PL:DP 0/1:142,0,255:218 0/1:173,0,255:216 0/1:190,0,255:224 0/1:76,0,255:55 0/1:145,0,255:68 0/1:125,0,255:138 0/1:101,0,255:238

6 539529 . G T 999 . . GT:PL:DP 0/1:255,0,255:231 0/1:255,0,167:226 0/1:255,0,221:231 0/1:255,0,131:42 0/1:255,0,194:46 0/1:255,0,255:108 0/1:255,0,255:224

6 540181 . A C 999 . . GT:PL:DP 0/1:209,0,255:141 0/1:223,0,220:145 0/1:209,0,204:130 0/1:139,0,173:26 0/1:47,0,160:27 0/1:130,0,196:44 0/1:196,0,250:138

6 540238 . T G 999 . . GT:PL:DP 0/1:255,0,255:174 0/1:253,0,255:178 0/1:255,0,255:163 0/1:97,0,216:32 0/1:153,0,255:38 0/1:255,0,255:55 0/1:213,0,255:195

6 813851 . T A 999 . . GT:PL:DP 0/1:255,0,16:21 0/1:255,0,107:26 0/1:206,0,46:16 0/1:78,9,0:3 0/1:28,0,23:2 0/1:110,0,39:7 0/1:255,0,79:25

6 813852 . G A 999 . . GT:PL:DP 0/1:240,0,19:20 0/1:255,0,107:26 0/1:212,0,60:16 0/1:98,9,0:3 0/1:25,0,27:3 0/1:110,0,38:7 0/1:255,0,101:25

6 947343 . T G 999 . . GT:PL:DP 0/1:134,0,183:35 0/1:126,0,127:55 0/1:118,0,156:55 0/1:40,0,89:10 0/1:70,0,128:19 0/1:9,0,171:18 0/1:120,0,204:127

6 947390 . C T 999 . . GT:PL:DP 0/1:149,0,148:44 0/1:126,0,123:66 0/1:131,0,146:65 0/1:102,0,110:16 0/1:106,0,77:21 0/1:62,0,39:27 0/1:132,0,197:179

6 947509 . T G 999 . . GT:PL:DP 0/1:255,0,255:58 0/1:255,0,158:81 0/1:255,0,171:65 0/1:133,0,91:12 0/1:255,0,90:49 0/1:244,0,140:111 0/1:255,0,216:182

6 947544 . C T 999 . . GT:PL:DP 0/1:221,0,216:55 0/1:255,0,148:85 0/1:255,0,152:68 0/1:107,0,149:17 0/1:255,0,82:51 0/1:255,0,113:102 0/1:255,0,255:154

6 947545 . G C 999 . . GT:PL:DP 0/1:255,0,207:55 0/1:255,0,143:86 0/1:255,0,136:68 0/1:144,0,121:17 0/1:255,0,60:51 0/1:225,0,52:99 0/1:255,0,206:154

6 947637 . G A 999 . . GT:PL:DP 0/1:89,0,157:27 0/1:113,0,90:36 0/1:127,0,151:43 0/1:57,0,78:13 0/1:20,0,107:20 0/1:36,0,54:40 0/1:124,0,204:47

6 989969 . G T 999 . . GT:PL:DP 0/1:255,0,255:158 0/1:255,0,255:145 0/1:255,0,255:148 0/1:128,0,203:30 0/1:66,0,66:50 0/1:82,0,191:132 0/1:157,0,255:127

6 998492 . C G 999 . . GT:PL:DP 0/1:47,0,115:27 0/1:126,0,43:18 0/1:119,0,82:22 0/1:46,0,14:5 0/1:7,0,59:4 0/1:69,0,83:10 0/1:107,0,122:32

6 1000570 . C T 999 . . GT:PL:DP 0/1:107,0,255:39 0/1:133,0,255:26 0/1:56,0,255:27 0/1:15,0,163:7 0/1:48,0,111:6 0/1:14,0,255:18 0/1:68,0,255:71

6 1000574 . A G 999 . . GT:PL:DP 0/1:126,0,255:40 0/1:121,0,255:25 0/1:59,0,255:26 0/1:15,0,163:7 0/1:51,0,102:6 0/1:23,0,255:18 0/1:77,0,255:69

6 1000708 . T A 999 . . GT:PL:DP 0/1:255,0,255:53 0/1:255,0,218:28 0/1:198,0,255:36 0/1:69,0,138:8 0/1:52,0,188:11 0/1:58,0,154:11 0/1:167,0,255:97

6 1000751 . T C 999 . . GT:PL:DP 0/1:78,0,255:60 0/1:45,0,255:37 0/1:120,0,255:34 0/1:15,0,145:7 0/1:61,0,237:14 0/1:27,0,231:13 0/1:92,0,255:106

6 1000764 . A T 999 . . GT:PL:DP 0/1:255,0,255:60 0/1:255,0,255:40 0/1:217,0,242:38 0/1:33,0,87:5 0/1:151,0,182:15 0/1:100,0,208:13 0/1:255,0,255:101

6 1000832 . G A 999 . . GT:PL:DP 0/1:255,0,255:67 0/1:255,0,255:37 0/1:231,0,255:32 0/1:63,0,118:7 0/1:63,0,81:5 0/1:180,0,114:15 0/1:255,0,255:104

6 1000868 . G T 999 . . GT:PL:DP 0/1:250,0,255:61 0/1:251,0,250:33 0/1:224,0,255:38 0/1:24,0,133:7 0/1:105,0,84:8 0/1:163,0,175:19 0/1:255,0,255:103

6 1000955 . G T 999 . . GT:PL:DP 0/1:175,0,255:72 0/1:219,0,173:39 0/1:139,0,255:34 0/1:66,0,74:7 0/1:67,0,169:12 0/1:59,0,179:13 0/1:225,0,255:120

6 1001016 . T C 999 . . GT:PL:DP 0/1:205,0,255:67 0/1:242,0,245:42 0/1:205,0,255:38 0/1:94,0,56:6 0/1:91,0,53:6 0/1:99,0,110:12 0/1:253,0,255:105

6 1001074 . T C 999 . . GT:PL:DP 0/1:186,0,255:58 0/1:221,0,255:43 0/1:236,0,255:41 0/1:35,0,91:5 0/1:96,0,22:5 0/1:109,0,179:15 0/1:248,0,255:93

6 1001099 . G A 999 . . GT:PL:DP 0/1:178,0,255:57 0/1:199,0,255:43 0/1:233,0,255:40 0/1:25,0,91:5 0/1:49,0,26:3 0/1:67,0,208:14 0/1:255,0,255:103

6 1001163 . A G 999 . . GT:PL:DP 0/1:255,0,182:57 0/1:255,0,233:40 0/1:240,0,252:34 0/1:138,0,59:8 0/1:25,0,96:5 0/1:71,0,132:8 0/1:235,0,255:124

6 1001222 . G A 999 . . GT:PL:DP 0/1:190,0,255:61 0/1:137,0,255:38 0/1:202,0,255:34 0/1:62,0,50:6 0/1:24,0,55:3 0/1:55,0,62:4 0/1:174,0,255:138

6 1140214 . A T 999 . . GT:PL:DP 0/1:227,0,57:34 0/1:249,0,140:36 0/1:228,0,123:40 0/1:40,0,1:4 0/1:15,0,77:9 0/1:66,3,0:5 0/1:255,0,189:67

6 1155167 . A G 999 . . GT:PL:DP 0/1:171,0,157:205 0/1:68,0,4:179 0/1:191,0,239:203 0/1:190,0,128:46 0/1:120,0,7:19 0/1:179,0,35:23 0/1:176,0,214:164

6 1155213 . T C 999 . . GT:PL:DP 0/1:195,0,208:173 0/1:162,0,175:151 0/1:216,0,255:176 0/1:227,0,152:61 0/1:226,0,82:25 0/1:233,0,145:49 0/1:231,0,255:188

6 1155559 . A T 999 . . GT:PL:DP 0/1:255,0,255:192 0/1:255,0,255:173 0/1:255,0,255:193 0/1:255,0,255:86 0/1:255,0,224:57 0/1:255,0,255:56 0/1:255,0,255:187

6 1155743 . G A 999 . . GT:PL:DP 0/1:255,0,154:202 0/1:255,0,162:169 0/1:255,0,229:210 0/1:255,0,161:87 0/1:255,0,141:61 0/1:255,0,206:60 0/1:255,0,255:189

6 1155934 . C T 999 . . GT:PL:DP 0/1:239,0,6:213 0/1:255,0,160:160 0/1:255,0,118:202 0/1:255,0,52:84 0/1:255,0,95:62 0/1:255,0,83:65 0/1:255,0,249:195

6 1349292 . C A 999 . . GT:PL:DP 0/1:255,0,244:31 0/1:207,0,255:32 0/1:255,0,183:35 0/1:90,0,116:7 0/1:73,6,0:2 0/1:107,9,0:3 0/1:255,0,255:46

6 1411998 . G C 999 . . GT:PL:DP 0/1:107,0,85:15 0/1:117,0,167:28 0/1:174,0,139:31 0/1:49,6,0:2 0/1:27,3,0:1 0/1:25,3,0:1 0/1:122,0,185:36

6 1461854 . G C 999 . . GT:PL:DP 0/1:192,0,220:37 0/1:135,0,14:16 0/1:155,0,55:21 0/1:31,0,27:2 0/1:59,6,0:3 0/1:38,9,0:4 0/1:150,0,67:26

6 1462297 . T A 999 . . GT:PL:DP 0/1:246,0,255:144 0/1:140,0,255:82 0/1:255,0,255:94 0/1:61,0,222:18 0/1:255,0,129:35 0/1:100,0,204:29 0/1:255,0,255:178

6 1462405 . C A 999 . . GT:PL:DP 0/1:197,0,255:122 0/1:222,0,240:97 0/1:255,0,195:65 0/1:86,0,190:21 0/1:218,0,232:45 0/1:81,0,227:30 0/1:255,0,189:178

6 1500822 . T C 999 . . GT:PL:DP 0/1:107,0,37:13 0/1:67,0,152:19 0/1:75,0,190:18 0/1:39,0,43:6 0/1:16,6,0:2 0/1:76,9,0:3 0/1:123,0,118:22

6 1504994 . T A 999 . . GT:PL:DP 0/1:50,0,10:4 0/1:75,0,17:5 0/1:65,0,21:10 0/1:14,3,0:1 0/1:42,6,0:2 0/1:44,6,0:2 0/1:42,0,23:6

6 1649634 . C A 999 . . GT:PL:DP 0/1:86,0,216:44 0/1:50,0,183:35 0/1:94,0,203:42 0/1:27,0,100:8 0/1:87,0,10:5 0/1:68,0,207:28 0/1:119,0,242:56

6 1763703 . T C 999 . . GT:PL:DP 0/1:251,6,0:25 0/1:163,0,11:11 0/1:210,6,0:13 0/1:40,6,0:2 0/1:25,3,0:1 0/1:55,6,0:2 0/1:255,0,18:30

6 1990369 . A G 999 . . GT:PL:DP 0/1:124,0,228:32 0/1:255,0,153:34 0/1:170,0,234:30 0/1:88,1,0:4 0/1:35,3,0:1 0/1:52,0,38:4 0/1:251,0,255:39

6 2032500 . T C 999 . . GT:PL:DP 0/1:255,0,108:57 0/1:255,0,73:41 0/1:255,0,50:63 0/1:176,0,13:10 0/1:121,0,63:9 0/1:160,0,95:17 0/1:255,0,199:82

6 2141560 . C T 999 . . GT:PL:DP 0/1:25,0,255:60 0/1:20,0,255:44 0/1:15,0,255:79 0/1:7,0,100:5 0/1:38,0,194:17 0/1:37,0,86:9 0/1:125,0,255:120

6 2152135 . T A 999 . . GT:PL:DP 0/1:105,0,243:155 0/1:82,0,234:130 0/1:43,0,207:136 0/1:126,0,255:29 0/1:62,0,132:22 0/1:80,0,62:26 0/1:11,0,198:199

6 2175292 . G C 999 . . GT:PL:DP 0/1:137,0,44:21 0/1:201,8,0:30 0/1:154,0,18:22 0/1:16,0,28:3 0/1:28,3,0:1 0/1:74,0,13:6 0/1:177,0,130:43

6 2175469 . A G 999 . . GT:PL:DP 0/1:231,0,99:21 0/1:255,0,74:29 0/1:255,0,142:30 0/1:106,0,24:5 0/1:14,0,63:5 0/1:23,0,153:16 0/1:232,0,217:39

6 2316422 . T C 999 . . GT:PL:DP 0/1:214,0,255:112 0/1:195,0,255:200 0/1:255,0,255:133 0/1:102,0,241:21 0/1:128,0,130:12 0/1:136,0,168:19 0/1:191,0,255:234

6 2365629 . G T 999 . . GT:PL:DP 0/1:174,0,193:25 0/1:232,0,255:37 0/1:180,0,200:29 0/1:16,0,59:3 0/1:66,6,0:2 0/1:91,0,42:6 0/1:166,0,255:42

6 2382467 . G A 999 . . GT:PL:DP 0/1:63,0,246:43 0/1:154,0,255:48 0/1:179,0,253:43 0/1:22,0,62:4 0/1:32,0,91:8 0/1:113,0,207:21 0/1:140,0,240:111

6 2408644 . T A 999 . . GT:PL:DP 0/1:193,0,132:26 0/1:17,0,195:30 0/1:66,0,255:28 0/1:48,0,36:4 0/1:7,0,78:7 0/1:96,0,99:10 0/1:222,0,255:55

6 2422385 . C A 999 . . GT:PL:DP 0/1:178,0,90:25 0/1:142,0,200:39 0/1:154,0,255:54 0/1:57,9,0:3 0/1:22,3,0:1 0/1:51,0,80:9 0/1:156,0,255:46

6 2422386 . T G 999 . . GT:PL:DP 0/1:178,0,90:25 0/1:141,0,205:40 0/1:147,0,255:54 0/1:57,9,0:3 0/1:22,3,0:1 0/1:51,0,71:9 0/1:156,0,255:46

6 2422703 . C T 999 . . GT:PL:DP 0/1:255,0,80:43 0/1:255,0,208:43 0/1:255,0,144:39 0/1:84,0,7:4 0/1:113,0,36:10 0/1:72,0,191:14 0/1:255,0,210:69

6 2424415 . C A 999 . . GT:PL:DP 0/1:255,0,255:55 0/1:255,0,255:50 0/1:255,0,225:36 0/1:119,0,115:10 0/1:106,0,8:6 0/1:60,0,96:10 0/1:235,0,255:67

6 2425267 . G A 999 . . GT:PL:DP 0/1:148,0,255:37 0/1:154,0,255:52 0/1:164,0,255:37 0/1:27,0,145:9 0/1:127,0,55:10 0/1:133,0,111:14 0/1:185,0,255:49

6 2425286 . T C 999 . . GT:PL:DP 0/1:132,0,255:35 0/1:135,0,255:51 0/1:127,0,255:39 0/1:27,0,162:9 0/1:155,0,4:9 0/1:105,0,133:14 0/1:149,0,255:52

6 2433901 . T G 999 . . GT:PL:DP 0/1:160,0,213:29 0/1:155,0,245:37 0/1:199,62,171:33 0/1:16,0,98:5 0/1:23,0,16:2 0/1:29,3,0:1 0/1:204,0,255:41

6 2454806 . C A 999 . . GT:PL:DP 0/1:255,0,127:66 0/1:251,0,159:38 0/1:255,0,102:78 0/1:38,0,38:6 0/1:208,0,56:20 0/1:102,0,92:14 0/1:255,0,163:115

6 2786614 . T C 999 . . GT:PL:DP 0/1:191,0,255:220 0/1:199,0,255:241 0/1:255,0,255:230 0/1:184,0,255:108 0/1:133,0,242:66 0/1:184,0,255:134 0/1:98,0,255:225

6 2936978 . A C 999 . . GT:PL:DP 0/1:26,0,121:13 0/1:63,0,110:23 0/1:17,0,106:21 0/1:36,0,22:5 0/1:32,0,0:4 0/1:41,6,0:2 0/1:46,0,134:27

6 3062878 . A G 999 . . GT:PL:DP 0/1:47,0,196:36 0/1:83,0,229:40 0/1:35,0,201:24 0/1:18,0,71:6 0/1:38,0,113:8 0/1:99,0,38:10 0/1:236,0,108:27

6 3284817 . T G 999 . . GT:PL:DP 0/1:224,0,181:108 0/1:245,0,245:114 0/1:212,0,196:136 0/1:252,0,135:30 0/1:45,0,96:15 0/1:84,0,4:9 0/1:255,0,251:227

6 3285888 . T C 999 . . GT:PL:DP 0/1:150,0,232:48 0/1:239,0,70:32 0/1:118,0,137:38 0/1:80,0,8:4 0/1:46,0,31:6 0/1:18,0,96:8 0/1:255,0,184:77

6 3285901 . T C 999 . . GT:PL:DP 0/1:138,0,238:47 0/1:240,0,151:36 0/1:101,0,176:41 0/1:60,0,57:7 0/1:59,0,41:7 0/1:22,0,77:7 0/1:246,0,196:75

6 3286508 . G A 999 . . GT:PL:DP 0/1:255,0,255:137 0/1:251,0,255:183 0/1:255,0,255:186 0/1:132,0,255:31 0/1:239,0,143:36 0/1:185,0,206:43 0/1:255,0,255:224

6 3286614 . C T 999 . . GT:PL:DP 0/1:255,0,255:228 0/1:232,0,255:221 0/1:191,0,255:211 0/1:255,0,255:64 0/1:233,0,255:81 0/1:212,0,255:150 0/1:255,0,255:213

6 3476445 . G C 999 . . GT:PL:DP 0/1:218,0,231:33 0/1:136,0,255:27 0/1:216,0,139:23 0/1:11,0,25:4 0/1:22,0,49:5 0/1:53,0,123:17 0/1:129,0,255:83

6 3476451 . T A 999 . . GT:PL:DP 0/1:219,0,238:33 0/1:131,0,255:27 0/1:227,0,161:22 0/1:11,0,13:4 0/1:24,0,55:5 0/1:55,0,113:16 0/1:130,0,255:81

6 3495957 . C T 999 . . GT:PL:DP 0/1:16,0,241:180 0/1:71,0,255:200 0/1:42,0,245:186 0/1:14,0,205:24 0/1:63,0,124:31 0/1:91,0,181:54 0/1:42,0,236:231

6 3495980 . G A 999 . . GT:PL:DP 0/1:255,0,244:209 0/1:255,0,255:222 0/1:255,0,255:228 0/1:207,0,123:34 0/1:94,0,229:63 0/1:92,0,255:108 0/1:254,0,245:253

6 3496118 . G T 999 . . GT:PL:DP 0/1:166,0,255:170 0/1:152,0,255:185 0/1:121,0,246:174 0/1:62,0,255:33 0/1:18,0,158:38 0/1:69,0,183:46 0/1:112,0,248:203

6 3516609 . G A 999 . . GT:PL:DP 0/1:180,0,252:44 0/1:229,0,255:73 0/1:156,0,253:50 0/1:145,0,37:15 0/1:194,0,74:22 0/1:52,0,83:8 0/1:227,0,255:118

6 3516668 . A T 999 . . GT:PL:DP 0/1:207,0,122:49 0/1:207,0,212:87 0/1:155,0,129:48 0/1:37,0,114:18 0/1:14,0,151:16 0/1:63,0,93:14 0/1:195,0,237:129

6 3516697 . C T 999 . . GT:PL:DP 0/1:73,0,255:51 0/1:204,0,255:93 0/1:129,0,224:46 0/1:95,0,134:17 0/1:117,0,64:15 0/1:59,0,110:12 0/1:109,0,241:115

6 3535690 . T C 999 . . GT:PL:DP 0/1:77,0,7:14 0/1:77,0,57:24 0/1:51,0,163:21 0/1:34,0,44:5 0/1:56,9,0:3 0/1:55,6,0:2 0/1:74,0,50:23

6 3535731 . T A 999 . . GT:PL:DP 0/1:52,0,53:21 0/1:90,0,104:25 0/1:53,0,162:22 0/1:12,0,50:3 0/1:55,6,0:2 0/1:55,6,0:2 0/1:85,0,47:24

6 3591506 . G A 999 . . GT:PL:DP 0/1:179,0,255:107 0/1:201,0,239:98 0/1:221,0,252:150 0/1:89,0,178:29 0/1:35,3,0:5 0/1:4,0,30:3 0/1:104,0,255:117

6 3591634 . A G 999 . . GT:PL:DP 0/1:255,0,255:199 0/1:255,0,255:165 0/1:255,0,255:212 0/1:255,0,255:61 0/1:229,0,216:51 0/1:182,0,170:46 0/1:255,0,255:222

6 3591644 . C G 999 . . GT:PL:DP 0/1:255,0,255:197 0/1:255,0,255:165 0/1:255,0,255:210 0/1:253,0,255:62 0/1:255,0,220:57 0/1:187,0,208:46 0/1:171,0,255:213

6 3591676 . C T 999 . . GT:PL:DP 0/1:255,0,255:196 0/1:255,0,255:174 0/1:255,0,255:201 0/1:255,0,255:64 0/1:255,0,255:55 0/1:234,0,190:33 0/1:255,0,255:209

6 3591793 . A T 999 . . GT:PL:DP 0/1:176,0,246:139 0/1:176,0,242:117 0/1:144,0,255:161 0/1:108,0,176:28 0/1:178,0,183:47 0/1:143,0,207:47 0/1:139,0,255:179

6 3672953 . C G 999 . . GT:PL:DP 0/1:255,0,167:142 0/1:255,0,132:138 0/1:243,0,128:144 0/1:188,0,57:26 0/1:87,0,37:54 0/1:97,0,110:147 0/1:255,0,174:193

6 3674037 . T A 999 . . GT:PL:DP 0/1:139,0,255:65 0/1:140,0,255:56 0/1:59,0,250:76 0/1:17,0,48:10 0/1:37,0,147:11 0/1:162,0,222:30 0/1:177,0,255:143

6 3705786 . G T 999 . . GT:PL:DP 0/1:157,0,155:68 0/1:135,0,255:32 0/1:150,0,158:41 0/1:71,0,14:5 0/1:112,0,41:7 0/1:56,0,29:7 0/1:177,0,172:64

6 3705974 . A G 999 . . GT:PL:DP 0/1:79,0,255:43 0/1:19,0,218:16 0/1:132,0,231:32 0/1:13,0,93:4 0/1:136,0,100:18 0/1:166,0,122:20 0/1:143,0,255:78

6 3705999 . T A 999 . . GT:PL:DP 0/1:128,0,255:107 0/1:53,0,255:46 0/1:156,0,255:85 0/1:28,0,185:10 0/1:198,0,128:24 0/1:224,0,188:35 0/1:118,0,255:168

6 3782579 . G A 999 . . GT:PL:DP 0/1:232,0,193:72 0/1:179,0,156:35 0/1:207,0,72:41 0/1:148,0,123:17 0/1:234,0,21:37 0/1:56,0,15:36 0/1:255,0,63:173

6 3817390 . G C 999 . . GT:PL:DP 0/1:82,0,16:11 0/1:65,0,37:9 0/1:117,0,48:15 0/1:42,6,0:2 0/1:21,3,0:1 0/1:19,0,24:4 0/1:86,22,54:21

6 4100723 . G A 999 . . GT:PL:DP 0/1:88,0,88:40 0/1:54,0,94:27 0/1:88,0,50:18 0/1:35,0,60:8 0/1:42,22,19:2 0/1:46,3,0:3 0/1:177,0,77:37

6 4500196 . A G 999 . . GT:PL:DP 0/1:114,0,85:18 0/1:32,0,99:7 0/1:82,0,109:9 0/1:34,3,0:1 0/1:52,0,23:3 0/1:98,5,0:8 0/1:108,0,48:11

6 4501090 . A C 999 . . GT:PL:DP 0/1:157,0,255:67 0/1:172,0,255:54 0/1:116,0,255:68 0/1:120,0,60:12 0/1:15,0,28:8 0/1:23,0,27:7 0/1:232,0,213:78

6 4634529 . A G 999 . . GT:PL:DP 0/1:98,0,39:19 0/1:156,0,12:34 0/1:92,0,42:19 0/1:36,6,0:2 0/1:17,0,2:3 0/1:15,3,0:1 0/1:95,5,0:18

6 4634530 . T G 999 . . GT:PL:DP 0/1:98,0,39:19 0/1:156,0,9:35 0/1:92,0,42:19 0/1:36,6,0:2 0/1:17,0,2:3 0/1:15,3,0:1 0/1:90,0,0:18

6 4703722 . T A 999 . . GT:PL:DP 0/1:140,0,119:33 0/1:59,0,141:34 0/1:149,0,124:39 0/1:7,0,23:6 0/1:17,0,2:3 0/1:20,0,0:2 0/1:123,0,164:55

6 4777188 . T C 999 . . GT:PL:DP 0/1:247,0,157:32 0/1:255,0,130:24 0/1:255,0,188:47 0/1:67,6,0:2 0/1:255,1,0:16 0/1:219,0,165:17 0/1:255,0,188:88

6 4882151 . C T 999 . . GT:PL:DP 0/1:255,0,152:155 0/1:255,0,255:69 0/1:229,0,67:220 0/1:100,0,190:15 0/1:255,0,143:44 0/1:255,0,126:48 0/1:255,0,198:219

6 4882270 . A G 999 . . GT:PL:DP 0/1:232,0,21:164 0/1:255,0,225:67 0/1:248,0,96:232 0/1:136,0,142:14 0/1:255,0,55:61 0/1:255,0,255:45 0/1:255,0,160:213

6 4882497 . T C 999 . . GT:PL:DP 0/1:255,0,145:192 0/1:255,0,255:81 0/1:255,0,154:223 0/1:205,0,95:14 0/1:255,0,93:42 0/1:255,0,143:45 0/1:255,0,89:223

6 4882642 . A G 999 . . GT:PL:DP 0/1:255,0,167:160 0/1:255,0,255:79 0/1:255,0,103:202 0/1:191,0,146:14 0/1:255,0,64:33 0/1:255,0,149:42 0/1:231,0,53:211

6 4882659 . G A 999 . . GT:PL:DP 0/1:255,0,126:180 0/1:255,0,255:79 0/1:234,0,45:222 0/1:177,0,122:17 0/1:255,0,45:32 0/1:255,0,159:41 0/1:227,0,56:204

6 4882825 . T C 999 . . GT:PL:DP 0/1:255,0,161:137 0/1:255,0,229:65 0/1:207,0,29:194 0/1:105,0,22:5 0/1:255,0,112:32 0/1:255,0,176:43 0/1:247,0,141:216

6 4882924 . A G 999 . . GT:PL:DP 0/1:255,0,227:122 0/1:255,0,233:70 0/1:204,0,31:221 0/1:135,0,43:8 0/1:250,0,129:36 0/1:231,0,186:31 0/1:249,0,183:210

6 4882949 . C T 999 . . GT:PL:DP 0/1:255,0,226:123 0/1:255,0,233:72 0/1:203,0,29:215 0/1:148,0,70:10 0/1:241,0,106:40 0/1:219,0,200:32 0/1:255,0,225:210

6 4882954 . A G 999 . . GT:PL:DP 0/1:255,0,219:123 0/1:255,0,236:73 0/1:210,0,15:218 0/1:145,0,73:9 0/1:254,0,141:49 0/1:227,0,204:37 0/1:255,0,226:211

6 4882963 . T A 999 . . GT:PL:DP 0/1:255,0,229:123 0/1:255,0,255:72 0/1:209,0,6:215 0/1:140,0,95:11 0/1:255,0,109:46 0/1:231,0,228:37 0/1:255,0,255:209

6 4883002 . A G 999 . . GT:PL:DP 0/1:255,0,255:120 0/1:240,0,255:81 0/1:255,0,120:206 0/1:127,0,123:10 0/1:255,0,191:39 0/1:226,0,149:31 0/1:255,0,255:202

6 4883117 . G A 999 . . GT:PL:DP 0/1:220,0,255:86 0/1:132,0,255:72 0/1:255,0,255:165 0/1:66,0,163:10 0/1:255,0,78:27 0/1:223,0,161:25 0/1:255,0,255:180

6 4883155 . C T 999 . . GT:PL:DP 0/1:210,0,255:75 0/1:89,0,255:67 0/1:166,0,255:143 0/1:45,0,167:10 0/1:167,0,81:36 0/1:170,0,156:24 0/1:255,0,255:153

6 4883194 . T C 999 . . GT:PL:DP 0/1:174,0,255:48 0/1:94,0,255:47 0/1:161,0,240:97 0/1:64,0,156:9 0/1:155,0,50:22 0/1:122,0,80:17 0/1:186,0,255:107

6 4918945 . T G 999 . . GT:PL:DP 0/1:120,0,199:63 0/1:73,0,255:79 0/1:255,0,190:55 0/1:36,0,136:11 0/1:18,0,24:18 0/1:30,0,67:34 0/1:211,0,255:104

6 4919538 . A T 999 . . GT:PL:DP 0/1:255,0,255:179 0/1:218,0,255:198 0/1:255,0,255:184 0/1:212,0,108:20 0/1:167,0,157:18 0/1:184,0,80:19 0/1:255,0,255:191

6 4987916 . T A 999 . . GT:PL:DP 0/1:255,0,48:34 0/1:255,0,20:38 0/1:255,0,18:55 0/1:156,0,5:8 0/1:140,0,116:12 0/1:115,0,197:20 0/1:255,0,6:63

6 4988345 . A G 999 . . GT:PL:DP 0/1:255,0,255:72 0/1:210,0,255:72 0/1:253,0,255:71 0/1:43,0,233:14 0/1:96,0,178:12 0/1:56,0,188:11 0/1:184,0,255:89

6 4991376 . A C 999 . . GT:PL:DP 0/1:46,0,6:6 0/1:48,9,0:3 0/1:118,0,15:17 0/1:68,6,0:2 0/1:34,0,3:3 0/1:25,0,6:2 0/1:57,0,66:9

6 4991377 . T C 999 . . GT:PL:DP 0/1:46,0,6:6 0/1:48,9,0:3 0/1:118,0,17:17 0/1:68,6,0:2 0/1:34,0,3:3 0/1:25,0,6:2 0/1:90,0,47:10

6 4999433 . G A 999 . . GT:PL:DP 0/1:138,0,187:29 0/1:212,0,156:29 0/1:27,0,87:16 0/1:11,0,11:3 0/1:6,0,42:9 0/1:23,0,4:3 0/1:47,0,170:31

6 4999434 . C A 999 . . GT:PL:DP 0/1:125,0,188:29 0/1:212,0,154:29 0/1:27,0,87:16 0/1:11,0,2:3 0/1:29,0,43:10 0/1:22,0,4:3 0/1:47,0,170:31

6 5282888 . G C 999 . . GT:PL:DP 0/1:255,0,255:81 0/1:125,0,255:138 0/1:255,0,255:66 0/1:37,3,0:1 0/1:46,0,255:22 0/1:45,0,184:9 0/1:255,0,255:56

6 5282967 . A T 999 . . GT:PL:DP 0/1:255,0,255:71 0/1:132,0,255:157 0/1:215,0,255:76 0/1:84,0,102:7 0/1:21,0,255:17 0/1:27,0,103:7 0/1:221,0,255:64

6 5378369 . C T 999 . . GT:PL:DP 0/1:128,0,255:226 0/1:47,0,177:239 0/1:109,0,234:224 0/1:185,0,255:222 0/1:83,0,211:58 0/1:14,0,255:63 0/1:164,0,255:228

6 5378553 . T A 999 . . GT:PL:DP 0/1:133,0,250:230 0/1:84,0,203:238 0/1:131,0,206:226 0/1:114,0,255:241 0/1:84,0,186:116 0/1:137,0,239:178 0/1:144,0,255:238

6 5378617 . C T 999 . . GT:PL:DP 0/1:88,0,186:235 0/1:74,0,181:240 0/1:94,0,205:229 0/1:45,0,255:244 0/1:64,0,183:84 0/1:98,0,238:145 0/1:195,0,255:239

6 5379417 . A T 999 . . GT:PL:DP 0/1:133,0,178:25 0/1:236,0,138:52 0/1:237,0,96:36 0/1:28,1,0:2 0/1:44,0,24:3 0/1:25,0,28:3 0/1:89,0,41:7

6 5415187 . G A 999 . . GT:PL:DP 0/1:137,0,255:98 0/1:132,0,255:97 0/1:117,0,255:106 0/1:122,0,156:19 0/1:38,0,45:53 0/1:20,0,10:49 0/1:99,0,255:145

6 5559951 . T G 999 . . GT:PL:DP 0/1:255,0,255:214 0/1:255,0,255:155 0/1:255,0,255:179 0/1:235,0,255:39 0/1:219,0,93:44 0/1:41,0,112:18 0/1:255,0,255:185

6 5611901 . G T 999 . . GT:PL:DP 0/1:233,0,161:62 0/1:184,0,188:65 0/1:157,0,164:66 0/1:124,0,102:12 0/1:83,0,137:12 0/1:94,0,57:24 0/1:210,0,255:57

6 5618184 . G C 999 . . GT:PL:DP 0/1:118,0,85:18 0/1:135,0,45:12 0/1:141,0,1:7 0/1:46,0,13:3 0/1:78,0,21:8 0/1:73,0,8:5 0/1:23,0,185:36

6 5715802 . C T 999 . . GT:PL:DP 0/1:153,0,155:57 0/1:22,0,155:35 0/1:166,0,188:47 0/1:53,0,63:7 0/1:26,0,25:2 0/1:46,0,32:5 0/1:181,0,253:107

6 5716429 . T C 999 . . GT:PL:DP 0/1:255,0,255:108 0/1:200,0,255:68 0/1:255,0,189:57 0/1:122,0,72:10 0/1:26,0,79:4 0/1:178,0,58:15 0/1:39,0,255:141

6 5872169 . G A 999 . . GT:PL:DP 0/1:157,0,211:18 0/1:219,0,196:21 0/1:255,0,186:54 0/1:115,0,78:9 0/1:108,0,27:5 0/1:169,0,8:12 0/1:255,0,255:79

6 5918704 . T A 999 . . GT:PL:DP 0/1:68,0,149:27 0/1:103,0,156:24 0/1:88,0,209:44 0/1:34,0,82:8 0/1:11,0,45:4 0/1:23,3,0:1 0/1:120,0,229:31

6 5921271 . G A 999 . . GT:PL:DP 0/1:58,0,117:25 0/1:65,0,58:32 0/1:31,0,102:39 0/1:52,2,0:4 0/1:26,0,23:4 0/1:27,1,0:2 0/1:64,0,149:77

6 5930195 . A T 999 . . GT:PL:DP 0/1:132,0,255:28 0/1:214,0,255:32 0/1:165,0,255:37 0/1:10,0,153:7 0/1:34,3,0:1 0/1:55,0,44:6 0/1:119,0,255:35

6 5983519 . A C 999 . . GT:PL:DP 0/1:44,0,52:10 0/1:30,0,112:11 0/1:91,0,68:16 0/1:46,0,64:5 0/1:34,9,0:3 0/1:9,3,0:1 0/1:23,0,56:9

6 6067186 . C A 999 . . GT:PL:DP 0/1:220,0,7:217 0/1:255,0,192:214 0/1:255,0,149:225 0/1:255,0,149:183 0/1:255,0,156:164 0/1:255,0,243:161 0/1:255,0,224:227

6 6140182 . A G 999 . . GT:PL:DP 0/1:165,0,62:15 0/1:171,0,150:24 0/1:161,0,129:19 0/1:16,0,19:2 0/1:79,9,0:3 0/1:62,6,0:2 0/1:161,0,73:19

6 6158025 . T C 999 . . GT:PL:DP 0/1:104,0,179:25 0/1:55,0,164:21 0/1:35,0,254:28 0/1:30,3,0:1 0/1:36,9,0:3 0/1:32,6,0:2 0/1:72,0,243:29

6 6544935 . A G 999 . . GT:PL:DP 0/1:37,3,0:1 0/1:64,0,16:3 0/1:66,6,0:2 0/1:67,6,0:2 0/1:47,6,0:2 0/1:55,6,0:2 0/1:76,31,45:5

6 6579426 . C T 999 . . GT:PL:DP 0/1:224,0,252:121 0/1:207,0,255:138 0/1:251,0,255:127 0/1:93,0,123:17 0/1:18,0,49:11 0/1:57,0,20:18 0/1:241,0,255:200

6 6579463 . C T 999 . . GT:PL:DP 0/1:203,0,255:209 0/1:201,0,255:202 0/1:255,0,255:199 0/1:36,0,193:35 0/1:84,0,61:19 0/1:46,0,98:31 0/1:255,0,255:211

6 6579474 . A G 999 . . GT:PL:DP 0/1:133,0,255:215 0/1:200,0,255:204 0/1:255,0,255:198 0/1:78,0,198:39 0/1:53,0,101:32 0/1:83,0,103:48 0/1:255,0,255:219

6 6623150 . C G 999 . . GT:PL:DP 0/1:255,0,255:208 0/1:255,0,255:216 0/1:216,0,255:206 0/1:155,0,255:42 0/1:234,0,255:57 0/1:223,0,232:50 0/1:83,0,255:202

6 6623443 . A T 999 . . GT:PL:DP 0/1:162,0,239:69 0/1:121,0,255:55 0/1:226,0,255:109 0/1:143,0,74:13 0/1:92,0,149:17 0/1:92,0,175:17 0/1:255,0,255:106

6 6684873 . C A 999 . . GT:PL:DP 0/1:70,0,15:4 0/1:40,0,39:4 0/1:70,0,12:5 0/1:28,3,0:1 0/1:37,6,0:2 0/1:26,3,0:1 0/1:104,12,0:4

6 6684874 . A G 999 . . GT:PL:DP 0/1:70,0,15:4 0/1:40,0,54:5 0/1:70,0,12:5 0/1:28,3,0:1 0/1:37,6,0:2 0/1:26,3,0:1 0/1:90,0,12:5

6 6762292 . C T 999 . . GT:PL:DP 0/1:90,0,15:22 0/1:76,0,70:19 0/1:20,0,142:13 0/1:35,0,10:3 0/1:104,0,8:7 0/1:20,0,38:3 0/1:24,0,181:29

6 6764624 . C T 999 . . GT:PL:DP 0/1:245,0,230:114 0/1:255,0,111:108 0/1:236,0,255:107 0/1:165,0,169:29 0/1:35,0,103:51 0/1:22,0,133:77 0/1:250,0,206:178

6 6764672 . C A 999 . . GT:PL:DP 0/1:250,0,225:98 0/1:253,0,143:101 0/1:182,0,255:98 0/1:145,0,134:24 0/1:31,0,63:44 0/1:59,0,53:56 0/1:255,0,225:131

6 6780281 . A G 999 . . GT:PL:DP 0/1:165,0,255:157 0/1:122,0,218:154 0/1:141,0,255:147 0/1:151,0,161:97 0/1:164,0,165:26 0/1:54,0,158:27 0/1:221,0,238:143

6 7036884 . A G 999 . . GT:PL:DP 0/1:129,0,180:25 0/1:123,0,15:9 0/1:140,0,150:23 0/1:95,0,47:8 0/1:47,6,0:2 0/1:69,9,0:3 0/1:231,9,0:25

6 7183975 . G T 999 . . GT:PL:DP 0/1:160,0,221:31 0/1:215,0,198:23 0/1:193,0,255:34 0/1:68,0,71:6 0/1:97,9,0:3 0/1:48,6,0:2 0/1:244,0,206:30

6 7184592 . T G 999 . . GT:PL:DP 0/1:255,0,237:34 0/1:255,0,234:31 0/1:231,0,255:29 0/1:81,0,25:4 0/1:70,6,0:2 0/1:15,0,175:8 0/1:255,0,255:61

6 7192887 . C T 999 . . GT:PL:DP 0/1:255,0,250:30 0/1:255,0,181:35 0/1:255,0,255:44 0/1:37,3,0:1 0/1:66,6,0:2 0/1:35,3,0:1 0/1:255,0,255:51

6 7248065 . A G 999 . . GT:PL:DP 0/1:163,0,125:18 0/1:186,0,150:26 0/1:201,0,133:27 0/1:81,9,0:3 0/1:77,9,0:3 0/1:39,6,0:2 0/1:138,0,255:36

6 7256244 . A G 999 . . GT:PL:DP 0/1:57,0,72:10 0/1:117,0,109:19 0/1:93,0,116:17 0/1:22,0,41:5 0/1:18,3,0:1 0/1:20,6,0:2 0/1:177,0,120:37

6 7565274 . T A 999 . . GT:PL:DP 0/1:97,0,50:14 0/1:127,0,19:17 0/1:84,0,26:20 0/1:31,0,11:3 0/1:31,0,31:5 0/1:18,0,10:2 0/1:121,0,39:27

6 7657426 . A T 999 . . GT:PL:DP 0/1:238,0,159:30 0/1:91,0,214:24 0/1:160,0,233:37 0/1:76,9,0:3 0/1:55,6,0:2 0/1:20,0,43:3 0/1:247,0,208:45

6 7670722 . C T 999 . . GT:PL:DP 0/1:147,0,139:47 0/1:161,0,187:83 0/1:105,0,203:97 0/1:25,0,92:15 0/1:38,0,98:13 0/1:85,0,98:16 0/1:140,0,213:151

6 7670754 . T C 999 . . GT:PL:DP 0/1:142,0,131:56 0/1:151,0,157:85 0/1:138,0,150:105 0/1:27,0,106:16 0/1:14,0,122:19 0/1:44,2,0:23 0/1:155,0,185:159

6 7705015 . G A 999 . . GT:PL:DP 0/1:102,0,84:29 0/1:100,0,158:25 0/1:138,0,110:38 0/1:22,0,21:2 0/1:43,0,119:9 0/1:32,0,77:11 0/1:112,0,142:24

6 7705260 . C A 999 . . GT:PL:DP 0/1:231,0,255:105 0/1:163,3,226:65 0/1:210,0,212:78 0/1:98,63,134:12 0/1:56,0,253:21 0/1:140,0,244:35 0/1:208,0,245:112

6 7705287 . T C 999 . . GT:PL:DP 0/1:242,0,255:99 0/1:137,0,255:61 0/1:209,0,255:83 0/1:47,0,181:14 0/1:62,0,255:29 0/1:113,0,255:48 0/1:194,0,255:113

6 7705321 . G A 999 . . GT:PL:DP 0/1:212,0,255:107 0/1:102,0,255:77 0/1:199,0,255:92 0/1:36,0,168:14 0/1:48,0,227:31 0/1:88,0,255:42 0/1:168,0,255:127

6 7705358 . A T 999 . . GT:PL:DP 0/1:190,0,255:117 0/1:71,0,245:82 0/1:156,0,255:104 0/1:56,0,176:14 0/1:39,0,203:31 0/1:59,0,254:43 0/1:169,0,255:128

6 7705563 . A T 999 . . GT:PL:DP 0/1:105,0,208:48 0/1:126,0,183:56 0/1:129,0,213:82 0/1:25,0,70:7 0/1:113,0,115:22 0/1:106,0,132:24 0/1:132,0,158:53

6 7705570 . A C 999 . . GT:PL:DP 0/1:99,0,216:48 0/1:177,0,201:54 0/1:136,0,205:81 0/1:14,0,100:9 0/1:87,0,189:23 0/1:30,0,189:26 0/1:119,0,189:57

6 7705764 . C T 999 . . GT:PL:DP 0/1:154,0,137:61 0/1:130,0,197:58 0/1:147,0,204:64 0/1:11,0,80:10 0/1:15,0,97:10 0/1:57,0,90:11 0/1:88,0,196:30

6 7705769 . A G 999 . . GT:PL:DP 0/1:154,0,149:64 0/1:129,0,199:63 0/1:146,0,204:65 0/1:17,0,70:10 0/1:26,0,97:10 0/1:68,0,104:13 0/1:89,0,199:31

6 7705778 . T A 999 . . GT:PL:DP 0/1:130,0,157:68 0/1:116,0,198:63 0/1:130,0,195:72 0/1:15,0,73:11 0/1:39,0,88:11 0/1:56,0,117:14 0/1:77,0,197:32

6 7706130 . T C 999 . . GT:PL:DP 0/1:202,0,211:68 0/1:161,0,220:64 0/1:144,0,246:80 0/1:117,3,0:13 0/1:27,0,90:7 0/1:7,0,64:7 0/1:226,0,248:78

6 7723016 . T G 999 . . GT:PL:DP 0/1:49,0,103:13 0/1:42,0,112:10 0/1:27,0,69:11 0/1:11,0,65:5 0/1:54,0,25:6 0/1:71,0,63:10 0/1:119,0,96:30

6 7930632 . C T 999 . . GT:PL:DP 0/1:158,0,255:219 0/1:166,0,255:201 0/1:255,0,255:225 0/1:79,0,255:46 0/1:181,0,255:83 0/1:169,0,255:149 0/1:210,0,255:229

6 8069449 . T A 999 . . GT:PL:DP 0/1:44,0,109:13 0/1:115,0,200:31 0/1:89,0,194:33 0/1:14,3,0:1 0/1:25,3,0:1 0/1:91,0,37:7 0/1:32,0,159:34

6 8160243 . T C 999 . . GT:PL:DP 0/1:210,0,140:20 0/1:145,0,242:34 0/1:193,0,226:30 0/1:22,3,0:1 0/1:64,9,0:3 0/1:65,5,0:4 0/1:249,0,232:44

6 8217509 . T C 999 . . GT:PL:DP 0/1:221,0,181:26 0/1:111,0,236:19 0/1:181,0,253:24 0/1:11,0,54:3 0/1:76,9,0:3 0/1:42,18,12:3 0/1:90,0,255:29

6 8252679 . A T 999 . . GT:PL:DP 0/1:255,0,255:230 0/1:248,0,255:137 0/1:229,0,255:186 0/1:131,0,255:54 0/1:164,0,145:25 0/1:72,0,192:21 0/1:142,0,255:223

6 8298130 . T A 999 . . GT:PL:DP 0/1:67,0,108:69 0/1:84,0,110:42 0/1:120,0,96:48 0/1:9,0,109:11 0/1:14,0,34:4 0/1:15,0,53:9 0/1:92,0,144:101

6 8337552 . C G 999 . . GT:PL:DP 0/1:104,0,82:14 0/1:93,0,74:14 0/1:66,0,107:17 0/1:36,6,0:2 0/1:46,9,0:3 0/1:27,0,44:4 0/1:51,0,98:11

6 8492301 . T C 999 . . GT:PL:DP 0/1:156,0,255:211 0/1:171,0,255:202 0/1:156,0,255:223 0/1:158,0,94:37 0/1:55,0,94:13 0/1:106,0,101:12 0/1:183,0,255:218

6 8492370 . G A 999 . . GT:PL:DP 0/1:133,0,255:187 0/1:121,0,255:193 0/1:140,0,255:193 0/1:197,0,129:41 0/1:95,0,91:14 0/1:91,0,32:13 0/1:196,0,255:199

6 8492451 . T C 999 . . GT:PL:DP 0/1:189,0,255:202 0/1:137,0,255:209 0/1:221,0,255:214 0/1:255,0,255:62 0/1:121,0,143:12 0/1:162,0,151:25 0/1:255,0,255:216

6 8511419 . A C 999 . . GT:PL:DP 0/1:255,0,255:194 0/1:255,0,255:183 0/1:255,0,255:206 0/1:231,0,224:43 0/1:129,0,7:16 0/1:214,0,107:66 0/1:255,0,255:201

6 8511466 . C T 999 . . GT:PL:DP 0/1:255,0,229:178 0/1:255,0,176:140 0/1:255,0,213:162 0/1:255,0,123:38 0/1:146,0,20:16 0/1:213,0,125:77 0/1:255,0,241:190

6 8511476 . A G 999 . . GT:PL:DP 0/1:255,0,255:166 0/1:255,0,255:135 0/1:237,0,255:151 0/1:179,0,139:33 0/1:34,0,60:10 0/1:135,0,183:51 0/1:255,0,255:188

6 8511627 . C T 999 . . GT:PL:DP 0/1:162,0,255:254 0/1:131,0,255:248 0/1:104,0,255:263 0/1:150,0,253:81 0/1:255,0,152:50 0/1:255,0,255:98 0/1:116,0,255:248

6 8594558 . T G 999 . . GT:PL:DP 0/1:255,0,255:106 0/1:255,0,255:90 0/1:255,0,255:200 0/1:202,0,255:29 0/1:104,0,179:13 0/1:212,0,234:22 0/1:255,0,255:120

6 8594575 . T C 999 . . GT:PL:DP 0/1:255,0,255:107 0/1:255,0,255:85 0/1:255,0,255:187 0/1:255,0,220:32 0/1:180,0,96:12 0/1:236,0,242:25 0/1:255,0,255:125

6 8594644 . A G 999 . . GT:PL:DP 0/1:255,0,255:121 0/1:255,0,255:97 0/1:255,0,255:172 0/1:255,0,255:33 0/1:92,0,86:8 0/1:179,0,142:15 0/1:255,0,255:139

6 8648744 . G A 999 . . GT:PL:DP 0/1:107,0,255:23 0/1:69,0,255:30 0/1:199,0,245:25 0/1:67,0,44:7 0/1:36,3,0:1 0/1:92,9,0:3 0/1:181,0,210:25

6 8648909 . T A 999 . . GT:PL:DP 0/1:226,0,255:31 0/1:133,0,255:29 0/1:255,0,255:35 0/1:167,0,8:7 0/1:99,9,0:3 0/1:71,6,0:2 0/1:255,0,255:47

6 8726273 . A G 999 . . GT:PL:DP 0/1:195,0,255:53 0/1:216,0,209:47 0/1:87,0,255:56 0/1:191,0,122:14 0/1:56,0,152:27 0/1:30,0,211:29 0/1:249,0,255:154

6 8726326 . T C 999 . . GT:PL:DP 0/1:240,0,255:45 0/1:174,0,255:47 0/1:99,0,255:63 0/1:155,0,132:12 0/1:130,0,255:29 0/1:93,0,255:28 0/1:255,0,255:180

6 8966487 . A T 999 . . GT:PL:DP 0/1:127,0,18:21 0/1:134,0,10:17 0/1:119,0,28:12 0/1:13,0,42:4 0/1:17,0,17:5 0/1:16,0,43:8 0/1:109,0,98:34

6 8967200 . A T 999 . . GT:PL:DP 0/1:175,0,227:43 0/1:255,2,0:28 0/1:234,0,11:46 0/1:73,0,87:7 0/1:56,0,51:9 0/1:93,0,36:7 0/1:254,0,22:79

6 9041996 . T C 999 . . GT:PL:DP 0/1:179,0,172:192 0/1:112,0,200:166 0/1:136,0,178:173 0/1:127,0,141:34 0/1:122,0,106:30 0/1:8,0,33:26 0/1:118,0,172:254

6 9042341 . G C 999 . . GT:PL:DP 0/1:255,0,255:225 0/1:255,0,255:214 0/1:239,0,255:227 0/1:182,0,221:95 0/1:242,0,255:72 0/1:255,0,255:112 0/1:236,0,255:236

6 9088638 . A G 999 . . GT:PL:DP 0/1:187,0,204:34 0/1:254,0,214:44 0/1:254,0,21:24 0/1:89,3,0:5 0/1:96,9,0:3 0/1:83,0,9:5 0/1:226,0,211:53

6 9143061 . A C 999 . . GT:PL:DP 0/1:214,0,106:26 0/1:246,0,108:32 0/1:101,0,77:14 0/1:24,0,15:4 0/1:88,9,0:3 0/1:24,0,20:2 0/1:193,0,34:26

6 9143087 . T C 999 . . GT:PL:DP 0/1:196,0,96:31 0/1:180,0,122:28 0/1:142,0,103:21 0/1:29,0,15:4 0/1:58,6,0:2 0/1:21,0,33:3 0/1:177,0,125:31

6 9152369 . G A 999 . . GT:PL:DP 0/1:235,0,255:234 0/1:165,0,255:222 0/1:110,0,228:234 0/1:200,0,255:91 0/1:136,0,255:42 0/1:143,0,255:51 0/1:204,0,255:217

6 9152390 . T C 999 . . GT:PL:DP 0/1:213,0,255:214 0/1:101,0,255:235 0/1:102,0,255:229 0/1:140,0,255:90 0/1:144,0,255:37 0/1:65,0,255:56 0/1:161,0,255:218

6 9152425 . G A 999 . . GT:PL:DP 0/1:186,0,255:239 0/1:172,0,255:236 0/1:158,0,255:231 0/1:194,0,255:99 0/1:111,0,255:44 0/1:153,0,255:66 0/1:145,0,255:228

6 9152449 . A G 999 . . GT:PL:DP 0/1:148,0,255:224 0/1:188,0,255:230 0/1:77,0,255:220 0/1:178,0,255:89 0/1:132,0,210:43 0/1:145,0,255:59 0/1:125,0,255:232

6 9152499 . G A 999 . . GT:PL:DP 0/1:140,0,255:225 0/1:157,0,255:220 0/1:118,0,255:225 0/1:133,0,255:108 0/1:116,0,234:50 0/1:10,0,252:45 0/1:76,0,255:229

6 9152512 . C T 999 . . GT:PL:DP 0/1:162,0,255:227 0/1:174,0,255:219 0/1:170,0,255:220 0/1:147,0,255:110 0/1:126,0,250:59 0/1:40,0,253:52 0/1:115,0,255:217

6 9152561 . T G 999 . . GT:PL:DP 0/1:231,0,255:216 0/1:153,0,255:226 0/1:194,0,255:219 0/1:198,0,255:129 0/1:149,0,246:92 0/1:141,0,255:85 0/1:140,0,255:224

6 9152784 . A T 999 . . GT:PL:DP 0/1:118,0,255:218 0/1:160,0,255:231 0/1:75,0,255:227 0/1:118,0,255:132 0/1:86,0,255:100 0/1:217,0,255:79 0/1:116,0,255:221

6 9152797 . C A 999 . . GT:PL:DP 0/1:255,0,255:221 0/1:255,0,255:223 0/1:255,0,255:235 0/1:255,0,229:125 0/1:255,0,194:97 0/1:255,0,255:77 0/1:255,0,255:223

6 9152848 . T A 999 . . GT:PL:DP 0/1:163,0,255:208 0/1:241,0,255:204 0/1:174,0,255:226 0/1:122,0,226:90 0/1:103,0,255:78 0/1:149,0,255:59 0/1:229,0,255:202

6 9183213 . T A 999 . . GT:PL:DP 0/1:130,0,255:207 0/1:255,0,255:222 0/1:255,0,255:203 0/1:113,0,255:29 0/1:23,0,255:27 0/1:129,0,255:24 0/1:156,0,255:234

6 9215702 . T A 999 . . GT:PL:DP 0/1:186,0,118:208 0/1:184,0,125:184 0/1:228,0,180:211 0/1:134,0,255:63 0/1:92,0,68:43 0/1:10,0,76:41 0/1:207,0,224:212

6 9215922 . A G 999 . . GT:PL:DP 0/1:67,0,255:225 0/1:193,0,255:229 0/1:196,0,255:235 0/1:178,0,255:53 0/1:255,0,219:85 0/1:255,0,96:113 0/1:255,0,255:223

6 9307121 . T G 999 . . GT:PL:DP 0/1:255,0,128:30 0/1:239,0,36:23 0/1:255,0,93:24 0/1:202,0,71:19 0/1:255,0,39:20 0/1:113,0,38:6 0/1:255,0,145:52

6 9427428 . A C 999 . . GT:PL:DP 0/1:245,0,34:211 0/1:255,0,141:201 0/1:247,0,16:222 0/1:255,0,255:159 0/1:189,0,104:120 0/1:186,0,129:143 0/1:255,0,255:208

6 9427436 . A G 999 . . GT:PL:DP 0/1:255,0,231:204 0/1:255,0,154:205 0/1:255,0,110:221 0/1:255,0,255:166 0/1:234,0,80:118 0/1:243,0,210:156 0/1:255,0,255:207

6 9594566 . A T 999 . . GT:PL:DP 0/1:125,0,200:66 0/1:14,0,237:54 0/1:94,0,103:56 0/1:32,0,27:6 0/1:32,0,30:10 0/1:9,0,70:5 0/1:77,0,195:89

6 9594579 . C A 999 . . GT:PL:DP 0/1:128,0,184:68 0/1:15,0,236:53 0/1:96,0,103:54 0/1:42,0,27:6 0/1:42,0,30:10 0/1:11,0,72:5 0/1:88,0,181:84

6 9594848 . A G 999 . . GT:PL:DP 0/1:76,0,165:37 0/1:88,0,157:45 0/1:242,0,115:47 0/1:68,0,25:7 0/1:7,0,50:11 0/1:24,0,151:18 0/1:155,0,115:90

6 9661019 . G A 999 . . GT:PL:DP 0/1:228,0,11:21 0/1:165,0,36:13 0/1:141,0,160:15 0/1:25,3,0:1 0/1:73,6,0:2 0/1:36,0,36:2 0/1:255,0,219:30

6 9722700 . G A 999 . . GT:PL:DP 0/1:112,0,127:32 0/1:107,0,33:16 0/1:74,0,114:24 0/1:34,6,0:2 0/1:19,0,89:5 0/1:14,0,105:8 0/1:101,0,200:44

6 9828735 . T C 999 . . GT:PL:DP 0/1:175,0,255:216 0/1:204,0,255:231 0/1:255,0,255:239 0/1:235,0,255:112 0/1:71,0,255:75 0/1:134,0,255:81 0/1:139,0,255:232

6 10089513 . G A 999 . . GT:PL:DP 0/1:139,0,169:25 0/1:100,0,180:33 0/1:48,0,208:24 0/1:14,0,24:2 0/1:50,0,35:4 0/1:16,0,114:9 0/1:84,0,91:21

6 10089847 . A T 999 . . GT:PL:DP 0/1:96,0,255:38 0/1:230,0,255:32 0/1:255,0,75:33 0/1:48,0,47:5 0/1:9,0,32:5 0/1:32,0,73:7 0/1:255,0,42:40

6 10092929 . A G 999 . . GT:PL:DP 0/1:255,0,255:111 0/1:234,0,255:110 0/1:177,0,255:107 0/1:162,0,121:17 0/1:70,0,101:77 0/1:20,0,146:45 0/1:144,0,255:159

6 10191642 . C T 999 . . GT:PL:DP 0/1:70,0,104:12 0/1:96,0,47:13 0/1:10,0,114:9 0/1:29,5,3:3 0/1:38,6,0:2 0/1:12,3,0:1 0/1:6,0,110:8

6 10320850 . G A 999 . . GT:PL:DP 0/1:255,0,255:42 0/1:255,0,145:23 0/1:19,0,255:25 0/1:25,0,91:4 0/1:33,3,0:1 0/1:120,0,255:19 0/1:255,0,255:58

6 10515442 . A G 999 . . GT:PL:DP 0/1:255,0,255:218 0/1:255,0,255:201 0/1:255,0,255:199 0/1:255,0,131:53 0/1:255,0,114:114 0/1:255,0,130:185 0/1:255,0,169:220

6 10520597 . C A 999 . . GT:PL:DP 0/1:159,6,0:22 0/1:204,0,44:28 0/1:91,0,104:21 0/1:12,3,0:1 0/1:26,0,18:3 0/1:40,6,0:2 0/1:202,4,0:31

6 10544819 . G C 999 . . GT:PL:DP 0/1:142,0,49:12 0/1:255,0,83:40 0/1:188,0,72:17 0/1:105,0,38:9 0/1:63,0,114:10 0/1:23,0,49:7 0/1:255,0,91:51

6 10584980 . C T 999 . . GT:PL:DP 0/1:140,0,255:32 0/1:201,0,255:34 0/1:252,0,197:26 0/1:95,0,41:6 0/1:77,9,0:3 0/1:93,9,0:3 0/1:255,0,255:45

6 10621961 . T C 999 . . GT:PL:DP 0/1:87,0,140:12 0/1:83,0,202:26 0/1:106,0,154:19 0/1:20,3,0:1 0/1:34,6,0:2 0/1:51,0,60:5 0/1:110,0,146:19

6 10722730 . T C 999 . . GT:PL:DP 0/1:62,0,239:95 0/1:46,0,255:65 0/1:128,0,217:86 0/1:50,0,130:12 0/1:158,0,136:16 0/1:166,0,197:23 0/1:255,0,255:139

6 10722908 . G A 999 . . GT:PL:DP 0/1:63,0,255:77 0/1:247,0,255:91 0/1:233,0,255:136 0/1:20,0,232:18 0/1:44,0,233:19 0/1:68,0,226:32 0/1:255,0,255:129

6 10722919 . T G 999 . . GT:PL:DP 0/1:88,0,255:79 0/1:226,0,255:89 0/1:241,0,255:142 0/1:20,0,228:18 0/1:41,0,210:20 0/1:80,0,236:34 0/1:255,0,255:111

6 10723140 . C A 999 . . GT:PL:DP 0/1:213,0,216:48 0/1:188,0,255:78 0/1:255,0,255:133 0/1:135,0,55:13 0/1:103,0,207:18 0/1:153,0,102:11 0/1:255,0,255:125

6 10871463 . G A 999 . . GT:PL:DP 0/1:99,0,69:37 0/1:51,0,157:48 0/1:68,0,131:50 0/1:53,0,20:8 0/1:15,0,21:7 0/1:61,0,110:18 0/1:65,0,141:68

6 10900887 . C T 999 . . GT:PL:DP 0/1:255,0,232:175 0/1:255,0,107:97 0/1:255,0,219:153 0/1:143,0,150:48 0/1:139,0,196:44 0/1:180,0,119:48 0/1:255,0,255:278

6 10941902 . T A 999 . . GT:PL:DP 0/1:255,0,255:187 0/1:255,0,255:181 0/1:255,0,255:166 0/1:82,0,255:31 0/1:86,0,233:87 0/1:82,0,255:142 0/1:211,0,255:151

6 10941909 . T A 999 . . GT:PL:DP 0/1:233,0,255:188 0/1:135,0,255:180 0/1:242,0,255:170 0/1:81,0,255:32 0/1:91,0,216:85 0/1:81,0,255:138 0/1:104,0,255:160

6 10959275 . T A 999 . . GT:PL:DP 0/1:255,0,255:92 0/1:255,0,255:91 0/1:255,0,253:91 0/1:135,0,79:13 0/1:42,0,43:12 0/1:26,0,81:16 0/1:255,0,255:177

6 10964689 . T C 999 . . GT:PL:DP 0/1:126,0,255:222 0/1:247,0,255:237 0/1:78,0,255:239 0/1:94,0,252:172 0/1:195,0,255:64 0/1:178,0,255:135 0/1:171,0,255:235

6 10964705 . C A 999 . . GT:PL:DP 0/1:255,0,255:220 0/1:255,0,255:237 0/1:245,0,255:241 0/1:255,0,255:171 0/1:249,0,255:70 0/1:254,0,255:132 0/1:255,0,255:234

6 10964774 . G A 999 . . GT:PL:DP 0/1:255,0,255:220 0/1:255,0,255:234 0/1:255,0,255:232 0/1:255,0,255:166 0/1:255,0,255:74 0/1:255,0,255:72 0/1:255,0,255:234

6 10989418 . A C 999 . . GT:PL:DP 0/1:74,0,50:8 0/1:69,0,96:15 0/1:74,0,44:6 0/1:16,3,0:1 0/1:15,0,57:3 0/1:20,0,11:2 0/1:64,0,84:9

6 10989421 . T C 999 . . GT:PL:DP 0/1:96,0,18:7 0/1:140,0,88:19 0/1:74,0,36:6 0/1:16,3,0:1 0/1:15,0,57:3 0/1:20,0,11:2 0/1:80,0,93:10

6 11039995 . C T 999 . . GT:PL:DP 0/1:124,0,73:16 0/1:171,0,63:30 0/1:68,0,55:18 0/1:31,0,16:3 0/1:35,9,0:3 0/1:12,3,0:1 0/1:103,0,99:24

6 11050231 . C T 999 . . GT:PL:DP 0/1:176,0,255:74 0/1:35,0,229:39 0/1:219,0,216:36 0/1:93,0,124:15 0/1:53,0,72:9 0/1:77,0,167:16 0/1:255,0,255:124

6 11083092 . T A 999 . . GT:PL:DP 0/1:146,0,255:64 0/1:49,0,250:60 0/1:32,0,255:60 0/1:56,0,224:22 0/1:97,0,171:27 0/1:163,0,255:61 0/1:154,0,255:92

6 11100082 . G A 999 . . GT:PL:DP 0/1:30,0,240:66 0/1:79,0,237:62 0/1:12,0,205:56 0/1:69,0,143:15 0/1:13,0,43:6 0/1:7,0,33:23 0/1:100,0,255:108

6 11100110 . C T 999 . . GT:PL:DP 0/1:250,0,183:73 0/1:183,0,240:64 0/1:176,0,208:65 0/1:82,0,129:18 0/1:40,0,31:6 0/1:73,0,46:22 0/1:157,0,255:117

6 11100172 . A G 999 . . GT:PL:DP 0/1:124,0,215:55 0/1:28,0,197:47 0/1:37,0,219:43 0/1:36,0,52:9 0/1:44,0,26:4 0/1:56,0,104:13 0/1:9,0,175:99

6 11100186 . T A 999 . . GT:PL:DP 0/1:125,0,249:61 0/1:26,0,218:50 0/1:41,0,243:42 0/1:27,0,83:12 0/1:51,0,26:4 0/1:56,0,104:13 0/1:10,0,197:98

6 11152153 . G A 999 . . GT:PL:DP 0/1:116,0,210:29 0/1:164,0,182:22 0/1:66,0,138:19 0/1:22,0,108:5 0/1:29,0,86:6 0/1:58,0,154:12 0/1:222,0,255:76

6 11195105 . A C 999 . . GT:PL:DP 0/1:255,0,23:30 0/1:255,0,32:30 0/1:206,0,15:19 0/1:53,0,7:4 0/1:88,5,0:6 0/1:18,0,29:3 0/1:255,0,105:49

6 11252236 . G A 999 . . GT:PL:DP 0/1:157,0,144:90 0/1:138,0,119:84 0/1:123,0,130:83 0/1:24,0,35:14 0/1:80,47,39:20 0/1:62,8,0:27 0/1:159,0,174:142

6 11253470 . C G 999 . . GT:PL:DP 0/1:152,0,219:61 0/1:123,0,238:50 0/1:123,0,206:49 0/1:82,0,107:23 0/1:64,0,45:10 0/1:97,0,90:17 0/1:176,0,255:114

6 11253516 . T A 999 . . GT:PL:DP 0/1:152,0,255:85 0/1:110,0,255:78 0/1:76,0,237:79 0/1:99,0,132:30 0/1:38,0,56:8 0/1:90,0,38:12 0/1:174,0,255:165

6 11264941 . G A 999 . . GT:PL:DP 0/1:246,0,255:258 0/1:224,0,255:255 0/1:255,0,255:289 0/1:255,0,255:245 0/1:221,0,255:179 0/1:255,0,255:172 0/1:255,0,255:240

6 11362168 . C G 999 . . GT:PL:DP 0/1:102,0,181:117 0/1:133,0,224:149 0/1:111,0,211:114 0/1:58,0,107:17 0/1:58,0,248:26 0/1:81,0,181:25 0/1:101,0,232:179

6 11362205 . C T 999 . . GT:PL:DP 0/1:27,0,137:130 0/1:100,0,250:188 0/1:196,0,239:149 0/1:99,0,128:29 0/1:150,0,220:24 0/1:129,0,218:29 0/1:65,0,250:212

6 11362281 . T G 999 . . GT:PL:DP 0/1:255,0,255:130 0/1:255,0,255:138 0/1:255,0,255:144 0/1:171,0,90:25 0/1:11,0,117:8 0/1:119,0,121:19 0/1:255,0,255:159

6 11362467 . T C 999 . . GT:PL:DP 0/1:209,0,255:203 0/1:167,0,255:207 0/1:214,0,255:194 0/1:148,0,255:39 0/1:220,0,255:60 0/1:123,0,255:49 0/1:217,0,255:212

6 11398961 . G A 999 . . GT:PL:DP 0/1:212,0,255:244 0/1:146,0,255:243 0/1:163,0,255:241 0/1:255,0,255:120 0/1:238,0,255:195 0/1:255,0,255:214 0/1:255,0,255:228

6 11417063 . A T 999 . . GT:PL:DP 0/1:108,0,255:118 0/1:148,0,245:60 0/1:122,0,255:75 0/1:93,0,147:13 0/1:8,0,0:3 0/1:47,3,0:3 0/1:149,0,255:142

6 11479643 . A T 999 . . GT:PL:DP 0/1:222,99,255:48 0/1:117,0,198:27 0/1:129,28,255:28 0/1:22,0,113:5 0/1:43,0,138:8 0/1:119,0,255:29 0/1:255,0,255:85

6 11479656 . A T 999 . . GT:PL:DP 0/1:255,0,242:51 0/1:78,0,171:26 0/1:149,0,255:33 0/1:7,0,94:4 0/1:50,0,145:8 0/1:143,0,255:30 0/1:255,0,255:59

6 11488449 . A T 999 . . GT:PL:DP 0/1:237,0,255:72 0/1:123,0,255:94 0/1:183,0,255:46 0/1:171,0,52:10 0/1:41,0,67:6 0/1:159,0,195:17 0/1:171,0,255:130

6 11488492 . A G 999 . . GT:PL:DP 0/1:232,0,255:73 0/1:76,0,255:113 0/1:81,0,255:47 0/1:142,0,83:10 0/1:53,0,73:4 0/1:130,0,165:11 0/1:145,0,255:158

6 11488850 . C T 999 . . GT:PL:DP 0/1:134,0,255:59 0/1:174,0,255:95 0/1:154,0,255:66 0/1:74,0,112:13 0/1:22,0,79:5 0/1:159,0,92:17 0/1:123,0,234:84

6 11545490 . C A 999 . . GT:PL:DP 0/1:195,0,255:153 0/1:255,0,134:166 0/1:232,0,230:133 0/1:221,0,107:37 0/1:77,5,0:13 0/1:49,0,128:18 0/1:255,0,239:192

6 11547094 . G A 999 . . GT:PL:DP 0/1:204,0,255:39 0/1:255,0,255:36 0/1:255,0,248:38 0/1:85,0,159:11 0/1:73,9,0:3 0/1:61,0,81:6 0/1:250,0,255:34

6 11576766 . C A 999 . . GT:PL:DP 0/1:134,0,81:238 0/1:111,0,60:237 0/1:145,0,100:233 0/1:255,0,205:245 0/1:169,0,83:226 0/1:112,0,55:205 0/1:154,0,120:220

6 11576909 . G A 999 . . GT:PL:DP 0/1:223,0,255:206 0/1:215,0,255:215 0/1:255,0,255:205 0/1:223,0,255:112 0/1:221,0,196:91 0/1:124,0,169:69 0/1:255,0,255:210

6 11619068 . C G 999 . . GT:PL:DP 0/1:206,0,124:21 0/1:157,0,255:27 0/1:119,0,255:28 0/1:78,6,0:2 0/1:69,6,0:2 0/1:58,9,0:3 0/1:255,0,255:48

6 11667307 . G C 999 . . GT:PL:DP 0/1:27,0,255:222 0/1:21,0,255:240 0/1:162,0,255:236 0/1:119,0,255:203 0/1:240,0,255:182 0/1:255,0,255:194 0/1:16,0,255:236

6 11667782 . G A 999 . . GT:PL:DP 0/1:255,0,115:204 0/1:255,0,169:137 0/1:255,0,183:167 0/1:255,0,197:43 0/1:152,0,89:22 0/1:37,0,69:12 0/1:255,0,195:127

6 11667788 . T A 999 . . GT:PL:DP 0/1:255,0,91:214 0/1:255,0,141:152 0/1:255,0,135:185 0/1:255,0,178:42 0/1:150,0,57:20 0/1:48,0,91:12 0/1:255,0,171:138

6 11731089 . A G 999 . . GT:PL:DP 0/1:255,0,255:170 0/1:191,0,255:153 0/1:206,0,255:165 0/1:45,0,188:16 0/1:37,0,205:44 0/1:79,0,255:44 0/1:15,0,255:149

6 11731115 . T A 999 . . GT:PL:DP 0/1:77,0,255:129 0/1:78,0,232:115 0/1:94,0,243:134 0/1:38,0,155:13 0/1:40,0,165:37 0/1:33,0,182:44 0/1:255,0,255:129

6 11752778 . G T 999 . . GT:PL:DP 0/1:34,0,255:73 0/1:202,0,242:37 0/1:49,0,255:46 0/1:29,0,115:6 0/1:25,0,70:4 0/1:97,0,235:23 0/1:194,0,255:158

6 11754018 . T G 999 . . GT:PL:DP 0/1:238,0,255:69 0/1:255,0,202:34 0/1:72,0,255:73 0/1:59,6,0:2 0/1:47,0,113:6 0/1:181,0,133:18 0/1:255,0,255:137

7 3985 . T C 999 . . GT:PL:DP 0/1:179,0,255:112 0/1:192,0,255:130 0/1:199,0,255:138 0/1:81,0,207:28 0/1:7,0,26:3 0/1:43,0,70:10 0/1:240,0,255:144

7 4323 . C T 999 . . GT:PL:DP 0/1:255,0,255:138 0/1:255,0,219:143 0/1:255,0,159:122 0/1:255,0,57:39 0/1:11,0,13:15 0/1:24,5,44:8 0/1:255,0,238:145

7 15677 . T C 999 . . GT:PL:DP 0/1:0,0,0:0 0/1:0,0,0:0 0/1:92,92,92:3 0/1:0,0,0:0 0/1:0,0,0:0 0/1:0,0,0:0 0/1:0,0,0:0

7 27960 . A G 999 . . GT:PL:DP 0/1:201,0,248:44 0/1:179,0,212:31 0/1:217,0,210:28 0/1:90,0,37:6 0/1:20,0,54:6 0/1:21,0,45:3 0/1:150,0,255:44

7 94080 . A G 999 . . GT:PL:DP 0/1:95,0,217:28 0/1:42,0,188:29 0/1:79,0,183:25 0/1:37,3,0:1 0/1:36,9,0:3 0/1:33,3,0:1 0/1:102,0,222:38

7 194112 . G A 999 . . GT:PL:DP 0/1:225,0,116:29 0/1:197,0,219:36 0/1:182,0,255:32 0/1:89,0,7:5 0/1:58,0,15:4 0/1:18,3,0:1 0/1:227,0,227:52

7 194117 . A T 999 . . GT:PL:DP 0/1:219,0,153:31 0/1:206,0,207:35 0/1:180,0,255:33 0/1:80,3,0:5 0/1:58,0,15:4 0/1:18,3,0:1 0/1:235,0,211:51

7 194130 . T C 999 . . GT:PL:DP 0/1:217,0,177:30 0/1:214,0,200:33 0/1:191,0,255:34 0/1:75,0,7:5 0/1:61,0,15:4 0/1:21,3,0:1 0/1:247,0,200:54

7 199260 . C T 999 . . GT:PL:DP 0/1:178,0,226:69 0/1:190,0,164:98 0/1:119,0,158:43 0/1:101,0,39:10 0/1:66,0,103:16 0/1:92,0,140:18 0/1:251,0,255:66

7 372507 . G A 999 . . GT:PL:DP 0/1:88,0,251:37 0/1:227,0,139:43 0/1:164,0,179:43 0/1:16,0,144:9 0/1:59,0,103:29 0/1:42,0,90:85 0/1:255,0,255:106

7 372542 . C G 999 . . GT:PL:DP 0/1:172,0,255:50 0/1:255,0,200:51 0/1:232,0,255:44 0/1:42,0,164:11 0/1:158,0,99:27 0/1:255,0,176:79 0/1:255,0,255:118

7 372611 . C T 999 . . GT:PL:DP 0/1:174,0,255:48 0/1:255,0,160:37 0/1:250,0,238:36 0/1:63,0,137:9 0/1:179,0,82:32 0/1:255,0,243:67 0/1:255,0,255:103

7 372629 . C T 999 . . GT:PL:DP 0/1:230,0,255:35 0/1:255,0,26:29 0/1:136,0,196:32 0/1:63,0,123:9 0/1:100,0,123:29 0/1:229,0,154:47 0/1:255,0,121:92

7 463669 . T A 999 . . GT:PL:DP 0/1:62,0,161:14 0/1:32,0,108:15 0/1:145,0,101:15 0/1:68,9,0:3 0/1:7,0,8:2 0/1:14,3,0:1 0/1:174,0,16:45

7 494532 . G A 999 . . GT:PL:DP 0/1:120,0,255:224 0/1:178,0,255:210 0/1:121,0,231:231 0/1:119,0,249:53 0/1:87,0,255:72 0/1:255,0,255:98 0/1:65,0,176:263

7 494646 . T A 999 . . GT:PL:DP 0/1:255,0,255:125 0/1:255,0,255:129 0/1:255,0,212:147 0/1:234,0,215:54 0/1:9,0,200:28 0/1:133,0,255:56 0/1:255,0,255:201

7 495556 . G C 999 . . GT:PL:DP 0/1:255,0,255:187 0/1:255,0,255:172 0/1:193,0,255:177 0/1:122,0,255:65 0/1:108,0,212:53 0/1:131,0,255:183 0/1:255,0,255:194

7 510768 . A G 999 . . GT:PL:DP 0/1:97,0,113:16 0/1:109,0,155:19 0/1:48,0,133:20 0/1:22,0,52:3 0/1:34,0,45:6 0/1:15,0,15:8 0/1:115,0,162:19

7 560710 . G A 999 . . GT:PL:DP 0/1:86,41,152:60 0/1:53,0,111:150 0/1:126,0,110:141 0/1:38,0,119:19 0/1:56,0,31:31 0/1:14,0,46:44 0/1:86,0,142:166

7 564630 . G A 999 . . GT:PL:DP 0/1:228,0,197:31 0/1:118,0,255:42 0/1:188,0,242:36 0/1:42,0,55:5 0/1:39,0,103:10 0/1:34,6,0:2 0/1:198,0,232:39

7 583054 . G A 999 . . GT:PL:DP 0/1:132,0,173:35 0/1:139,0,222:43 0/1:96,0,241:37 0/1:12,0,67:5 0/1:60,0,90:14 0/1:82,0,151:15 0/1:178,0,237:85

7 626844 . T C 999 . . GT:PL:DP 0/1:158,0,73:20 0/1:115,0,199:25 0/1:175,0,61:12 0/1:26,0,25:3 0/1:6,0,35:3 0/1:7,0,52:3 0/1:206,0,48:31

7 713218 . G T 999 . . GT:PL:DP 0/1:93,0,212:68 0/1:40,0,108:55 0/1:36,0,15:30 0/1:109,0,120:16 0/1:19,0,22:3 0/1:13,1,0:5 0/1:148,0,99:83

7 713299 . A T 999 . . GT:PL:DP 0/1:125,0,255:74 0/1:154,0,255:58 0/1:189,0,253:47 0/1:28,0,246:16 0/1:28,0,49:16 0/1:39,0,137:15 0/1:220,0,255:110

7 720677 . T C 999 . . GT:PL:DP 0/1:133,0,203:34 0/1:93,0,120:24 0/1:133,0,140:28 0/1:77,9,0:3 0/1:56,0,12:4 0/1:15,0,22:3 0/1:199,0,97:50

7 815963 . A C 999 . . GT:PL:DP 0/1:245,0,255:38 0/1:255,0,255:40 0/1:255,0,255:52 0/1:67,6,0:2 0/1:42,6,0:2 0/1:48,0,166:8 0/1:255,0,255:51

7 816127 . C T 999 . . GT:PL:DP 0/1:255,0,255:43 0/1:255,0,254:35 0/1:255,0,223:28 0/1:128,0,26:7 0/1:186,0,62:11 0/1:28,0,43:4 0/1:255,0,255:56

7 816258 . G T 999 . . GT:PL:DP 0/1:236,0,255:31 0/1:237,0,242:29 0/1:243,0,255:36 0/1:79,9,0:3 0/1:90,0,101:9 0/1:6,0,83:4 0/1:252,0,255:43

7 840726 . G A 999 . . GT:PL:DP 0/1:189,0,255:259 0/1:255,0,255:257 0/1:255,0,255:245 0/1:241,0,255:125 0/1:29,0,82:7 0/1:215,0,255:56 0/1:234,0,255:243

7 841370 . T C 999 . . GT:PL:DP 0/1:121,0,255:247 0/1:126,0,255:243 0/1:83,0,255:242 0/1:151,0,255:213 0/1:185,0,255:153 0/1:60,0,251:173 0/1:69,0,255:246

7 841381 . G C 999 . . GT:PL:DP 0/1:205,0,255:242 0/1:193,0,255:238 0/1:96,0,255:239 0/1:203,0,255:208 0/1:209,0,255:141 0/1:108,0,255:182 0/1:191,0,255:247

7 841437 . A C 999 . . GT:PL:DP 0/1:255,0,255:235 0/1:255,0,255:230 0/1:246,0,255:225 0/1:244,0,255:152 0/1:255,0,255:69 0/1:255,0,255:115 0/1:178,0,255:237

7 916355 . T C 999 . . GT:PL:DP 0/1:235,0,255:139 0/1:255,0,255:158 0/1:255,0,255:131 0/1:251,0,188:31 0/1:136,0,164:52 0/1:172,0,160:88 0/1:140,0,255:252

7 916433 . T A 999 . . GT:PL:DP 0/1:255,0,255:189 0/1:255,0,255:194 0/1:255,0,255:182 0/1:255,0,232:43 0/1:198,0,155:64 0/1:235,0,179:105 0/1:255,0,255:233

7 982383 . C A 999 . . GT:PL:DP 0/1:255,0,255:229 0/1:255,0,255:212 0/1:255,0,255:221 0/1:255,0,241:67 0/1:111,0,166:73 0/1:99,0,127:67 0/1:255,0,255:216

7 998707 . G T 999 . . GT:PL:DP 0/1:155,0,177:37 0/1:132,0,127:18 0/1:132,0,181:27 0/1:39,0,18:5 0/1:22,0,80:4 0/1:10,3,0:1 0/1:173,0,236:32

7 1034263 . C T 999 . . GT:PL:DP 0/1:255,0,255:140 0/1:255,0,255:183 0/1:255,0,255:154 0/1:151,0,148:27 0/1:107,4,0:49 0/1:20,0,40:93 0/1:255,0,255:197

7 1395319 . A G 999 . . GT:PL:DP 0/1:22,0,206:211 0/1:31,0,244:202 0/1:91,0,252:202 0/1:13,0,243:22 0/1:67,0,192:17 0/1:122,0,159:31 0/1:108,0,255:161

7 1395806 . A G 999 . . GT:PL:DP 0/1:90,0,89:110 0/1:72,0,101:110 0/1:95,0,143:141 0/1:48,0,58:26 0/1:7,0,71:21 0/1:72,0,2:39 0/1:115,0,150:129

7 1408464 . G C 999 . . GT:PL:DP 0/1:127,0,255:184 0/1:96,0,255:148 0/1:36,0,255:177 0/1:95,0,168:27 0/1:52,0,179:17 0/1:127,0,255:37 0/1:128,0,255:205

7 1408474 . C G 999 . . GT:PL:DP 0/1:132,0,255:185 0/1:105,0,255:140 0/1:39,0,255:171 0/1:115,0,113:23 0/1:57,0,182:18 0/1:129,0,255:34 0/1:127,0,255:208

7 1408479 . A G 999 . . GT:PL:DP 0/1:135,0,255:190 0/1:116,0,255:144 0/1:38,0,255:172 0/1:121,0,113:24 0/1:64,0,219:19 0/1:134,0,255:35 0/1:128,0,255:213

7 1408482 . A T 999 . . GT:PL:DP 0/1:138,0,255:191 0/1:120,0,255:143 0/1:64,0,255:170 0/1:121,0,113:24 0/1:64,0,222:19 0/1:134,0,255:35 0/1:130,0,255:214

7 1408499 . G A 999 . . GT:PL:DP 0/1:145,0,255:173 0/1:126,0,255:134 0/1:90,0,255:168 0/1:123,0,122:26 0/1:71,0,202:19 0/1:135,0,232:31 0/1:132,0,255:222

7 1408533 . T C 999 . . GT:PL:DP 0/1:138,0,255:169 0/1:108,0,255:143 0/1:78,0,255:157 0/1:101,0,82:28 0/1:119,0,255:26 0/1:159,0,233:44 0/1:132,0,255:209

7 1408548 . T G 999 . . GT:PL:DP 0/1:135,0,255:152 0/1:107,0,255:138 0/1:85,0,255:155 0/1:102,0,67:26 0/1:107,0,247:24 0/1:153,0,209:40 0/1:134,0,255:175

7 1408557 . G A 999 . . GT:PL:DP 0/1:132,0,177:142 0/1:104,0,255:135 0/1:81,0,255:146 0/1:102,0,61:26 0/1:107,0,206:24 0/1:153,0,182:39 0/1:139,0,255:159

7 1408559 . C T 999 . . GT:PL:DP 0/1:144,0,188:133 0/1:97,0,255:129 0/1:80,0,255:138 0/1:102,0,61:26 0/1:108,0,202:26 0/1:144,0,172:40 0/1:137,0,255:158

7 1408562 . T G 999 . . GT:PL:DP 0/1:145,0,190:129 0/1:100,0,255:127 0/1:74,0,255:135 0/1:104,0,71:25 0/1:108,0,203:26 0/1:143,0,184:41 0/1:138,0,255:155

7 1408563 . A G 999 . . GT:PL:DP 0/1:145,0,190:129 0/1:103,0,255:125 0/1:72,0,255:135 0/1:104,0,71:25 0/1:108,0,203:26 0/1:144,0,174:41 0/1:139,0,255:154

7 1408592 . G A 999 . . GT:PL:DP 0/1:98,0,141:107 0/1:99,0,255:99 0/1:105,0,255:115 0/1:82,0,64:19 0/1:68,0,122:22 0/1:130,0,167:43 0/1:140,0,255:102

7 1408623 . A G 999 . . GT:PL:DP 0/1:52,0,61:75 0/1:75,0,206:79 0/1:61,0,238:92 0/1:67,0,64:16 0/1:36,0,67:10 0/1:49,0,104:21 0/1:118,0,247:62

7 1408632 . A G 999 . . GT:PL:DP 0/1:62,0,87:42 0/1:26,0,171:67 0/1:11,0,163:65 0/1:55,0,60:15 0/1:23,0,68:9 0/1:18,0,95:19 0/1:57,0,233:46

7 1580358 . G C 999 . . GT:PL:DP 0/1:42,5,255:138 0/1:39,0,238:120 0/1:13,0,217:192 0/1:29,3,187:32 0/1:27,0,217:25 0/1:138,0,255:52 0/1:52,0,241:182

7 1580600 . T A 999 . . GT:PL:DP 0/1:197,0,247:142 0/1:155,0,248:200 0/1:189,0,255:188 0/1:108,0,204:53 0/1:25,0,176:21 0/1:138,0,135:41 0/1:168,0,255:179

7 1580658 . A G 999 . . GT:PL:DP 0/1:169,0,150:95 0/1:139,0,187:140 0/1:187,0,151:103 0/1:79,0,135:36 0/1:61,33,95:12 0/1:114,28,31:21 0/1:164,0,223:79

7 1601926 . A G 999 . . GT:PL:DP 0/1:50,0,154:14 0/1:109,0,81:13 0/1:80,0,130:16 0/1:32,0,28:3 0/1:61,0,28:3 0/1:75,0,30:4 0/1:15,0,158:12

7 1604368 . T C 999 . . GT:PL:DP 0/1:63,0,255:62 0/1:64,0,255:58 0/1:249,0,255:69 0/1:112,0,232:23 0/1:152,0,231:26 0/1:73,0,255:31 0/1:189,0,255:173

7 1604471 . T A 999 . . GT:PL:DP 0/1:253,0,143:54 0/1:249,0,178:55 0/1:255,0,155:68 0/1:23,0,124:17 0/1:141,0,70:13 0/1:144,0,207:29 0/1:234,0,255:150

7 1624134 . A T 999 . . GT:PL:DP 0/1:255,0,81:61 0/1:255,0,69:49 0/1:255,0,37:49 0/1:83,0,9:5 0/1:133,0,25:11 0/1:114,0,39:18 0/1:244,0,202:85

7 1624221 . T C 999 . . GT:PL:DP 0/1:255,0,93:73 0/1:255,0,89:68 0/1:255,0,80:63 0/1:115,0,21:8 0/1:108,0,81:12 0/1:98,0,116:17 0/1:181,0,236:92

7 1624398 . A T 999 . . GT:PL:DP 0/1:250,0,49:45 0/1:242,0,18:68 0/1:222,0,13:62 0/1:31,0,34:6 0/1:43,0,17:5 0/1:115,0,64:15 0/1:184,0,255:86

7 1624487 . G T 999 . . GT:PL:DP 0/1:255,0,85:54 0/1:255,0,64:74 0/1:255,0,19:50 0/1:115,0,45:11 0/1:52,0,108:11 0/1:32,0,196:19 0/1:187,0,255:88

7 1625424 . G A 999 . . GT:PL:DP 0/1:255,0,62:135 0/1:255,0,255:154 0/1:255,0,255:205 0/1:213,0,99:31 0/1:220,0,140:36 0/1:255,0,79:28 0/1:255,0,48:172

7 1625448 . T C 999 . . GT:PL:DP 0/1:255,0,42:127 0/1:255,0,255:143 0/1:255,0,201:218 0/1:162,0,183:34 0/1:163,0,152:30 0/1:191,0,224:34 0/1:255,0,255:167

7 1625465 . T C 999 . . GT:PL:DP 0/1:255,0,28:124 0/1:255,0,255:137 0/1:255,0,191:215 0/1:142,0,190:35 0/1:159,0,144:33 0/1:132,0,236:32 0/1:255,0,255:160

7 1625790 . A G 999 . . GT:PL:DP 0/1:255,0,174:206 0/1:255,0,255:221 0/1:255,0,152:238 0/1:186,0,251:58 0/1:215,0,119:46 0/1:123,0,200:36 0/1:180,0,255:225

7 1625834 . G T 999 . . GT:PL:DP 0/1:255,0,125:220 0/1:255,0,231:203 0/1:255,0,113:238 0/1:186,0,182:57 0/1:157,0,19:34 0/1:72,0,105:22 0/1:174,0,255:218

7 1625867 . C T 999 . . GT:PL:DP 0/1:197,0,160:163 0/1:128,0,255:153 0/1:52,0,87:176 0/1:99,0,177:46 0/1:75,0,120:30 0/1:17,0,185:19 0/1:148,0,255:203

7 1626073 . T A 999 . . GT:PL:DP 0/1:255,0,255:175 0/1:255,0,255:175 0/1:255,0,255:228 0/1:54,0,255:38 0/1:255,0,196:35 0/1:33,0,181:11 0/1:227,0,255:213

7 1626701 . C T 999 . . GT:PL:DP 0/1:255,0,136:56 0/1:255,0,232:44 0/1:255,0,164:48 0/1:80,0,74:7 0/1:46,0,95:6 0/1:226,0,130:18 0/1:255,0,255:107

7 1627077 . T G 999 . . GT:PL:DP 0/1:255,0,255:66 0/1:255,0,225:66 0/1:255,0,248:71 0/1:196,0,149:17 0/1:72,0,84:6 0/1:221,0,82:18 0/1:255,0,255:154

7 1628226 . G A 999 . . GT:PL:DP 0/1:255,0,93:107 0/1:255,0,255:83 0/1:255,0,114:124 0/1:249,0,255:29 0/1:118,0,171:14 0/1:170,0,149:16 0/1:255,0,255:160

7 1630484 . A G 999 . . GT:PL:DP 0/1:255,0,109:84 0/1:255,0,255:99 0/1:255,0,255:118 0/1:157,0,213:18 0/1:110,0,120:9 0/1:225,0,112:16 0/1:255,0,255:146

7 1632998 . G A 999 . . GT:PL:DP 0/1:38,0,255:72 0/1:237,0,255:65 0/1:255,0,255:82 0/1:14,0,255:19 0/1:79,0,148:9 0/1:22,0,255:18 0/1:255,0,255:177

7 1674708 . T G 999 . . GT:PL:DP 0/1:55,0,120:11 0/1:46,0,74:11 0/1:89,0,147:15 0/1:31,6,0:2 0/1:27,3,0:1 0/1:52,6,0:2 0/1:70,0,79:13

7 1714326 . T C 999 . . GT:PL:DP 0/1:51,0,86:16 0/1:26,0,102:13 0/1:52,0,80:12 0/1:20,3,0:1 0/1:30,0,42:5 0/1:11,3,0:1 0/1:116,0,185:36

7 1982035 . C A 999 . . GT:PL:DP 0/1:76,2,98:88 0/1:110,0,175:86 0/1:28,0,84:74 0/1:24,7,0:11 0/1:37,0,17:18 0/1:88,6,0:19 0/1:118,0,121:106

7 2188551 . A T 999 . . GT:PL:DP 0/1:255,0,160:35 0/1:243,0,169:39 0/1:253,0,179:52 0/1:52,0,61:7 0/1:28,0,21:2 0/1:68,0,86:10 0/1:157,0,224:49

7 2194320 . T C 999 . . GT:PL:DP 0/1:255,0,184:39 0/1:117,0,255:33 0/1:255,0,255:32 0/1:65,0,16:3 0/1:35,0,32:2 0/1:36,0,30:3 0/1:255,0,255:53

7 2210809 . A G 999 . . GT:PL:DP 0/1:150,0,107:109 0/1:153,0,144:124 0/1:124,0,157:73 0/1:71,0,84:9 0/1:23,0,26:6 0/1:19,0,50:8 0/1:126,0,221:107

7 2210810 . T A 999 . . GT:PL:DP 0/1:147,0,107:108 0/1:150,0,144:122 0/1:129,0,160:73 0/1:73,0,53:8 0/1:23,0,26:6 0/1:19,0,49:8 0/1:125,0,223:103

7 2210822 . A G 999 . . GT:PL:DP 0/1:111,0,92:120 0/1:121,0,123:122 0/1:116,0,138:79 0/1:45,0,25:13 0/1:27,2,0:4 0/1:24,2,0:6 0/1:121,0,163:101

7 2249090 . C A 999 . . GT:PL:DP 0/1:137,0,255:26 0/1:154,0,228:29 0/1:112,0,255:34 0/1:79,0,80:7 0/1:63,0,198:11 0/1:23,0,35:2 0/1:229,0,255:60

7 2249092 . G A 999 . . GT:PL:DP 0/1:131,0,253:25 0/1:138,0,233:26 0/1:109,0,255:31 0/1:47,0,83:6 0/1:43,0,223:12 0/1:23,0,34:2 0/1:234,0,255:60

7 2249093 . A T 999 . . GT:PL:DP 0/1:128,0,255:26 0/1:129,0,238:23 0/1:106,0,255:32 0/1:63,0,102:7 0/1:43,0,220:12 0/1:23,0,35:2 0/1:220,0,255:56

7 2249110 . A G 999 . . GT:PL:DP 0/1:243,0,247:43 0/1:246,0,215:40 0/1:218,0,255:47 0/1:36,0,121:8 0/1:72,0,215:15 0/1:34,0,65:6 0/1:255,0,255:66

7 2277780 . T G 999 . . GT:PL:DP 0/1:255,0,165:37 0/1:233,0,6:29 0/1:187,0,176:24 0/1:32,0,12:3 0/1:32,0,7:2 0/1:95,9,0:3 0/1:175,0,255:34

7 2278219 . A T 999 . . GT:PL:DP 0/1:58,0,161:28 0/1:237,0,53:33 0/1:191,0,138:43 0/1:16,0,65:6 0/1:42,0,86:9 0/1:74,0,14:7 0/1:182,0,157:50

7 2318988 . A T 999 . . GT:PL:DP 0/1:173,5,0:9 0/1:72,0,6:9 0/1:205,10,0:11 0/1:79,9,0:3 0/1:57,9,0:3 0/1:29,3,0:1 0/1:44,0,1:8

7 2377595 . A T 999 . . GT:PL:DP 0/1:103,0,84:17 0/1:98,0,12:15 0/1:99,0,115:13 0/1:8,0,63:6 0/1:26,0,30:5 0/1:38,0,56:4 0/1:105,0,215:34

7 2559934 . C T 999 . . GT:PL:DP 0/1:100,0,179:20 0/1:19,0,213:23 0/1:112,0,255:26 0/1:21,0,69:5 0/1:51,0,95:9 0/1:30,0,154:8 0/1:11,0,206:21

7 2803340 . T A 999 . . GT:PL:DP 0/1:75,0,174:98 0/1:59,0,141:50 0/1:96,0,114:63 0/1:33,0,54:4 0/1:11,0,119:6 0/1:26,6,0:2 0/1:104,0,245:85

7 2816142 . A T 999 . . GT:PL:DP 0/1:255,0,1:51 0/1:255,0,255:53 0/1:255,0,57:38 0/1:24,0,168:8 0/1:185,0,39:15 0/1:40,0,1:12 0/1:255,0,46:71

7 2909045 . G C 999 . . GT:PL:DP 0/1:164,0,185:176 0/1:135,0,70:168 0/1:173,0,160:176 0/1:79,0,127:19 0/1:95,0,120:16 0/1:60,0,140:14 0/1:151,0,174:98

7 2909874 . G A 999 . . GT:PL:DP 0/1:255,0,255:208 0/1:255,0,255:199 0/1:255,0,238:205 0/1:204,0,255:91 0/1:109,0,89:84 0/1:70,0,3:75 0/1:255,0,255:195

7 2909920 . A C 999 . . GT:PL:DP 0/1:186,0,129:182 0/1:150,0,133:180 0/1:163,0,108:183 0/1:176,0,133:73 0/1:80,5,0:50 0/1:68,0,17:41 0/1:206,0,161:171

7 2940031 . C A 999 . . GT:PL:DP 0/1:154,0,255:157 0/1:121,0,224:138 0/1:74,0,255:117 0/1:24,18,162:20 0/1:54,0,165:34 0/1:117,0,194:64 0/1:35,0,223:232

7 2956006 . C T 999 . . GT:PL:DP 0/1:241,0,255:192 0/1:230,0,255:183 0/1:207,0,255:189 0/1:255,0,255:69 0/1:231,0,244:146 0/1:254,0,255:183 0/1:229,0,255:196

7 2958509 . G A 999 . . GT:PL:DP 0/1:217,0,121:30 0/1:121,0,213:25 0/1:135,0,237:32 0/1:40,6,0:2 0/1:23,0,38:3 0/1:19,0,45:3 0/1:180,0,227:38

7 2959064 . G A 999 . . GT:PL:DP 0/1:90,0,185:29 0/1:57,0,190:26 0/1:105,0,228:37 0/1:71,9,0:3 0/1:9,0,17:5 0/1:83,0,50:7 0/1:158,0,255:45

7 3001541 . C A 999 . . GT:PL:DP 0/1:255,255,255:28 0/1:255,255,255:25 0/1:255,255,255:28 0/1:74,74,74:2 0/1:67,67,67:3 0/1:129,129,129:4 0/1:255,255,255:35

7 3010497 . C T 999 . . GT:PL:DP 0/1:220,0,195:59 0/1:255,0,199:93 0/1:230,0,146:85 0/1:139,0,163:28 0/1:72,0,54:11 0/1:16,0,32:6 0/1:148,0,163:87

7 3011313 . A T 999 . . GT:PL:DP 0/1:241,0,255:79 0/1:255,0,255:95 0/1:249,0,218:81 0/1:107,0,96:11 0/1:78,0,65:11 0/1:77,0,69:9 0/1:255,0,236:76

7 3128758 . A C 999 . . GT:PL:DP 0/1:46,0,13:6 0/1:22,0,41:7 0/1:20,0,26:3 0/1:26,3,0:1 0/1:37,9,0:3 0/1:9,0,10:2 0/1:39,0,61:16

7 3128759 . T C 999 . . GT:PL:DP 0/1:46,0,13:6 0/1:22,0,37:7 0/1:20,0,26:3 0/1:26,3,0:1 0/1:37,9,0:3 0/1:9,0,10:2 0/1:39,0,61:16

7 3201276 . T C 999 . . GT:PL:DP 0/1:255,0,178:52 0/1:255,0,141:36 0/1:101,0,255:47 0/1:23,0,184:13 0/1:97,0,131:9 0/1:62,0,113:7 0/1:92,0,255:42

7 3258256 . G C 999 . . GT:PL:DP 0/1:97,0,14:10 0/1:109,0,191:28 0/1:104,0,124:15 0/1:40,6,0:2 0/1:44,6,0:2 0/1:61,9,0:3 0/1:129,0,157:23

7 3274886 . T C 999 . . GT:PL:DP 0/1:98,0,42:23 0/1:57,0,21:15 0/1:67,0,48:18 0/1:37,6,0:2 0/1:13,0,8:2 0/1:26,6,0:2 0/1:83,0,16:14

7 3316043 . T C 999 . . GT:PL:DP 0/1:149,0,255:204 0/1:255,0,255:80 0/1:255,0,255:101 0/1:8,0,255:33 0/1:151,0,255:32 0/1:193,0,255:35 0/1:46,0,255:201

7 3317201 . C T 999 . . GT:PL:DP 0/1:255,0,255:217 0/1:188,0,255:176 0/1:235,0,255:192 0/1:47,0,255:50 0/1:170,0,255:67 0/1:229,0,255:49 0/1:230,0,255:222

7 3350567 . A T 999 . . GT:PL:DP 0/1:223,0,162:74 0/1:207,0,138:53 0/1:247,0,181:53 0/1:41,0,24:9 0/1:135,0,23:10 0/1:24,0,85:14 0/1:160,0,214:95

7 3350651 . T G 999 . . GT:PL:DP 0/1:210,0,183:62 0/1:215,0,88:56 0/1:253,0,151:61 0/1:40,0,40:8 0/1:18,0,10:5 0/1:77,0,104:14 0/1:146,0,194:79

7 3682649 . A T 999 . . GT:PL:DP 0/1:26,0,88:37 0/1:30,0,92:34 0/1:87,0,66:43 0/1:54,0,98:28 0/1:19,0,63:17 0/1:43,0,98:27 0/1:20,0,91:70

7 3783128 . G T 999 . . GT:PL:DP 0/1:110,0,206:77 0/1:22,0,123:79 0/1:147,0,171:103 0/1:24,0,39:6 0/1:8,0,98:13 0/1:120,0,141:17 0/1:34,0,242:100

7 3858796 . C T 999 . . GT:PL:DP 0/1:132,0,252:44 0/1:192,0,255:69 0/1:55,0,226:95 0/1:65,0,132:12 0/1:44,0,150:8 0/1:85,0,184:14 0/1:188,0,246:56

7 3858848 . C T 999 . . GT:PL:DP 0/1:154,0,213:42 0/1:195,0,255:74 0/1:117,0,186:102 0/1:44,0,143:13 0/1:33,0,99:10 0/1:61,0,144:18 0/1:207,0,244:64

7 3859145 . C T 999 . . GT:PL:DP 0/1:7,0,119:26 0/1:48,0,121:28 0/1:81,0,127:34 0/1:38,0,59:6 0/1:49,0,59:10 0/1:15,0,86:6 0/1:89,0,76:14

7 4034254 . G T 999 . . GT:PL:DP 0/1:12,0,219:18 0/1:135,0,242:31 0/1:9,0,255:29 0/1:65,9,0:3 0/1:18,0,24:2 0/1:29,3,0:1 0/1:91,0,255:30

7 4108230 . A T 999 . . GT:PL:DP 0/1:64,0,131:22 0/1:103,0,156:38 0/1:168,0,178:37 0/1:26,0,42:5 0/1:30,0,4:3 0/1:11,0,47:5 0/1:145,0,180:22

7 4188328 . T G 999 . . GT:PL:DP 0/1:208,0,90:26 0/1:190,0,100:25 0/1:12,0,17:2 0/1:12,0,21:2 0/1:56,0,27:5 0/1:48,9,0:3 0/1:209,0,139:32

7 4215924 . A G 999 . . GT:PL:DP 0/1:13,0,161:14 0/1:15,0,194:26 0/1:27,0,118:28 0/1:17,0,51:3 0/1:45,0,19:4 0/1:31,3,0:1 0/1:26,0,107:8

7 4216028 . C T 999 . . GT:PL:DP 0/1:86,0,60:162 0/1:167,0,213:145 0/1:125,0,111:145 0/1:123,0,15:33 0/1:149,0,78:43 0/1:82,0,45:75 0/1:91,0,26:169

7 4216061 . G A 999 . . GT:PL:DP 0/1:136,0,121:178 0/1:181,0,255:164 0/1:170,0,181:151 0/1:131,0,21:35 0/1:160,0,56:38 0/1:54,0,42:72 0/1:97,0,33:193

7 4216063 . C T 999 . . GT:PL:DP 0/1:126,0,89:171 0/1:205,0,255:164 0/1:191,0,190:141 0/1:143,0,26:35 0/1:156,0,108:37 0/1:48,0,42:72 0/1:101,0,22:190

7 4406684 . A G 999 . . GT:PL:DP 0/1:145,0,255:227 0/1:63,0,250:229 0/1:187,0,255:226 0/1:96,0,255:119 0/1:143,0,255:122 0/1:255,0,255:154 0/1:129,0,255:253

7 4495046 . C A 999 . . GT:PL:DP 0/1:255,0,234:78 0/1:255,0,163:98 0/1:208,0,105:41 0/1:142,0,89:24 0/1:166,0,29:18 0/1:255,0,204:71 0/1:255,0,226:173

7 4690758 . C T 999 . . GT:PL:DP 0/1:79,0,26:18 0/1:84,0,0:13 0/1:103,0,49:14 0/1:15,3,0:1 0/1:76,0,16:7 0/1:34,0,0:4 0/1:94,0,77:22

7 4729230 . A T 999 . . GT:PL:DP 0/1:15,0,109:13 0/1:57,0,169:27 0/1:166,0,79:27 0/1:108,6,0:6 0/1:15,6,0:2 0/1:29,9,0:3 0/1:135,0,228:47

7 4740540 . T A 999 . . GT:PL:DP 0/1:255,0,255:164 0/1:238,0,227:159 0/1:255,0,244:187 0/1:136,0,157:23 0/1:41,0,51:14 0/1:154,0,15:14 0/1:255,0,231:166

7 4900401 . A T 999 . . GT:PL:DP 0/1:183,0,157:21 0/1:203,0,93:24 0/1:129,0,148:13 0/1:32,3,0:1 0/1:117,0,55:8 0/1:102,0,87:11 0/1:255,0,255:55

7 4970426 . G A 999 . . GT:PL:DP 0/1:155,0,5:10 0/1:44,0,157:17 0/1:104,0,116:16 0/1:20,3,0:1 0/1:27,0,49:3 0/1:149,0,255:37 0/1:205,0,201:31

7 5016572 . T C 999 . . GT:PL:DP 0/1:160,0,207:45 0/1:141,0,238:39 0/1:132,0,188:38 0/1:31,0,9:3 0/1:59,0,59:9 0/1:20,0,57:6 0/1:123,0,31:14

7 5037984 . G A 999 . . GT:PL:DP 0/1:224,0,255:41 0/1:245,0,255:54 0/1:116,0,237:26 0/1:74,0,49:9 0/1:126,0,40:9 0/1:68,0,189:17 0/1:219,0,255:46

7 5038345 . G T 999 . . GT:PL:DP 0/1:127,0,255:45 0/1:140,0,255:43 0/1:184,0,255:37 0/1:51,0,125:8 0/1:42,0,95:7 0/1:20,0,185:12 0/1:211,0,255:75

7 5038389 . A G 999 . . GT:PL:DP 0/1:130,0,255:36 0/1:189,0,255:48 0/1:197,0,201:34 0/1:19,0,98:5 0/1:25,0,107:11 0/1:28,0,124:17 0/1:209,0,255:70

7 5101306 . C T 999 . . GT:PL:DP 0/1:133,0,188:23 0/1:159,0,146:22 0/1:42,0,54:10 0/1:8,0,68:4 0/1:18,0,73:4 0/1:75,9,0:3 0/1:190,0,173:36

7 5123764 . T A 999 . . GT:PL:DP 0/1:110,0,56:14 0/1:79,0,108:20 0/1:83,0,110:21 0/1:10,3,0:1 0/1:43,0,16:4 0/1:12,0,10:5 0/1:119,0,34:16

7 5303769 . T C 999 . . GT:PL:DP 0/1:235,0,255:83 0/1:220,0,255:112 0/1:115,0,255:187 0/1:86,0,141:20 0/1:44,0,240:19 0/1:41,0,255:51 0/1:228,0,255:165

7 5303775 . G A 999 . . GT:PL:DP 0/1:190,0,255:87 0/1:152,0,230:119 0/1:255,0,255:184 0/1:110,0,149:21 0/1:169,0,156:22 0/1:255,0,122:55 0/1:163,0,255:174

7 5328876 . T G 999 . . GT:PL:DP 0/1:213,0,250:32 0/1:111,0,255:33 0/1:255,0,91:43 0/1:34,0,87:6 0/1:27,6,0:2 0/1:44,0,94:8 0/1:255,0,80:33

7 5341048 . C T 999 . . GT:PL:DP 0/1:148,0,12:7 0/1:209,0,227:33 0/1:187,0,138:18 0/1:60,6,0:2 0/1:62,6,0:2 0/1:26,3,0:1 0/1:146,0,183:23

7 5366076 . A T 999 . . GT:PL:DP 0/1:20,0,47:5 0/1:180,0,144:29 0/1:21,0,134:14 0/1:37,3,0:1 0/1:72,0,19:5 0/1:31,3,0:1 0/1:21,0,200:13

7 5404710 . G T 999 . . GT:PL:DP 0/1:108,0,245:210 0/1:138,0,236:175 0/1:215,0,255:195 0/1:70,0,210:60 0/1:38,0,50:11 0/1:23,0,115:22 0/1:236,0,255:201

7 5638989 . C T 999 . . GT:PL:DP 0/1:124,0,255:29 0/1:49,0,255:25 0/1:182,0,245:37 0/1:37,0,60:4 0/1:44,0,69:6 0/1:21,0,25:2 0/1:174,0,255:47

7 5639668 . G A 999 . . GT:PL:DP 0/1:253,0,255:39 0/1:226,0,255:43 0/1:255,0,255:40 0/1:105,0,136:9 0/1:89,0,97:8 0/1:120,0,88:9 0/1:255,0,255:49

7 5676865 . C A 999 . . GT:PL:DP 0/1:255,0,255:82 0/1:255,0,255:78 0/1:85,0,255:202 0/1:154,0,255:40 0/1:27,0,50:3 0/1:229,0,255:34 0/1:255,0,255:145

7 5676904 . C A 999 . . GT:PL:DP 0/1:255,0,255:86 0/1:255,0,255:77 0/1:142,0,255:192 0/1:174,0,255:44 0/1:69,11,13:4 0/1:219,0,255:26 0/1:255,0,255:114

7 5676988 . C T 999 . . GT:PL:DP 0/1:255,0,255:88 0/1:255,0,255:72 0/1:255,0,124:200 0/1:255,0,54:44 0/1:48,0,159:8 0/1:255,0,86:25 0/1:255,0,255:160

7 5677445 . T C 999 . . GT:PL:DP 0/1:255,0,255:161 0/1:255,0,255:115 0/1:255,0,125:197 0/1:255,0,66:26 0/1:65,0,227:15 0/1:255,0,130:29 0/1:255,0,255:141

7 5677618 . A G 999 . . GT:PL:DP 0/1:255,0,255:148 0/1:255,0,255:141 0/1:160,0,255:207 0/1:104,0,255:44 0/1:239,0,55:19 0/1:101,0,255:37 0/1:255,0,255:183

7 5709295 . C T 999 . . GT:PL:DP 0/1:182,0,255:34 0/1:61,0,213:16 0/1:57,0,174:10 0/1:24,0,78:4 0/1:61,6,0:2 0/1:28,0,46:3 0/1:140,0,216:23

7 5733783 . A G 999 . . GT:PL:DP 0/1:70,0,178:24 0/1:55,0,106:8 0/1:166,0,210:40 0/1:17,3,0:1 0/1:12,3,0:1 0/1:32,3,0:1 0/1:89,0,135:17

7 5864426 . G A 999 . . GT:PL:DP 0/1:79,0,255:46 0/1:119,0,252:34 0/1:174,0,255:78 0/1:72,0,52:9 0/1:27,0,179:10 0/1:63,0,182:12 0/1:156,0,255:75

7 5864429 . G T 999 . . GT:PL:DP 0/1:94,0,255:47 0/1:109,0,236:35 0/1:166,0,255:80 0/1:78,0,61:9 0/1:27,0,189:11 0/1:60,0,201:13 0/1:160,0,255:77

7 5864437 . C A 999 . . GT:PL:DP 0/1:91,0,255:59 0/1:97,0,255:42 0/1:201,0,255:86 0/1:79,0,128:13 0/1:36,0,213:17 0/1:67,0,209:17 0/1:160,0,255:78

7 6017452 . T G 999 . . GT:PL:DP 0/1:255,0,115:24 0/1:120,0,255:65 0/1:255,0,255:66 0/1:44,0,194:10 0/1:38,0,157:11 0/1:31,0,33:3 0/1:255,0,255:76

7 6072428 . C T 20.5 . . GT:PL:DP 0/1:12,3,0:1 0/1:12,3,0:1 0/1:44,12,0:4 0/1:14,3,0:1 0/1:28,9,0:3 0/1:12,3,0:1 0/1:16,6,0:2

7 6199073 . G A 999 . . GT:PL:DP 0/1:240,0,254:53 0/1:232,0,205:38 0/1:184,0,255:38 0/1:104,0,73:9 0/1:67,0,33:5 0/1:152,0,102:14 0/1:226,0,255:73

7 6218763 . T C 999 . . GT:PL:DP 0/1:50,0,81:9 0/1:39,0,205:13 0/1:163,0,208:20 0/1:95,9,0:3 0/1:23,0,46:5 0/1:69,0,105:9 0/1:167,0,255:73

7 6255026 . G T 999 . . GT:PL:DP 0/1:214,0,254:26 0/1:130,0,255:25 0/1:178,0,158:18 0/1:19,0,113:6 0/1:27,0,33:2 0/1:99,0,116:9 0/1:216,0,255:43

7 6285478 . T C 999 . . GT:PL:DP 0/1:63,0,4:9 0/1:48,0,49:11 0/1:85,0,11:12 0/1:39,0,12:4 0/1:17,3,0:1 0/1:37,5,0:4 0/1:56,0,36:15

7 6287421 . A G 999 . . GT:PL:DP 0/1:132,0,255:149 0/1:145,0,255:137 0/1:109,0,220:115 0/1:166,0,137:50 0/1:57,0,90:20 0/1:72,0,103:23 0/1:177,0,255:243

7 6287437 . A C 999 . . GT:PL:DP 0/1:184,0,255:180 0/1:152,0,237:157 0/1:137,0,234:136 0/1:174,0,181:57 0/1:123,0,78:31 0/1:129,0,67:43 0/1:170,0,203:252

7 6302224 . A T 999 . . GT:PL:DP 0/1:142,4,0:14 0/1:171,0,61:38 0/1:176,0,69:48 0/1:28,0,10:3 0/1:29,0,64:9 0/1:43,0,42:7 0/1:115,0,53:47

7 6302476 . C T 999 . . GT:PL:DP 0/1:159,5,0:26 0/1:117,0,99:42 0/1:138,0,63:51 0/1:14,0,3:2 0/1:54,0,74:11 0/1:127,0,37:13 0/1:90,0,117:65

7 6338450 . C A 999 . . GT:PL:DP 0/1:255,0,142:53 0/1:255,0,234:56 0/1:118,0,217:63 0/1:84,0,155:21 0/1:48,0,44:15 0/1:173,6,0:39 0/1:255,0,255:131

7 6512484 . A G 999 . . GT:PL:DP 0/1:88,0,178:21 0/1:48,0,117:14 0/1:43,0,190:14 0/1:20,0,76:4 0/1:72,9,0:3 0/1:19,3,0:1 0/1:116,0,133:22

7 6565405 . G A 999 . . GT:PL:DP 0/1:137,0,94:107 0/1:49,0,122:112 0/1:35,0,137:101 0/1:14,0,126:69 0/1:49,0,50:113 0/1:11,0,13:146 0/1:62,0,160:157

7 6590718 . G A 999 . . GT:PL:DP 0/1:186,0,255:48 0/1:195,0,255:38 0/1:204,0,255:54 0/1:65,0,99:8 0/1:46,0,176:10 0/1:101,0,193:16 0/1:69,0,255:162

7 6611845 . A C 999 . . GT:PL:DP 0/1:168,0,166:146 0/1:148,0,168:110 0/1:138,0,87:131 0/1:139,0,105:20 0/1:117,0,106:15 0/1:131,0,62:17 0/1:219,0,185:228

7 6635328 . A G 999 . . GT:PL:DP 0/1:85,0,197:121 0/1:76,0,176:117 0/1:130,0,96:138 0/1:38,0,111:34 0/1:19,5,0:57 0/1:41,0,56:98 0/1:49,0,176:165

7 6681763 . A G 999 . . GT:PL:DP 0/1:44,0,121:104 0/1:61,0,161:94 0/1:125,0,104:115 0/1:29,2,0:10 0/1:17,0,22:11 0/1:95,5,0:24 0/1:76,0,115:84

7 6739804 . G C 999 . . GT:PL:DP 0/1:255,0,108:38 0/1:183,0,255:29 0/1:132,0,255:27 0/1:128,0,63:11 0/1:128,0,80:8 0/1:14,0,127:5 0/1:255,0,255:56

7 6739811 . T A 999 . . GT:PL:DP 0/1:254,0,92:33 0/1:194,0,247:28 0/1:130,0,222:23 0/1:112,0,86:11 0/1:102,0,83:7 0/1:20,0,127:5 0/1:255,0,255:54

7 6741060 . G T 999 . . GT:PL:DP 0/1:151,0,64:16 0/1:44,0,198:22 0/1:76,0,202:28 0/1:19,0,27:2 0/1:30,0,21:3 0/1:10,0,47:3 0/1:69,0,212:31

7 6742385 . C A 999 . . GT:PL:DP 0/1:222,0,47:23 0/1:137,0,133:26 0/1:232,0,27:23 0/1:70,9,0:3 0/1:61,0,10:4 0/1:56,0,12:4 0/1:81,0,122:20

7 6755948 . A G 999 . . GT:PL:DP 0/1:255,0,255:60 0/1:255,0,255:46 0/1:255,0,218:36 0/1:23,0,237:12 0/1:104,0,205:23 0/1:52,0,18:3 0/1:255,0,255:70

7 6763733 . T A 999 . . GT:PL:DP 0/1:218,0,170:39 0/1:32,0,186:19 0/1:209,0,78:30 0/1:110,0,63:7 0/1:124,0,107:27 0/1:230,0,197:57 0/1:255,0,255:91

7 6827415 . G A 999 . . GT:PL:DP 0/1:131,0,89:23 0/1:202,0,16:29 0/1:213,0,18:28 0/1:37,0,28:3 0/1:56,0,18:5 0/1:47,0,85:5 0/1:225,0,64:42

7 7192137 . G A 999 . . GT:PL:DP 0/1:81,0,100:22 0/1:103,0,115:24 0/1:135,0,44:22 0/1:57,0,10:5 0/1:27,0,10:3 0/1:21,0,20:5 0/1:100,0,106:32

7 7192144 . T G 999 . . GT:PL:DP 0/1:65,0,82:21 0/1:102,0,90:26 0/1:136,0,23:25 0/1:42,0,7:4 0/1:27,0,10:3 0/1:21,0,15:5 0/1:91,0,52:28

7 7192145 . T G 999 . . GT:PL:DP 0/1:75,0,63:21 0/1:76,0,116:24 0/1:20,0,139:25 0/1:6,0,42:4 0/1:10,0,27:3 0/1:13,0,35:5 0/1:44,0,112:28

7 7206622 . C T 999 . . GT:PL:DP 0/1:31,2,0:6 0/1:98,0,128:26 0/1:76,0,120:25 0/1:43,0,18:4 0/1:36,3,0:1 0/1:59,9,0:3 0/1:30,6,0:2

7 7225161 . T A 999 . . GT:PL:DP 0/1:87,0,1:7 0/1:67,9,29:9 0/1:68,6,12:6 0/1:18,0,17:2 0/1:34,6,0:2 0/1:34,6,0:2 0/1:139,22,11:16

7 7412683 . C T 999 . . GT:PL:DP 0/1:255,0,35:33 0/1:205,0,47:18 0/1:239,0,52:19 0/1:24,0,30:2 0/1:119,2,0:7 0/1:13,0,59:7 0/1:238,0,221:45

7 7472421 . C T 999 . . GT:PL:DP 0/1:233,0,255:240 0/1:233,0,255:189 0/1:255,0,255:165 0/1:76,0,255:35 0/1:18,0,232:32 0/1:17,0,110:17 0/1:90,0,255:231

7 7541368 . A T 999 . . GT:PL:DP 0/1:93,0,197:28 0/1:56,0,255:40 0/1:108,0,54:10 0/1:76,0,196:14 0/1:9,3,0:1 0/1:42,0,92:7 0/1:157,0,255:65

7 7615627 . C T 999 . . GT:PL:DP 0/1:255,0,153:220 0/1:255,0,91:233 0/1:255,0,255:222 0/1:255,0,255:65 0/1:255,0,255:52 0/1:255,0,255:92 0/1:255,0,255:224

7 7616345 . A G 999 . . GT:PL:DP 0/1:255,0,255:203 0/1:255,0,255:232 0/1:255,0,255:222 0/1:255,0,185:57 0/1:71,0,239:41 0/1:213,0,255:51 0/1:255,0,255:220

7 7645837 . G A 999 . . GT:PL:DP 0/1:174,0,244:33 0/1:171,0,65:19 0/1:151,0,255:33 0/1:79,0,13:8 0/1:64,0,17:5 0/1:25,0,149:9 0/1:255,0,255:56

7 7647256 . C A 999 . . GT:PL:DP 0/1:255,0,145:31 0/1:255,0,173:53 0/1:255,0,17:26 0/1:90,0,43:5 0/1:101,0,91:8 0/1:79,0,25:4 0/1:255,0,177:31

7 7649938 . A T 999 . . GT:PL:DP 0/1:65,0,255:40 0/1:183,0,255:59 0/1:186,0,255:58 0/1:27,0,243:20 0/1:70,0,105:9 0/1:112,0,68:8 0/1:53,0,255:44

7 7651112 . A G 999 . . GT:PL:DP 0/1:255,0,255:52 0/1:255,0,255:73 0/1:207,0,255:64 0/1:19,0,124:6 0/1:92,0,192:11 0/1:255,0,92:20 0/1:255,0,255:122

7 7652133 . T A 999 . . GT:PL:DP 0/1:169,0,182:27 0/1:186,0,255:42 0/1:183,0,255:58 0/1:45,6,0:2 0/1:24,0,96:6 0/1:136,0,0:11 0/1:255,0,239:71

7 7652313 . C T 999 . . GT:PL:DP 0/1:36,0,255:38 0/1:226,0,255:61 0/1:255,0,255:66 0/1:51,0,118:7 0/1:70,0,135:8 0/1:40,0,117:6 0/1:210,0,255:143

7 7652368 . C A 999 . . GT:PL:DP 0/1:41,0,255:36 0/1:227,0,255:57 0/1:255,0,255:74 0/1:156,0,77:10 0/1:59,0,177:10 0/1:78,0,190:12 0/1:255,0,255:164

7 7656940 . G C 999 . . GT:PL:DP 0/1:157,0,255:44 0/1:199,0,255:53 0/1:206,0,255:38 0/1:84,1,0:9 0/1:36,0,155:14 0/1:107,0,89:21 0/1:207,0,255:69

7 7657033 . G A 999 . . GT:PL:DP 0/1:235,0,255:50 0/1:255,0,255:51 0/1:255,0,255:37 0/1:139,0,36:7 0/1:124,0,209:18 0/1:255,0,44:19 0/1:255,0,255:79

7 7663099 . T C 999 . . GT:PL:DP 0/1:255,0,255:56 0/1:255,0,226:52 0/1:188,0,255:30 0/1:8,0,81:4 0/1:67,6,0:2 0/1:102,0,124:8 0/1:255,0,255:125

7 7663204 . C T 999 . . GT:PL:DP 0/1:255,0,255:62 0/1:255,0,92:41 0/1:210,0,205:22 0/1:24,0,139:8 0/1:48,0,86:6 0/1:12,0,208:11 0/1:255,0,255:81

7 7663207 . A T 999 . . GT:PL:DP 0/1:255,0,255:64 0/1:255,0,103:44 0/1:219,0,192:21 0/1:24,0,139:8 0/1:54,0,61:4 0/1:14,0,212:12 0/1:255,0,255:103

7 7663740 . C G 999 . . GT:PL:DP 0/1:37,0,255:33 0/1:143,0,254:27 0/1:138,0,255:27 0/1:7,0,200:10 0/1:25,0,97:4 0/1:40,0,117:7 0/1:167,0,255:82

7 7663831 . G T 999 . . GT:PL:DP 0/1:255,0,142:24 0/1:218,0,156:26 0/1:166,0,182:23 0/1:33,0,105:6 0/1:31,0,31:2 0/1:60,0,61:4 0/1:255,0,255:73

7 7675901 . C T 999 . . GT:PL:DP 0/1:152,0,152:63 0/1:195,0,193:62 0/1:201,0,129:75 0/1:72,0,36:7 0/1:19,0,123:13 0/1:118,0,118:17 0/1:208,0,195:121

7 7756923 . T G 999 . . GT:PL:DP 0/1:101,0,74:16 0/1:127,0,99:18 0/1:185,0,92:25 0/1:28,0,62:6 0/1:23,0,23:2 0/1:66,9,0:3 0/1:146,0,169:30

7 7762727 . A T 999 . . GT:PL:DP 0/1:87,9,0:3 0/1:150,0,158:34 0/1:149,0,103:19 0/1:69,0,25:4 0/1:39,3,0:1 0/1:116,0,53:8 0/1:248,0,249:30

7 7776703 . G A 999 . . GT:PL:DP 0/1:243,0,255:102 0/1:255,0,255:73 0/1:255,0,255:72 0/1:120,0,213:15 0/1:120,0,224:17 0/1:155,0,220:20 0/1:225,0,255:64

7 7776873 . C G 999 . . GT:PL:DP 0/1:255,0,255:92 0/1:255,0,255:70 0/1:254,0,255:54 0/1:52,0,211:13 0/1:166,0,99:17 0/1:141,0,191:23 0/1:251,0,255:57

7 7777203 . T G 999 . . GT:PL:DP 0/1:236,0,237:67 0/1:254,0,223:68 0/1:202,0,172:44 0/1:15,0,86:6 0/1:191,0,74:16 0/1:92,0,54:11 0/1:194,0,175:49

7 7783482 . A G 999 . . GT:PL:DP 0/1:224,0,99:62 0/1:231,0,255:48 0/1:218,0,170:67 0/1:92,0,41:7 0/1:83,0,27:7 0/1:56,0,112:9 0/1:188,0,102:83

7 7791934 . A T 999 . . GT:PL:DP 0/1:219,0,255:121 0/1:255,0,255:58 0/1:210,0,255:58 0/1:251,0,197:30 0/1:67,0,134:11 0/1:48,0,174:12 0/1:255,0,255:209

7 7797061 . G A 999 . . GT:PL:DP 0/1:11,0,255:62 0/1:61,0,255:45 0/1:111,0,255:63 0/1:68,0,184:11 0/1:40,0,148:11 0/1:42,0,201:11 0/1:160,0,255:125

7 7807454 . A G 999 . . GT:PL:DP 0/1:255,0,255:51 0/1:255,0,255:69 0/1:214,0,255:32 0/1:85,0,226:14 0/1:33,0,222:12 0/1:27,0,255:14 0/1:255,0,255:65

7 7825034 . G T 999 . . GT:PL:DP 0/1:224,0,115:51 0/1:115,0,255:61 0/1:255,0,55:60 0/1:85,0,20:9 0/1:163,0,112:19 0/1:141,0,42:16 0/1:230,0,92:216

7 7830759 . T C 999 . . GT:PL:DP 0/1:255,0,33:34 0/1:225,0,255:42 0/1:255,0,167:53 0/1:103,0,49:6 0/1:41,6,0:2 0/1:22,0,52:3 0/1:255,0,255:83

7 7830793 . C T 999 . . GT:PL:DP 0/1:255,0,5:40 0/1:255,0,255:43 0/1:255,0,102:44 0/1:130,0,46:7 0/1:24,3,0:1 0/1:60,0,25:5 0/1:255,0,67:53

7 7834658 . G C 999 . . GT:PL:DP 0/1:191,0,233:107 0/1:48,0,212:124 0/1:255,0,176:67 0/1:97,0,122:18 0/1:22,0,172:20 0/1:67,0,158:15 0/1:175,0,212:174

7 7842075 . A C 999 . . GT:PL:DP 0/1:255,0,163:33 0/1:168,0,235:28 0/1:225,0,238:40 0/1:88,0,126:9 0/1:59,6,0:2 0/1:54,0,99:8 0/1:255,0,102:47

7 7849038 . C T 999 . . GT:PL:DP 0/1:255,0,206:50 0/1:255,0,255:110 0/1:255,0,237:59 0/1:27,0,115:5 0/1:57,0,57:4 0/1:160,0,83:10 0/1:255,0,255:78

7 7862663 . G C 999 . . GT:PL:DP 0/1:218,0,255:74 0/1:58,0,255:84 0/1:208,0,255:80 0/1:84,0,208:17 0/1:31,0,132:9 0/1:70,0,216:14 0/1:144,0,255:162

7 7862827 . C T 999 . . GT:PL:DP 0/1:255,0,70:36 0/1:227,0,255:46 0/1:255,0,244:67 0/1:105,0,84:9 0/1:117,0,36:13 0/1:109,0,61:11 0/1:255,0,143:82

7 7862911 . A G 999 . . GT:PL:DP 0/1:255,0,187:57 0/1:222,0,255:49 0/1:178,0,255:63 0/1:43,0,110:10 0/1:118,0,26:9 0/1:71,0,72:8 0/1:255,0,255:107

7 7866453 . A G 999 . . GT:PL:DP 0/1:119,0,173:38 0/1:117,0,216:67 0/1:162,0,246:88 0/1:77,0,87:14 0/1:9,0,73:6 0/1:72,0,21:5 0/1:153,0,174:39

7 7866480 . G T 999 . . GT:PL:DP 0/1:106,0,228:38 0/1:48,0,198:57 0/1:29,0,236:73 0/1:69,0,59:11 0/1:51,0,88:12 0/1:27,0,85:9 0/1:34,0,237:43

7 7866660 . C T 999 . . GT:PL:DP 0/1:99,0,106:56 0/1:54,0,87:79 0/1:86,0,99:88 0/1:51,0,28:11 0/1:121,0,21:22 0/1:15,2,16:10 0/1:189,0,66:48

7 7869699 . A T 999 . . GT:PL:DP 0/1:201,0,255:30 0/1:149,0,254:27 0/1:154,0,255:23 0/1:37,0,48:6 0/1:25,0,51:4 0/1:32,0,56:4 0/1:255,0,255:52

7 8024693 . C A 999 . . GT:PL:DP 0/1:190,0,219:58 0/1:196,0,242:50 0/1:107,0,193:95 0/1:42,0,120:9 0/1:82,0,58:5 0/1:99,0,168:14 0/1:39,0,255:97

7 8088304 . A T 999 . . GT:PL:DP 0/1:184,0,140:31 0/1:156,0,173:29 0/1:182,0,131:31 0/1:55,0,101:9 0/1:31,0,14:2 0/1:17,3,0:1 0/1:174,0,194:35

7 8100431 . A T 999 . . GT:PL:DP 0/1:159,0,255:96 0/1:194,0,255:126 0/1:198,0,255:115 0/1:131,0,66:12 0/1:109,0,35:14 0/1:124,0,180:36 0/1:161,0,255:198

7 8157816 . A T 999 . . GT:PL:DP 0/1:126,0,142:88 0/1:170,0,159:109 0/1:126,0,204:97 0/1:111,0,42:20 0/1:131,0,101:17 0/1:76,0,47:9 0/1:192,0,120:134

7 8158111 . C T 999 . . GT:PL:DP 0/1:255,0,255:221 0/1:243,0,255:226 0/1:255,0,255:217 0/1:241,0,163:56 0/1:57,0,156:70 0/1:16,0,177:93 0/1:104,0,255:245

7 8218188 . G A 999 . . GT:PL:DP 0/1:224,0,74:26 0/1:95,0,75:14 0/1:68,0,253:20 0/1:12,0,19:2 0/1:57,14,8:3 0/1:25,0,57:5 0/1:248,0,116:24

7 8273900 . G A 999 . . GT:PL:DP 0/1:119,0,225:147 0/1:107,0,161:160 0/1:104,0,255:157 0/1:90,0,187:44 0/1:8,0,111:25 0/1:46,0,18:14 0/1:175,0,255:144

7 8274083 . C G 999 . . GT:PL:DP 0/1:226,0,173:84 0/1:250,0,245:113 0/1:255,0,247:104 0/1:103,0,249:32 0/1:159,3,21:50 0/1:90,0,141:52 0/1:255,0,255:224

7 8274138 . C T 999 . . GT:PL:DP 0/1:255,0,255:234 0/1:255,0,255:252 0/1:255,0,255:257 0/1:248,0,255:59 0/1:149,0,181:77 0/1:127,0,186:71 0/1:255,0,255:287

7 8293753 . C A 999 . . GT:PL:DP 0/1:75,0,244:19 0/1:110,0,247:21 0/1:203,0,123:22 0/1:83,0,8:4 0/1:66,6,0:2 0/1:150,0,9:7 0/1:208,0,249:34

7 8294950 . C A 999 . . GT:PL:DP 0/1:126,0,48:23 0/1:118,0,132:28 0/1:101,0,50:16 0/1:21,0,40:5 0/1:22,0,24:3 0/1:18,3,0:1 0/1:89,0,61:23

7 8319431 . G C 999 . . GT:PL:DP 0/1:171,0,231:122 0/1:148,0,255:109 0/1:255,0,255:108 0/1:31,0,255:24 0/1:88,0,185:17 0/1:82,0,187:19 0/1:205,0,255:178

7 8319452 . G A 999 . . GT:PL:DP 0/1:255,0,255:117 0/1:194,0,255:111 0/1:138,0,255:115 0/1:121,0,249:25 0/1:142,0,120:20 0/1:146,0,120:18 0/1:246,0,255:180

7 8319860 . A G 999 . . GT:PL:DP 0/1:84,0,255:90 0/1:47,0,255:63 0/1:93,0,255:56 0/1:34,0,80:8 0/1:100,0,199:19 0/1:120,0,255:35 0/1:103,0,255:92

7 8353242 . C G 999 . . GT:PL:DP 0/1:212,0,255:62 0/1:255,0,255:90 0/1:255,0,226:41 0/1:94,0,126:9 0/1:130,0,234:21 0/1:148,0,179:17 0/1:255,0,255:128

7 8353296 . G A 999 . . GT:PL:DP 0/1:238,0,255:47 0/1:255,0,255:82 0/1:255,0,173:36 0/1:158,0,16:7 0/1:88,0,99:8 0/1:102,0,154:14 0/1:255,0,255:108

7 8353713 . C A 999 . . GT:PL:DP 0/1:95,0,135:18 0/1:201,0,76:36 0/1:177,0,97:17 0/1:56,0,83:7 0/1:29,0,38:3 0/1:70,6,0:2 0/1:181,0,182:83

7 8365706 . T A 999 . . GT:PL:DP 0/1:46,0,113:13 0/1:139,5,0:10 0/1:118,0,26:11 0/1:30,3,0:1 0/1:70,9,0:3 0/1:39,0,6:4 0/1:129,0,7:16

7 8414771 . C T 999 . . GT:PL:DP 0/1:111,0,217:48 0/1:232,0,255:82 0/1:73,0,223:60 0/1:90,0,129:12 0/1:142,0,255:25 0/1:98,0,255:35 0/1:219,0,255:161

7 8500182 . G A 999 . . GT:PL:DP 0/1:205,0,255:25 0/1:216,0,255:26 0/1:255,0,249:29 0/1:5,0,30:2 0/1:72,6,0:2 0/1:49,0,189:9 0/1:255,0,255:43

7 8527110 . A G 999 . . GT:PL:DP 0/1:226,0,128:228 0/1:248,0,145:233 0/1:196,0,98:236 0/1:239,0,39:79 0/1:30,0,39:5 0/1:75,0,15:6 0/1:195,0,120:222

7 8527183 . C T 999 . . GT:PL:DP 0/1:255,0,233:140 0/1:255,0,207:170 0/1:255,0,193:134 0/1:255,0,106:59 0/1:94,0,102:12 0/1:78,0,27:8 0/1:255,0,222:141

7 8561789 . G A 999 . . GT:PL:DP 0/1:249,0,249:196 0/1:167,0,210:211 0/1:161,0,239:178 0/1:157,0,88:47 0/1:24,5,0:78 0/1:11,0,20:49 0/1:166,0,255:225

7 8598863 . T A 999 . . GT:PL:DP 0/1:135,0,105:17 0/1:144,0,178:20 0/1:100,0,91:11 0/1:67,9,0:3 0/1:59,0,126:8 0/1:85,0,74:7 0/1:160,0,255:41

7 8758421 . A T 999 . . GT:PL:DP 0/1:90,12,0:4 0/1:80,0,111:17 0/1:16,0,72:4 0/1:45,0,28:3 0/1:35,3,0:1 0/1:62,6,0:2 0/1:30,0,93:12

7 8769402 . T A 999 . . GT:PL:DP 0/1:255,0,255:176 0/1:255,0,255:199 0/1:255,0,255:181 0/1:204,0,219:31 0/1:180,0,156:20 0/1:136,0,241:39 0/1:255,0,255:215

7 8770136 . T C 999 . . GT:PL:DP 0/1:255,0,255:116 0/1:255,0,255:143 0/1:255,0,255:151 0/1:43,0,255:26 0/1:202,0,255:49 0/1:105,0,243:33 0/1:249,0,255:175

7 8830282 . T C 999 . . GT:PL:DP 0/1:210,0,255:25 0/1:255,0,62:20 0/1:209,0,160:25 0/1:74,9,0:3 0/1:20,0,18:2 0/1:121,7,0:6 0/1:255,0,255:44

7 8859726 . A G 999 . . GT:PL:DP 0/1:188,0,255:99 0/1:243,0,255:114 0/1:18,0,255:87 0/1:12,0,255:24 0/1:10,0,162:23 0/1:186,0,229:33 0/1:205,0,255:221

7 8865033 . A C 999 . . GT:PL:DP 0/1:206,0,255:232 0/1:143,0,255:220 0/1:217,0,255:232 0/1:75,0,255:47 0/1:16,0,126:19 0/1:20,0,169:29 0/1:186,0,255:237

7 8960654 . G A 999 . . GT:PL:DP 0/1:168,0,155:25 0/1:51,0,107:16 0/1:121,0,89:24 0/1:54,6,0:2 0/1:54,6,0:2 0/1:16,0,30:4 0/1:162,0,68:25

7 8969612 . A G 999 . . GT:PL:DP 0/1:48,0,129:20 0/1:142,0,147:39 0/1:92,0,134:26 0/1:67,12,0:4 0/1:14,3,0:1 0/1:26,3,0:1 0/1:132,0,207:32

7 8988876 . C T 999 . . GT:PL:DP 0/1:183,0,227:33 0/1:133,0,255:31 0/1:192,0,163:34 0/1:19,0,31:2 0/1:100,0,95:13 0/1:79,0,89:10 0/1:85,0,255:125

7 9012943 . A T 999 . . GT:PL:DP 0/1:139,0,71:38 0/1:116,0,80:20 0/1:125,0,101:21 0/1:24,0,16:3 0/1:16,0,140:14 0/1:47,0,176:15 0/1:200,0,163:113

7 9020121 . A T 999 . . GT:PL:DP 0/1:255,0,255:245 0/1:213,0,255:250 0/1:255,0,255:243 0/1:255,0,255:186 0/1:228,0,255:172 0/1:255,0,255:174 0/1:251,0,255:232

7 9020337 . A T 999 . . GT:PL:DP 0/1:255,0,255:230 0/1:255,0,226:235 0/1:255,0,222:241 0/1:255,0,255:207 0/1:255,0,246:189 0/1:255,0,255:205 0/1:255,0,255:243

7 9020587 . T C 999 . . GT:PL:DP 0/1:28,0,214:198 0/1:30,0,235:198 0/1:89,0,255:201 0/1:12,0,209:88 0/1:125,0,255:135 0/1:103,0,255:172 0/1:110,0,255:190

7 9020618 . A G 999 . . GT:PL:DP 0/1:81,0,230:88 0/1:75,0,232:91 0/1:66,0,255:117 0/1:26,0,123:44 0/1:137,0,229:59 0/1:157,0,181:50 0/1:113,0,255:94

7 9046926 . A T 999 . . GT:PL:DP 0/1:180,0,238:27 0/1:189,0,173:28 0/1:170,0,171:21 0/1:11,3,0:1 0/1:35,6,0:2 0/1:15,0,63:3 0/1:104,0,193:17

7 9046938 . T C 999 . . GT:PL:DP 0/1:197,0,252:30 0/1:180,0,232:30 0/1:174,0,159:21 0/1:66,6,0:2 0/1:63,31,25:3 0/1:15,0,57:3 0/1:171,0,179:23

7 9139398 . T C 999 . . GT:PL:DP 0/1:255,0,255:39 0/1:136,0,255:23 0/1:198,0,255:36 0/1:111,0,120:8 0/1:54,0,100:6 0/1:62,0,56:4 0/1:200,0,255:49

7 9229922 . A T 999 . . GT:PL:DP 0/1:81,0,21:6 0/1:175,0,6:14 0/1:170,0,20:17 0/1:24,3,0:1 0/1:23,0,25:2 0/1:45,0,54:5 0/1:214,0,19:20

7 9329779 . C G 115.633 . . GT:PL:DP 0/1:50,9,0:3 0/1:98,12,0:7 0/1:31,12,0:4 0/1:20,6,0:2 0/1:24,3,0:1 0/1:23,3,0:1 0/1:10,3,0:1

7 9356225 . G A 999 . . GT:PL:DP 0/1:231,0,231:114 0/1:255,0,250:144 0/1:255,0,153:135 0/1:173,0,92:34 0/1:26,0,20:9 0/1:27,0,51:6 0/1:255,0,131:219

7 9356439 . C T 999 . . GT:PL:DP 0/1:255,0,255:191 0/1:255,0,255:220 0/1:255,0,211:198 0/1:255,0,210:52 0/1:148,0,105:33 0/1:126,0,149:34 0/1:255,0,200:201

7 9405807 . C G 999 . . GT:PL:DP 0/1:95,0,40:12 0/1:81,0,19:12 0/1:100,0,6:14 0/1:22,3,0:1 0/1:43,0,19:3 0/1:11,1,0:2 0/1:125,0,69:20

7 9405818 . C T 999 . . GT:PL:DP 0/1:140,0,203:234 0/1:61,0,171:208 0/1:68,0,197:214 0/1:17,0,220:55 0/1:102,0,189:35 0/1:118,0,184:37 0/1:131,0,188:233

7 9411594 . G C 999 . . GT:PL:DP 0/1:55,0,121:12 0/1:89,0,155:21 0/1:117,0,127:20 0/1:49,0,13:3 0/1:48,6,0:2 0/1:70,2,0:5 0/1:181,0,126:21

7 9411598 . G A 999 . . GT:PL:DP 0/1:30,0,115:13 0/1:65,0,150:22 0/1:74,0,123:19 0/1:59,0,13:3 0/1:80,9,0:3 1/1:105,20,8:5 0/1:190,0,124:22

7 9452147 . A T 999 . . GT:PL:DP 0/1:193,0,120:24 0/1:137,0,68:14 0/1:114,0,16:7 0/1:59,0,20:3 0/1:35,3,0:1 0/1:22,0,45:6 0/1:169,0,219:22

8 14240 . A G 999 . . GT:PL:DP 0/1:255,0,86:44 0/1:255,0,199:79 0/1:215,0,255:39 0/1:220,0,54:18 0/1:26,0,28:3 0/1:55,0,3:5 0/1:255,0,255:74

8 39403 . C G 999 . . GT:PL:DP 0/1:193,0,255:115 0/1:111,0,255:167 0/1:13,0,255:170 0/1:59,0,255:28 0/1:18,0,255:19 0/1:236,0,199:26 0/1:157,0,255:155

8 42243 . A G 999 . . GT:PL:DP 0/1:201,0,255:79 0/1:243,0,255:50 0/1:255,0,255:103 0/1:92,0,53:5 0/1:113,0,60:6 0/1:62,0,255:24 0/1:255,0,127:97

8 42993 . T G 999 . . GT:PL:DP 0/1:255,0,255:77 0/1:255,0,255:48 0/1:255,0,255:90 0/1:113,0,185:13 0/1:126,0,46:8 0/1:88,0,255:28 0/1:255,0,255:104

8 43293 . T C 999 . . GT:PL:DP 0/1:213,0,255:65 0/1:234,0,255:59 0/1:255,0,255:94 0/1:158,0,112:11 0/1:76,0,62:5 0/1:72,0,147:9 0/1:255,0,255:118

8 43369 . G A 999 . . GT:PL:DP 0/1:177,0,255:66 0/1:213,0,255:40 0/1:255,0,255:94 0/1:144,0,107:10 0/1:202,0,140:18 0/1:37,0,196:13 0/1:255,0,255:82

8 96233 . C A 999 . . GT:PL:DP 0/1:159,0,255:38 0/1:174,0,255:31 0/1:166,0,255:28 0/1:78,0,22:5 0/1:105,0,187:20 0/1:69,0,224:19 0/1:177,0,255:114

8 147440 . A G 999 . . GT:PL:DP 0/1:255,0,255:86 0/1:66,0,255:54 0/1:245,0,255:64 0/1:156,0,154:16 0/1:161,0,238:22 0/1:154,0,255:26 0/1:251,0,24:14

8 154788 . A T 999 . . GT:PL:DP 0/1:255,0,238:42 0/1:255,0,255:46 0/1:204,0,195:24 0/1:119,0,16:9 0/1:30,3,0:1 0/1:40,0,45:11 0/1:255,0,255:91

8 155475 . C A 999 . . GT:PL:DP 0/1:138,0,12:29 0/1:116,0,77:17 0/1:104,0,92:15 0/1:20,0,31:2 0/1:98,0,9:10 0/1:93,0,49:9 0/1:134,0,114:37

8 170597 . C T 999 . . GT:PL:DP 0/1:124,0,255:242 0/1:127,0,255:239 0/1:139,0,255:246 0/1:62,0,255:213 0/1:178,0,255:216 0/1:94,0,235:200 0/1:157,0,204:235

8 170635 . C T 999 . . GT:PL:DP 0/1:203,0,255:150 0/1:135,0,255:142 0/1:62,0,255:141 0/1:220,0,255:163 0/1:57,0,177:84 0/1:19,0,181:75 0/1:158,0,255:138

8 303083 . G A 999 . . GT:PL:DP 0/1:120,0,16:7 0/1:13,0,31:2 0/1:131,0,67:11 0/1:78,9,0:3 0/1:34,3,0:1 0/1:106,0,17:7 0/1:134,0,111:15

8 375249 . T C 999 . . GT:PL:DP 0/1:255,0,138:94 0/1:204,0,119:121 0/1:248,0,103:103 0/1:191,0,20:12 0/1:228,0,125:43 0/1:255,0,45:40 0/1:255,0,175:227

8 377143 . G A 999 . . GT:PL:DP 0/1:142,0,255:68 0/1:124,0,255:53 0/1:27,0,255:81 0/1:121,0,166:16 0/1:195,0,154:24 0/1:50,0,183:17 0/1:71,0,255:128

8 424954 . A T 999 . . GT:PL:DP 0/1:32,0,255:46 0/1:75,0,255:34 0/1:121,0,255:55 0/1:51,0,22:5 0/1:30,0,98:6 0/1:38,0,58:5 0/1:184,0,255:80

8 755089 . G T 999 . . GT:PL:DP 0/1:255,0,255:140 0/1:255,0,255:90 0/1:255,0,255:130 0/1:132,0,255:25 0/1:255,0,100:27 0/1:237,0,195:45 0/1:255,0,255:180

8 755244 . A T 999 . . GT:PL:DP 0/1:85,0,218:78 0/1:88,0,220:61 0/1:93,0,163:57 0/1:22,0,141:13 0/1:156,0,192:37 0/1:193,0,228:36 0/1:145,0,255:77

8 824202 . A G 999 . . GT:PL:DP 0/1:82,0,217:94 0/1:156,0,215:78 0/1:178,0,255:81 0/1:102,0,86:10 0/1:91,0,50:13 0/1:27,0,105:10 0/1:198,0,255:135

8 824701 . A G 999 . . GT:PL:DP 0/1:179,0,255:110 0/1:165,0,255:117 0/1:255,0,255:139 0/1:44,0,255:22 0/1:61,0,46:9 0/1:27,0,43:13 0/1:202,0,255:147

8 871814 . G T 999 . . GT:PL:DP 0/1:204,0,93:18 0/1:178,0,14:19 0/1:201,0,109:26 0/1:87,0,20:5 0/1:25,0,95:7 0/1:12,0,10:2 0/1:233,0,87:42

8 1011415 . T C 999 . . GT:PL:DP 0/1:200,0,215:27 0/1:216,0,255:42 0/1:174,0,255:28 0/1:37,0,54:4 0/1:36,3,0:1 0/1:89,9,0:3 0/1:255,0,255:43

8 1106577 . C A 999 . . GT:PL:DP 0/1:231,0,66:206 0/1:255,0,255:108 0/1:255,0,139:176 0/1:255,0,77:39 0/1:255,0,174:40 0/1:255,0,128:59 0/1:255,0,255:215

8 1126402 . T C 999 . . GT:PL:DP 0/1:137,0,245:47 0/1:65,0,218:43 0/1:52,0,255:44 0/1:23,0,92:5 0/1:37,0,46:8 0/1:24,4,0:4 0/1:152,0,255:65

8 1149286 . A G 999 . . GT:PL:DP 0/1:255,0,145:91 0/1:255,0,208:61 0/1:255,0,163:111 0/1:137,0,115:22 0/1:189,0,8:35 0/1:155,0,58:19 0/1:255,0,255:224

8 1315207 . G T 999 . . GT:PL:DP 0/1:199,0,17:15 0/1:186,0,46:21 0/1:255,0,17:31 0/1:30,0,9:4 0/1:112,0,54:10 0/1:96,0,48:8 0/1:238,0,157:50

8 1315208 . G T 999 . . GT:PL:DP 0/1:196,0,0:15 0/1:182,0,24:21 0/1:255,0,6:30 0/1:30,0,9:4 0/1:112,0,56:10 0/1:115,0,29:8 0/1:239,0,158:49

8 1315242 . C T 999 . . GT:PL:DP 0/1:225,0,46:18 0/1:227,0,54:19 0/1:255,0,61:33 0/1:88,0,9:4 0/1:77,0,73:8 0/1:126,0,16:8 0/1:255,0,167:57

8 1315293 . G A 999 . . GT:PL:DP 0/1:179,0,144:19 0/1:215,0,73:20 0/1:195,0,162:35 0/1:49,0,12:3 0/1:49,0,62:6 0/1:112,8,0:5 0/1:165,0,227:52

8 1315329 . G C 999 . . GT:PL:DP 0/1:120,0,177:21 0/1:141,0,158:20 0/1:96,0,232:36 0/1:51,9,0:3 0/1:22,0,81:6 0/1:41,0,22:5 0/1:127,0,213:45

8 1437367 . T A 999 . . GT:PL:DP 0/1:104,0,184:20 0/1:118,0,85:14 0/1:185,0,150:26 0/1:65,9,0:3 0/1:38,9,0:3 0/1:21,3,0:1 0/1:97,0,196:25

8 1590148 . A C 999 . . GT:PL:DP 0/1:255,0,255:224 0/1:255,0,255:208 0/1:255,0,255:222 0/1:255,0,255:49 0/1:255,0,224:92 0/1:255,0,255:182 0/1:255,0,255:207

8 1619362 . C T 999 . . GT:PL:DP 0/1:36,0,124:33 0/1:51,0,29:12 0/1:54,0,77:22 0/1:21,3,0:1 0/1:32,0,11:3 0/1:9,0,14:2 0/1:59,0,108:25

8 1619377 . C T 999 . . GT:PL:DP 0/1:61,0,42:12 0/1:53,0,69:14 0/1:51,0,24:15 0/1:12,0,20:3 0/1:41,6,0:2 0/1:15,3,0:1 0/1:54,0,62:17

8 1619383 . C A 999 . . GT:PL:DP 0/1:59,0,60:11 0/1:55,0,69:14 0/1:49,0,24:15 0/1:44,9,0:3 0/1:15,0,23:2 0/1:15,3,0:1 0/1:55,0,62:17

8 1619687 . G A 999 . . GT:PL:DP 0/1:100,0,255:218 0/1:169,0,244:176 0/1:76,0,247:221 0/1:184,0,201:32 0/1:114,0,193:71 0/1:52,0,193:71 0/1:164,0,255:221

8 1625691 . G A 999 . . GT:PL:DP 0/1:194,0,254:27 0/1:190,0,68:14 0/1:255,0,232:40 0/1:93,0,20:7 0/1:16,0,66:15 0/1:10,0,27:8 0/1:250,0,189:24

8 1748430 . C A 999 . . GT:PL:DP 0/1:255,0,224:46 0/1:100,0,255:36 0/1:255,0,255:49 0/1:67,1,0:4 0/1:123,0,92:13 0/1:196,0,255:36 0/1:255,0,255:89

8 1748471 . G A 999 . . GT:PL:DP 0/1:174,0,255:44 0/1:67,0,255:36 0/1:190,0,255:41 0/1:67,6,0:2 0/1:30,0,181:10 0/1:20,0,255:25 0/1:187,0,255:66

8 1769486 . T C 999 . . GT:PL:DP 0/1:255,0,255:177 0/1:255,0,255:207 0/1:255,0,255:202 0/1:139,0,181:21 0/1:193,0,146:35 0/1:159,0,41:19 0/1:158,0,255:194

8 1769545 . T C 999 . . GT:PL:DP 0/1:177,0,255:171 0/1:218,0,255:207 0/1:228,0,255:203 0/1:129,0,233:25 0/1:101,0,216:26 0/1:60,0,84:14 0/1:87,0,255:198

8 1770031 . G A 999 . . GT:PL:DP 0/1:251,0,255:154 0/1:255,0,255:159 0/1:170,0,255:175 0/1:36,0,255:27 0/1:75,0,133:19 0/1:11,0,21:8 0/1:19,0,255:187

8 1781763 . T C 999 . . GT:PL:DP 0/1:98,0,178:39 0/1:93,0,195:37 0/1:115,0,214:36 0/1:17,0,73:6 0/1:77,0,101:10 0/1:39,0,158:9 0/1:76,0,226:42

8 1799891 . T C 999 . . GT:PL:DP 0/1:72,0,255:88 0/1:55,0,255:107 0/1:13,0,255:107 0/1:98,0,46:13 0/1:20,0,63:4 0/1:45,0,89:5 0/1:114,0,255:213

8 1799895 . A T 999 . . GT:PL:DP 0/1:103,0,249:80 0/1:66,0,240:102 0/1:66,0,255:96 0/1:83,0,0:13 0/1:21,0,66:3 0/1:45,0,95:5 0/1:154,0,255:92

8 1999138 . G A 999 . . GT:PL:DP 0/1:157,0,118:14 0/1:158,0,101:18 0/1:93,0,137:10 0/1:31,0,31:2 0/1:64,6,0:2 0/1:94,0,130:10 0/1:118,0,152:17

8 2145843 . G A 999 . . GT:PL:DP 0/1:171,0,255:70 0/1:114,0,255:26 0/1:210,0,42:23 0/1:83,0,50:6 0/1:13,0,90:6 0/1:78,0,117:10 0/1:204,0,160:30

8 2145853 . G A 999 . . GT:PL:DP 0/1:137,0,255:60 0/1:122,0,255:25 0/1:205,0,44:22 0/1:85,0,22:5 0/1:11,0,87:6 0/1:80,0,113:10 0/1:200,0,161:26

8 2246406 . G T 999 . . GT:PL:DP 0/1:120,0,255:170 0/1:199,0,255:129 0/1:152,0,239:114 0/1:85,0,127:19 0/1:50,0,96:8 0/1:38,0,86:16 0/1:57,0,237:167

8 2286180 . C T 999 . . GT:PL:DP 0/1:224,0,255:80 0/1:255,0,255:51 0/1:213,0,255:55 0/1:208,0,119:16 0/1:23,0,53:6 0/1:182,0,152:16 0/1:255,0,250:117

8 2302054 . G A 999 . . GT:PL:DP 0/1:68,0,255:28 0/1:97,0,255:39 0/1:170,0,255:58 0/1:81,0,49:6 0/1:79,0,84:10 0/1:45,0,98:8 0/1:156,0,255:50

8 2366213 . A G 999 . . GT:PL:DP 0/1:202,0,242:126 0/1:224,0,255:146 0/1:133,0,227:132 0/1:109,0,184:19 0/1:108,0,64:32 0/1:26,0,197:42 0/1:245,0,255:174

8 2366361 . G A 999 . . GT:PL:DP 0/1:116,0,255:140 0/1:124,0,255:176 0/1:123,0,255:162 0/1:33,0,119:17 0/1:101,0,140:78 0/1:120,0,180:91 0/1:81,0,255:165

8 2366399 . G C 999 . . GT:PL:DP 0/1:92,0,255:130 0/1:115,0,255:145 0/1:115,0,225:141 0/1:37,0,78:15 0/1:111,0,134:66 0/1:164,0,246:100 0/1:97,0,255:136

8 2403794 . C T 999 . . GT:PL:DP 0/1:202,0,56:28 0/1:154,0,88:29 0/1:181,0,73:29 0/1:79,0,17:5 0/1:16,0,22:3 0/1:54,0,9:11 0/1:203,0,85:46

8 2424113 . C T 999 . . GT:PL:DP 0/1:255,0,255:204 0/1:255,0,254:205 0/1:255,0,255:195 0/1:255,0,255:135 0/1:189,0,107:194 0/1:248,0,162:215 0/1:255,0,255:204

8 2424187 . G C 999 . . GT:PL:DP 0/1:247,0,255:184 0/1:72,0,255:183 0/1:167,0,255:160 0/1:232,0,255:108 0/1:135,0,255:182 0/1:175,0,255:144 0/1:221,0,255:173

8 2424189 . T C 999 . . GT:PL:DP 0/1:249,0,255:182 0/1:90,0,255:181 0/1:175,0,255:156 0/1:240,0,255:106 0/1:136,0,255:184 0/1:179,0,255:145 0/1:255,0,255:172

8 2459446 . A T 999 . . GT:PL:DP 0/1:255,0,255:239 0/1:96,0,249:233 0/1:186,0,255:231 0/1:248,0,255:105 0/1:150,0,255:103 0/1:94,0,255:184 0/1:141,0,255:229

8 2459503 . C T 999 . . GT:PL:DP 0/1:196,0,255:238 0/1:255,0,255:237 0/1:255,0,255:234 0/1:221,0,255:79 0/1:252,0,255:101 0/1:31,0,209:174 0/1:255,0,255:232

8 2491883 . T C 999 . . GT:PL:DP 0/1:255,0,255:61 0/1:255,0,255:78 0/1:255,0,255:61 0/1:28,0,65:3 0/1:148,0,72:11 0/1:204,0,182:16 0/1:255,0,255:124

8 2492356 . G A 999 . . GT:PL:DP 0/1:129,0,255:112 0/1:241,0,255:99 0/1:179,0,255:110 0/1:171,0,218:17 0/1:10,0,255:21 0/1:62,0,152:16 0/1:255,0,255:191

8 2492392 . T C 999 . . GT:PL:DP 0/1:250,0,255:105 0/1:255,0,255:85 0/1:230,0,255:97 0/1:156,0,252:20 0/1:93,0,216:24 0/1:33,0,102:16 0/1:216,0,255:191

8 2492665 . T C 999 . . GT:PL:DP 0/1:191,0,188:61 0/1:126,0,57:36 0/1:167,0,136:41 0/1:70,0,19:8 0/1:142,0,52:10 0/1:75,0,44:8 0/1:187,0,142:95

8 2554600 . A G 999 . . GT:PL:DP 0/1:115,0,255:128 0/1:143,0,255:77 0/1:48,0,236:125 0/1:158,0,149:33 0/1:131,0,148:34 0/1:170,0,98:25 0/1:228,0,255:181

8 2558204 . G A 999 . . GT:PL:DP 0/1:239,0,246:207 0/1:92,2,0:212 0/1:224,0,112:211 0/1:145,0,163:53 0/1:32,6,0:9 0/1:51,0,33:20 0/1:255,0,255:179

8 2624208 . G T 999 . . GT:PL:DP 0/1:255,0,62:225 0/1:255,0,255:231 0/1:242,0,60:211 0/1:161,0,228:47 0/1:255,0,241:64 0/1:255,85,132:42 0/1:255,0,255:231

8 2624865 . T C 999 . . GT:PL:DP 0/1:255,0,255:214 0/1:255,0,255:221 0/1:255,0,120:165 0/1:255,0,198:28 0/1:255,0,196:48 0/1:176,0,255:35 0/1:255,0,255:238

8 2625015 . C T 999 . . GT:PL:DP 0/1:255,0,255:193 0/1:255,0,255:206 0/1:255,0,134:149 0/1:200,0,163:30 0/1:154,0,182:51 0/1:131,0,255:62 0/1:195,0,255:211

8 2705634 . G T 999 . . GT:PL:DP 0/1:122,0,57:68 0/1:192,0,255:165 0/1:242,0,242:92 0/1:194,0,137:27 0/1:52,0,133:10 0/1:203,0,178:25 0/1:255,0,255:138

8 2705709 . G C 999 . . GT:PL:DP 0/1:132,0,23:29 0/1:136,0,255:86 0/1:93,0,252:33 0/1:101,0,91:9 0/1:42,0,94:8 0/1:99,0,46:23 0/1:183,0,255:72

8 2706852 . A T 999 . . GT:PL:DP 0/1:133,0,239:23 0/1:84,16,7:4 0/1:246,0,139:32 0/1:23,0,93:4 0/1:75,9,0:3 0/1:46,6,0:2 0/1:110,0,231:30

8 2825362 . A G 999 . . GT:PL:DP 0/1:178,0,255:65 0/1:118,0,255:45 0/1:40,0,162:47 0/1:25,0,63:4 0/1:74,0,144:9 0/1:104,0,255:33 0/1:34,0,255:84

8 2851912 . C A 999 . . GT:PL:DP 0/1:193,0,124:19 0/1:255,0,181:30 0/1:230,0,104:27 0/1:135,0,82:10 0/1:58,6,0:2 0/1:30,3,0:1 0/1:255,0,89:52

8 2856860 . C T 999 . . GT:PL:DP 0/1:7,0,255:29 0/1:84,0,218:39 0/1:44,0,255:28 0/1:25,3,0:1 0/1:68,0,106:9 0/1:47,0,119:9 0/1:55,0,221:26

8 2940298 . T C 999 . . GT:PL:DP 0/1:161,0,134:31 0/1:59,0,149:12 0/1:176,0,250:34 0/1:165,0,4:9 0/1:53,6,0:2 0/1:37,0,6:5 0/1:169,0,220:34

8 2940318 . C T 999 . . GT:PL:DP 0/1:130,0,134:31 0/1:82,0,155:14 0/1:144,0,235:33 0/1:106,6,0:6 0/1:72,9,0:3 0/1:54,6,0:2 0/1:164,0,208:37

8 3022521 . G T 999 . . GT:PL:DP 0/1:151,0,47:261 0/1:213,0,136:248 0/1:255,0,203:260 0/1:244,0,117:53 0/1:255,0,153:111 0/1:255,0,255:114 0/1:167,0,58:322

8 3025421 . A T 999 . . GT:PL:DP 0/1:220,0,255:213 0/1:160,0,255:197 0/1:243,0,255:201 0/1:235,0,255:125 0/1:255,0,255:211 0/1:255,0,255:212 0/1:255,0,255:198

8 3025539 . C T 999 . . GT:PL:DP 0/1:244,0,255:216 0/1:255,0,255:197 0/1:246,0,255:198 0/1:255,0,255:144 0/1:255,0,255:164 0/1:255,0,255:172 0/1:255,0,255:180

8 3162040 . T A 999 . . GT:PL:DP 0/1:213,0,255:115 0/1:49,0,255:71 0/1:141,0,249:39 0/1:37,0,75:6 0/1:27,0,26:2 0/1:13,0,116:11 0/1:169,0,255:129

8 3170042 . A G 999 . . GT:PL:DP 0/1:166,0,201:140 0/1:158,0,204:161 0/1:166,0,169:139 0/1:137,0,234:90 0/1:246,0,147:98 0/1:212,0,149:93 0/1:185,0,147:120

8 3170200 . G A 999 . . GT:PL:DP 0/1:66,0,241:227 0/1:25,0,192:230 0/1:88,0,181:214 0/1:157,0,255:218 0/1:51,0,159:237 0/1:114,0,218:234 0/1:46,0,166:216

8 3210286 . A G 999 . . GT:PL:DP 0/1:145,0,139:50 0/1:106,0,151:53 0/1:154,0,153:54 0/1:65,0,11:7 0/1:49,0,79:10 0/1:36,3,0:1 0/1:160,0,182:50

8 3281351 . A G 999 . . GT:PL:DP 0/1:255,0,255:133 0/1:255,0,255:98 0/1:255,0,255:85 0/1:118,0,64:12 0/1:110,0,156:10 0/1:255,0,186:24 0/1:255,0,255:113

8 3305649 . A T 999 . . GT:PL:DP 0/1:139,0,187:127 0/1:106,0,45:123 0/1:99,0,59:119 0/1:149,0,110:121 0/1:61,0,92:24 0/1:128,0,188:72 0/1:83,0,85:129

8 3305700 . T C 999 . . GT:PL:DP 0/1:212,0,219:148 0/1:171,0,83:144 0/1:185,0,108:144 0/1:240,0,152:162 0/1:76,0,76:11 0/1:162,0,185:49 0/1:215,0,119:146

8 3305816 . C T 999 . . GT:PL:DP 0/1:71,0,141:102 0/1:102,0,156:98 0/1:101,0,116:100 0/1:186,0,85:61 0/1:72,0,2:15 0/1:67,0,22:12 0/1:119,0,98:104

8 3306132 . A T 999 . . GT:PL:DP 0/1:219,0,153:184 0/1:162,0,149:164 0/1:183,0,152:168 0/1:255,0,234:211 0/1:255,0,208:159 0/1:255,0,186:134 0/1:176,0,173:134

8 3306201 . T C 999 . . GT:PL:DP 0/1:221,0,111:197 0/1:202,0,147:192 0/1:166,0,173:181 0/1:255,0,182:219 0/1:255,0,255:223 0/1:255,0,255:207 0/1:189,0,161:163

8 3325378 . C G 999 . . GT:PL:DP 0/1:8,0,127:9 0/1:94,0,147:21 0/1:119,0,179:23 0/1:38,6,0:2 0/1:27,0,71:5 0/1:11,0,62:3 0/1:15,0,130:23

8 3378432 . T C 999 . . GT:PL:DP 0/1:67,0,255:79 0/1:55,0,255:64 0/1:44,0,255:77 0/1:26,0,200:18 0/1:138,0,26:17 0/1:38,0,116:13 0/1:71,0,255:86

8 3512130 . G C 999 . . GT:PL:DP 0/1:125,0,42:9 0/1:67,0,85:13 0/1:122,0,66:10 0/1:30,3,0:1 0/1:22,0,29:3 0/1:100,0,8:6 0/1:146,0,177:32

8 3552171 . A T 999 . . GT:PL:DP 0/1:175,0,170:28 0/1:230,0,147:41 0/1:188,0,237:37 0/1:33,0,116:7 0/1:26,0,85:6 0/1:48,6,0:2 0/1:215,0,214:54

8 3552742 . G A 999 . . GT:PL:DP 0/1:255,0,255:40 0/1:239,0,255:35 0/1:245,0,187:22 0/1:69,0,136:9 0/1:25,0,70:4 0/1:8,0,137:6 0/1:255,0,255:50

8 3552895 . C T 999 . . GT:PL:DP 0/1:255,0,255:36 0/1:255,0,255:43 0/1:255,0,255:42 0/1:46,0,90:5 0/1:22,0,85:5 0/1:47,0,55:4 0/1:255,0,255:56

8 3579113 . G C 999 . . GT:PL:DP 0/1:207,0,255:41 0/1:255,0,255:38 0/1:255,0,190:37 0/1:93,0,9:4 0/1:109,0,121:8 0/1:19,0,99:4 0/1:255,0,250:72

8 3579126 . T A 999 . . GT:PL:DP 0/1:240,0,255:37 0/1:255,0,255:43 0/1:255,0,213:37 0/1:37,0,28:3 0/1:99,0,143:9 0/1:53,0,65:4 0/1:255,0,251:65

8 3592380 . G C 999 . . GT:PL:DP 0/1:111,0,188:22 0/1:29,0,221:19 0/1:189,0,139:32 0/1:25,0,24:2 0/1:31,3,0:1 0/1:33,3,0:1 0/1:147,0,164:26

8 3612055 . G C 999 . . GT:PL:DP 0/1:93,0,205:25 0/1:138,0,20:18 0/1:110,0,216:38 0/1:25,3,0:1 0/1:28,0,45:4 0/1:34,0,81:7 0/1:114,0,248:39

8 3621544 . T G 999 . . GT:PL:DP 0/1:92,0,65:8 0/1:81,0,72:9 0/1:63,0,40:8 0/1:14,0,44:3 0/1:8,0,49:3 0/1:17,0,24:2 0/1:142,0,76:14

8 3626196 . T C 999 . . GT:PL:DP 0/1:109,0,243:67 0/1:66,0,224:66 0/1:113,0,214:82 0/1:41,0,57:7 0/1:50,10,0:6 0/1:69,9,0:3 0/1:115,0,218:140

8 3626291 . T A 999 . . GT:PL:DP 0/1:190,0,254:131 0/1:137,0,209:115 0/1:158,0,223:148 0/1:109,0,168:26 0/1:101,0,114:27 0/1:154,0,119:29 0/1:91,0,154:207

8 3659638 . T G 999 . . GT:PL:DP 0/1:50,0,220:235 0/1:249,0,255:234 0/1:109,0,255:231 0/1:205,0,255:163 0/1:190,0,255:125 0/1:178,0,255:111 0/1:198,0,255:238

8 3659809 . G T 999 . . GT:PL:DP 0/1:255,0,255:234 0/1:232,0,255:231 0/1:255,0,228:230 0/1:178,0,136:48 0/1:201,0,196:87 0/1:168,0,255:86 0/1:255,0,213:207

8 3660241 . G T 999 . . GT:PL:DP 0/1:165,0,255:154 0/1:116,0,255:178 0/1:179,0,255:154 0/1:255,0,255:134 0/1:110,0,255:94 0/1:117,0,255:143 0/1:255,0,255:161

8 3660242 . G A 999 . . GT:PL:DP 0/1:158,0,255:158 0/1:99,0,255:177 0/1:173,0,255:154 0/1:255,0,255:134 0/1:112,0,255:93 0/1:122,0,255:146 0/1:255,0,255:162

8 3660245 . C G 999 . . GT:PL:DP 0/1:255,0,255:102 0/1:250,0,255:136 0/1:106,0,243:100 0/1:76,0,255:82 0/1:173,0,255:73 0/1:133,0,255:120 0/1:177,0,255:94

8 3660250 . G A 999 . . GT:PL:DP 0/1:126,0,255:141 0/1:49,0,255:166 0/1:141,0,255:135 0/1:255,0,255:128 0/1:42,0,255:75 0/1:62,0,255:145 0/1:237,0,255:132

8 3660251 . A G 999 . . GT:PL:DP 0/1:124,0,255:140 0/1:49,0,255:165 0/1:149,0,255:132 0/1:255,0,255:126 0/1:74,0,255:76 0/1:74,0,255:147 0/1:231,0,255:132

8 3660426 . C T 999 . . GT:PL:DP 0/1:255,0,216:237 0/1:255,0,255:242 0/1:255,0,255:239 0/1:255,0,255:130 0/1:255,0,250:143 0/1:255,0,194:227 0/1:255,0,255:244

8 3660443 . T G 999 . . GT:PL:DP 0/1:142,0,255:241 0/1:255,0,255:242 0/1:153,0,255:226 0/1:200,0,255:137 0/1:201,0,255:181 0/1:79,0,255:221 0/1:208,0,255:237

8 3660582 . T G 999 . . GT:PL:DP 0/1:255,0,255:238 0/1:255,0,255:245 0/1:255,0,255:237 0/1:255,0,255:161 0/1:168,0,211:91 0/1:240,0,255:127 0/1:255,0,255:243

8 3660619 . G A 999 . . GT:PL:DP 0/1:255,0,255:239 0/1:255,0,255:243 0/1:255,0,255:238 0/1:255,0,255:166 0/1:201,0,211:78 0/1:242,0,255:96 0/1:255,0,255:238

8 3661006 . C T 999 . . GT:PL:DP 0/1:255,0,255:254 0/1:152,0,255:258 0/1:255,0,255:251 0/1:255,0,255:103 0/1:171,0,219:81 0/1:90,0,238:89 0/1:255,0,255:231

8 3690544 . G A 999 . . GT:PL:DP 0/1:140,0,255:30 0/1:73,0,247:23 0/1:98,0,196:21 0/1:5,0,57:3 0/1:18,3,0:1 0/1:30,3,0:1 0/1:107,0,255:26

8 3692173 . A G 999 . . GT:PL:DP 0/1:178,0,156:33 0/1:67,0,195:48 0/1:162,0,155:34 0/1:57,6,0:2 0/1:78,0,26:7 0/1:66,0,20:9 0/1:192,0,51:42

8 3798838 . T G 999 . . GT:PL:DP 0/1:157,0,5:16 0/1:224,0,30:33 0/1:167,0,13:13 0/1:58,6,0:2 0/1:39,6,0:2 0/1:97,5,0:6 0/1:194,0,26:24

8 3798840 . T A 999 . . GT:PL:DP 0/1:157,0,11:16 0/1:224,0,48:32 0/1:167,0,24:13 0/1:41,6,0:2 0/1:39,6,0:2 0/1:97,5,0:6 0/1:188,0,46:24

8 3798841 . A T 999 . . GT:PL:DP 0/1:157,0,19:16 0/1:223,0,62:31 0/1:140,0,45:12 0/1:58,6,0:2 0/1:39,6,0:2 0/1:97,5,0:6 0/1:188,0,41:23

8 3798842 . A T 999 . . GT:PL:DP 0/1:151,0,19:16 0/1:221,0,59:31 0/1:136,0,45:12 0/1:58,6,0:2 0/1:39,6,0:2 0/1:97,5,0:6 0/1:187,0,23:22

8 3798843 . A T 999 . . GT:PL:DP 0/1:157,0,17:16 0/1:221,0,16:28 0/1:141,0,45:12 0/1:41,6,0:2 0/1:39,6,0:2 0/1:97,5,0:6 0/1:187,0,15:22

8 3813378 . C A 999 . . GT:PL:DP 0/1:255,0,4:87 0/1:241,0,80:84 0/1:229,0,57:84 0/1:115,0,50:11 0/1:37,0,114:19 0/1:164,0,84:22 0/1:255,8,0:112

8 3813388 . A T 999 . . GT:PL:DP 0/1:255,0,36:85 0/1:235,0,74:83 0/1:238,0,25:75 0/1:91,0,50:11 0/1:12,0,127:19 0/1:171,0,82:21 0/1:255,0,9:111

8 3834462 . C G 999 . . GT:PL:DP 0/1:97,0,189:34 0/1:198,0,145:63 0/1:152,0,179:51 0/1:28,0,28:3 0/1:77,0,42:9 0/1:29,0,41:3 0/1:255,0,206:64

8 3944798 . T C 999 . . GT:PL:DP 0/1:55,0,58:9 0/1:15,0,45:7 0/1:67,0,40:8 0/1:53,0,15:3 0/1:20,0,43:4 0/1:16,0,14:3 0/1:58,0,90:14

8 3944800 . T A 999 . . GT:PL:DP 0/1:110,60,51:12 0/1:72,47,44:7 0/1:112,47,38:8 0/1:73,21,15:3 0/1:20,0,37:4 0/1:49,17,11:4 0/1:150,109,103:13

8 3967767 . A T 999 . . GT:PL:DP 0/1:30,0,215:29 0/1:119,0,38:15 0/1:89,0,230:37 0/1:14,0,57:6 0/1:48,0,44:7 0/1:79,0,5:7 0/1:183,0,42:38

8 4094785 . G T 999 . . GT:PL:DP 0/1:30,0,109:20 0/1:207,0,136:33 0/1:99,0,142:33 0/1:21,0,65:4 0/1:36,0,60:6 0/1:62,0,44:5 0/1:97,0,175:35

8 4124243 . C A 999 . . GT:PL:DP 0/1:126,0,255:235 0/1:74,0,241:248 0/1:213,0,255:243 0/1:255,0,255:202 0/1:215,0,255:207 0/1:84,0,255:229 0/1:126,0,255:232

8 4195431 . G A 999 . . GT:PL:DP 0/1:101,0,255:75 0/1:52,0,61:85 0/1:112,0,206:102 0/1:9,0,31:2 0/1:14,0,77:9 0/1:17,0,129:7 0/1:130,0,217:101

8 4195889 . T A 999 . . GT:PL:DP 0/1:199,0,236:68 0/1:219,0,205:73 0/1:40,0,199:74 0/1:75,0,107:11 0/1:28,0,86:10 0/1:48,0,86:14 0/1:31,0,153:51

8 4195890 . G A 999 . . GT:PL:DP 0/1:235,0,210:86 0/1:255,0,178:91 0/1:142,0,230:86 0/1:73,0,109:14 0/1:28,0,98:13 0/1:65,0,85:15 0/1:78,0,171:62

8 4195956 . A C 999 . . GT:PL:DP 0/1:174,0,247:285 0/1:194,0,186:265 0/1:194,0,233:286 0/1:10,0,121:90 0/1:7,0,251:69 0/1:169,0,225:98 0/1:164,0,197:295

8 4243190 . C T 999 . . GT:PL:DP 0/1:255,0,104:54 0/1:255,0,176:53 0/1:255,0,152:43 0/1:191,0,11:11 0/1:47,0,183:16 0/1:160,0,220:22 0/1:255,0,185:56

8 4314749 . G A 999 . . GT:PL:DP 0/1:139,0,113:35 0/1:211,0,94:39 0/1:229,0,108:41 0/1:22,0,118:11 0/1:144,0,54:13 0/1:40,0,22:7 0/1:182,0,203:68

8 4317609 . A C 999 . . GT:PL:DP 0/1:94,0,205:30 0/1:24,0,84:12 0/1:12,0,145:13 0/1:44,0,41:5 0/1:44,0,199:12 0/1:30,0,85:6 0/1:11,0,95:11

8 4335803 . C T 999 . . GT:PL:DP 0/1:88,0,60:17 0/1:57,0,5:7 0/1:87,0,37:17 0/1:17,0,17:3 0/1:67,6,0:2 0/1:36,0,5:4 0/1:56,0,130:18

8 4343643 . A G 999 . . GT:PL:DP 0/1:42,0,152:9 0/1:146,0,155:23 0/1:83,0,53:9 0/1:69,0,20:4 0/1:43,0,42:6 0/1:67,0,198:20 0/1:141,0,255:66

8 4379621 . C T 999 . . GT:PL:DP 0/1:255,0,221:35 0/1:255,0,255:44 0/1:255,0,234:33 0/1:72,6,0:2 0/1:55,0,33:3 0/1:51,0,64:4 0/1:255,0,142:36

8 4447975 . C T 999 . . GT:PL:DP 0/1:108,0,10:57 0/1:110,0,78:51 0/1:114,0,54:56 0/1:7,0,49:10 0/1:52,28,22:5 0/1:19,0,31:8 0/1:127,0,75:101

8 4583932 . T G 999 . . GT:PL:DP 0/1:82,0,231:23 0/1:39,0,180:12 0/1:37,0,177:17 0/1:59,0,17:4 0/1:46,0,77:6 0/1:20,0,91:6 0/1:150,0,118:21

8 4605892 . T G 999 . . GT:PL:DP 0/1:147,0,255:54 0/1:142,0,255:49 0/1:235,0,208:50 0/1:55,0,96:7 0/1:101,0,110:13 0/1:115,0,40:8 0/1:250,0,218:95

8 4606008 . T A 999 . . GT:PL:DP 0/1:255,0,226:56 0/1:255,0,114:51 0/1:234,0,207:44 0/1:22,0,65:5 0/1:16,0,50:6 0/1:47,0,57:6 0/1:255,0,201:95

8 4623154 . A T 999 . . GT:PL:DP 0/1:255,0,255:195 0/1:255,0,255:182 0/1:255,0,255:193 0/1:230,0,255:57 0/1:242,0,126:34 0/1:255,0,204:59 0/1:255,0,255:166

8 4625467 . G A 999 . . GT:PL:DP 0/1:96,0,255:74 0/1:75,0,255:64 0/1:80,0,255:47 0/1:47,0,140:10 0/1:54,0,138:20 0/1:57,0,83:10 0/1:167,0,255:71

8 4626107 . T A 999 . . GT:PL:DP 0/1:130,0,226:50 0/1:115,0,252:34 0/1:68,0,255:38 0/1:15,0,26:2 0/1:65,0,201:16 0/1:89,0,190:12 0/1:102,0,255:53

8 4626313 . A T 999 . . GT:PL:DP 0/1:55,0,255:63 0/1:47,0,255:28 0/1:144,0,255:46 0/1:77,0,81:8 0/1:27,0,251:17 0/1:69,0,58:6 0/1:203,0,255:72

8 4628738 . A G 999 . . GT:PL:DP 0/1:255,0,218:58 0/1:255,0,210:45 0/1:207,0,255:43 0/1:100,0,97:8 0/1:133,0,146:13 0/1:124,0,255:19 0/1:255,0,255:77

8 4632295 . G C 999 . . GT:PL:DP 0/1:255,0,215:81 0/1:255,0,255:58 0/1:155,0,255:54 0/1:106,0,22:5 0/1:106,0,22:8 0/1:7,0,255:19 0/1:255,0,255:71

8 4632296 . G C 999 . . GT:PL:DP 0/1:255,0,221:80 0/1:255,0,255:59 0/1:155,0,255:54 0/1:111,0,22:5 0/1:105,0,18:7 0/1:56,0,255:22 0/1:255,0,255:72

8 4632297 . T C 999 . . GT:PL:DP 0/1:255,0,204:81 0/1:255,0,255:59 0/1:157,0,255:54 0/1:96,0,22:5 0/1:108,0,10:7 0/1:26,0,255:22 0/1:255,0,255:73

8 4675268 . T G 999 . . GT:PL:DP 0/1:119,0,201:32 0/1:156,0,249:38 0/1:97,0,253:44 0/1:95,0,37:9 0/1:19,2,0:4 0/1:61,0,40:6 0/1:142,0,255:89

8 4675356 . C T 999 . . GT:PL:DP 0/1:241,0,223:39 0/1:142,0,255:44 0/1:198,0,255:58 0/1:90,0,20:7 0/1:28,0,18:4 0/1:27,3,0:1 0/1:231,0,244:84

8 4708504 . G A 999 . . GT:PL:DP 0/1:133,0,205:98 0/1:177,0,205:175 0/1:165,0,193:180 0/1:51,0,63:12 0/1:27,0,133:17 0/1:44,0,85:39 0/1:134,0,150:162

8 4714766 . T C 999 . . GT:PL:DP 0/1:255,0,144:257 0/1:196,0,23:249 0/1:230,0,46:231 0/1:255,0,203:73 0/1:90,0,190:45 0/1:51,0,198:58 0/1:255,0,255:224

8 4714776 . G A 999 . . GT:PL:DP 0/1:255,0,255:244 0/1:255,0,197:246 0/1:255,0,255:217 0/1:255,0,255:73 0/1:36,0,214:44 0/1:97,0,201:59 0/1:255,0,255:221

8 4714810 . A G 999 . . GT:PL:DP 0/1:255,0,221:149 0/1:255,0,129:138 0/1:255,0,178:163 0/1:255,0,255:56 0/1:25,0,184:39 0/1:81,0,189:52 0/1:255,0,255:188

8 4714908 . A T 999 . . GT:PL:DP 0/1:196,0,84:133 0/1:179,0,0:120 0/1:209,0,145:138 0/1:183,0,145:28 0/1:91,9,0:3 0/1:12,0,30:2 0/1:243,0,220:140

8 4715338 . T A 999 . . GT:PL:DP 0/1:112,0,94:52 0/1:117,0,76:27 0/1:105,0,126:24 0/1:77,0,60:11 0/1:25,6,0:2 0/1:31,0,11:3 0/1:118,0,97:52

8 4780316 . C T 999 . . GT:PL:DP 0/1:255,0,208:45 0/1:234,0,255:70 0/1:255,0,255:63 0/1:115,0,100:15 0/1:96,0,238:18 0/1:108,0,106:10 0/1:255,0,255:59

8 4780321 . T C 999 . . GT:PL:DP 0/1:254,0,213:40 0/1:255,0,255:68 0/1:255,0,255:62 0/1:173,0,85:15 0/1:101,0,206:16 0/1:110,0,84:9 0/1:254,0,255:56

8 4780331 . G A 999 . . GT:PL:DP 0/1:240,0,228:40 0/1:255,0,255:59 0/1:255,0,255:60 0/1:187,0,96:17 0/1:80,0,205:15 0/1:116,0,82:10 0/1:255,0,255:54

8 4808931 . A G 999 . . GT:PL:DP 0/1:57,0,82:8 0/1:64,0,145:17 0/1:95,0,156:14 0/1:67,6,0:2 0/1:39,6,0:2 0/1:24,0,31:2 0/1:120,0,150:22

8 4822996 . C T 999 . . GT:PL:DP 0/1:112,0,194:87 0/1:125,0,224:62 0/1:140,0,255:123 0/1:107,0,129:17 0/1:89,0,8:10 0/1:44,9,0:3 0/1:63,0,255:125

8 4823828 . C G 999 . . GT:PL:DP 0/1:255,0,251:72 0/1:238,0,71:81 0/1:255,0,255:95 0/1:61,0,215:16 0/1:85,0,79:15 0/1:135,0,1:11 0/1:254,0,255:125

8 4824115 . G A 999 . . GT:PL:DP 0/1:252,0,240:102 0/1:198,0,17:108 0/1:255,0,203:100 0/1:173,0,27:14 0/1:48,0,36:9 0/1:20,0,66:14 0/1:223,0,174:150

8 4824179 . A G 999 . . GT:PL:DP 0/1:228,0,172:69 0/1:119,0,94:71 0/1:149,0,233:82 0/1:107,0,51:13 0/1:40,0,26:7 0/1:33,0,14:8 0/1:223,0,245:94

8 4893684 . C T 999 . . GT:PL:DP 0/1:96,0,120:17 0/1:123,0,139:24 0/1:129,0,82:14 0/1:64,0,17:4 0/1:15,0,31:2 0/1:67,0,93:7 0/1:144,0,122:23

8 4900880 . A G 999 . . GT:PL:DP 0/1:91,0,79:34 0/1:163,0,217:58 0/1:140,0,167:55 0/1:89,0,32:13 0/1:35,6,0:2 0/1:29,2,0:4 0/1:171,0,127:82

8 4902382 . G A 999 . . GT:PL:DP 0/1:255,0,247:73 0/1:255,0,109:51 0/1:255,0,250:61 0/1:136,0,159:17 0/1:196,0,9:18 0/1:74,0,48:9 0/1:255,0,255:172

8 4902452 . A G 999 . . GT:PL:DP 0/1:255,0,140:46 0/1:218,0,197:45 0/1:219,0,251:47 0/1:62,0,162:11 0/1:143,0,110:16 0/1:8,0,86:8 0/1:255,0,255:132

8 5045234 . C G 999 . . GT:PL:DP 0/1:199,0,79:17 0/1:135,0,65:17 0/1:248,0,99:32 0/1:101,0,18:5 0/1:99,9,0:3 0/1:31,3,0:1 0/1:254,0,172:38

8 5045238 . C A 999 . . GT:PL:DP 0/1:205,0,79:17 0/1:114,0,68:16 0/1:211,0,99:32 0/1:101,0,18:5 0/1:99,9,0:3 0/1:31,3,0:1 0/1:250,0,155:36

8 5047369 . T G 999 . . GT:PL:DP 0/1:133,0,88:28 0/1:108,0,103:29 0/1:103,0,80:20 0/1:49,0,17:4 0/1:21,0,10:3 0/1:21,0,1:3 0/1:95,0,107:44

8 5055797 . A C 999 . . GT:PL:DP 0/1:201,0,143:29 0/1:252,0,49:37 0/1:133,0,54:22 0/1:39,0,25:4 0/1:50,9,0:3 0/1:60,6,0:2 0/1:89,0,122:33

8 5056393 . A T 999 . . GT:PL:DP 0/1:102,0,50:13 0/1:67,0,90:9 0/1:91,0,178:16 0/1:22,3,0:1 0/1:63,9,0:3 0/1:9,0,55:4 0/1:45,0,105:15

8 5129668 . G A 999 . . GT:PL:DP 0/1:254,0,255:67 0/1:167,0,255:113 0/1:223,0,255:153 0/1:162,0,255:31 0/1:124,0,255:25 0/1:113,0,255:38 0/1:255,0,255:141

8 5131399 . T C 999 . . GT:PL:DP 0/1:185,0,181:32 0/1:10,0,147:22 0/1:141,0,189:25 0/1:49,0,45:4 0/1:10,0,58:8 0/1:115,0,147:26 0/1:255,0,132:63

8 5210465 . A T 999 . . GT:PL:DP 0/1:177,0,50:29 0/1:104,0,138:20 0/1:93,0,86:15 0/1:20,3,0:1 0/1:19,6,0:2 0/1:24,0,37:5 0/1:19,0,98:13

8 5256419 . C T 999 . . GT:PL:DP 0/1:197,0,255:41 0/1:175,0,255:33 0/1:255,0,255:37 0/1:17,0,134:6 0/1:67,0,85:6 0/1:51,0,73:5 0/1:255,0,255:39

8 5287622 . T A 999 . . GT:PL:DP 0/1:60,0,110:14 0/1:121,0,229:29 0/1:78,0,157:21 0/1:17,3,0:1 0/1:59,9,0:3 0/1:30,6,0:2 0/1:191,0,204:55

8 5333572 . G T 999 . . GT:PL:DP 0/1:151,0,221:48 0/1:174,0,195:37 0/1:122,0,202:33 0/1:18,0,78:5 0/1:74,0,39:8 0/1:52,0,232:23 0/1:106,0,206:22

8 5448731 . T A 999 . . GT:PL:DP 0/1:9,0,9:3 0/1:79,0,81:12 0/1:86,0,25:10 0/1:28,0,0:3 0/1:17,3,0:1 0/1:14,0,57:10 0/1:68,0,32:11

8 5486512 . G A 999 . . GT:PL:DP 0/1:197,0,148:18 0/1:255,0,211:41 0/1:255,0,255:41 0/1:23,0,100:5 0/1:61,0,54:5 0/1:39,3,0:1 0/1:255,0,220:35

8 5487174 . C G 999 . . GT:PL:DP 0/1:75,0,136:23 0/1:128,0,80:25 0/1:11,0,208:27 0/1:25,0,5:4 0/1:54,0,3:7 0/1:28,0,17:3 0/1:126,0,68:27

8 5556770 . A T 999 . . GT:PL:DP 0/1:160,0,198:32 0/1:255,0,75:33 0/1:180,0,235:40 0/1:59,0,20:6 0/1:70,0,4:5 0/1:35,0,43:8 0/1:126,0,255:54

8 5687674 . T C 999 . . GT:PL:DP 0/1:255,0,206:35 0/1:255,0,255:46 0/1:255,0,255:45 0/1:111,0,84:7 0/1:104,0,97:8 0/1:113,0,120:8 0/1:255,0,255:51

8 5724987 . C T 999 . . GT:PL:DP 0/1:188,0,122:55 0/1:255,0,117:82 0/1:220,0,161:77 0/1:114,0,79:13 0/1:116,0,3:12 0/1:53,0,76:8 0/1:253,0,225:149

8 5725060 . G A 999 . . GT:PL:DP 0/1:210,0,86:145 0/1:226,0,255:155 0/1:232,0,255:143 0/1:138,0,129:22 0/1:118,0,110:28 0/1:108,0,83:13 0/1:123,0,255:226

8 5725097 . A C 999 . . GT:PL:DP 0/1:201,0,14:162 0/1:255,0,253:170 0/1:255,0,255:169 0/1:210,0,110:21 0/1:126,0,144:35 0/1:134,0,140:19 0/1:255,0,255:218

8 5730180 . G T 999 . . GT:PL:DP 0/1:176,0,171:292 0/1:157,0,138:302 0/1:130,0,124:309 0/1:135,0,115:102 0/1:96,0,92:170 0/1:52,0,12:112 0/1:187,0,163:283

8 5730619 . G A 999 . . GT:PL:DP 0/1:255,0,255:235 0/1:255,0,255:237 0/1:255,0,255:240 0/1:255,0,255:127 0/1:255,0,255:171 0/1:255,0,252:63 0/1:255,0,255:233

8 5730665 . A C 999 . . GT:PL:DP 0/1:255,0,255:237 0/1:255,0,255:235 0/1:255,0,255:237 0/1:255,0,255:126 0/1:157,0,255:71 0/1:121,0,79:23 0/1:255,0,249:228

8 5730799 . A T 999 . . GT:PL:DP 0/1:98,0,249:231 0/1:186,0,255:241 0/1:98,0,248:245 0/1:126,0,255:193 0/1:25,0,241:115 0/1:21,0,211:49 0/1:29,0,243:236

8 5737596 . G T 999 . . GT:PL:DP 0/1:219,0,241:35 0/1:155,0,209:22 0/1:223,0,255:47 0/1:77,0,46:5 0/1:67,6,0:2 0/1:37,3,0:1 0/1:212,0,255:41

8 5756602 . T A 999 . . GT:PL:DP 0/1:96,0,107:25 0/1:118,0,56:17 0/1:126,0,143:24 0/1:29,0,27:2 0/1:63,9,0:3 0/1:19,3,0:1 0/1:14,0,205:23

8 5839819 . G A 999 . . GT:PL:DP 0/1:255,0,217:37 0/1:255,0,247:42 0/1:93,0,243:21 0/1:126,0,30:9 0/1:50,0,52:4 0/1:142,0,36:8 0/1:255,0,242:41

8 5889735 . T A 999 . . GT:PL:DP 0/1:255,0,255:53 0/1:214,0,255:32 0/1:255,0,255:46 0/1:97,0,50:6 0/1:135,12,0:4 0/1:19,0,122:6 0/1:255,0,255:60

8 5944119 . C G 999 . . GT:PL:DP 0/1:96,0,16:17 0/1:96,0,27:31 0/1:101,0,82:21 0/1:28,6,0:2 0/1:15,0,27:4 0/1:28,0,36:2 0/1:176,0,43:18

8 6004714 . G C 999 . . GT:PL:DP 0/1:238,0,230:38 0/1:187,0,255:34 0/1:255,0,244:42 0/1:60,0,161:10 0/1:94,0,82:10 0/1:68,0,7:6 0/1:193,0,255:47

8 6088609 . A G 999 . . GT:PL:DP 0/1:150,0,24:13 0/1:148,5,0:11 0/1:193,0,12:13 0/1:77,9,0:3 0/1:78,0,39:7 0/1:86,9,0:3 0/1:255,0,9:37

8 6104426 . T C 999 . . GT:PL:DP 0/1:255,0,255:34 0/1:255,0,255:34 0/1:245,0,255:34 0/1:112,0,21:5 0/1:69,0,82:6 0/1:30,3,0:1 0/1:255,0,218:40

8 6168258 . G A 999 . . GT:PL:DP 0/1:180,0,255:40 0/1:138,0,245:27 0/1:211,0,162:23 0/1:120,0,71:9 0/1:75,9,0:3 0/1:84,0,50:5 0/1:218,0,255:42

8 6225417 . A T 999 . . GT:PL:DP 0/1:225,0,164:27 0/1:215,0,142:27 0/1:116,0,198:26 0/1:12,0,116:7 0/1:25,0,21:2 0/1:25,0,23:2 0/1:99,0,240:34

8 6225965 . G A 999 . . GT:PL:DP 0/1:222,0,255:34 0/1:237,0,255:32 0/1:253,0,255:35 0/1:92,9,0:3 0/1:19,0,105:5 0/1:81,0,107:7 0/1:255,0,255:47

8 6305387 . T C 999 . . GT:PL:DP 0/1:115,0,36:21 0/1:205,0,27:21 0/1:99,0,50:22 0/1:30,0,8:2 0/1:55,9,0:3 0/1:66,9,0:3 0/1:130,0,33:25

8 6493377 . A G 999 . . GT:PL:DP 0/1:203,0,235:220 0/1:90,0,194:198 0/1:152,0,220:221 0/1:163,0,255:94 0/1:138,0,136:133 0/1:26,0,17:164 0/1:223,0,255:233

8 6493492 . G T 999 . . GT:PL:DP 0/1:122,0,233:233 0/1:48,0,179:236 0/1:136,0,255:228 0/1:130,0,255:93 0/1:29,0,88:58 0/1:108,0,81:70 0/1:213,0,255:235

8 6566815 . A G 999 . . GT:PL:DP 0/1:55,0,123:15 0/1:211,0,222:33 0/1:215,0,181:31 0/1:36,0,68:5 0/1:31,0,23:2 0/1:92,9,0:3 0/1:172,0,255:35

8 6726884 . C T 999 . . GT:PL:DP 0/1:211,0,161:33 0/1:194,0,121:33 0/1:98,0,122:20 0/1:59,0,9:3 0/1:25,0,15:3 0/1:8,0,85:9 0/1:149,0,208:44

8 6734408 . G A 999 . . GT:PL:DP 0/1:236,0,255:138 0/1:173,0,73:185 0/1:220,0,128:154 0/1:155,0,154:28 0/1:27,0,16:2 0/1:122,0,31:11 0/1:227,0,255:157

8 6734410 . G A 999 . . GT:PL:DP 0/1:223,0,238:134 0/1:168,0,53:185 0/1:219,0,128:154 0/1:154,0,156:27 0/1:27,0,20:2 0/1:123,0,7:10 0/1:219,0,255:157

8 6740098 . A T 999 . . GT:PL:DP 0/1:168,0,255:39 0/1:241,0,244:35 0/1:210,0,250:36 0/1:22,0,84:5 0/1:17,0,46:3 0/1:17,0,50:3 0/1:255,0,255:54

8 6740104 . G A 999 . . GT:PL:DP 0/1:165,0,255:38 0/1:235,0,252:36 0/1:206,0,251:35 0/1:20,0,84:5 0/1:36,0,28:3 0/1:17,0,62:3 0/1:255,0,255:50

8 6845398 . A T 999 . . GT:PL:DP 0/1:162,0,254:35 0/1:204,0,255:45 0/1:205,0,200:33 0/1:13,0,66:4 0/1:58,6,0:2 0/1:35,0,39:5 0/1:95,0,184:21

8 6862673 . C G 999 . . GT:PL:DP 0/1:73,0,228:22 0/1:255,0,169:61 0/1:255,0,255:53 0/1:203,0,19:11 0/1:138,0,168:14 0/1:245,0,143:20 0/1:255,0,55:189

8 6862740 . T C 999 . . GT:PL:DP 0/1:17,0,209:19 0/1:255,64,81:60 0/1:255,0,255:58 0/1:214,0,61:11 0/1:136,0,20:7 0/1:176,0,255:21 0/1:255,0,35:165

8 6887209 . C A 999 . . GT:PL:DP 0/1:255,0,255:169 0/1:169,0,255:155 0/1:231,0,255:136 0/1:19,0,254:20 0/1:158,0,145:16 0/1:83,0,199:16 0/1:207,0,255:181

8 6887210 . A T 999 . . GT:PL:DP 0/1:253,0,255:168 0/1:158,0,255:150 0/1:237,0,255:135 0/1:8,0,254:20 0/1:137,0,165:17 0/1:80,0,238:17 0/1:214,0,255:178

8 6949446 . C G 999 . . GT:PL:DP 0/1:255,0,255:190 0/1:255,0,255:186 0/1:255,0,255:208 0/1:255,0,255:128 0/1:255,0,255:205 0/1:255,0,255:155 0/1:217,0,255:196

8 6949484 . C T 999 . . GT:PL:DP 0/1:255,0,255:183 0/1:255,0,255:196 0/1:255,0,255:207 0/1:255,0,255:132 0/1:255,0,255:198 0/1:248,0,255:135 0/1:208,0,255:189

8 6995079 . A G 999 . . GT:PL:DP 0/1:222,0,189:21 0/1:207,0,193:22 0/1:255,0,153:30 0/1:94,9,0:3 0/1:7,0,62:3 0/1:26,0,31:2 0/1:255,0,255:37

8 7189458 . G C 999 . . GT:PL:DP 0/1:255,0,82:49 0/1:255,0,70:56 0/1:255,0,101:61 0/1:191,0,7:10 0/1:207,0,129:26 0/1:49,0,139:37 0/1:255,0,183:81

8 7216666 . G C 999 . . GT:PL:DP 0/1:163,0,209:74 0/1:125,0,218:67 0/1:170,0,183:70 0/1:56,0,34:10 0/1:11,0,48:21 0/1:34,0,15:12 0/1:248,0,178:132

8 7249758 . A G 999 . . GT:PL:DP 0/1:197,0,255:36 0/1:255,0,255:39 0/1:249,0,255:35 0/1:62,0,56:4 0/1:56,0,88:5 0/1:95,0,182:11 0/1:255,0,255:46

8 7249893 . A T 999 . . GT:PL:DP 0/1:255,0,158:29 0/1:255,0,255:48 0/1:161,0,255:25 0/1:41,0,62:4 0/1:93,9,0:3 0/1:46,0,73:5 0/1:255,0,255:33

8 7252261 . C G 999 . . GT:PL:DP 0/1:247,0,255:33 0/1:255,0,255:35 0/1:255,0,255:50 0/1:87,0,91:6 0/1:81,0,106:8 0/1:157,0,181:14 0/1:255,0,173:32

8 7460975 . A T 999 . . GT:PL:DP 0/1:19,0,14:2 0/1:163,0,14:10 0/1:129,0,54:11 0/1:30,3,0:1 0/1:41,6,0:2 0/1:42,6,0:2 0/1:153,0,52:14

8 7509929 . T G 999 . . GT:PL:DP 0/1:135,0,130:20 0/1:109,0,221:24 0/1:151,0,228:32 0/1:33,6,0:2 0/1:38,0,53:6 0/1:13,0,52:3 0/1:191,0,203:28

8 7569189 . A G 999 . . GT:PL:DP 0/1:255,0,255:223 0/1:235,0,255:219 0/1:254,0,255:235 0/1:255,0,255:119 0/1:162,0,200:59 0/1:73,0,192:72 0/1:239,0,255:232

8 7587044 . T C 999 . . GT:PL:DP 0/1:255,0,255:106 0/1:255,0,255:109 0/1:255,0,255:95 0/1:105,0,152:12 0/1:162,0,153:22 0/1:117,0,209:31 0/1:255,0,255:245

8 7587413 . A G 999 . . GT:PL:DP 0/1:255,0,196:149 0/1:255,0,255:165 0/1:255,0,251:135 0/1:177,0,205:18 0/1:253,0,179:23 0/1:255,0,223:31 0/1:255,0,255:217

8 7633451 . A G 999 . . GT:PL:DP 0/1:130,0,255:247 0/1:195,0,255:237 0/1:109,0,250:243 0/1:255,0,233:110 0/1:62,0,116:16 0/1:249,0,255:76 0/1:207,0,255:221

8 7700355 . G A 999 . . GT:PL:DP 0/1:255,0,147:50 0/1:255,0,151:50 0/1:188,0,255:32 0/1:136,0,48:7 0/1:60,0,74:7 0/1:9,0,141:9 0/1:255,0,255:68

8 7702052 . T C 999 . . GT:PL:DP 0/1:255,0,145:34 0/1:255,0,145:35 0/1:255,0,30:48 0/1:174,0,66:13 0/1:109,0,19:6 0/1:89,0,88:7 0/1:194,0,208:38

8 7704362 . G A 999 . . GT:PL:DP 0/1:237,0,243:36 0/1:141,0,235:36 0/1:126,0,255:43 0/1:101,0,254:19 0/1:21,0,189:10 0/1:59,0,57:5 0/1:159,0,255:62

8 7704391 . T C 999 . . GT:PL:DP 0/1:143,0,255:44 0/1:99,0,208:42 0/1:85,0,255:48 0/1:87,0,241:18 0/1:22,0,129:8 0/1:11,0,64:4 0/1:147,0,255:63

8 7730462 . G A 999 . . GT:PL:DP 0/1:176,0,121:62 0/1:202,0,131:79 0/1:131,0,127:61 0/1:105,0,49:13 0/1:64,0,106:22 0/1:87,0,166:36 0/1:158,0,158:115

8 7730518 . C T 999 . . GT:PL:DP 0/1:217,0,128:117 0/1:227,0,146:141 0/1:130,0,190:111 0/1:135,0,46:18 0/1:68,0,63:17 0/1:91,0,159:31 0/1:255,0,222:179

8 7730640 . A T 999 . . GT:PL:DP 0/1:255,0,255:205 0/1:222,0,255:201 0/1:255,0,255:175 0/1:132,0,198:25 0/1:14,0,242:34 0/1:168,0,255:125 0/1:255,0,255:194

8 7730742 . C T 999 . . GT:PL:DP 0/1:200,0,255:213 0/1:161,0,255:208 0/1:165,0,255:205 0/1:135,0,255:30 0/1:255,0,246:54 0/1:255,0,255:136 0/1:234,0,255:206

8 7731603 . G T 999 . . GT:PL:DP 0/1:255,0,238:211 0/1:255,0,255:214 0/1:255,0,255:214 0/1:167,0,144:20 0/1:144,0,194:38 0/1:109,0,255:88 0/1:255,0,255:210

8 7810382 . G A 999 . . GT:PL:DP 0/1:148,0,146:38 0/1:147,0,147:51 0/1:160,0,147:53 0/1:107,0,15:8 0/1:26,0,68:6 0/1:12,0,93:5 0/1:162,0,141:56

8 7889997 . T G 999 . . GT:PL:DP 0/1:124,0,255:50 0/1:110,0,255:51 0/1:126,0,255:57 0/1:15,0,159:9 0/1:75,0,87:6 0/1:53,0,224:18 0/1:119,0,255:42

8 7890000 . T G 999 . . GT:PL:DP 0/1:71,0,255:46 0/1:82,0,255:49 0/1:92,0,255:55 0/1:15,0,133:9 0/1:42,0,72:5 0/1:19,0,217:15 0/1:37,0,255:43

8 7992288 . T C 999 . . GT:PL:DP 0/1:54,0,224:194 0/1:97,0,255:150 0/1:135,0,206:151 0/1:7,0,118:20 0/1:35,0,9:18 0/1:11,0,73:89 0/1:55,0,253:193

8 8010521 . T A 999 . . GT:PL:DP 0/1:255,0,163:38 0/1:255,0,227:84 0/1:143,0,255:43 0/1:191,0,125:14 0/1:205,0,126:21 0/1:200,0,204:36 0/1:255,0,255:192

8 8023014 . A T 999 . . GT:PL:DP 0/1:255,0,189:60 0/1:255,0,46:186 0/1:255,0,28:139 0/1:164,0,43:12 0/1:78,0,150:19 0/1:255,0,192:43 0/1:255,0,112:157

8 8023261 . T C 999 . . GT:PL:DP 0/1:198,0,255:39 0/1:255,0,255:196 0/1:255,0,141:145 0/1:163,0,68:12 0/1:79,0,22:5 0/1:72,0,117:13 0/1:255,0,255:168

8 8030570 . C A 999 . . GT:PL:DP 0/1:148,0,17:12 0/1:67,0,57:12 0/1:76,3,0:5 0/1:46,6,0:2 0/1:31,3,0:1 0/1:21,0,27:3 0/1:129,0,53:18

8 8030571 . A C 999 . . GT:PL:DP 0/1:148,0,17:12 0/1:45,0,93:12 0/1:47,0,0:4 0/1:58,6,0:2 0/1:31,3,0:1 0/1:21,0,28:3 0/1:122,0,99:18

8 8113471 . A T 999 . . GT:PL:DP 0/1:108,0,78:18 0/1:179,0,164:28 0/1:186,0,24:17 0/1:49,0,25:4 0/1:27,0,21:3 0/1:24,0,87:10 0/1:182,0,255:48

8 8230589 . G C 999 . . GT:PL:DP 0/1:255,0,44:61 0/1:255,0,98:63 0/1:255,0,88:60 0/1:128,0,14:7 0/1:95,0,15:7 0/1:185,0,45:13 0/1:255,0,54:81

8 8386438 . T A 999 . . GT:PL:DP 0/1:210,0,17:23 0/1:130,0,143:30 0/1:67,0,191:31 0/1:103,0,16:7 0/1:33,0,47:6 0/1:40,0,79:10 0/1:98,0,252:42

8 8443276 . A C 999 . . GT:PL:DP 0/1:204,0,255:88 0/1:218,0,249:80 0/1:255,0,239:157 0/1:79,0,55:9 0/1:146,0,252:39 0/1:219,0,95:46 0/1:246,0,255:159

8 8443312 . C T 999 . . GT:PL:DP 0/1:202,0,255:75 0/1:190,0,255:63 0/1:212,0,252:123 0/1:61,0,119:10 0/1:52,0,222:35 0/1:49,0,66:35 0/1:160,0,226:115

8 8443412 . A T 999 . . GT:PL:DP 0/1:89,0,223:47 0/1:95,0,180:34 0/1:76,0,136:64 0/1:38,1,0:4 0/1:49,0,128:15 0/1:82,0,65:12 0/1:48,0,201:80

8 8444418 . T C 999 . . GT:PL:DP 0/1:255,0,255:223 0/1:179,0,255:195 0/1:254,0,255:206 0/1:90,0,220:44 0/1:29,0,130:17 0/1:25,0,108:17 0/1:171,0,255:264

8 8530211 . A T 999 . . GT:PL:DP 0/1:255,0,255:217 0/1:255,0,255:204 0/1:255,0,255:214 0/1:110,0,255:77 0/1:125,0,255:46 0/1:255,0,255:59 0/1:255,0,255:239

8 8530367 . C T 999 . . GT:PL:DP 0/1:255,0,255:201 0/1:255,0,255:193 0/1:255,0,255:207 0/1:106,0,188:72 0/1:98,0,255:37 0/1:207,0,255:29 0/1:255,0,255:213

8 8595821 . A C 999 . . GT:PL:DP 0/1:255,0,161:235 0/1:255,0,246:244 0/1:255,0,145:239 0/1:246,0,141:60 0/1:255,0,72:79 0/1:255,0,83:125 0/1:255,0,255:261

8 8595858 . G C 999 . . GT:PL:DP 0/1:65,0,245:160 0/1:24,0,185:148 0/1:99,0,218:137 0/1:141,0,196:42 0/1:36,0,44:33 0/1:88,0,162:68 0/1:73,0,229:162

8 8596041 . T A 999 . . GT:PL:DP 0/1:255,0,79:239 0/1:255,0,133:235 0/1:255,0,207:234 0/1:255,0,187:60 0/1:238,0,125:110 0/1:222,0,45:170 0/1:255,0,225:236

8 8600820 . A G 999 . . GT:PL:DP 0/1:255,0,255:152 0/1:255,0,255:197 0/1:124,0,255:178 0/1:49,0,255:50 0/1:248,0,255:45 0/1:184,0,236:23 0/1:34,0,255:236

8 8601757 . C T 999 . . GT:PL:DP 0/1:255,0,255:129 0/1:255,0,255:138 0/1:255,0,173:154 0/1:255,0,138:40 0/1:201,0,163:17 0/1:183,0,255:29 0/1:255,0,162:226

8 8606081 . A G 999 . . GT:PL:DP 0/1:166,0,199:34 0/1:205,0,176:41 0/1:174,0,138:25 0/1:126,0,16:7 0/1:80,0,125:19 0/1:163,0,246:58 0/1:255,0,201:88

8 8609908 . C T 999 . . GT:PL:DP 0/1:152,0,153:24 0/1:177,0,45:27 0/1:187,0,221:36 0/1:57,0,44:7 0/1:32,0,5:4 0/1:46,3,0:3 0/1:146,0,255:41

8 8651761 . G A 999 . . GT:PL:DP 0/1:241,0,23:17 0/1:118,0,55:13 0/1:126,0,50:15 0/1:71,6,0:2 0/1:97,0,11:6 0/1:37,0,0:5 0/1:210,0,50:30

8 8726838 . G A 999 . . GT:PL:DP 0/1:70,0,194:97 0/1:28,0,241:72 0/1:117,0,196:89 0/1:73,0,155:19 0/1:35,0,9:16 0/1:78,0,15:11 0/1:235,0,234:157

8 8729173 . C A 999 . . GT:PL:DP 0/1:201,0,163:60 0/1:178,0,200:45 0/1:232,0,160:100 0/1:43,0,120:7 0/1:142,0,199:31 0/1:200,0,220:50 0/1:233,0,226:86

8 8914022 . A T 999 . . GT:PL:DP 0/1:255,0,202:88 0/1:255,0,186:72 0/1:255,0,214:94 0/1:194,0,96:21 0/1:35,0,78:20 0/1:57,0,36:17 0/1:217,0,185:130

8 8927169 . A T 999 . . GT:PL:DP 0/1:125,0,128:55 0/1:135,0,135:70 0/1:123,0,100:71 0/1:55,0,77:13 0/1:18,3,0:1 0/1:34,3,0:1 0/1:96,0,171:172

8 8980038 . A T 999 . . GT:PL:DP 0/1:189,0,255:65 0/1:255,0,245:67 0/1:65,0,242:78 0/1:86,0,194:16 0/1:133,0,8:8 0/1:120,0,181:17 0/1:255,0,100:101

8 8980045 . A T 999 . . GT:PL:DP 0/1:171,0,255:62 0/1:77,0,255:65 0/1:150,0,255:78 0/1:42,0,178:12 0/1:103,0,58:8 0/1:125,0,140:15 0/1:174,0,255:98

8 8980143 . G A 999 . . GT:PL:DP 0/1:198,3,0:18 0/1:130,0,39:12 0/1:185,0,80:33 0/1:75,9,0:3 0/1:32,6,0:2 0/1:9,0,53:4 0/1:113,0,98:29

8 9046879 . C G 999 . . GT:PL:DP 0/1:255,0,214:56 0/1:106,0,255:27 0/1:103,0,255:72 0/1:118,0,78:10 0/1:12,0,0:5 0/1:55,0,25:6 0/1:255,0,255:98

8 9079184 . G A 999 . . GT:PL:DP 0/1:179,0,255:124 0/1:209,0,255:121 0/1:255,0,255:135 0/1:83,0,255:17 0/1:125,0,255:29 0/1:139,0,255:56 0/1:57,0,255:201

8 9223446 . T G 999 . . GT:PL:DP 0/1:81,2,0:10 0/1:120,0,0:15 0/1:72,0,21:11 0/1:50,6,0:5 0/1:19,3,0:1 0/1:11,0,2:4 0/1:85,0,18:11

8 9270282 . A G 999 . . GT:PL:DP 0/1:30,3,0:1 0/1:104,9,0:3 0/1:77,9,0:3 0/1:9,3,0:1 0/1:58,9,0:3 0/1:50,6,0:2 0/1:98,0,71:14

8 9287876 . T G 999 . . GT:PL:DP 0/1:82,0,152:20 0/1:89,0,36:13 0/1:53,0,99:15 0/1:48,6,0:2 0/1:34,6,0:2 0/1:16,3,0:1 0/1:74,0,12:21

8 9320933 . C A 999 . . GT:PL:DP 0/1:178,0,3:13 0/1:180,0,8:13 0/1:173,0,15:17 0/1:47,6,0:2 0/1:58,3,0:3 0/1:41,0,8:6 0/1:218,0,12:27

8 9320934 . A T 999 . . GT:PL:DP 0/1:191,0,22:14 0/1:182,0,8:13 0/1:173,0,18:17 0/1:47,6,0:2 0/1:58,3,0:3 0/1:47,0,8:6 0/1:221,0,15:27

8 9333440 . C A 999 . . GT:PL:DP 0/1:151,0,145:23 0/1:95,0,182:28 0/1:132,0,127:22 0/1:74,9,0:3 0/1:64,0,43:6 0/1:30,0,58:5 0/1:142,0,196:44

8 9379234 . C T 999 . . GT:PL:DP 0/1:255,0,255:72 0/1:255,0,255:65 0/1:255,0,255:67 0/1:103,0,126:11 0/1:47,0,124:10 0/1:189,0,255:41 0/1:217,0,255:107

8 9379377 . C T 999 . . GT:PL:DP 0/1:235,0,181:55 0/1:255,0,209:56 0/1:255,0,201:76 0/1:133,0,87:12 0/1:28,0,57:3 0/1:18,0,47:8 0/1:255,0,255:90

8 9385933 . C T 999 . . GT:PL:DP 0/1:146,0,252:179 0/1:95,0,255:170 0/1:140,0,255:177 0/1:74,0,196:44 0/1:78,0,79:10 0/1:27,0,96:12 0/1:159,0,255:192

8 9385983 . T A 999 . . GT:PL:DP 0/1:126,0,255:220 0/1:81,0,255:215 0/1:84,0,255:211 0/1:58,0,255:64 0/1:85,0,184:32 0/1:22,0,172:32 0/1:89,0,255:224

8 9386440 . G C 999 . . GT:PL:DP 0/1:255,0,255:179 0/1:235,0,245:177 0/1:252,0,253:185 0/1:223,0,172:67 0/1:142,0,153:50 0/1:114,0,219:67 0/1:255,0,244:162

8 9386600 . A G 999 . . GT:PL:DP 0/1:150,0,115:128 0/1:138,0,119:126 0/1:131,0,106:126 0/1:105,0,62:21 0/1:90,0,9:8 0/1:88,0,8:9 0/1:143,0,14:133

8 9386645 . A C 999 . . GT:PL:DP 0/1:117,28,112:126 0/1:104,0,164:136 0/1:98,0,115:137 0/1:81,0,52:14 0/1:76,0,40:8 0/1:70,0,91:9 0/1:108,0,89:114

8 9386697 . A G 999 . . GT:PL:DP 0/1:100,0,140:93 0/1:95,0,176:94 0/1:103,0,134:111 0/1:54,0,54:8 0/1:31,0,6:3 0/1:34,0,43:5 0/1:116,0,122:63

8 9408163 . T C 999 . . GT:PL:DP 0/1:84,0,255:144 0/1:203,0,248:78 0/1:227,0,255:148 0/1:113,0,33:17 0/1:32,1,0:2 0/1:13,0,33:8 0/1:165,0,204:168

8 9544332 . C T 999 . . GT:PL:DP 0/1:81,0,181:21 0/1:71,0,139:23 0/1:71,0,215:32 0/1:15,0,71:5 0/1:85,0,64:12 0/1:43,0,23:4 0/1:94,0,223:43

8 9544351 . C A 999 . . GT:PL:DP 0/1:149,0,133:22 0/1:218,0,58:30 0/1:202,0,124:32 0/1:83,0,46:6 0/1:41,0,101:11 0/1:68,0,38:6 0/1:211,0,154:41

8 9544375 . G C 999 . . GT:PL:DP 0/1:132,0,160:24 0/1:37,0,217:24 0/1:97,0,189:26 0/1:49,0,67:5 0/1:61,0,64:9 0/1:14,0,81:6 0/1:123,0,225:39

8 9544584 . T G 999 . . GT:PL:DP 0/1:255,0,228:37 0/1:255,0,141:34 0/1:203,0,158:24 0/1:89,0,31:5 0/1:130,0,93:9 0/1:94,0,50:7 0/1:255,0,255:44

8 9587154 . T G 999 . . GT:PL:DP 0/1:176,0,125:66 0/1:180,0,30:65 0/1:145,0,117:88 0/1:105,0,50:11 0/1:97,0,44:9 0/1:18,0,68:8 0/1:193,0,153:136

8 9629708 . A C 999 . . GT:PL:DP 0/1:255,0,110:271 0/1:255,0,123:258 0/1:253,0,117:246 0/1:255,0,227:85 0/1:127,0,152:126 0/1:208,0,248:151 0/1:233,0,131:243

8 9629751 . G A 999 . . GT:PL:DP 0/1:255,0,250:239 0/1:243,0,217:232 0/1:255,0,182:222 0/1:255,0,224:97 0/1:149,0,197:97 0/1:255,0,255:143 0/1:249,0,245:231

8 9629770 . T C 999 . . GT:PL:DP 0/1:255,0,255:227 0/1:255,0,255:229 0/1:255,0,216:216 0/1:255,0,255:111 0/1:210,0,200:121 0/1:252,0,255:163 0/1:254,0,249:235

8 9629795 . C T 999 . . GT:PL:DP 0/1:255,0,255:236 0/1:242,0,255:240 0/1:255,0,255:224 0/1:255,0,247:124 0/1:216,0,255:127 0/1:255,0,255:181 0/1:236,0,252:232

8 9630025 . T C 999 . . GT:PL:DP 0/1:186,0,255:251 0/1:212,0,255:245 0/1:125,0,255:222 0/1:239,0,255:166 0/1:156,0,255:147 0/1:124,0,255:148 0/1:50,0,255:239

8 9653659 . G A 999 . . GT:PL:DP 0/1:69,0,107:26 0/1:109,0,102:31 0/1:88,0,157:39 0/1:105,0,39:14 0/1:35,0,84:15 0/1:80,0,129:13 0/1:109,0,245:69

8 9693182 . C T 999 . . GT:PL:DP 0/1:130,0,127:95 0/1:156,0,128:94 0/1:144,0,174:94 0/1:76,0,36:13 0/1:114,0,12:8 0/1:36,0,58:15 0/1:162,0,65:30

8 9800259 . C A 999 . . GT:PL:DP 0/1:170,0,255:243 0/1:184,0,225:238 0/1:107,0,255:245 0/1:131,0,255:117 0/1:41,0,246:54 0/1:160,0,255:52 0/1:134,0,255:232

8 9807616 . G A 999 . . GT:PL:DP 0/1:140,0,94:18 0/1:189,0,76:29 0/1:174,0,165:46 0/1:13,0,62:6 0/1:12,0,63:7 0/1:12,0,82:6 0/1:159,0,190:65

8 9934391 . A G 999 . . GT:PL:DP 0/1:149,0,152:83 0/1:151,0,37:34 0/1:137,0,249:68 0/1:126,0,80:18 0/1:52,1,0:15 0/1:13,0,0:7 0/1:141,0,255:64

8 9934407 . G A 999 . . GT:PL:DP 0/1:141,0,143:88 0/1:136,0,37:38 0/1:131,0,250:72 0/1:112,0,71:22 0/1:30,1,0:14 0/1:13,0,0:7 0/1:163,0,255:69

8 9934411 . C G 999 . . GT:PL:DP 0/1:136,0,135:88 0/1:132,0,29:38 0/1:122,0,247:73 0/1:110,0,73:22 0/1:31,2,0:13 0/1:16,1,0:7 0/1:165,0,255:71

8 9934433 . G A 999 . . GT:PL:DP 0/1:141,0,150:97 0/1:130,0,29:40 0/1:135,0,185:75 0/1:106,0,44:23 0/1:13,4,0:9 0/1:11,0,21:8 0/1:161,0,243:71

8 9934564 . A T 999 . . GT:PL:DP 0/1:252,0,255:164 0/1:249,0,186:76 0/1:248,0,255:100 0/1:233,0,130:37 0/1:85,0,162:19 0/1:85,0,20:5 0/1:255,0,245:162

8 9934655 . C T 999 . . GT:PL:DP 0/1:255,0,255:216 0/1:222,0,201:92 0/1:224,0,255:178 0/1:223,0,73:50 0/1:163,0,64:19 0/1:94,0,148:16 0/1:234,0,236:204

8 9935500 . G T 999 . . GT:PL:DP 0/1:108,0,183:95 0/1:105,0,227:60 0/1:97,0,239:60 0/1:89,0,12:14 0/1:23,0,141:17 0/1:44,0,194:46 0/1:111,0,225:118

8 9935657 . A T 999 . . GT:PL:DP 0/1:103,0,220:64 0/1:121,0,157:38 0/1:66,0,185:50 0/1:131,50,81:15 0/1:20,0,48:8 0/1:79,0,68:14 0/1:58,0,214:67

8 9935728 . C T 999 . . GT:PL:DP 0/1:128,0,187:73 0/1:131,0,134:44 0/1:111,0,169:56 0/1:138,0,22:15 0/1:110,0,116:16 0/1:160,0,161:22 0/1:130,0,240:60

8 9951338 . T A 999 . . GT:PL:DP 0/1:71,0,155:235 0/1:108,0,242:229 0/1:69,0,162:241 0/1:200,0,255:217 0/1:123,0,255:205 0/1:85,0,255:151 0/1:7,0,148:221

8 9951429 . C A 999 . . GT:PL:DP 0/1:255,0,202:237 0/1:223,0,186:227 0/1:231,0,205:238 0/1:255,0,255:224 0/1:128,0,3:144 0/1:190,0,105:138 0/1:234,0,163:220

8 9951612 . G A 999 . . GT:PL:DP 0/1:178,0,255:220 0/1:255,0,255:215 0/1:185,0,255:224 0/1:235,0,255:215 0/1:61,0,255:228 0/1:23,0,255:201 0/1:127,0,255:207

8 9951676 . A T 999 . . GT:PL:DP 0/1:176,0,255:215 0/1:255,0,255:189 0/1:107,0,255:209 0/1:111,0,255:200 0/1:139,0,255:146 0/1:115,0,255:124 0/1:104,0,255:186

8 9951684 . C A 999 . . GT:PL:DP 0/1:168,0,255:211 0/1:255,0,255:186 0/1:106,0,255:203 0/1:93,0,255:194 0/1:148,0,255:131 0/1:160,0,255:107 0/1:96,0,255:185

8 9969545 . C T 999 . . GT:PL:DP 0/1:255,0,255:224 0/1:252,0,255:226 0/1:255,0,255:229 0/1:244,0,255:93 0/1:169,0,235:69 0/1:101,0,255:65 0/1:242,0,255:239

8 9990948 . T A 999 . . GT:PL:DP 0/1:46,0,44:124 0/1:137,0,128:96 0/1:83,0,106:140 0/1:90,0,19:12 0/1:24,4,0:10 0/1:50,3,0:34 0/1:126,0,24:174

8 9991020 . A T 999 . . GT:PL:DP 0/1:244,0,77:164 0/1:255,0,131:111 0/1:255,0,184:201 0/1:109,0,21:18 0/1:108,7,0:15 0/1:102,6,0:38 0/1:255,0,132:181

8 10231998 . T A 999 . . GT:PL:DP 0/1:13,0,245:56 0/1:103,0,237:43 0/1:85,0,243:42 0/1:22,0,39:3 0/1:41,0,89:7 0/1:82,0,186:21 0/1:21,0,255:96

8 10332697 . G A 999 . . GT:PL:DP 0/1:245,0,255:104 0/1:193,0,255:118 0/1:255,0,255:112 0/1:172,0,233:26 0/1:37,0,96:24 0/1:43,0,155:33 0/1:255,0,255:198

8 10415469 . A T 999 . . GT:PL:DP 0/1:24,0,78:66 0/1:61,0,124:90 0/1:104,0,143:119 0/1:44,0,23:20 0/1:73,0,80:107 0/1:9,0,144:159 0/1:37,0,162:188

8 10425670 . C T 999 . . GT:PL:DP 0/1:29,0,255:24 0/1:131,0,210:31 0/1:170,0,96:15 0/1:32,0,31:2 0/1:47,0,74:8 0/1:196,0,115:16 0/1:129,0,255:35

8 10591933 . A C 999 . . GT:PL:DP 0/1:25,6,0:2 0/1:98,0,33:10 0/1:34,3,0:1 0/1:97,9,0:3 0/1:51,6,0:2 0/1:63,0,19:4 0/1:65,0,41:6

8 10735670 . A C 999 . . GT:PL:DP 0/1:151,0,238:46 0/1:114,0,217:30 0/1:212,0,67:38 0/1:30,0,20:3 0/1:50,0,38:5 0/1:40,0,43:4 0/1:205,0,188:57

8 10801704 . G T 999 . . GT:PL:DP 0/1:137,0,132:45 0/1:92,0,170:25 0/1:158,0,178:26 0/1:22,0,82:5 0/1:77,0,68:9 0/1:62,0,75:16 0/1:192,0,207:39

8 10801788 . A G 999 . . GT:PL:DP 0/1:84,0,133:55 0/1:21,0,128:34 0/1:100,0,227:31 0/1:25,0,52:4 0/1:64,3,0:3 0/1:47,2,0:7 0/1:87,0,169:28

8 10811518 . A T 999 . . GT:PL:DP 0/1:29,0,181:19 0/1:197,0,163:38 0/1:27,0,184:29 0/1:23,0,20:4 0/1:27,0,50:4 0/1:63,0,18:6 0/1:129,0,231:51

8 10925861 . T G 999 . . GT:PL:DP 0/1:74,0,69:24 0/1:38,0,106:21 0/1:34,0,94:13 0/1:71,9,0:3 0/1:17,3,0:1 0/1:19,0,25:4 0/1:132,0,46:27

8 10979894 . A T 999 . . GT:PL:DP 0/1:255,0,255:36 0/1:196,0,255:33 0/1:255,0,255:41 0/1:42,0,64:5 0/1:106,0,114:8 0/1:73,0,122:8 0/1:255,0,255:46

8 10981591 . G A 999 . . GT:PL:DP 0/1:255,0,255:37 0/1:255,0,250:41 0/1:255,0,247:56 0/1:84,0,103:9 0/1:24,0,35:2 0/1:44,0,52:5 0/1:240,0,193:31

8 11004015 . A G 999 . . GT:PL:DP 0/1:41,0,14:11 0/1:74,4,0:7 0/1:85,0,21:11 0/1:29,6,0:2 0/1:38,6,0:2 0/1:19,3,0:1 0/1:131,0,4:13

8 11116589 . A T 999 . . GT:PL:DP 0/1:218,0,255:58 0/1:147,0,129:22 0/1:156,0,211:35 0/1:97,0,118:13 0/1:18,0,120:10 0/1:36,0,149:22 0/1:152,0,255:126

8 11205541 . T C 999 . . GT:PL:DP 0/1:255,0,255:239 0/1:255,0,255:220 0/1:84,0,237:187 0/1:222,0,255:41 0/1:226,0,203:43 0/1:255,0,255:73 0/1:208,0,255:227

8 11317388 . T C 999 . . GT:PL:DP 0/1:195,0,255:33 0/1:195,0,219:26 0/1:200,0,110:19 0/1:77,9,0:3 0/1:82,9,0:3 0/1:178,3,0:8 0/1:166,0,255:41

8 11328703 . G C 999 . . GT:PL:DP 0/1:149,0,189:29 0/1:252,0,180:53 0/1:207,0,186:26 0/1:95,0,55:7 0/1:73,0,5:5 0/1:133,2,0:7 0/1:236,0,238:78

8 11343979 . G A 999 . . GT:PL:DP 0/1:255,0,185:32 0/1:255,0,236:46 0/1:255,0,226:36 0/1:25,0,89:4 0/1:31,0,30:2 0/1:45,0,59:5 0/1:255,0,252:50

8 11344153 . T A 999 . . GT:PL:DP 0/1:199,0,239:35 0/1:255,0,135:37 0/1:241,0,185:35 0/1:31,0,113:8 0/1:35,0,21:3 0/1:30,0,46:4 0/1:249,0,201:48

8 11344385 . T A 999 . . GT:PL:DP 0/1:129,0,72:35 0/1:131,0,15:30 0/1:101,0,182:27 0/1:17,0,40:5 0/1:34,0,11:5 0/1:7,0,61:4 0/1:81,0,127:38

8 11344404 . T C 999 . . GT:PL:DP 0/1:166,0,77:32 0/1:160,3,0:24 0/1:104,0,197:29 0/1:6,0,37:4 0/1:36,0,11:5 0/1:10,0,42:3 0/1:102,0,94:37

8 11344932 . A C 999 . . GT:PL:DP 0/1:177,0,255:41 0/1:89,0,255:35 0/1:98,0,108:11 0/1:29,0,31:2 0/1:49,0,26:3 0/1:19,0,25:2 0/1:99,0,255:30

8 11344942 . A T 999 . . GT:PL:DP 0/1:150,0,255:44 0/1:74,0,255:30 0/1:78,0,83:9 0/1:29,0,31:2 0/1:23,0,29:2 0/1:19,0,15:2 0/1:88,0,255:30

8 11345147 . T C 999 . . GT:PL:DP 0/1:81,0,255:35 0/1:33,0,201:16 0/1:48,0,130:17 0/1:30,0,95:5 0/1:17,0,83:6 0/1:15,0,120:9 0/1:54,0,255:22

8 11345159 . T C 999 . . GT:PL:DP 0/1:81,0,255:35 0/1:52,0,173:16 0/1:56,0,137:18 0/1:30,0,95:5 0/1:8,0,95:5 0/1:12,0,161:10 0/1:74,0,255:23

8 11345166 . T C 156.362 . . GT:PL:DP 0/1:62,0,255:36 0/1:40,0,188:17 0/1:51,0,113:18 0/1:30,0,90:5 0/1:7,0,57:4 0/1:12,0,122:10 0/1:42,0,255:24

8 11345195 . C T 999 . . GT:PL:DP 0/1:102,0,255:33 0/1:124,0,238:22 0/1:178,0,106:20 0/1:62,0,62:4 0/1:47,0,26:4 0/1:31,0,109:7 0/1:126,0,255:23

8 11345230 . A T 999 . . GT:PL:DP 0/1:190,0,255:39 0/1:176,0,164:20 0/1:165,0,185:21 0/1:64,0,28:3 0/1:62,0,51:5 0/1:73,0,158:10 0/1:173,0,255:26

8 11355550 . A T 999 . . GT:PL:DP 0/1:116,0,255:52 0/1:173,0,255:58 0/1:83,0,255:82 0/1:14,0,79:6 0/1:87,0,98:26 0/1:31,0,124:43 0/1:124,0,255:102

8 11355805 . C T 999 . . GT:PL:DP 0/1:94,0,143:27 0/1:134,0,110:23 0/1:119,0,202:27 0/1:55,6,0:2 0/1:103,0,5:7 0/1:115,0,9:8 0/1:86,0,170:21

8 11374830 . A G 999 . . GT:PL:DP 0/1:204,0,133:23 0/1:172,0,47:16 0/1:112,0,176:21 0/1:23,0,50:3 0/1:34,3,0:1 0/1:44,0,150:9 0/1:151,0,153:19

8 11390238 . A T 999 . . GT:PL:DP 0/1:56,0,45:15 0/1:37,0,31:33 0/1:104,0,29:44 0/1:16,0,10:4 0/1:42,1,0:3 0/1:17,0,6:3 0/1:154,0,78:34

8 11452531 . C T 999 . . GT:PL:DP 0/1:62,0,225:29 0/1:9,0,179:12 0/1:120,0,28:14 0/1:25,3,0:1 0/1:45,0,46:4 0/1:55,6,0:2 0/1:141,0,255:38

8 11462944 . C A 999 . . GT:PL:DP 0/1:113,0,4:22 0/1:116,0,3:12 0/1:139,0,3:22 0/1:38,6,0:2 0/1:25,0,5:3 0/1:12,0,8:2 0/1:69,0,255:34

8 11462949 . C A 999 . . GT:PL:DP 0/1:135,2,0:24 0/1:112,0,12:15 0/1:138,0,36:23 0/1:88,12,0:4 0/1:25,0,5:3 0/1:9,0,13:3 0/1:123,0,255:35

8 11462950 . T C 999 . . GT:PL:DP 0/1:140,2,0:24 0/1:112,0,11:15 0/1:139,0,36:23 0/1:88,12,0:4 0/1:25,0,5:3 0/1:9,0,13:3 0/1:71,0,255:36

8 11464361 . A G 999 . . GT:PL:DP 0/1:255,0,255:107 0/1:132,0,255:44 0/1:218,0,255:37 0/1:255,0,124:21 0/1:255,0,245:27 0/1:76,0,229:13 0/1:255,0,255:128

8 11464529 . C A 999 . . GT:PL:DP 0/1:255,0,204:64 0/1:155,0,157:28 0/1:255,0,125:22 0/1:156,0,67:18 0/1:244,0,220:32 0/1:55,0,161:11 0/1:255,0,255:93

8 11500608 . T A 999 . . GT:PL:DP 0/1:255,0,255:126 0/1:255,0,199:115 0/1:255,0,102:144 0/1:251,0,83:22 0/1:98,0,44:9 0/1:230,0,109:32 0/1:255,0,205:172

8 11501102 . C T 999 . . GT:PL:DP 0/1:177,0,214:64 0/1:202,0,149:60 0/1:112,0,234:76 0/1:21,0,94:7 0/1:18,0,58:6 0/1:115,0,91:22 0/1:255,0,255:133

9 235836 . G A 999 . . GT:PL:DP 0/1:206,0,56:22 0/1:146,0,169:21 0/1:128,0,173:22 0/1:25,3,0:1 0/1:53,6,0:2 0/1:119,6,0:8 0/1:191,0,120:25

9 434716 . C A 999 . . GT:PL:DP 0/1:182,0,55:22 0/1:194,0,75:23 0/1:90,0,192:22 0/1:53,6,0:2 0/1:70,0,13:4 0/1:66,0,38:6 0/1:217,0,81:39

9 492079 . A G 999 . . GT:PL:DP 0/1:165,0,255:92 0/1:199,0,255:82 0/1:231,0,240:46 0/1:138,0,15:7 0/1:118,0,219:20 0/1:18,0,255:19 0/1:211,0,255:43

9 492089 . G A 999 . . GT:PL:DP 0/1:164,0,255:93 0/1:250,0,255:81 0/1:226,0,209:44 0/1:146,0,12:8 0/1:107,0,226:19 0/1:21,0,255:20 0/1:202,0,255:44

9 492274 . C G 999 . . GT:PL:DP 0/1:175,0,255:81 0/1:167,0,255:76 0/1:255,0,184:35 0/1:50,0,113:6 0/1:178,0,113:13 0/1:145,0,255:35 0/1:211,0,252:31

9 638860 . T C 999 . . GT:PL:DP 0/1:216,0,134:61 0/1:141,0,203:76 0/1:104,0,172:49 0/1:12,0,112:19 0/1:166,0,212:20 0/1:186,0,255:34 0/1:126,0,165:113

9 638935 . A T 999 . . GT:PL:DP 0/1:255,0,180:73 0/1:164,0,228:76 0/1:118,0,255:50 0/1:18,0,121:17 0/1:41,0,129:9 0/1:52,0,75:5 0/1:245,0,234:115

9 639255 . A T 999 . . GT:PL:DP 0/1:30,0,201:141 0/1:151,0,255:141 0/1:116,0,255:162 0/1:132,0,168:17 0/1:46,0,45:5 0/1:74,0,130:14 0/1:112,0,239:195

9 639597 . A T 999 . . GT:PL:DP 0/1:255,0,255:176 0/1:254,0,255:170 0/1:255,0,255:211 0/1:21,0,255:34 0/1:130,0,255:30 0/1:125,0,134:19 0/1:255,0,255:194

9 639840 . G A 999 . . GT:PL:DP 0/1:255,0,255:214 0/1:219,0,255:208 0/1:216,0,255:225 0/1:17,0,255:42 0/1:150,0,255:36 0/1:121,0,255:40 0/1:255,0,255:197

9 640240 . G A 999 . . GT:PL:DP 0/1:255,0,255:145 0/1:255,0,255:119 0/1:255,0,255:166 0/1:86,0,255:22 0/1:151,0,255:31 0/1:165,0,153:22 0/1:255,0,255:189

9 640254 . A T 999 . . GT:PL:DP 0/1:255,0,255:138 0/1:255,0,255:118 0/1:255,0,255:164 0/1:127,0,255:24 0/1:177,0,253:27 0/1:179,0,173:22 0/1:255,0,255:190

9 640586 . T A 999 . . GT:PL:DP 0/1:255,0,246:95 0/1:255,0,255:99 0/1:255,0,255:102 0/1:20,0,126:9 0/1:246,0,173:29 0/1:171,0,149:15 0/1:255,0,255:177

9 732356 . G A 999 . . GT:PL:DP 0/1:35,3,0:1 0/1:55,25,22:2 0/1:60,29,26:2 0/1:36,3,0:1 0/1:22,0,9:3 0/1:38,6,0:2 0/1:32,3,0:1

9 732357 . A G 999 . . GT:PL:DP 0/1:25,3,0:1 0/1:30,0,22:2 0/1:31,0,26:2 0/1:36,3,0:1 0/1:36,12,9:3 0/1:53,3,0:4 0/1:22,3,0:1

9 795424 . A G 999 . . GT:PL:DP 0/1:76,5,0:12 0/1:66,0,60:9 0/1:37,0,102:9 0/1:20,0,17:2 0/1:43,0,10:5 0/1:91,0,61:9 0/1:16,0,140:14

9 879971 . G A 999 . . GT:PL:DP 0/1:255,0,255:179 0/1:255,0,255:166 0/1:255,0,255:163 0/1:255,0,255:108 0/1:197,0,231:56 0/1:244,0,255:67 0/1:255,0,255:153

9 880227 . A T 999 . . GT:PL:DP 0/1:255,0,255:187 0/1:225,0,255:183 0/1:224,0,255:181 0/1:224,0,255:113 0/1:97,0,108:30 0/1:161,0,72:49 0/1:161,0,255:161

9 880228 . A T 999 . . GT:PL:DP 0/1:255,0,255:188 0/1:208,0,255:184 0/1:227,0,255:186 0/1:233,0,255:113 0/1:96,0,107:32 0/1:192,0,76:51 0/1:162,0,255:162

9 880286 . T C 999 . . GT:PL:DP 0/1:255,0,255:221 0/1:255,0,255:224 0/1:255,0,255:207 0/1:210,0,255:214 0/1:254,0,180:88 0/1:255,0,229:144 0/1:238,0,255:189

9 880304 . T A 999 . . GT:PL:DP 0/1:255,0,255:215 0/1:253,0,255:209 0/1:249,0,255:200 0/1:197,0,255:212 0/1:206,0,189:79 0/1:218,0,219:130 0/1:136,0,255:181

9 880440 . A T 999 . . GT:PL:DP 0/1:185,0,255:203 0/1:195,0,255:200 0/1:181,0,255:204 0/1:143,0,255:142 0/1:183,0,247:78 0/1:78,0,223:35 0/1:159,0,255:180

9 880789 . A T 999 . . GT:PL:DP 0/1:147,0,237:154 0/1:186,0,255:166 0/1:191,0,255:153 0/1:135,0,239:71 0/1:81,0,107:33 0/1:48,0,228:48 0/1:161,0,255:134

9 881140 . T A 999 . . GT:PL:DP 0/1:233,0,255:191 0/1:137,0,255:188 0/1:239,0,255:198 0/1:172,0,255:97 0/1:87,0,134:26 0/1:41,0,142:36 0/1:255,0,255:182

9 920531 . A G 999 . . GT:PL:DP 0/1:255,0,255:50 0/1:220,0,219:28 0/1:255,0,255:48 0/1:156,0,19:11 0/1:55,0,66:12 0/1:13,0,147:21 0/1:255,0,168:69

9 984501 . A G 999 . . GT:PL:DP 0/1:255,0,255:35 0/1:132,0,255:38 0/1:255,0,201:35 0/1:76,0,85:7 0/1:54,9,0:3 0/1:69,9,0:3 0/1:255,0,255:54

9 1036302 . A G 999 . . GT:PL:DP 0/1:255,0,154:55 0/1:255,0,122:40 0/1:255,0,144:54 0/1:141,0,90:11 0/1:20,0,57:3 0/1:97,0,96:9 0/1:255,0,175:87

9 1036306 . C T 999 . . GT:PL:DP 0/1:255,0,154:55 0/1:246,0,126:38 0/1:253,0,135:51 0/1:116,0,89:11 0/1:17,0,44:4 0/1:70,0,98:9 0/1:255,0,187:87

9 1036953 . T C 999 . . GT:PL:DP 0/1:220,0,255:74 0/1:162,0,255:64 0/1:63,0,255:46 0/1:52,0,134:10 0/1:169,0,53:12 0/1:109,0,98:12 0/1:251,0,255:162

9 1036962 . A G 999 . . GT:PL:DP 0/1:226,0,255:72 0/1:203,0,255:63 0/1:99,0,255:49 0/1:78,0,141:10 0/1:147,0,67:10 0/1:107,0,123:14 0/1:255,0,255:162

9 1039437 . G A 999 . . GT:PL:DP 0/1:188,0,255:72 0/1:174,0,255:45 0/1:155,0,255:54 0/1:62,0,81:7 0/1:99,0,145:11 0/1:70,0,64:7 0/1:158,0,255:77

9 1121577 . A G 999 . . GT:PL:DP 0/1:223,0,154:23 0/1:199,0,81:16 0/1:187,0,109:22 0/1:53,0,59:5 0/1:32,0,169:10 0/1:105,0,126:9 0/1:81,0,249:20

9 1121680 . G A 999 . . GT:PL:DP 0/1:144,0,141:16 0/1:126,0,83:10 0/1:73,0,97:10 0/1:50,0,20:4 0/1:46,0,63:5 0/1:82,0,63:8 0/1:96,0,140:17

9 1229944 . A G 999 . . GT:PL:DP 0/1:75,0,64:38 0/1:26,0,52:20 0/1:109,0,42:30 0/1:22,0,19:5 0/1:51,5,0:10 0/1:23,0,5:21 0/1:58,0,39:60

9 1387353 . A C 999 . . GT:PL:DP 0/1:162,0,39:17 0/1:136,0,20:13 0/1:129,0,17:19 0/1:36,6,0:2 0/1:17,0,33:3 0/1:59,9,0:3 0/1:153,0,64:15

9 1387355 . T G 999 . . GT:PL:DP 0/1:162,0,39:17 0/1:146,0,26:11 0/1:137,0,14:19 0/1:36,6,0:2 0/1:17,0,33:3 0/1:59,9,0:3 0/1:153,0,57:15

9 2036610 . T G 999 . . GT:PL:DP 0/1:169,0,248:35 0/1:184,0,211:31 0/1:180,0,152:19 0/1:101,0,44:7 0/1:33,3,0:1 0/1:51,0,58:8 0/1:217,0,255:59

9 2178355 . T G 999 . . GT:PL:DP 0/1:109,0,119:17 0/1:143,0,86:27 0/1:57,0,167:20 0/1:47,0,75:6 0/1:41,9,0:3 0/1:17,3,0:1 0/1:104,0,225:30

9 2178402 . A T 999 . . GT:PL:DP 0/1:51,0,84:13 0/1:105,0,55:24 0/1:36,0,138:18 0/1:43,0,47:5 0/1:48,0,17:5 0/1:11,0,22:2 0/1:81,0,230:30

9 2370547 . C T 999 . . GT:PL:DP 0/1:226,0,50:31 0/1:234,0,40:28 0/1:243,0,92:41 0/1:155,0,35:11 0/1:27,0,23:5 0/1:49,0,73:5 0/1:255,0,132:51

9 2394524 . C G 999 . . GT:PL:DP 0/1:148,0,106:24 0/1:151,0,139:27 0/1:125,0,88:20 0/1:22,3,0:1 0/1:80,9,0:3 0/1:23,0,23:2 0/1:101,0,215:30

9 2426323 . A T 999 . . GT:PL:DP 0/1:14,0,99:12 0/1:15,0,72:7 0/1:58,0,41:10 0/1:18,3,0:1 0/1:32,6,0:2 0/1:32,6,0:2 0/1:32,0,127:16

9 2492966 . A G 999 . . GT:PL:DP 0/1:172,0,176:38 0/1:185,0,246:30 0/1:147,0,172:35 0/1:11,0,124:6 0/1:34,0,81:7 0/1:21,0,79:6 0/1:219,0,240:71

9 2492972 . A G 999 . . GT:PL:DP 0/1:51,0,195:43 0/1:114,0,255:28 0/1:181,0,145:36 0/1:87,0,63:6 0/1:18,0,124:7 0/1:21,0,90:6 0/1:47,0,239:71

9 2609911 . A T 97.7243 . . GT:PL:DP 0/1:16,3,0:1 0/1:21,3,0:1 0/1:70,12,0:4 0/1:21,3,0:1 0/1:20,3,0:1 0/1:16,3,0:1 0/1:59,9,0:3

9 2609912 . T C 93.5028 . . GT:PL:DP 0/1:16,3,0:1 0/1:21,3,0:1 0/1:70,12,0:4 0/1:11,3,0:1 0/1:20,3,0:1 0/1:16,3,0:1 0/1:59,9,0:3

9 2644350 . C T 999 . . GT:PL:DP 0/1:154,0,211:29 0/1:177,0,211:24 0/1:158,0,230:36 0/1:20,0,50:4 0/1:122,0,24:8 0/1:31,13,7:3 0/1:213,0,255:56

9 2654745 . G A 999 . . GT:PL:DP 0/1:216,0,223:57 0/1:255,0,209:57 0/1:255,0,125:36 0/1:126,0,72:9 0/1:96,0,35:15 0/1:139,0,75:23 0/1:255,0,218:102

9 2654824 . G A 999 . . GT:PL:DP 0/1:255,0,255:81 0/1:255,0,205:65 0/1:255,0,247:55 0/1:71,0,75:8 0/1:106,0,74:10 0/1:137,0,141:21 0/1:255,0,255:108

9 2675103 . G T 999 . . GT:PL:DP 0/1:255,0,15:43 0/1:255,0,69:44 0/1:255,0,100:37 0/1:64,0,59:5 0/1:18,0,139:7 0/1:126,0,84:10 0/1:255,0,22:45

9 2675139 . C A 999 . . GT:PL:DP 0/1:255,0,31:44 0/1:255,0,96:36 0/1:255,0,94:35 0/1:94,0,56:6 0/1:29,0,36:4 0/1:149,0,131:12 0/1:255,0,23:51

9 2681811 . A G 999 . . GT:PL:DP 0/1:249,0,122:29 0/1:254,0,74:39 0/1:255,0,151:33 0/1:63,6,0:2 0/1:86,0,81:8 0/1:205,0,55:10 0/1:255,0,166:66

9 2692906 . C T 999 . . GT:PL:DP 0/1:255,0,255:241 0/1:255,0,204:244 0/1:255,0,178:246 0/1:255,0,208:122 0/1:255,0,251:93 0/1:255,0,225:125 0/1:255,0,255:235

9 2692919 . G A 999 . . GT:PL:DP 0/1:255,0,255:241 0/1:255,0,221:241 0/1:255,0,144:244 0/1:255,0,239:112 0/1:255,0,255:95 0/1:255,0,229:116 0/1:255,0,255:235

9 2693115 . G T 999 . . GT:PL:DP 0/1:240,0,255:245 0/1:255,0,255:238 0/1:255,0,255:242 0/1:255,0,255:129 0/1:255,0,255:98 0/1:223,0,255:112 0/1:188,0,255:238

9 2693245 . C T 999 . . GT:PL:DP 0/1:255,0,255:244 0/1:255,0,255:240 0/1:255,0,255:247 0/1:255,0,255:113 0/1:255,0,255:117 0/1:228,0,255:134 0/1:223,0,255:233

9 2693389 . G A 999 . . GT:PL:DP 0/1:255,0,255:245 0/1:255,0,255:243 0/1:255,0,255:246 0/1:255,0,255:108 0/1:255,0,255:104 0/1:206,0,255:122 0/1:254,0,255:242

9 2693824 . G A 999 . . GT:PL:DP 0/1:160,0,255:244 0/1:217,0,255:237 0/1:230,0,255:235 0/1:223,0,214:94 0/1:226,0,255:88 0/1:166,0,255:90 0/1:183,0,255:236

9 2693998 . C T 999 . . GT:PL:DP 0/1:143,0,255:223 0/1:44,0,191:231 0/1:11,0,183:233 0/1:159,0,255:65 0/1:96,0,255:76 0/1:255,0,255:118 0/1:241,0,255:222

9 2694016 . T C 999 . . GT:PL:DP 0/1:238,0,255:212 0/1:255,0,255:231 0/1:255,0,255:218 0/1:216,0,255:59 0/1:255,0,255:72 0/1:236,0,255:112 0/1:206,0,255:199

9 2694070 . G A 999 . . GT:PL:DP 0/1:206,0,255:208 0/1:255,0,255:204 0/1:255,0,255:222 0/1:134,0,242:61 0/1:233,0,255:65 0/1:172,0,255:101 0/1:228,0,255:196

9 2694079 . G T 999 . . GT:PL:DP 0/1:187,0,255:193 0/1:206,0,255:211 0/1:227,0,255:221 0/1:110,0,244:56 0/1:199,0,255:55 0/1:237,0,255:92 0/1:187,0,255:189

9 2708853 . T A 999 . . GT:PL:DP 0/1:77,0,153:22 0/1:194,0,88:34 0/1:151,0,183:20 0/1:104,0,40:6 0/1:58,0,19:6 0/1:39,0,80:5 0/1:54,0,181:27

9 2709366 . G T 999 . . GT:PL:DP 0/1:170,0,100:23 0/1:255,0,200:71 0/1:255,0,140:57 0/1:216,0,13:19 0/1:23,2,0:8 0/1:37,2,0:7 0/1:253,0,40:92

9 2768566 . G A 999 . . GT:PL:DP 0/1:243,0,166:160 0/1:203,0,178:107 0/1:255,0,146:167 0/1:187,0,77:27 0/1:169,0,145:45 0/1:186,0,161:42 0/1:255,0,175:253

9 2862821 . T A 999 . . GT:PL:DP 0/1:255,0,255:100 0/1:255,0,255:103 0/1:255,0,240:125 0/1:112,0,161:24 0/1:18,0,121:8 0/1:255,0,203:50 0/1:255,0,255:200

9 2865339 . T C 999 . . GT:PL:DP 0/1:145,0,132:27 0/1:89,0,153:22 0/1:154,0,188:32 0/1:52,0,60:8 0/1:66,9,0:3 0/1:66,9,0:3 0/1:150,0,215:63

9 2996842 . G A 999 . . GT:PL:DP 0/1:154,0,255:98 0/1:188,0,255:93 0/1:183,0,255:127 0/1:51,0,129:14 0/1:117,0,125:16 0/1:104,0,207:27 0/1:210,0,255:152

9 3078335 . G A 999 . . GT:PL:DP 0/1:35,0,228:26 0/1:142,0,112:18 0/1:147,0,78:19 0/1:46,0,6:6 0/1:58,0,41:5 0/1:54,6,0:2 0/1:72,0,164:27

9 3078351 . G C 999 . . GT:PL:DP 0/1:33,0,235:23 0/1:77,0,114:19 0/1:101,0,87:24 0/1:22,6,0:6 0/1:75,5,0:4 0/1:55,9,0:3 0/1:45,0,148:26

9 3078352 . G T 999 . . GT:PL:DP 0/1:27,0,222:21 0/1:84,0,133:12 0/1:138,0,113:14 0/1:20,0,18:2 0/1:49,3,0:3 0/1:54,6,0:2 0/1:37,0,144:19

9 3128302 . T A 999 . . GT:PL:DP 0/1:255,0,255:216 0/1:189,0,255:191 0/1:255,0,255:218 0/1:218,0,255:54 0/1:18,0,142:15 0/1:43,0,83:37 0/1:255,0,255:203

9 3262311 . A T 999 . . GT:PL:DP 0/1:77,0,255:23 0/1:40,0,255:25 0/1:65,0,214:19 0/1:42,0,90:5 0/1:26,3,0:1 0/1:54,6,0:2 0/1:38,0,255:33

9 3293338 . A G 999 . . GT:PL:DP 0/1:253,0,246:36 0/1:179,0,255:42 0/1:228,0,234:29 0/1:38,0,103:8 0/1:53,6,0:2 0/1:77,6,0:2 0/1:89,0,255:38

9 3297776 . A T 999 . . GT:PL:DP 0/1:228,0,123:39 0/1:180,0,208:31 0/1:204,0,197:58 0/1:33,0,46:4 0/1:38,0,106:8 0/1:73,0,97:9 0/1:214,0,255:61

9 3297884 . G A 999 . . GT:PL:DP 0/1:166,0,181:30 0/1:196,0,10:23 0/1:188,0,235:55 0/1:2,0,67:4 0/1:51,0,62:8 0/1:10,7,70:7 0/1:220,0,255:72

9 3495089 . C T 999 . . GT:PL:DP 0/1:98,0,217:232 0/1:35,0,158:224 0/1:71,0,255:223 0/1:88,0,194:67 0/1:26,0,222:49 0/1:68,0,240:49 0/1:136,0,255:216

9 3706248 . A C 999 . . GT:PL:DP 0/1:209,0,140:26 0/1:216,0,151:23 0/1:207,0,213:30 0/1:85,0,52:6 0/1:11,0,86:4 0/1:98,9,0:3 0/1:211,0,253:34

9 3762074 . A C 999 . . GT:PL:DP 0/1:239,0,255:173 0/1:238,0,255:206 0/1:166,0,255:194 0/1:229,0,255:53 0/1:255,0,255:121 0/1:255,0,255:115 0/1:255,0,255:204

9 3774922 . T G 999 . . GT:PL:DP 0/1:173,0,246:27 0/1:125,0,237:19 0/1:203,0,2:22 0/1:36,0,83:6 0/1:30,0,74:5 0/1:67,9,0:3 0/1:202,0,255:32

9 3823377 . A G 999 . . GT:PL:DP 0/1:112,0,81:17 0/1:87,0,150:13 0/1:162,0,56:19 0/1:52,6,0:2 0/1:28,0,65:3 0/1:58,0,1:3 0/1:161,0,212:30

9 3899723 . C T 999 . . GT:PL:DP 0/1:66,0,180:30 0/1:50,0,137:26 0/1:83,0,196:37 0/1:27,0,84:6 0/1:39,9,0:3 0/1:25,0,27:8 0/1:60,0,249:33

9 3900641 . G T 999 . . GT:PL:DP 0/1:122,0,255:209 0/1:128,0,255:210 0/1:105,0,255:204 0/1:157,0,255:207 0/1:188,0,255:170 0/1:156,0,255:170 0/1:71,0,255:181

9 3900693 . C G 999 . . GT:PL:DP 0/1:81,0,255:198 0/1:136,0,231:205 0/1:125,0,245:182 0/1:149,0,255:181 0/1:186,0,255:197 0/1:166,0,255:176 0/1:95,0,244:168

9 3900721 . G A 999 . . GT:PL:DP 0/1:126,0,255:192 0/1:99,0,255:194 0/1:100,0,241:178 0/1:95,0,255:177 0/1:138,0,255:194 0/1:198,0,255:187 0/1:38,0,253:159

9 3900889 . T A 999 . . GT:PL:DP 0/1:71,0,255:219 0/1:19,0,255:215 0/1:96,0,255:205 0/1:136,0,255:215 0/1:116,0,255:222 0/1:166,0,255:218 0/1:87,0,185:181

9 3900968 . T C 999 . . GT:PL:DP 0/1:97,0,255:214 0/1:210,0,255:214 0/1:84,0,236:203 0/1:206,0,255:216 0/1:255,0,255:219 0/1:251,0,255:208 0/1:57,0,175:185

9 3900971 . C T 999 . . GT:PL:DP 0/1:92,0,255:212 0/1:76,0,255:214 0/1:192,0,255:202 0/1:136,0,255:218 0/1:64,0,255:217 0/1:84,0,255:209 0/1:102,0,230:187

9 3931197 . A G 999 . . GT:PL:DP 0/1:154,0,255:243 0/1:124,0,255:248 0/1:43,0,218:250 0/1:153,0,255:219 0/1:176,0,255:173 0/1:51,0,156:211 0/1:172,0,255:251

9 3943629 . C T 999 . . GT:PL:DP 0/1:153,0,210:255 0/1:147,0,247:237 0/1:153,0,255:254 0/1:255,0,255:84 0/1:221,0,255:68 0/1:242,0,255:80 0/1:145,0,241:300

9 3964376 . T A 999 . . GT:PL:DP 0/1:63,0,76:31 0/1:50,0,169:32 0/1:80,0,122:32 0/1:9,0,22:9 0/1:15,0,2:6 0/1:21,0,14:6 0/1:76,0,133:56

9 3964378 . C T 999 . . GT:PL:DP 0/1:63,0,76:32 0/1:50,0,168:32 0/1:83,0,128:32 0/1:9,0,22:9 0/1:17,0,0:6 0/1:22,0,14:7 0/1:81,0,120:56

9 3999647 . A T 999 . . GT:PL:DP 0/1:31,0,27:2 0/1:55,0,54:4 0/1:33,0,30:2 0/1:31,0,30:2 0/1:33,3,0:1 0/1:25,1,0:2 0/1:20,0,84:4

9 4106730 . C T 999 . . GT:PL:DP 0/1:249,0,120:48 0/1:183,0,77:78 0/1:220,4,0:48 0/1:102,0,19:8 0/1:36,3,0:1 0/1:23,0,32:2 0/1:255,0,153:78

9 4130041 . C G 999 . . GT:PL:DP 0/1:63,0,245:81 0/1:160,0,249:68 0/1:12,0,172:69 0/1:51,0,73:13 0/1:31,10,0:13 0/1:47,0,18:10 0/1:92,0,201:65

9 4132142 . C T 999 . . GT:PL:DP 0/1:161,0,255:75 0/1:106,0,255:75 0/1:224,0,255:62 0/1:11,0,139:12 0/1:48,0,67:41 0/1:25,0,103:47 0/1:108,0,255:91

9 4272710 . G A 999 . . GT:PL:DP 0/1:226,0,56:122 0/1:255,0,195:134 0/1:255,0,229:129 0/1:185,0,109:18 0/1:246,0,147:54 0/1:255,0,172:85 0/1:255,0,255:144

9 4418593 . G C 101.571 . . GT:PL:DP 0/1:70,0,69:21 0/1:13,0,60:18 0/1:16,0,81:25 0/1:17,6,0:2 0/1:23,18,9:4 0/1:25,18,9:4 0/1:46,0,64:21

9 4490312 . T A 999 . . GT:PL:DP 0/1:174,0,225:21 0/1:255,0,239:29 0/1:227,0,185:26 0/1:107,0,104:8 0/1:97,9,0:3 0/1:71,6,0:2 0/1:255,0,255:41

9 4516492 . C T 999 . . GT:PL:DP 0/1:73,0,164:38 0/1:106,0,99:26 0/1:116,0,175:31 0/1:41,0,16:7 0/1:34,0,110:24 0/1:53,0,60:56 0/1:122,0,255:98

9 4545354 . T C 999 . . GT:PL:DP 0/1:255,0,255:206 0/1:255,0,255:204 0/1:255,0,237:193 0/1:255,0,176:57 0/1:255,0,190:55 0/1:255,0,81:60 0/1:255,0,255:206

9 4594876 . A T 999 . . GT:PL:DP 0/1:196,0,72:24 0/1:196,0,20:33 0/1:180,1,0:22 0/1:47,6,0:2 0/1:103,0,35:9 0/1:8,6,0:2 0/1:240,0,234:68

9 4723652 . C T 999 . . GT:PL:DP 0/1:165,0,185:29 0/1:136,0,219:28 0/1:144,0,255:39 0/1:39,0,28:3 0/1:12,0,97:5 0/1:44,6,0:2 0/1:172,0,255:34

9 4723684 . G C 999 . . GT:PL:DP 0/1:179,0,175:25 0/1:225,0,224:33 0/1:168,0,255:37 0/1:33,0,89:5 0/1:15,0,91:4 0/1:25,3,0:1 0/1:174,0,255:35

9 4747551 . A C 999 . . GT:PL:DP 0/1:255,0,255:88 0/1:255,0,255:78 0/1:255,0,255:159 0/1:215,0,255:45 0/1:152,0,154:16 0/1:255,0,255:43 0/1:255,0,255:225

9 4747818 . A G 999 . . GT:PL:DP 0/1:100,0,209:36 0/1:79,0,159:32 0/1:182,0,255:68 0/1:100,0,83:12 0/1:7,0,107:14 0/1:68,0,94:20 0/1:252,0,255:189

9 4747930 . A C 999 . . GT:PL:DP 0/1:233,0,140:53 0/1:114,0,255:65 0/1:255,0,255:107 0/1:194,0,224:25 0/1:195,0,87:38 0/1:65,0,127:60 0/1:255,0,226:179

9 4850364 . C T 135.733 . . GT:PL:DP 0/1:55,0,255:27 0/1:63,0,255:47 0/1:21,0,255:45 0/1:25,0,140:13 0/1:16,0,50:6 0/1:19,0,21:6 0/1:48,0,255:95

9 4850491 . G T 999 . . GT:PL:DP 0/1:246,0,204:33 0/1:255,0,255:54 0/1:226,0,228:56 0/1:164,0,145:15 0/1:48,8,0:5 0/1:54,9,0:3 0/1:255,0,255:127

9 4850638 . G A 999 . . GT:PL:DP 0/1:121,0,255:40 0/1:185,0,255:55 0/1:241,0,255:100 0/1:26,0,182:15 0/1:17,0,74:5 0/1:90,0,137:14 0/1:219,0,255:117

9 4982491 . G A 999 . . GT:PL:DP 0/1:157,0,255:77 0/1:239,0,255:66 0/1:227,0,255:98 0/1:79,0,50:6 0/1:14,0,63:4 0/1:43,0,66:5 0/1:153,0,255:114

9 4982613 . C T 999 . . GT:PL:DP 0/1:210,0,255:63 0/1:254,0,255:72 0/1:211,0,255:80 0/1:28,0,65:3 0/1:80,0,82:6 0/1:15,0,125:6 0/1:95,0,255:122

9 5069814 . T G 999 . . GT:PL:DP 0/1:81,0,40:20 0/1:118,0,34:23 0/1:52,0,77:23 0/1:37,0,3:5 0/1:43,6,0:2 0/1:34,0,4:5 0/1:94,0,79:29

9 5069815 . C A 999 . . GT:PL:DP 0/1:57,0,76:19 0/1:118,0,73:23 0/1:49,0,89:24 0/1:37,0,7:5 0/1:13,3,0:1 0/1:34,0,4:5 0/1:96,0,82:29

9 5069818 . C T 999 . . GT:PL:DP 0/1:57,0,70:19 0/1:51,0,72:20 0/1:24,0,103:24 0/1:37,0,7:5 0/1:13,3,0:1 0/1:34,0,4:5 0/1:62,0,78:28

9 5156457 . C T 999 . . GT:PL:DP 0/1:170,0,255:38 0/1:118,0,255:31 0/1:67,0,255:36 0/1:36,0,27:2 0/1:100,0,60:11 0/1:35,0,197:13 0/1:255,0,111:42

9 5185434 . C A 999 . . GT:PL:DP 0/1:107,0,127:17 0/1:98,0,192:29 0/1:191,0,161:24 0/1:43,6,0:2 0/1:27,3,0:1 0/1:28,3,0:1 0/1:142,0,189:24

9 5227477 . C T 999 . . GT:PL:DP 0/1:121,0,94:60 0/1:48,0,129:47 0/1:96,0,105:55 0/1:25,0,30:4 0/1:15,0,28:10 0/1:94,0,100:26 0/1:110,0,148:74

9 5268512 . C A 999 . . GT:PL:DP 0/1:255,0,255:59 0/1:255,0,255:59 0/1:255,0,255:68 0/1:132,0,200:15 0/1:42,0,114:7 0/1:128,0,85:9 0/1:255,0,255:117

9 5268534 . T C 999 . . GT:PL:DP 0/1:133,0,255:55 0/1:115,0,255:58 0/1:229,0,255:64 0/1:35,0,159:12 0/1:71,0,76:9 0/1:41,0,122:9 0/1:205,0,255:120

9 5268934 . T C 999 . . GT:PL:DP 0/1:108,0,19:6 0/1:59,0,28:3 0/1:125,0,16:7 0/1:37,3,0:1 0/1:40,3,0:1 0/1:41,6,0:2 0/1:82,0,120:9

9 5368504 . C T 999 . . GT:PL:DP 0/1:224,0,227:41 0/1:191,0,207:22 0/1:161,0,183:22 0/1:46,0,132:12 0/1:76,9,0:3 0/1:8,3,0:1 0/1:152,0,255:38

9 5417106 . G A 999 . . GT:PL:DP 0/1:176,0,189:33 0/1:119,0,125:17 0/1:104,0,255:42 0/1:22,0,81:5 0/1:50,9,0:3 0/1:20,3,0:1 0/1:138,0,156:37

9 5472355 . A C 999 . . GT:PL:DP 0/1:255,0,255:174 0/1:255,0,255:158 0/1:255,0,255:180 0/1:93,0,255:39 0/1:111,0,239:34 0/1:205,0,255:71 0/1:157,0,255:211

9 5721310 . G A 999 . . GT:PL:DP 0/1:216,0,255:32 0/1:255,0,255:38 0/1:227,0,255:32 0/1:16,0,141:7 0/1:57,6,0:2 0/1:46,0,26:3 0/1:255,0,255:55

9 5831352 . G A 999 . . GT:PL:DP 0/1:185,0,250:35 0/1:159,0,178:21 0/1:222,0,109:26 0/1:74,0,46:6 0/1:50,0,69:5 0/1:32,3,0:1 0/1:180,0,255:73

9 5904315 . C T 999 . . GT:PL:DP 0/1:82,0,255:39 0/1:87,0,255:36 0/1:140,0,255:35 0/1:64,0,158:12 0/1:29,0,138:8 0/1:9,0,91:4 0/1:166,0,255:50

9 5904859 . T A 999 . . GT:PL:DP 0/1:255,0,255:73 0/1:213,0,255:88 0/1:255,0,255:66 0/1:100,0,244:20 0/1:173,0,215:20 0/1:30,0,181:11 0/1:255,0,255:99

9 5904871 . A T 999 . . GT:PL:DP 0/1:255,0,255:75 0/1:255,0,217:87 0/1:255,0,255:68 0/1:248,0,133:20 0/1:212,0,183:21 0/1:187,0,113:14 0/1:255,0,255:90

9 5904987 . G A 999 . . GT:PL:DP 0/1:213,0,255:71 0/1:204,0,174:61 0/1:217,0,255:62 0/1:154,0,134:16 0/1:155,0,80:16 0/1:22,0,197:15 0/1:219,0,255:102

9 5905632 . C A 999 . . GT:PL:DP 0/1:255,0,255:73 0/1:255,0,255:61 0/1:230,0,255:70 0/1:15,0,145:7 0/1:82,0,97:7 0/1:59,0,4:3 0/1:215,0,255:80

9 5912011 . C T 999 . . GT:PL:DP 0/1:143,0,32:14 0/1:148,5,0:14 0/1:54,0,25:3 0/1:37,3,0:1 0/1:80,0,1:5 0/1:86,0,49:6 0/1:148,0,63:17

9 5985443 . G T 999 . . GT:PL:DP 0/1:212,0,74:20 0/1:174,0,174:21 0/1:189,0,83:27 0/1:36,0,27:3 0/1:13,3,0:1 0/1:54,6,0:2 0/1:162,0,255:43

9 6311978 . G A 999 . . GT:PL:DP 0/1:40,0,83:24 0/1:120,0,10:18 0/1:29,0,72:21 0/1:13,0,6:3 0/1:17,1,0:3 0/1:23,3,0:1 0/1:56,0,80:27

9 6328321 . T C 999 . . GT:PL:DP 0/1:255,0,255:40 0/1:255,0,201:40 0/1:255,0,235:41 0/1:19,0,116:6 0/1:90,0,41:6 0/1:100,9,0:3 0/1:255,0,246:62

9 6742057 . G A 999 . . GT:PL:DP 0/1:68,0,105:17 0/1:73,0,64:20 0/1:104,0,118:28 0/1:15,0,50:5 0/1:50,9,0:3 0/1:68,9,0:3 0/1:66,0,140:31

9 6763488 . C G 999 . . GT:PL:DP 0/1:135,0,69:9 0/1:137,0,132:13 0/1:35,0,172:11 0/1:12,0,31:2 0/1:31,3,0:1 0/1:26,0,28:2 0/1:146,0,73:10

9 6835266 . A T 999 . . GT:PL:DP 0/1:98,0,102:27 0/1:80,0,124:22 0/1:53,0,49:9 0/1:46,6,0:3 0/1:55,6,0:2 0/1:63,9,0:3 0/1:109,0,12:36

9 6835294 . T G 999 . . GT:PL:DP 0/1:104,0,10:36 0/1:50,0,109:24 0/1:69,0,0:16 0/1:23,1,0:5 0/1:43,0,7:3 0/1:35,6,0:2 0/1:46,0,42:53

9 6959839 . A G 999 . . GT:PL:DP 0/1:126,0,255:217 0/1:255,0,255:204 0/1:255,0,255:223 0/1:162,0,255:44 0/1:183,0,255:57 0/1:141,0,255:127 0/1:232,0,255:216

9 6959902 . C T 999 . . GT:PL:DP 0/1:176,0,255:237 0/1:131,0,255:228 0/1:113,0,255:235 0/1:145,0,255:40 0/1:100,0,255:61 0/1:153,0,255:130 0/1:213,0,255:225

9 6960226 . A G 999 . . GT:PL:DP 0/1:255,0,255:230 0/1:255,0,255:221 0/1:255,0,255:235 0/1:255,0,255:69 0/1:255,0,255:63 0/1:255,0,255:162 0/1:255,0,255:224

9 6962894 . A G 999 . . GT:PL:DP 0/1:255,0,255:213 0/1:255,0,255:176 0/1:255,0,255:206 0/1:255,0,151:39 0/1:157,0,22:17 0/1:255,0,207:82 0/1:255,0,255:211

9 7032001 . C T 999 . . GT:PL:DP 0/1:255,0,255:71 0/1:57,0,255:83 0/1:240,0,186:46 0/1:148,0,179:26 0/1:119,0,167:14 0/1:9,0,255:21 0/1:255,0,195:188

9 7032525 . A G 999 . . GT:PL:DP 0/1:255,0,255:125 0/1:255,0,178:125 0/1:255,0,255:114 0/1:255,0,230:36 0/1:196,0,255:38 0/1:252,0,255:44 0/1:255,0,255:187

9 7032726 . A G 999 . . GT:PL:DP 0/1:255,0,255:106 0/1:255,0,109:104 0/1:255,0,173:99 0/1:166,0,221:25 0/1:241,0,255:29 0/1:122,0,255:30 0/1:255,0,255:195

9 7099635 . G A 149.26 . . GT:PL:DP 0/1:22,3,0:1 0/1:16,0,15:2 0/1:30,0,52:6 0/1:24,3,0:1 0/1:56,9,0:3 0/1:40,6,0:2 0/1:81,5,0:8

9 7111252 . G A 999 . . GT:PL:DP 0/1:137,0,139:146 0/1:124,0,114:135 0/1:135,0,119:142 0/1:97,0,47:11 0/1:55,0,39:23 0/1:29,2,0:6 0/1:102,0,242:105

9 7134810 . A T 999 . . GT:PL:DP 0/1:83,0,255:52 0/1:191,0,226:34 0/1:72,38,255:60 0/1:64,0,61:7 0/1:61,0,83:7 0/1:75,9,0:3 0/1:248,0,255:81

9 7135232 . A G 999 . . GT:PL:DP 0/1:77,0,161:56 0/1:91,0,115:57 0/1:150,0,109:44 0/1:53,0,28:10 0/1:40,5,0:11 0/1:21,6,0:3 0/1:118,0,158:59

9 7135234 . A G 999 . . GT:PL:DP 0/1:79,0,176:57 0/1:97,0,128:55 0/1:156,0,121:44 0/1:45,0,34:9 0/1:43,0,6:10 0/1:33,9,0:3 0/1:119,0,175:59

9 7135388 . T C 999 . . GT:PL:DP 0/1:255,0,63:65 0/1:255,0,183:50 0/1:255,0,18:40 0/1:66,0,107:7 0/1:191,0,62:12 0/1:76,0,20:5 0/1:255,0,192:54

9 7136303 . A T 999 . . GT:PL:DP 0/1:84,0,55:11 0/1:114,0,53:18 0/1:61,0,123:15 0/1:68,9,0:3 0/1:43,0,51:4 0/1:74,0,112:11 0/1:164,0,63:25

9 7141261 . G A 999 . . GT:PL:DP 0/1:255,5,0:37 0/1:255,0,37:43 0/1:255,0,46:48 0/1:150,0,13:8 0/1:70,0,128:9 0/1:226,0,53:19 0/1:255,0,255:52

9 7161174 . G A 999 . . GT:PL:DP 0/1:255,0,255:160 0/1:138,0,255:128 0/1:255,0,255:157 0/1:160,0,124:17 0/1:37,0,136:62 0/1:42,0,128:67 0/1:159,0,255:205

9 7161229 . T A 999 . . GT:PL:DP 0/1:255,0,255:177 0/1:249,0,255:143 0/1:255,0,255:144 0/1:149,0,247:20 0/1:255,0,242:96 0/1:255,0,255:99 0/1:255,0,255:185

9 7221567 . A T 999 . . GT:PL:DP 0/1:97,0,164:22 0/1:193,0,106:23 0/1:150,0,14:14 0/1:21,0,23:2 0/1:29,0,17:5 0/1:40,6,0:2 0/1:131,0,146:26

9 7394782 . C G 999 . . GT:PL:DP 0/1:90,0,26:10 0/1:122,0,27:13 0/1:82,0,4:9 0/1:45,9,0:3 0/1:11,0,17:3 0/1:33,0,2:4 0/1:132,0,12:17

9 7414705 . G A 999 . . GT:PL:DP 0/1:41,0,88:219 0/1:27,0,154:161 0/1:35,0,64:184 0/1:56,8,0:35 0/1:75,0,66:70 0/1:65,7,0:58 0/1:40,0,102:203

9 7434354 . C T 999 . . GT:PL:DP 0/1:255,0,161:60 0/1:237,0,111:129 0/1:255,0,119:57 0/1:134,0,52:12 0/1:155,0,47:41 0/1:169,0,101:28 0/1:255,0,255:112

9 7434469 . T C 999 . . GT:PL:DP 0/1:255,0,255:98 0/1:255,0,255:132 0/1:240,0,255:73 0/1:144,0,140:20 0/1:244,0,255:67 0/1:255,0,255:87 0/1:255,0,255:123

9 7434841 . T A 999 . . GT:PL:DP 0/1:255,0,77:55 0/1:255,0,227:78 0/1:255,0,83:55 0/1:137,0,27:8 0/1:255,0,19:32 0/1:255,0,69:31 0/1:255,0,67:80

9 7450797 . T C 999 . . GT:PL:DP 0/1:41,0,212:251 0/1:45,0,204:260 0/1:91,0,208:268 0/1:11,0,141:22 0/1:47,0,170:36 0/1:7,0,152:23 0/1:28,0,206:129

9 7450808 . C T 999 . . GT:PL:DP 0/1:43,0,210:256 0/1:50,0,204:259 0/1:86,0,202:270 0/1:30,0,153:24 0/1:25,0,162:39 0/1:17,0,161:23 0/1:13,0,176:131

9 7459010 . G A 999 . . GT:PL:DP 0/1:227,0,169:34 0/1:138,0,235:54 0/1:235,0,29:35 0/1:44,0,81:10 0/1:131,0,162:22 0/1:210,0,50:35 0/1:255,0,66:42

9 7459136 . T A 999 . . GT:PL:DP 0/1:144,0,171:22 0/1:147,0,255:36 0/1:255,0,69:41 0/1:82,0,66:7 0/1:76,0,190:14 0/1:255,0,1:18 0/1:255,0,58:39

9 7464016 . A G 999 . . GT:PL:DP 0/1:209,0,104:33 0/1:228,0,213:54 0/1:218,0,104:43 0/1:64,0,9:4 0/1:105,0,72:16 0/1:167,0,147:24 0/1:138,0,146:37

9 7471651 . G C 999 . . GT:PL:DP 0/1:9,0,255:36 0/1:112,0,58:31 0/1:13,0,249:31 0/1:17,0,133:8 0/1:20,0,92:20 0/1:28,0,109:19 0/1:142,0,106:30

9 7471669 . C T 999 . . GT:PL:DP 0/1:21,0,255:43 0/1:97,0,151:42 0/1:106,0,255:36 0/1:7,0,150:8 0/1:106,0,74:24 0/1:110,0,111:26 0/1:121,0,164:33

9 7471705 . T A 999 . . GT:PL:DP 0/1:11,0,255:37 0/1:103,0,166:38 0/1:66,0,255:37 0/1:10,0,99:7 0/1:111,0,107:22 0/1:71,0,198:27 0/1:117,0,197:34

9 7490181 . T A 999 . . GT:PL:DP 0/1:103,0,121:24 0/1:23,0,102:15 0/1:126,0,102:24 0/1:37,3,0:1 0/1:66,3,0:3 0/1:24,3,0:1 0/1:138,0,43:11

9 7560812 . T A 999 . . GT:PL:DP 0/1:127,0,160:91 0/1:135,0,201:101 0/1:103,0,77:88 0/1:56,0,96:22 0/1:64,0,25:11 0/1:93,0,79:22 0/1:67,0,94:220

9 7567939 . C T 999 . . GT:PL:DP 0/1:255,0,138:37 0/1:53,0,250:27 0/1:156,0,212:28 0/1:91,0,10:9 0/1:12,0,158:10 0/1:49,0,189:20 0/1:255,0,255:110

9 7610906 . T G 999 . . GT:PL:DP 0/1:187,0,255:33 0/1:255,0,154:41 0/1:140,0,255:32 0/1:54,0,2:3 0/1:202,0,14:13 0/1:212,6,0:13 0/1:242,0,255:44

9 7712555 . A G 999 . . GT:PL:DP 0/1:128,0,255:49 0/1:99,0,255:51 0/1:192,0,255:58 0/1:20,0,172:9 0/1:170,0,155:13 0/1:179,0,227:21 0/1:255,0,255:87

9 7754808 . T C 999 . . GT:PL:DP 0/1:160,0,255:30 0/1:180,0,255:40 0/1:244,0,155:31 0/1:80,0,100:8 0/1:75,0,100:12 0/1:92,12,0:4 0/1:218,0,255:56

9 8053675 . T A 999 . . GT:PL:DP 0/1:106,0,227:33 0/1:57,0,119:20 0/1:145,0,201:31 0/1:10,0,71:5 0/1:35,0,34:4 0/1:21,3,0:1 0/1:159,0,139:33

9 8099982 . G A 999 . . GT:PL:DP 0/1:199,0,4:37 0/1:162,0,93:18 0/1:172,0,12:18 0/1:72,9,0:3 0/1:42,0,10:4 0/1:22,0,15:3 0/1:215,2,0:43

9 8637475 . G T 999 . . GT:PL:DP 0/1:42,0,91:20 0/1:36,0,51:12 0/1:46,0,100:26 0/1:41,0,23:7 0/1:30,0,99:8 0/1:30,0,157:22 0/1:62,0,100:22

9 8637633 . A G 999 . . GT:PL:DP 0/1:38,0,126:28 0/1:33,0,118:22 0/1:37,0,98:26 0/1:40,0,46:9 0/1:47,0,78:9 0/1:53,0,142:20 0/1:86,0,48:18

9 8637638 . G A 999 . . GT:PL:DP 0/1:35,0,133:31 0/1:44,0,112:23 0/1:40,0,103:25 0/1:40,0,33:9 0/1:41,0,100:11 0/1:58,0,136:21 0/1:82,0,67:19

9 8637639 . G A 999 . . GT:PL:DP 0/1:26,0,140:30 0/1:44,0,112:23 0/1:28,0,109:25 0/1:40,0,46:9 0/1:41,0,97:11 0/1:58,0,134:21 0/1:83,0,69:19

9 8637671 . C T 999 . . GT:PL:DP 0/1:85,0,112:31 0/1:78,0,81:20 0/1:23,0,119:26 0/1:40,0,42:8 0/1:58,0,84:10 0/1:36,0,127:22 0/1:67,0,63:19

9 8637692 . G C 999 . . GT:PL:DP 0/1:9,0,116:22 0/1:60,0,85:16 0/1:17,0,121:19 0/1:15,0,41:4 0/1:58,0,72:10 0/1:53,0,100:16 0/1:39,0,62:12

9 8643578 . T C 999 . . GT:PL:DP 0/1:255,0,255:242 0/1:255,0,255:228 0/1:237,0,255:228 0/1:255,0,255:95 0/1:255,0,255:93 0/1:255,0,255:149 0/1:255,0,255:237

9 8740680 . T G 999 . . GT:PL:DP 0/1:255,0,249:108 0/1:166,0,255:81 0/1:234,0,232:82 0/1:109,0,185:21 0/1:54,0,151:25 0/1:103,0,117:30 0/1:255,0,255:90

9 8962324 . G A 999 . . GT:PL:DP 0/1:122,0,215:26 0/1:148,0,237:28 0/1:121,0,195:28 0/1:53,0,25:3 0/1:12,0,83:4 0/1:79,0,97:9 0/1:108,0,255:50

9 8962326 . A T 999 . . GT:PL:DP 0/1:122,0,219:26 0/1:142,0,230:28 0/1:132,0,195:28 0/1:34,0,16:3 0/1:12,0,83:4 0/1:79,0,100:9 0/1:138,0,255:50

9 8962327 . A T 999 . . GT:PL:DP 0/1:120,0,215:26 0/1:136,0,241:28 0/1:147,0,194:29 0/1:53,0,25:3 0/1:12,0,83:4 0/1:79,0,100:9 0/1:150,0,255:51

9 8962328 . A T 999 . . GT:PL:DP 0/1:122,0,219:26 0/1:142,0,235:28 0/1:145,0,212:30 0/1:53,0,25:3 0/1:12,0,73:4 0/1:76,0,99:10 0/1:156,0,255:51

9 8962348 . A C 999 . . GT:PL:DP 0/1:115,0,167:25 0/1:141,0,232:33 0/1:165,0,168:31 0/1:41,0,25:3 0/1:69,0,45:10 0/1:73,0,24:11 0/1:127,0,255:56

9 9090663 . A G 999 . . GT:PL:DP 0/1:70,0,219:23 0/1:35,0,173:20 0/1:38,0,255:19 0/1:67,12,0:4 0/1:18,9,0:3 0/1:27,3,0:1 0/1:56,0,255:38

9 9202381 . A T 999 . . GT:PL:DP 0/1:47,0,197:20 0/1:91,0,68:13 0/1:142,4,0:11 0/1:69,0,16:4 0/1:42,6,0:2 0/1:39,0,6:3 0/1:147,0,31:15

9 9248372 . T A 999 . . GT:PL:DP 0/1:192,0,255:214 0/1:255,0,233:218 0/1:159,0,215:216 0/1:191,0,250:88 0/1:210,0,116:40 0/1:177,0,164:46 0/1:218,0,196:205

9 9248606 . T A 999 . . GT:PL:DP 0/1:103,0,255:214 0/1:192,0,255:200 0/1:85,0,255:205 0/1:139,0,255:112 0/1:62,0,255:29 0/1:54,0,255:46 0/1:120,0,255:205

9 9248707 . C A 999 . . GT:PL:DP 0/1:255,0,255:215 0/1:255,0,248:223 0/1:255,0,212:211 0/1:255,0,255:131 0/1:255,0,255:76 0/1:255,0,255:89 0/1:255,0,255:201

9 9248831 . T A 999 . . GT:PL:DP 0/1:255,0,255:173 0/1:255,0,147:166 0/1:255,0,154:185 0/1:255,0,246:93 0/1:248,0,156:52 0/1:255,0,111:58 0/1:255,0,173:180

9 9248853 . G A 999 . . GT:PL:DP 0/1:255,0,255:151 0/1:224,0,110:149 0/1:199,0,69:182 0/1:255,0,225:80 0/1:68,0,49:26 0/1:61,0,16:33 0/1:222,0,112:195

9 9248994 . T A 999 . . GT:PL:DP 0/1:255,0,232:213 0/1:255,0,209:209 0/1:255,0,138:210 0/1:255,0,143:57 0/1:138,0,119:16 0/1:173,0,26:17 0/1:255,0,127:191

9 9249062 . T G 999 . . GT:PL:DP 0/1:255,0,255:203 0/1:255,0,255:193 0/1:255,0,255:207 0/1:178,0,255:56 0/1:136,0,221:29 0/1:188,0,226:39 0/1:255,0,255:201

9 9432125 . G A 999 . . GT:PL:DP 0/1:156,0,29:25 0/1:102,0,20:16 0/1:38,0,28:9 0/1:62,6,0:2 0/1:28,3,0:1 0/1:20,3,0:1 0/1:152,0,24:42

9 9489073 . G A 999 . . GT:PL:DP 0/1:105,0,70:10 0/1:49,0,163:10 0/1:86,0,50:8 0/1:7,0,10:3 0/1:57,0,19:4 0/1:48,0,72:4 0/1:255,0,99:23

9 9664061 . T A 999 . . GT:PL:DP 0/1:239,0,255:39 0/1:246,0,255:38 0/1:227,0,255:34 0/1:118,0,107:10 0/1:50,0,48:4 0/1:62,6,0:2 0/1:255,0,255:41

9 9665934 . G A 999 . . GT:PL:DP 0/1:248,0,211:69 0/1:255,0,236:95 0/1:223,0,255:122 0/1:158,0,41:8 0/1:173,0,173:18 0/1:255,0,220:40 0/1:255,0,255:211

9 9666399 . A T 999 . . GT:PL:DP 0/1:25,0,101:14 0/1:119,69,144:42 0/1:100,0,196:42 0/1:30,6,1:7 0/1:52,6,0:2 0/1:27,0,37:8 0/1:90,0,241:60

9 9667405 . C A 999 . . GT:PL:DP 0/1:35,6,0:2 0/1:90,0,43:8 0/1:92,0,79:9 0/1:37,3,0:1 0/1:29,0,23:3 0/1:63,8,0:5 0/1:127,0,38:17

9 9733852 . A G 999 . . GT:PL:DP 0/1:104,0,188:49 0/1:156,0,255:51 0/1:111,0,211:52 0/1:82,0,42:5 0/1:111,0,74:12 0/1:40,0,48:9 0/1:16,0,255:46

9 9850265 . T C 999 . . GT:PL:DP 0/1:81,0,1:4 0/1:67,0,37:6 0/1:102,0,89:15 0/1:18,0,35:4 0/1:79,9,0:3 0/1:37,6,0:2 0/1:84,0,216:53

9 9953089 . G A 999 . . GT:PL:DP 0/1:185,0,142:33 0/1:94,0,181:35 0/1:129,0,116:30 0/1:35,0,75:8 0/1:45,9,0:3 0/1:46,5,0:4 0/1:134,0,166:41

9 10061060 . A G 999 . . GT:PL:DP 0/1:5,0,4:3 0/1:21,0,36:4 0/1:41,15,12:2 0/1:35,3,0:1 0/1:34,3,0:1 0/1:25,3,0:1 0/1:87,0,161:15

9 10280101 . G A 999 . . GT:PL:DP 0/1:40,0,94:13 0/1:29,0,34:4 0/1:68,0,31:9 0/1:66,0,31:4 0/1:32,0,132:16 0/1:57,0,226:35 0/1:30,0,55:12

9 10413341 . A G 999 . . GT:PL:DP 0/1:255,0,255:210 0/1:255,0,255:225 0/1:255,0,255:232 0/1:158,0,255:41 0/1:32,0,235:105 0/1:113,0,219:109 0/1:255,0,255:235

9 10468690 . G C 999 . . GT:PL:DP 0/1:27,0,182:112 0/1:73,0,253:119 0/1:65,0,160:122 0/1:27,0,134:23 0/1:88,0,246:41 0/1:61,0,210:51 0/1:62,0,250:129

9 10468948 . C A 999 . . GT:PL:DP 0/1:206,0,255:126 0/1:218,0,255:140 0/1:206,0,255:131 0/1:117,0,200:23 0/1:79,0,210:67 0/1:77,0,231:70 0/1:231,0,255:183

9 10490360 . C T 999 . . GT:PL:DP 0/1:89,0,255:69 0/1:214,0,244:48 0/1:222,0,211:54 0/1:45,0,122:8 0/1:83,0,50:7 0/1:11,0,90:8 0/1:148,0,178:52

9 10490433 . A G 999 . . GT:PL:DP 0/1:130,0,158:91 0/1:118,0,33:51 0/1:187,0,109:68 0/1:90,0,78:9 0/1:67,0,19:7 0/1:118,0,31:13 0/1:186,0,165:73

9 10583527 . A T 999 . . GT:PL:DP 0/1:255,0,48:114 0/1:255,0,210:223 0/1:255,0,239:139 0/1:30,0,96:14 0/1:255,0,64:49 0/1:255,0,17:56 0/1:255,0,238:161

9 10669881 . A G 999 . . GT:PL:DP 0/1:164,0,111:18 0/1:145,0,134:14 0/1:192,0,148:22 0/1:108,0,16:6 0/1:79,0,55:5 0/1:68,6,0:2 0/1:214,0,146:25

9 10734329 . T C 999 . . GT:PL:DP 0/1:55,0,255:84 0/1:255,0,173:46 0/1:209,0,255:33 0/1:43,0,80:6 0/1:83,0,151:17 0/1:122,0,255:66 0/1:196,0,255:122

9 10734363 . T A 999 . . GT:PL:DP 0/1:255,0,115:82 0/1:103,0,255:57 0/1:250,0,213:40 0/1:96,0,36:7 0/1:103,0,142:15 0/1:255,0,135:72 0/1:255,0,196:113

9 10734861 . T A 999 . . GT:PL:DP 0/1:254,0,46:28 0/1:227,0,252:44 0/1:202,0,197:30 0/1:49,0,97:8 0/1:82,0,15:8 0/1:46,0,74:9 0/1:255,0,255:117

9 10734862 . C T 999 . . GT:PL:DP 0/1:255,0,46:28 0/1:225,0,247:44 0/1:199,0,193:30 0/1:49,0,97:8 0/1:82,0,15:8 0/1:24,0,81:8 0/1:255,0,255:116

9 10735301 . G A 999 . . GT:PL:DP 0/1:255,0,156:81 0/1:243,0,255:113 0/1:251,0,255:92 0/1:159,0,163:17 0/1:246,0,121:23 0/1:92,0,50:7 0/1:255,0,255:111

9 10735452 . G A 999 . . GT:PL:DP 0/1:255,0,184:112 0/1:255,0,255:133 0/1:255,0,255:142 0/1:116,0,196:15 0/1:105,0,40:15 0/1:84,0,93:10 0/1:255,0,255:189

9 10737294 . A G 999 . . GT:PL:DP 0/1:255,0,214:124 0/1:255,0,255:124 0/1:255,0,255:126 0/1:117,0,239:33 0/1:200,0,50:14 0/1:186,0,54:13 0/1:255,0,255:196

9 10737425 . C A 999 . . GT:PL:DP 0/1:200,0,189:70 0/1:246,0,251:112 0/1:193,0,210:93 0/1:102,0,102:13 0/1:29,0,26:3 0/1:52,0,212:18 0/1:171,0,255:140

9 10903453 . G A 999 . . GT:PL:DP 0/1:255,0,255:165 0/1:226,0,215:158 0/1:218,0,255:125 0/1:124,0,135:15 0/1:200,0,12:34 0/1:136,0,77:25 0/1:180,0,159:201

9 10903474 . A G 999 . . GT:PL:DP 0/1:221,0,255:133 0/1:241,0,255:131 0/1:172,0,255:93 0/1:92,0,112:13 0/1:128,0,10:19 0/1:47,0,112:11 0/1:139,0,117:173

9 10903480 . C A 999 . . GT:PL:DP 0/1:216,0,255:133 0/1:240,0,255:128 0/1:189,0,255:88 0/1:71,0,128:11 0/1:154,0,2:20 0/1:51,0,111:9 0/1:156,0,128:159

9 10903501 . A T 999 . . GT:PL:DP 0/1:226,0,195:116 0/1:252,0,255:102 0/1:178,0,255:80 0/1:49,0,60:8 0/1:178,79,73:18 0/1:37,30,50:10 0/1:157,0,12:142

9 10903626 . A G 999 . . GT:PL:DP 0/1:255,0,218:71 0/1:94,0,255:49 0/1:208,0,255:60 0/1:13,0,118:8 0/1:116,12,13:11 0/1:16,0,52:11 0/1:255,0,87:109

9 10904027 . T C 999 . . GT:PL:DP 0/1:165,0,244:134 0/1:176,0,255:118 0/1:137,0,191:103 0/1:30,0,174:23 0/1:75,0,89:29 0/1:27,0,77:24 0/1:239,0,255:129

9 10905123 . A G 999 . . GT:PL:DP 0/1:229,0,255:35 0/1:255,0,255:66 0/1:159,0,188:43 0/1:96,0,84:7 0/1:58,3,0:24 0/1:69,0,13:32 0/1:255,0,255:89

9 10905382 . A T 999 . . GT:PL:DP 0/1:85,0,234:19 0/1:255,0,117:37 0/1:132,0,255:31 0/1:180,0,34:10 0/1:255,9,0:14 0/1:148,0,40:9 0/1:255,0,255:84

9 10905460 . T A 999 . . GT:PL:DP 0/1:64,0,112:12 0/1:124,0,117:23 0/1:92,0,88:16 0/1:47,0,16:3 0/1:34,6,0:2 0/1:51,0,53:5 0/1:79,0,255:46

9 10905592 . C G 999 . . GT:PL:DP 0/1:48,0,232:31 0/1:177,0,184:32 0/1:46,0,233:51 0/1:46,0,0:8 0/1:115,5,0:15 0/1:13,0,48:9 0/1:103,0,255:64

9 10909422 . G A 999 . . GT:PL:DP 0/1:255,0,255:217 0/1:255,0,255:218 0/1:255,0,255:179 0/1:255,0,255:48 0/1:255,0,22:65 0/1:255,0,227:59 0/1:255,0,255:217

9 10909437 . C T 999 . . GT:PL:DP 0/1:255,0,255:226 0/1:255,0,255:224 0/1:255,0,255:187 0/1:255,0,255:44 0/1:255,0,27:67 0/1:255,0,255:74 0/1:255,0,255:217

9 10909547 . G A 999 . . GT:PL:DP 0/1:255,0,255:217 0/1:255,0,255:225 0/1:255,0,255:195 0/1:255,0,255:49 0/1:255,0,103:86 0/1:255,0,255:107 0/1:255,0,255:217

9 11002383 . C G 999 . . GT:PL:DP 0/1:27,0,154:12 0/1:17,0,201:22 0/1:114,0,203:31 0/1:18,0,55:4 0/1:21,0,23:2 0/1:41,0,20:3 0/1:17,0,238:31

9 11069265 . G C 999 . . GT:PL:DP 0/1:255,0,255:157 0/1:255,0,255:156 0/1:255,0,243:162 0/1:164,0,199:27 0/1:47,0,71:7 0/1:148,0,32:9 0/1:255,0,255:213

9 11069299 . C T 999 . . GT:PL:DP 0/1:255,0,225:186 0/1:255,0,255:192 0/1:255,0,255:209 0/1:171,0,177:35 0/1:28,0,60:16 0/1:129,0,38:17 0/1:255,0,255:239

9 11069391 . G A 999 . . GT:PL:DP 0/1:255,0,191:127 0/1:255,0,255:149 0/1:247,0,235:134 0/1:152,0,117:29 0/1:51,5,0:11 0/1:14,3,0:16 0/1:255,0,255:145

9 11311911 . G A 999 . . GT:PL:DP 0/1:13,0,255:37 0/1:57,0,255:35 0/1:78,0,255:42 0/1:25,0,19:2 0/1:34,3,0:1 0/1:58,0,247:19 0/1:157,0,255:62

9 11315205 . T C 999 . . GT:PL:DP 0/1:255,0,255:207 0/1:255,0,255:167 0/1:255,0,255:198 0/1:233,0,217:27 0/1:178,0,188:25 0/1:205,0,205:36 0/1:255,0,255:192

9 11455160 . G T 999 . . GT:PL:DP 0/1:5,0,154:41 0/1:155,0,146:70 0/1:151,0,130:99 0/1:11,0,139:8 0/1:17,0,103:15 0/1:14,0,119:12 0/1:159,0,159:76

9 11605387 . G A 999 . . GT:PL:DP 0/1:180,0,229:55 0/1:207,0,195:46 0/1:226,0,201:37 0/1:95,0,76:10 0/1:10,0,66:5 0/1:65,0,87:11 0/1:200,0,218:47

9 11605488 . T C 999 . . GT:PL:DP 0/1:252,0,255:58 0/1:255,0,237:61 0/1:255,0,255:55 0/1:51,0,111:9 0/1:148,0,17:6 0/1:125,0,179:13 0/1:255,0,224:47

9 11606193 . G C 999 . . GT:PL:DP 0/1:255,0,255:67 0/1:255,0,255:57 0/1:255,0,255:50 0/1:108,0,105:9 0/1:170,0,41:10 0/1:7,0,185:9 0/1:255,0,255:54

9 11606451 . C T 999 . . GT:PL:DP 0/1:76,0,255:40 0/1:186,0,193:39 0/1:173,0,209:31 0/1:49,0,151:8 0/1:43,0,187:9 0/1:44,0,86:7 0/1:212,0,249:43

9 11848057 . T A 999 . . GT:PL:DP 0/1:127,0,78:19 0/1:148,0,168:31 0/1:178,0,130:31 0/1:63,0,69:7 0/1:76,9,0:3 0/1:66,9,0:3 0/1:211,0,197:37

9 11984193 . A C 77.7345 . . GT:PL:DP 0/1:17,6,0:2 0/1:20,0,11:2 0/1:53,12,0:4 0/1:16,3,0:1 0/1:14,3,0:1 0/1:20,3,0:1 0/1:57,0,13:5

9 12075685 . G A 999 . . GT:PL:DP 0/1:255,0,177:76 0/1:255,0,161:59 0/1:255,0,98:49 0/1:22,0,53:5 0/1:65,0,19:12 0/1:24,0,15:8 0/1:255,0,172:105

9 12098857 . T A 999 . . GT:PL:DP 0/1:8,0,168:31 0/1:117,0,178:25 0/1:53,0,148:27 0/1:13,0,51:4 0/1:27,0,23:3 0/1:38,0,48:7 0/1:76,0,166:30

9 12125395 . T C 999 . . GT:PL:DP 0/1:255,0,255:161 0/1:158,0,255:156 0/1:169,0,255:175 0/1:123,0,231:22 0/1:43,0,228:27 0/1:127,0,197:34 0/1:133,0,255:172

10 11909 . G A 999 . . GT:PL:DP 0/1:225,0,224:64 0/1:93,0,254:40 0/1:167,0,224:51 0/1:11,0,28:3 0/1:65,0,197:15 0/1:129,0,249:29 0/1:40,0,255:79

10 101580 . T C 999 . . GT:PL:DP 0/1:255,0,255:215 0/1:255,0,255:218 0/1:255,0,255:209 0/1:255,0,241:200 0/1:255,0,188:156 0/1:255,0,148:125 0/1:255,0,255:198

10 208481 . T G 999 . . GT:PL:DP 0/1:104,0,97:33 0/1:94,0,115:22 0/1:136,0,145:40 0/1:62,0,75:7 0/1:34,6,0:2 0/1:17,0,39:7 0/1:149,102,215:36

10 225109 . T C 999 . . GT:PL:DP 0/1:191,0,173:58 0/1:164,0,159:55 0/1:85,0,212:67 0/1:137,7,0:9 0/1:83,0,47:13 0/1:37,0,178:29 0/1:255,0,255:158

10 225352 . C T 999 . . GT:PL:DP 0/1:160,0,255:58 0/1:69,0,255:77 0/1:189,0,255:67 0/1:38,0,201:10 0/1:168,0,146:17 0/1:255,0,227:31 0/1:9,0,255:186

10 225537 . T A 999 . . GT:PL:DP 0/1:203,0,255:47 0/1:215,0,255:67 0/1:255,0,255:29 0/1:125,0,107:10 0/1:198,0,125:14 0/1:94,0,139:12 0/1:255,0,255:175

10 225595 . A G 999 . . GT:PL:DP 0/1:164,0,255:37 0/1:255,0,255:68 0/1:255,0,255:46 0/1:166,0,104:11 0/1:46,0,141:9 0/1:101,0,87:13 0/1:255,0,255:149

10 286401 . A G 999 . . GT:PL:DP 0/1:205,10,0:18 0/1:32,0,52:14 0/1:144,0,173:48 0/1:81,0,133:13 0/1:29,0,87:12 0/1:16,0,30:4 0/1:222,0,73:43

10 319041 . A T 999 . . GT:PL:DP 0/1:47,0,255:18 0/1:100,0,193:15 0/1:255,0,183:26 0/1:55,0,81:6 0/1:55,0,30:3 0/1:22,0,157:9 0/1:196,0,187:19

10 415686 . G T 999 . . GT:PL:DP 0/1:202,0,143:28 0/1:218,0,78:26 0/1:142,0,192:22 0/1:42,0,8:3 0/1:87,0,23:8 0/1:42,0,47:5 0/1:236,0,30:30

10 415687 . C T 999 . . GT:PL:DP 0/1:209,0,135:27 0/1:223,0,78:26 0/1:142,0,182:22 0/1:49,0,5:3 0/1:87,0,23:8 0/1:42,0,47:5 0/1:237,0,35:30

10 421792 . A T 999 . . GT:PL:DP 0/1:82,0,40:7 0/1:82,0,39:6 0/1:70,0,10:6 0/1:69,9,0:3 0/1:67,0,16:4 0/1:33,3,0:1 0/1:119,0,255:40

10 486258 . T G 999 . . GT:PL:DP 0/1:187,0,90:21 0/1:255,0,190:42 0/1:153,0,123:22 0/1:76,5,0:6 0/1:83,0,181:17 0/1:99,0,133:17 0/1:255,0,218:105

10 486271 . A T 999 . . GT:PL:DP 0/1:195,0,87:21 0/1:255,0,138:41 0/1:167,0,108:22 0/1:68,3,0:5 0/1:125,0,189:22 0/1:110,0,153:16 0/1:255,0,220:110

10 486274 . T C 999 . . GT:PL:DP 0/1:189,0,91:20 0/1:217,0,202:41 0/1:197,0,105:23 0/1:54,0,21:6 0/1:124,0,192:22 0/1:99,0,181:17 0/1:255,0,229:112

10 541200 . G T 999 . . GT:PL:DP 0/1:77,0,229:22 0/1:99,0,215:26 0/1:16,0,197:14 0/1:40,9,0:3 0/1:65,0,15:8 0/1:26,0,109:7 0/1:145,0,187:33

10 644798 . C T 999 . . GT:PL:DP 0/1:181,0,163:48 0/1:236,0,252:57 0/1:255,0,200:74 0/1:120,0,60:8 0/1:110,0,219:19 0/1:193,0,212:51 0/1:252,0,239:76

10 703077 . T G 999 . . GT:PL:DP 0/1:118,0,241:27 0/1:189,0,254:27 0/1:140,0,190:20 0/1:74,6,0:2 0/1:56,0,43:4 0/1:18,0,70:4 0/1:174,0,255:29

10 951845 . T C 999 . . GT:PL:DP 0/1:255,0,255:45 0/1:254,0,255:36 0/1:255,0,127:30 0/1:107,0,105:9 0/1:84,9,0:3 0/1:127,0,19:6 0/1:226,0,255:35

10 1007361 . G C 999 . . GT:PL:DP 0/1:69,0,255:132 0/1:122,0,255:122 0/1:77,0,255:151 0/1:103,0,223:29 0/1:81,0,176:46 0/1:14,0,255:35 0/1:136,0,255:220

10 1007377 . T G 999 . . GT:PL:DP 0/1:109,0,255:149 0/1:198,0,255:140 0/1:158,0,255:164 0/1:119,0,222:33 0/1:107,0,184:47 0/1:67,0,255:30 0/1:166,0,255:232

10 1007394 . T A 999 . . GT:PL:DP 0/1:159,0,255:157 0/1:203,0,255:152 0/1:130,0,255:170 0/1:89,0,255:34 0/1:102,0,211:49 0/1:51,0,255:36 0/1:70,0,255:242

10 1007397 . G C 999 . . GT:PL:DP 0/1:163,0,255:157 0/1:197,0,255:157 0/1:122,0,255:170 0/1:80,0,255:33 0/1:99,0,240:51 0/1:49,0,255:37 0/1:54,0,255:245

10 1008862 . C T 999 . . GT:PL:DP 0/1:214,0,212:30 0/1:195,0,231:30 0/1:255,0,212:48 0/1:84,0,107:10 0/1:51,0,28:3 0/1:75,0,35:5 0/1:196,0,251:37

10 1065722 . A G 999 . . GT:PL:DP 0/1:17,0,241:31 0/1:30,0,214:24 0/1:12,0,222:22 0/1:10,0,105:7 0/1:92,0,113:16 0/1:27,0,179:22 0/1:46,0,215:71

10 1085113 . A G 999 . . GT:PL:DP 0/1:81,0,255:83 0/1:29,0,243:86 0/1:7,0,255:85 0/1:43,0,122:19 0/1:69,0,76:10 0/1:21,0,189:29 0/1:40,0,255:91

10 1085258 . A T 999 . . GT:PL:DP 0/1:55,0,255:94 0/1:63,0,255:83 0/1:41,0,255:117 0/1:114,0,208:21 0/1:74,0,100:11 0/1:8,0,236:18 0/1:147,0,255:188

10 1085323 . C T 999 . . GT:PL:DP 0/1:255,0,255:116 0/1:221,0,255:113 0/1:197,0,255:115 0/1:152,0,255:30 0/1:136,0,59:17 0/1:211,0,255:36 0/1:255,0,255:195

10 1102751 . A G 999 . . GT:PL:DP 0/1:80,9,0:3 0/1:172,0,5:12 0/1:28,3,0:1 0/1:68,9,0:3 0/1:34,3,0:1 0/1:34,3,0:1 0/1:149,1,0:12

10 1102752 . C G 999 . . GT:PL:DP 0/1:70,3,0:5 0/1:177,2,0:13 0/1:28,3,0:1 0/1:67,9,0:3 0/1:34,3,0:1 0/1:34,3,0:1 0/1:134,0,3:12

10 1329447 . G C 999 . . GT:PL:DP 0/1:127,0,236:31 0/1:84,0,241:29 0/1:148,0,181:26 0/1:0,9,84:3 0/1:24,0,70:4 0/1:69,0,27:7 0/1:180,0,215:33

10 1505279 . C T 999 . . GT:PL:DP 0/1:152,0,191:46 0/1:112,0,255:53 0/1:190,0,255:62 0/1:32,0,228:19 0/1:110,0,155:23 0/1:138,0,196:46 0/1:75,0,255:63

10 1505507 . C A 999 . . GT:PL:DP 0/1:135,0,207:99 0/1:79,0,235:100 0/1:139,0,231:116 0/1:61,0,125:12 0/1:23,0,67:11 0/1:63,0,69:23 0/1:255,0,255:100

10 1520971 . A T 999 . . GT:PL:DP 0/1:41,0,211:110 0/1:81,0,254:137 0/1:105,0,217:112 0/1:212,0,255:70 0/1:91,0,32:103 0/1:50,0,101:79 0/1:255,0,255:166

10 1521436 . T C 999 . . GT:PL:DP 0/1:245,0,255:177 0/1:255,0,255:171 0/1:255,0,255:157 0/1:130,0,237:25 0/1:173,0,192:20 0/1:195,0,86:25 0/1:255,0,255:184

10 1521440 . C G 999 . . GT:PL:DP 0/1:250,0,255:163 0/1:255,0,255:167 0/1:255,0,255:156 0/1:127,0,251:26 0/1:169,0,211:21 0/1:196,0,84:24 0/1:255,0,255:179

10 1534571 . T C 999 . . GT:PL:DP 0/1:255,0,255:57 0/1:255,0,175:50 0/1:110,0,255:41 0/1:27,0,29:4 0/1:140,6,0:7 0/1:81,0,112:14 0/1:31,0,255:54

10 1608722 . A C 999 . . GT:PL:DP 0/1:176,0,143:35 0/1:133,0,185:34 0/1:146,0,186:39 0/1:45,0,85:11 0/1:21,3,0:1 0/1:6,0,40:3 0/1:204,0,212:38

10 1624979 . A G 999 . . GT:PL:DP 0/1:159,0,151:28 0/1:255,0,172:37 0/1:147,0,111:17 0/1:125,0,76:13 0/1:127,0,95:20 0/1:203,0,124:42 0/1:227,0,173:63

10 1633158 . T C 999 . . GT:PL:DP 0/1:110,0,139:30 0/1:124,0,75:28 0/1:84,0,72:26 0/1:19,0,15:5 0/1:20,0,36:4 0/1:73,0,32:8 0/1:162,0,129:42

10 1674310 . T G 999 . . GT:PL:DP 0/1:92,0,108:83 0/1:68,0,232:57 0/1:29,0,176:108 0/1:12,0,24:7 0/1:20,0,105:12 0/1:7,0,125:8 0/1:65,0,242:102

10 1674641 . A T 999 . . GT:PL:DP 0/1:218,0,252:133 0/1:214,0,255:129 0/1:143,0,255:129 0/1:114,0,255:39 0/1:115,0,194:25 0/1:120,0,197:24 0/1:214,0,255:180

10 1674644 . G T 999 . . GT:PL:DP 0/1:189,37,136:134 0/1:201,0,246:134 0/1:239,71,255:126 0/1:113,0,255:40 0/1:136,0,113:28 0/1:144,26,124:23 0/1:233,29,255:180

10 1674867 . A G 999 . . GT:PL:DP 0/1:73,5,0:101 0/1:115,0,127:76 0/1:165,0,120:131 0/1:126,0,61:22 0/1:38,7,0:7 0/1:42,0,28:6 0/1:115,0,197:109

10 1937092 . A G 999 . . GT:PL:DP 0/1:255,0,255:44 0/1:255,0,173:37 0/1:255,0,255:43 0/1:51,0,153:10 0/1:109,0,19:5 0/1:83,9,0:3 0/1:255,0,187:43

10 2027946 . G T 999 . . GT:PL:DP 0/1:37,0,151:14 0/1:101,0,38:14 0/1:143,0,27:11 0/1:20,0,19:2 0/1:41,20,17:2 0/1:64,44,59:5 0/1:89,0,197:31

10 2027947 . C G 999 . . GT:PL:DP 0/1:25,0,133:12 0/1:101,0,38:14 0/1:141,0,27:11 0/1:20,0,19:2 0/1:41,20,17:2 0/1:64,44,59:5 0/1:78,0,197:28

10 2089613 . G T 999 . . GT:PL:DP 0/1:255,0,189:30 0/1:255,0,255:40 0/1:232,0,255:35 0/1:134,0,48:7 0/1:66,0,140:8 0/1:26,0,31:2 0/1:246,0,255:32

10 2089711 . G T 999 . . GT:PL:DP 0/1:244,0,48:35 0/1:197,0,211:35 0/1:250,0,209:32 0/1:81,0,54:5 0/1:78,0,17:4 0/1:31,3,0:1 0/1:254,0,181:35

10 2089725 . T C 999 . . GT:PL:DP 0/1:238,0,56:37 0/1:137,0,183:36 0/1:219,0,215:29 0/1:83,0,20:4 0/1:64,6,0:2 0/1:52,26,23:2 0/1:180,0,150:31

10 2090086 . C T 999 . . GT:PL:DP 0/1:255,0,255:45 0/1:255,0,255:61 0/1:255,0,255:39 0/1:32,0,120:7 0/1:146,0,80:9 0/1:57,0,29:3 0/1:255,0,255:65

10 2090100 . G T 999 . . GT:PL:DP 0/1:255,0,255:48 0/1:255,0,255:62 0/1:255,0,255:40 0/1:16,0,141:7 0/1:153,0,52:8 0/1:57,0,29:3 0/1:255,0,255:66

10 2090108 . A G 999 . . GT:PL:DP 0/1:237,0,255:49 0/1:255,0,255:57 0/1:255,0,255:37 0/1:121,0,22:5 0/1:46,0,184:10 0/1:29,0,57:3 0/1:255,0,255:66

10 2090360 . A G 999 . . GT:PL:DP 0/1:255,0,255:44 0/1:255,0,151:43 0/1:220,0,255:33 0/1:87,0,50:6 0/1:66,0,20:4 0/1:75,0,139:8 0/1:255,0,255:49

10 2199637 . A G 999 . . GT:PL:DP 0/1:102,0,1:15 0/1:98,0,235:29 0/1:135,0,80:20 0/1:51,6,0:2 0/1:13,0,54:3 0/1:13,0,29:2 0/1:131,0,196:34

10 2225330 . A T 999 . . GT:PL:DP 0/1:229,0,92:37 0/1:255,0,179:44 0/1:96,0,208:34 0/1:79,0,13:4 0/1:129,0,83:13 0/1:224,0,102:32 0/1:255,0,157:109

10 2225339 . T A 999 . . GT:PL:DP 0/1:244,0,109:42 0/1:255,0,180:46 0/1:118,0,192:35 0/1:61,0,24:3 0/1:78,0,89:12 0/1:133,0,134:34 0/1:255,0,186:110

10 2225854 . G T 999 . . GT:PL:DP 0/1:206,0,252:56 0/1:201,0,218:76 0/1:152,0,255:56 0/1:54,0,85:8 0/1:92,0,106:10 0/1:114,0,18:11 0/1:49,0,255:79

10 2226104 . A C 999 . . GT:PL:DP 0/1:165,0,255:87 0/1:80,0,248:130 0/1:53,0,255:80 0/1:9,0,239:19 0/1:71,0,228:18 0/1:36,0,169:11 0/1:154,0,246:159

10 2226190 . T C 999 . . GT:PL:DP 0/1:255,0,255:95 0/1:233,0,255:160 0/1:117,0,255:106 0/1:59,0,216:17 0/1:105,0,203:20 0/1:60,0,168:14 0/1:255,0,215:167

10 2283767 . G A 999 . . GT:PL:DP 0/1:149,0,255:74 0/1:68,0,255:49 0/1:94,0,255:34 0/1:10,0,204:9 0/1:95,0,111:8 0/1:35,0,182:12 0/1:127,0,255:70

10 2283769 . C A 999 . . GT:PL:DP 0/1:144,0,255:74 0/1:76,0,255:48 0/1:92,0,255:33 0/1:19,0,152:6 0/1:95,0,111:8 0/1:35,0,178:12 0/1:111,0,255:69

10 2292697 . C G 999 . . GT:PL:DP 0/1:255,0,131:63 0/1:201,0,255:63 0/1:255,0,103:56 0/1:123,0,16:7 0/1:26,0,66:5 0/1:53,0,58:6 0/1:255,0,183:54

10 2311572 . T C 999 . . GT:PL:DP 0/1:13,0,204:14 0/1:123,0,210:23 0/1:220,0,130:19 0/1:55,0,24:3 0/1:68,6,0:2 0/1:72,0,233:12 0/1:254,0,81:22

10 2313562 . G A 999 . . GT:PL:DP 0/1:118,6,0:6 0/1:146,0,144:23 0/1:161,0,128:29 0/1:115,0,71:8 0/1:37,3,0:1 0/1:30,0,60:3 0/1:169,0,213:33

10 2316244 . A C 999 . . GT:PL:DP 0/1:202,0,254:50 0/1:163,0,255:50 0/1:172,0,222:42 0/1:48,0,66:5 0/1:44,0,77:7 0/1:45,0,89:7 0/1:216,0,244:72

10 2316579 . C G 999 . . GT:PL:DP 0/1:119,0,235:23 0/1:184,0,232:32 0/1:157,0,155:26 0/1:50,0,79:5 0/1:61,0,45:4 0/1:57,0,24:4 0/1:13,0,255:39

10 2319857 . C T 999 . . GT:PL:DP 0/1:216,0,125:27 0/1:168,0,88:18 0/1:255,0,93:42 0/1:30,0,3:3 0/1:58,0,75:16 0/1:17,0,27:3 0/1:255,0,78:50

10 2320948 . T C 999 . . GT:PL:DP 0/1:221,0,255:146 0/1:165,0,255:97 0/1:134,0,255:141 0/1:9,0,230:48 0/1:14,0,50:10 0/1:88,0,167:60 0/1:59,0,243:146

10 2321098 . A T 999 . . GT:PL:DP 0/1:130,0,255:117 0/1:188,0,255:149 0/1:160,0,255:121 0/1:101,0,127:57 0/1:126,0,255:50 0/1:161,0,198:41 0/1:146,0,255:116

10 2321243 . A G 999 . . GT:PL:DP 0/1:144,0,214:65 0/1:170,0,148:44 0/1:177,0,193:66 0/1:37,0,40:5 0/1:73,0,149:14 0/1:82,0,20:9 0/1:207,0,218:95

10 2321281 . T C 999 . . GT:PL:DP 0/1:174,0,97:39 0/1:172,0,53:31 0/1:228,0,31:33 0/1:51,6,0:2 0/1:83,0,76:12 0/1:61,0,21:8 0/1:236,0,62:51

10 2327920 . C T 999 . . GT:PL:DP 0/1:97,0,77:11 0/1:72,0,170:28 0/1:101,0,108:24 0/1:16,0,45:3 0/1:55,6,0:2 0/1:18,0,46:6 0/1:90,0,121:13

10 2338573 . C T 999 . . GT:PL:DP 0/1:16,0,23:4 0/1:113,0,95:21 0/1:29,0,58:6 0/1:35,0,40:4 0/1:50,6,0:2 0/1:25,3,0:1 0/1:117,0,84:16

10 2373189 . T A 999 . . GT:PL:DP 0/1:205,0,255:243 0/1:191,0,255:244 0/1:235,0,255:238 0/1:203,0,255:49 0/1:222,0,193:127 0/1:180,0,255:187 0/1:198,0,255:281

10 2373260 . A T 999 . . GT:PL:DP 0/1:248,0,255:132 0/1:225,0,255:145 0/1:255,0,255:154 0/1:211,0,255:48 0/1:255,0,255:108 0/1:178,0,163:63 0/1:116,0,255:131

10 2433186 . A G 999 . . GT:PL:DP 0/1:103,0,95:11 0/1:144,0,54:12 0/1:120,0,72:11 0/1:82,6,0:2 0/1:22,0,32:6 0/1:67,0,24:18 0/1:205,0,161:31

10 2555654 . C T 999 . . GT:PL:DP 0/1:255,0,255:118 0/1:255,0,254:110 0/1:255,0,215:96 0/1:255,0,201:67 0/1:255,0,244:144 0/1:255,0,253:130 0/1:255,0,255:133

10 2593563 . C T 115.037 . . GT:PL:DP 0/1:35,0,113:31 0/1:22,0,131:31 0/1:20,0,68:18 0/1:36,3,0:1 0/1:23,0,33:4 0/1:7,0,59:6 0/1:78,0,155:58

10 2608234 . G C 999 . . GT:PL:DP 0/1:238,0,44:27 0/1:231,0,55:22 0/1:203,0,29:20 0/1:25,3,0:1 0/1:51,0,62:5 0/1:76,5,0:4 0/1:218,0,122:33

10 2617114 . C A 999 . . GT:PL:DP 0/1:151,0,255:35 0/1:211,0,234:31 0/1:181,0,208:30 0/1:81,0,78:7 0/1:27,0,139:9 0/1:25,0,30:3 0/1:159,0,255:36

10 2617163 . A G 999 . . GT:PL:DP 0/1:210,0,255:41 0/1:255,0,229:45 0/1:211,0,211:34 0/1:135,0,78:9 0/1:59,0,109:11 0/1:70,0,60:5 0/1:232,0,255:44

10 2617484 . A T 999 . . GT:PL:DP 0/1:139,0,255:46 0/1:115,0,160:31 0/1:160,0,250:30 0/1:56,0,17:4 0/1:52,8,2:3 0/1:52,0,104:7 0/1:204,0,239:44

10 2617523 . C A 999 . . GT:PL:DP 0/1:255,0,117:37 0/1:157,0,87:30 0/1:206,0,142:20 0/1:17,0,56:4 0/1:32,0,21:3 0/1:103,0,28:6 0/1:255,0,181:43

10 2791766 . C A 999 . . GT:PL:DP 0/1:77,0,248:24 0/1:99,0,255:26 0/1:50,0,255:25 0/1:23,3,0:1 0/1:29,23,20:2 0/1:34,3,0:1 0/1:52,0,255:32

10 2848109 . T A 999 . . GT:PL:DP 0/1:168,0,255:144 0/1:184,0,255:179 0/1:184,0,255:156 0/1:32,0,199:35 0/1:49,0,54:4 0/1:36,0,117:15 0/1:182,0,181:152

10 2848113 . T G 999 . . GT:PL:DP 0/1:165,0,255:166 0/1:221,0,255:196 0/1:196,0,255:188 0/1:71,0,198:35 0/1:41,0,54:5 0/1:37,0,115:16 0/1:189,0,145:170

10 2848505 . A C 999 . . GT:PL:DP 0/1:255,0,255:280 0/1:229,0,255:300 0/1:217,0,255:303 0/1:253,0,255:84 0/1:160,0,123:68 0/1:216,0,110:102 0/1:255,0,194:311

10 2848820 . G C 999 . . GT:PL:DP 0/1:1,0,163:59 0/1:97,0,249:69 0/1:20,0,149:74 0/1:14,0,107:10 0/1:58,0,97:10 0/1:117,0,148:16 0/1:51,0,254:99

10 2907456 . A T 999 . . GT:PL:DP 0/1:23,0,156:17 0/1:31,0,130:14 0/1:47,0,135:18 0/1:88,0,31:7 0/1:23,0,35:5 0/1:123,0,11:6 0/1:60,0,109:20

10 2953349 . A C 999 . . GT:PL:DP 0/1:255,0,137:257 0/1:255,0,215:256 0/1:255,0,255:258 0/1:255,0,232:139 0/1:255,0,255:64 0/1:255,0,255:68 0/1:169,0,76:263

10 2953418 . C T 999 . . GT:PL:DP 0/1:255,0,195:246 0/1:255,0,255:244 0/1:255,0,250:243 0/1:255,0,255:145 0/1:255,0,255:59 0/1:255,0,221:70 0/1:255,0,159:236

10 2953672 . T C 999 . . GT:PL:DP 0/1:233,0,163:238 0/1:255,0,255:236 0/1:255,0,255:231 0/1:245,0,181:144 0/1:255,0,255:138 0/1:255,0,255:83 0/1:255,0,255:233

10 2953700 . G T 999 . . GT:PL:DP 0/1:228,0,143:238 0/1:255,0,255:236 0/1:255,0,255:239 0/1:226,0,166:127 0/1:227,0,255:123 0/1:208,0,210:80 0/1:255,0,222:230

10 2953708 . C A 999 . . GT:PL:DP 0/1:79,0,233:238 0/1:134,0,235:222 0/1:201,0,255:221 0/1:46,0,224:125 0/1:205,0,255:137 0/1:159,0,255:94 0/1:68,0,204:224

10 2953718 . A G 999 . . GT:PL:DP 0/1:216,0,81:234 0/1:200,0,93:219 0/1:255,0,219:218 0/1:252,0,149:120 0/1:255,0,227:122 0/1:238,0,116:100 0/1:201,0,72:220

10 2953795 . A C 999 . . GT:PL:DP 0/1:255,0,159:213 0/1:255,0,184:200 0/1:255,0,229:206 0/1:254,0,115:72 0/1:255,0,37:155 0/1:255,0,59:103 0/1:251,0,110:210

10 2984091 . T A 999 . . GT:PL:DP 0/1:232,0,255:233 0/1:255,0,255:228 0/1:255,0,255:220 0/1:255,0,226:45 0/1:140,0,140:40 0/1:134,0,164:70 0/1:255,0,255:235

10 3037573 . A G 999 . . GT:PL:DP 0/1:255,0,125:40 0/1:213,0,244:33 0/1:16,0,255:38 0/1:138,0,196:17 0/1:15,0,144:8 0/1:28,0,27:3 0/1:227,0,255:83

10 3252353 . A G 999 . . GT:PL:DP 0/1:27,0,79:14 0/1:124,0,116:22 0/1:213,0,215:70 0/1:20,0,37:4 0/1:18,0,24:3 0/1:31,3,0:1 0/1:70,0,204:55

10 3419508 . T A 999 . . GT:PL:DP 0/1:122,0,71:24 0/1:144,0,117:41 0/1:151,4,0:26 0/1:54,0,83:10 0/1:52,0,33:5 0/1:8,0,33:3 0/1:144,0,39:30

10 3543743 . C T 999 . . GT:PL:DP 0/1:163,0,255:31 0/1:209,0,146:18 0/1:148,0,189:22 0/1:87,0,22:4 0/1:42,0,89:7 0/1:26,3,0:1 0/1:218,0,255:36

10 3547794 . A G 999 . . GT:PL:DP 0/1:144,0,68:12 0/1:93,0,22:5 0/1:90,9,0:3 0/1:83,9,0:3 0/1:96,9,0:3 0/1:103,9,0:3 0/1:119,0,162:18

10 3564160 . A G 999 . . GT:PL:DP 0/1:255,0,255:135 0/1:255,0,255:187 0/1:210,0,255:150 0/1:179,0,216:26 0/1:183,0,180:30 0/1:255,0,165:35 0/1:255,0,255:203

10 3624748 . G T 999 . . GT:PL:DP 0/1:103,0,255:33 0/1:174,0,133:21 0/1:151,0,123:27 0/1:55,0,41:6 0/1:40,0,40:5 0/1:23,0,53:4 0/1:182,0,205:38

10 3780292 . G T 999 . . GT:PL:DP 0/1:85,0,136:42 0/1:110,0,204:33 0/1:114,0,200:37 0/1:14,0,42:5 0/1:23,0,27:5 0/1:107,0,29:14 0/1:82,0,46:14

10 3780675 . T C 999 . . GT:PL:DP 0/1:50,0,185:24 0/1:26,0,136:23 0/1:57,0,66:25 0/1:46,0,19:3 0/1:23,0,54:5 0/1:27,3,0:3 0/1:17,0,62:13

10 3803889 . A G 999 . . GT:PL:DP 0/1:174,0,255:56 0/1:142,0,255:52 0/1:117,0,255:72 0/1:23,0,216:14 0/1:51,0,159:11 0/1:85,0,182:11 0/1:115,0,255:78

10 3912778 . A G 999 . . GT:PL:DP 0/1:251,0,255:39 0/1:226,0,254:42 0/1:233,0,255:45 0/1:78,0,47:5 0/1:26,0,43:4 0/1:83,0,20:4 0/1:215,0,255:47

10 4038920 . T C 999 . . GT:PL:DP 0/1:144,0,188:23 0/1:165,0,200:28 0/1:79,0,231:31 0/1:29,0,39:4 0/1:39,0,133:15 0/1:20,0,29:6 0/1:166,0,255:50

10 4115102 . A T 999 . . GT:PL:DP 0/1:217,0,255:33 0/1:220,0,196:29 0/1:255,0,215:33 0/1:110,0,44:7 0/1:61,0,28:5 0/1:23,0,31:4 0/1:218,0,255:85

10 4115252 . G A 999 . . GT:PL:DP 0/1:177,0,163:29 0/1:206,0,255:46 0/1:255,0,194:41 0/1:108,0,39:7 0/1:24,0,20:5 0/1:41,0,83:9 0/1:224,0,255:100

10 4127373 . G C 999 . . GT:PL:DP 0/1:109,0,202:34 0/1:142,0,178:37 0/1:170,0,137:32 0/1:66,0,82:9 0/1:18,3,0:1 0/1:58,9,0:3 0/1:196,0,95:41

10 4137602 . C G 999 . . GT:PL:DP 0/1:221,0,255:43 0/1:150,0,255:76 0/1:119,0,255:116 0/1:164,0,94:13 0/1:25,0,127:12 0/1:92,0,203:25 0/1:200,0,255:94

10 4137736 . C T 999 . . GT:PL:DP 0/1:255,0,197:43 0/1:255,0,255:96 0/1:255,0,255:146 0/1:243,0,90:16 0/1:83,0,106:9 0/1:166,0,255:29 0/1:223,0,255:100

10 4197708 . C T 999 . . GT:PL:DP 0/1:217,0,247:33 0/1:236,0,243:37 0/1:231,0,255:46 0/1:98,0,80:7 0/1:53,0,61:5 0/1:98,9,0:4 0/1:211,0,255:44

10 4241415 . T A 999 . . GT:PL:DP 0/1:76,0,122:14 0/1:159,0,125:20 0/1:65,0,184:27 0/1:40,0,55:4 0/1:58,6,0:2 0/1:19,0,47:3 0/1:117,0,225:36

10 4243557 . T A 999 . . GT:PL:DP 0/1:153,0,255:80 0/1:35,0,255:89 0/1:120,0,255:73 0/1:44,0,156:12 0/1:47,0,50:5 0/1:26,0,255:26 0/1:169,0,255:90

10 4243558 . A T 999 . . GT:PL:DP 0/1:156,0,255:80 0/1:54,0,255:89 0/1:116,0,255:73 0/1:86,0,161:12 0/1:18,0,61:4 0/1:30,0,255:26 0/1:162,0,255:91

10 4244233 . G A 999 . . GT:PL:DP 0/1:237,0,255:28 0/1:255,0,255:53 0/1:194,0,255:29 0/1:125,0,184:12 0/1:168,0,156:17 0/1:255,0,255:60 0/1:255,0,255:50

10 4244266 . T C 999 . . GT:PL:DP 0/1:102,0,255:28 0/1:244,0,255:37 0/1:120,0,255:21 0/1:88,0,137:11 0/1:154,0,129:14 0/1:255,0,255:56 0/1:255,0,255:49

10 4244292 . G A 999 . . GT:PL:DP 0/1:111,0,255:25 0/1:214,0,238:32 0/1:117,0,245:19 0/1:89,0,131:11 0/1:18,0,57:10 0/1:186,0,113:47 0/1:255,0,255:44

10 4290876 . C T 999 . . GT:PL:DP 0/1:72,0,26:9 0/1:79,0,31:14 0/1:136,0,11:16 0/1:35,0,9:3 0/1:10,4,0:5 0/1:9,3,0:1 0/1:212,0,63:29

10 4413160 . A T 999 . . GT:PL:DP 0/1:234,0,255:56 0/1:254,0,255:122 0/1:166,0,255:85 0/1:99,0,108:11 0/1:69,0,62:7 0/1:51,1,0:5 0/1:233,0,255:123

10 4447981 . C G 999 . . GT:PL:DP 0/1:215,0,221:44 0/1:117,0,255:40 0/1:219,0,137:28 0/1:55,0,19:3 0/1:122,0,76:14 0/1:40,6,0:2 0/1:187,0,225:50

10 4644039 . T C 999 . . GT:PL:DP 0/1:255,0,195:40 0/1:255,0,255:45 0/1:224,0,255:39 0/1:93,9,0:3 0/1:107,9,0:3 0/1:70,0,25:4 0/1:255,0,255:55

10 4790671 . G C 999 . . GT:PL:DP 0/1:61,0,122:42 0/1:51,0,178:61 0/1:31,0,174:42 0/1:59,0,11:12 0/1:15,0,59:9 0/1:37,0,78:14 0/1:91,0,142:65

10 4818110 . G A 999 . . GT:PL:DP 0/1:255,0,206:40 0/1:185,0,245:31 0/1:205,0,255:41 0/1:141,0,100:11 0/1:97,0,48:6 0/1:62,0,56:4 0/1:255,0,255:42

10 5038444 . G A 999 . . GT:PL:DP 0/1:255,0,255:165 0/1:202,0,255:152 0/1:255,0,255:162 0/1:132,0,255:32 0/1:31,0,211:43 0/1:137,0,230:113 0/1:185,0,255:170

10 5038463 . A T 999 . . GT:PL:DP 0/1:255,0,255:175 0/1:255,0,255:151 0/1:255,0,255:164 0/1:255,0,179:41 0/1:191,0,84:41 0/1:222,0,218:110 0/1:255,0,255:168

10 5149627 . C A 999 . . GT:PL:DP 0/1:232,0,255:50 0/1:255,0,255:43 0/1:189,0,203:18 0/1:150,0,13:7 0/1:78,0,124:9 0/1:24,0,103:5 0/1:255,0,255:37

10 5193116 . A G 999 . . GT:PL:DP 0/1:76,0,144:24 0/1:16,0,122:32 0/1:10,0,143:20 0/1:51,0,26:4 0/1:71,0,19:5 0/1:29,0,56:9 0/1:134,0,194:35

10 5230279 . C A 999 . . GT:PL:DP 0/1:255,0,255:141 0/1:255,0,255:159 0/1:255,0,255:159 0/1:255,0,255:86 0/1:150,0,209:102 0/1:7,0,206:130 0/1:224,0,255:173

10 5230345 . A C 999 . . GT:PL:DP 0/1:189,0,196:83 0/1:251,0,188:73 0/1:242,0,237:99 0/1:152,0,175:24 0/1:58,0,50:34 0/1:101,81,178:61 0/1:221,0,255:127

10 5230496 . C T 999 . . GT:PL:DP 0/1:255,0,128:126 0/1:246,0,208:162 0/1:219,0,232:190 0/1:247,0,141:35 0/1:126,0,23:36 0/1:106,0,53:22 0/1:255,0,211:209

10 5398401 . C T 999 . . GT:PL:DP 0/1:82,0,126:15 0/1:68,0,177:21 0/1:24,0,124:11 0/1:25,3,0:1 0/1:60,6,0:2 0/1:46,6,0:2 0/1:217,0,158:26

10 5451059 . C T 999 . . GT:PL:DP 0/1:161,0,147:131 0/1:128,0,43:100 0/1:161,0,116:101 0/1:95,0,64:16 0/1:92,0,24:22 0/1:18,0,20:27 0/1:154,0,130:84

10 5451072 . T A 999 . . GT:PL:DP 0/1:156,0,160:116 0/1:114,0,40:94 0/1:160,0,131:79 0/1:53,0,72:9 0/1:102,0,27:16 0/1:19,0,15:17 0/1:147,0,103:62

10 5466850 . C T 999 . . GT:PL:DP 0/1:252,0,128:60 0/1:162,0,255:77 0/1:248,0,255:94 0/1:147,0,185:20 0/1:18,0,178:23 0/1:40,0,155:31 0/1:96,0,255:200

10 5467044 . T C 999 . . GT:PL:DP 0/1:130,0,255:61 0/1:76,0,255:69 0/1:183,0,151:46 0/1:10,0,173:11 0/1:156,0,242:25 0/1:127,0,255:26 0/1:194,0,255:210

10 5508105 . A G 999 . . GT:PL:DP 0/1:255,0,173:26 0/1:159,0,255:27 0/1:179,0,211:19 0/1:25,0,95:4 0/1:66,0,53:5 0/1:82,0,80:6 0/1:255,0,255:49

10 5671878 . G A 999 . . GT:PL:DP 0/1:130,0,255:37 0/1:84,0,253:44 0/1:102,0,198:29 0/1:11,0,33:3 0/1:59,0,47:9 0/1:55,0,157:16 0/1:29,0,249:54

10 5710432 . T C 999 . . GT:PL:DP 0/1:66,0,167:38 0/1:16,0,166:28 0/1:61,0,207:62 0/1:6,0,38:4 0/1:48,0,72:13 0/1:19,0,59:6 0/1:155,0,177:66

10 5710545 . T A 999 . . GT:PL:DP 0/1:133,0,129:41 0/1:55,0,147:30 0/1:158,0,121:44 0/1:49,0,26:6 0/1:30,0,41:9 0/1:65,0,6:8 0/1:179,0,148:85

10 5710681 . C A 999 . . GT:PL:DP 0/1:198,0,118:35 0/1:224,0,134:39 0/1:252,0,154:50 0/1:87,0,63:7 0/1:71,0,88:9 0/1:35,0,76:9 0/1:255,0,128:71

10 5710704 . A G 999 . . GT:PL:DP 0/1:95,0,222:32 0/1:135,0,236:33 0/1:102,0,255:45 0/1:63,0,90:7 0/1:36,0,110:8 0/1:41,0,66:7 0/1:106,0,255:65

10 5710707 . T A 999 . . GT:PL:DP 0/1:179,0,121:29 0/1:174,0,145:32 0/1:249,0,116:45 0/1:50,0,63:7 0/1:78,0,43:6 0/1:46,0,60:6 0/1:255,0,109:62

10 5887102 . T A 999 . . GT:PL:DP 0/1:255,0,255:211 0/1:255,0,255:174 0/1:230,0,9:124 0/1:255,0,133:34 0/1:233,0,255:38 0/1:255,0,223:35 0/1:255,0,255:213

10 5887891 . C T 999 . . GT:PL:DP 0/1:244,0,255:139 0/1:111,0,255:82 0/1:151,0,255:47 0/1:109,0,78:9 0/1:9,0,255:20 0/1:130,0,255:20 0/1:255,0,255:142

10 5922061 . G T 999 . . GT:PL:DP 0/1:130,0,26:21 0/1:145,0,12:24 0/1:142,7,0:20 0/1:22,3,0:1 0/1:13,0,23:3 0/1:56,0,29:5 0/1:96,0,87:23

10 5999901 . G A 999 . . GT:PL:DP 0/1:255,0,255:41 0/1:255,0,255:38 0/1:255,0,255:34 0/1:54,0,103:6 0/1:91,0,48:5 0/1:96,0,108:8 0/1:255,0,255:45

10 6182077 . G T 999 . . GT:PL:DP 0/1:150,0,255:192 0/1:208,0,255:175 0/1:73,0,255:189 0/1:139,0,255:142 0/1:61,0,255:176 0/1:81,0,244:206 0/1:224,0,255:233

10 6256665 . A T 999 . . GT:PL:DP 0/1:83,0,208:163 0/1:145,0,206:184 0/1:139,0,209:185 0/1:166,0,211:59 0/1:100,0,204:37 0/1:103,0,255:72 0/1:64,0,192:189

10 6403724 . T C 999 . . GT:PL:DP 0/1:70,0,81:15 0/1:63,0,105:13 0/1:74,0,102:16 0/1:15,0,17:2 0/1:18,0,13:2 0/1:9,0,42:5 0/1:127,0,66:23

10 6403727 . G A 999 . . GT:PL:DP 0/1:33,0,28:14 0/1:56,0,15:14 0/1:93,21,31:18 0/1:15,0,2:2 0/1:18,0,0:2 0/1:9,0,18:5 0/1:120,8,0:21

10 6403728 . C G 999 . . GT:PL:DP 0/1:37,0,28:14 0/1:58,0,16:13 0/1:107,0,10:18 0/1:15,0,2:2 0/1:18,0,0:2 0/1:9,0,18:5 0/1:98,5,0:22

10 6450397 . G A 999 . . GT:PL:DP 0/1:237,0,217:28 0/1:183,0,156:24 0/1:60,0,248:21 0/1:62,6,0:2 0/1:87,0,79:37 0/1:160,0,255:87 0/1:223,0,223:50

10 6489113 . G C 999 . . GT:PL:DP 0/1:122,0,67:11 0/1:61,0,176:22 0/1:117,0,95:17 0/1:35,0,28:2 0/1:37,0,107:12 0/1:168,0,71:27 0/1:61,0,94:26

10 6498631 . G A 999 . . GT:PL:DP 0/1:218,0,255:227 0/1:255,0,255:208 0/1:237,0,255:223 0/1:128,0,242:59 0/1:255,0,255:94 0/1:255,0,255:119 0/1:255,0,255:215

10 6521184 . T A 999 . . GT:PL:DP 0/1:253,0,243:232 0/1:196,0,83:238 0/1:255,0,224:248 0/1:228,0,136:51 0/1:39,0,14:15 0/1:44,0,42:26 0/1:137,0,100:265

10 6550185 . A C 999 . . GT:PL:DP 0/1:88,0,250:202 0/1:169,0,255:205 0/1:61,0,227:210 0/1:198,0,255:95 0/1:62,0,198:42 0/1:82,0,255:57 0/1:132,0,255:227

10 6550190 . T C 999 . . GT:PL:DP 0/1:71,0,250:206 0/1:164,0,255:208 0/1:51,0,213:212 0/1:207,0,255:97 0/1:92,0,204:48 0/1:76,0,255:68 0/1:145,0,255:226

10 6634047 . A T 999 . . GT:PL:DP 0/1:254,0,3:32 0/1:133,0,71:18 0/1:255,0,4:40 0/1:15,1,0:2 0/1:104,0,43:9 0/1:34,0,107:21 0/1:255,0,11:61

10 6662876 . T A 999 . . GT:PL:DP 0/1:158,0,255:81 0/1:133,0,255:46 0/1:195,0,255:99 0/1:91,0,107:8 0/1:45,0,190:12 0/1:108,0,102:15 0/1:223,0,255:116

10 6715241 . C T 999 . . GT:PL:DP 0/1:132,0,160:98 0/1:47,0,123:120 0/1:116,0,148:130 0/1:91,0,153:28 0/1:43,0,67:9 0/1:27,0,52:7 0/1:177,0,206:137

10 6716000 . C G 999 . . GT:PL:DP 0/1:255,0,255:254 0/1:255,0,255:268 0/1:255,0,255:278 0/1:255,0,255:73 0/1:255,0,255:103 0/1:255,0,255:159 0/1:255,0,255:246

10 6716032 . T A 999 . . GT:PL:DP 0/1:255,0,255:225 0/1:255,0,255:223 0/1:255,0,255:236 0/1:255,0,255:79 0/1:255,0,255:93 0/1:255,0,255:136 0/1:255,0,255:241

10 6759454 . T C 999 . . GT:PL:DP 0/1:220,0,215:31 0/1:198,0,255:42 0/1:246,0,255:50 0/1:120,0,16:10 0/1:18,0,14:4 0/1:13,0,29:2 0/1:205,0,233:40

10 6759521 . A T 999 . . GT:PL:DP 0/1:219,0,250:30 0/1:255,0,255:55 0/1:247,0,255:58 0/1:39,0,81:7 0/1:89,0,22:5 0/1:35,3,0:1 0/1:236,0,255:40

10 6759873 . G A 999 . . GT:PL:DP 0/1:234,0,255:36 0/1:255,0,254:38 0/1:255,0,210:42 0/1:43,0,128:8 0/1:92,0,73:7 0/1:35,3,0:1 0/1:206,0,255:47

10 6760389 . C T 999 . . GT:PL:DP 0/1:255,0,61:24 0/1:233,0,187:29 0/1:255,0,227:31 0/1:93,0,17:7 0/1:89,0,38:8 0/1:38,0,88:6 0/1:255,0,147:33

10 6836352 . A G 999 . . GT:PL:DP 0/1:132,0,255:26 0/1:83,0,187:25 0/1:30,0,175:18 0/1:38,0,93:7 0/1:12,0,105:8 0/1:56,0,36:4 0/1:157,0,215:32

10 6863357 . A G 999 . . GT:PL:DP 0/1:255,0,255:202 0/1:255,0,255:211 0/1:255,0,255:206 0/1:255,0,249:98 0/1:217,0,255:83 0/1:96,0,255:199 0/1:255,0,255:187

10 6863447 . A G 999 . . GT:PL:DP 0/1:255,0,255:198 0/1:255,0,255:211 0/1:255,0,255:201 0/1:255,0,255:85 0/1:64,0,255:83 0/1:204,0,255:185 0/1:225,0,255:182

10 6884524 . T A 999 . . GT:PL:DP 0/1:232,0,5:19 0/1:220,0,16:15 0/1:221,0,11:16 0/1:82,5,0:4 0/1:26,3,0:1 0/1:17,0,47:3 0/1:234,0,57:24

10 6887623 . C A 999 . . GT:PL:DP 0/1:93,0,200:32 0/1:91,0,174:25 0/1:95,0,163:19 0/1:26,0,43:7 0/1:19,0,16:2 0/1:47,0,71:9 0/1:59,0,187:25

10 6887645 . C T 999 . . GT:PL:DP 0/1:255,0,31:41 0/1:133,0,185:29 0/1:139,0,135:23 0/1:45,0,14:5 0/1:19,0,16:2 0/1:68,0,8:6 0/1:131,0,199:29

10 6942065 . T C 999 . . GT:PL:DP 0/1:111,0,255:35 0/1:42,0,229:22 0/1:84,0,255:18 0/1:13,0,134:8 0/1:106,0,148:11 0/1:59,0,199:13 0/1:163,0,255:54

10 6942083 . G C 999 . . GT:PL:DP 0/1:103,0,255:37 0/1:81,0,246:28 0/1:49,0,249:30 0/1:10,0,161:9 0/1:124,0,147:13 0/1:49,0,201:15 0/1:149,0,255:67

10 7042962 . A G 999 . . GT:PL:DP 0/1:51,0,255:43 0/1:151,0,255:88 0/1:84,0,255:46 0/1:70,0,117:11 0/1:30,0,217:15 0/1:80,0,216:21 0/1:247,0,255:160

10 7042980 . A G 999 . . GT:PL:DP 0/1:254,0,23:37 0/1:255,0,49:80 0/1:255,0,80:50 0/1:120,0,54:11 0/1:165,0,39:12 0/1:214,0,64:24 0/1:255,0,209:154

10 7043004 . A G 999 . . GT:PL:DP 0/1:39,0,220:31 0/1:9,0,240:82 0/1:65,0,254:53 0/1:58,0,91:9 0/1:47,0,109:9 0/1:59,0,255:26 0/1:181,0,255:135

10 7059859 . G A 999 . . GT:PL:DP 0/1:192,0,255:42 0/1:253,0,231:43 0/1:255,0,187:33 0/1:145,0,91:14 0/1:26,0,47:4 0/1:27,0,84:4 0/1:255,0,75:24

10 7123435 . T A 999 . . GT:PL:DP 0/1:35,0,117:21 0/1:59,0,156:27 0/1:46,0,105:25 0/1:14,0,95:6 0/1:9,0,22:10 0/1:43,0,13:7 0/1:104,0,154:54

10 7184623 . G A 999 . . GT:PL:DP 0/1:255,0,255:204 0/1:255,0,255:221 0/1:255,0,255:227 0/1:255,0,249:39 0/1:255,0,255:49 0/1:255,0,255:83 0/1:255,0,255:228

10 7185024 . G A 999 . . GT:PL:DP 0/1:83,0,157:68 0/1:255,0,134:84 0/1:147,0,182:93 0/1:52,0,117:12 0/1:71,0,59:10 0/1:30,0,201:34 0/1:228,0,255:131

10 7200438 . G A 999 . . GT:PL:DP 0/1:110,0,14:12 0/1:126,0,56:13 0/1:98,0,1:10 0/1:63,9,0:3 0/1:32,3,0:1 0/1:30,6,0:2 0/1:199,0,26:18

10 7200439 . A T 999 . . GT:PL:DP 0/1:86,0,7:9 0/1:122,0,36:13 0/1:98,0,1:10 0/1:63,9,0:3 0/1:32,3,0:1 0/1:30,6,0:2 0/1:194,3,0:18

10 7200441 . G T 999 . . GT:PL:DP 0/1:85,0,11:10 0/1:110,0,46:12 0/1:98,0,1:10 0/1:63,9,0:3 0/1:32,3,0:1 0/1:30,6,0:2 0/1:187,0,16:17

10 7203572 . A T 999 . . GT:PL:DP 0/1:235,0,255:46 0/1:255,0,241:30 0/1:255,0,227:49 0/1:70,0,59:5 0/1:197,4,0:10 0/1:46,0,129:7 0/1:255,0,255:40

10 7238357 . C T 999 . . GT:PL:DP 0/1:255,0,255:164 0/1:255,0,255:192 0/1:255,0,255:182 0/1:193,0,172:23 0/1:181,0,204:19 0/1:151,0,230:21 0/1:255,0,255:174

10 7238435 . G A 999 . . GT:PL:DP 0/1:255,0,255:82 0/1:255,0,227:100 0/1:255,0,255:77 0/1:133,0,182:19 0/1:68,0,20:10 0/1:98,0,10:9 0/1:255,0,255:92

10 7238562 . C T 999 . . GT:PL:DP 0/1:198,0,255:130 0/1:43,0,214:206 0/1:196,0,255:166 0/1:255,0,162:44 0/1:42,0,93:11 0/1:86,0,21:11 0/1:221,0,255:195

10 7238792 . A G 999 . . GT:PL:DP 0/1:255,0,255:173 0/1:255,0,255:199 0/1:255,0,255:180 0/1:201,0,255:55 0/1:231,0,255:97 0/1:243,0,255:59 0/1:175,0,249:183

10 7407566 . T G 999 . . GT:PL:DP 0/1:255,0,27:34 0/1:202,0,7:17 0/1:247,0,81:22 0/1:54,6,0:2 0/1:81,9,0:3 0/1:76,0,21:4 0/1:250,4,0:27

10 7470654 . A C 999 . . GT:PL:DP 0/1:148,0,255:157 0/1:108,0,255:145 0/1:214,0,255:152 0/1:27,0,127:25 0/1:65,0,139:36 0/1:64,0,255:115 0/1:255,0,255:208

10 7470655 . T A 999 . . GT:PL:DP 0/1:151,0,255:157 0/1:108,0,255:145 0/1:217,0,255:150 0/1:27,0,119:25 0/1:65,0,131:36 0/1:97,0,255:115 0/1:255,0,255:208

10 7486368 . T C 999 . . GT:PL:DP 0/1:255,0,207:27 0/1:255,0,255:37 0/1:255,0,255:41 0/1:82,9,0:3 0/1:62,6,0:2 0/1:72,9,0:3 0/1:255,0,242:37

10 7525385 . G T 999 . . GT:PL:DP 0/1:207,0,163:25 0/1:179,0,93:21 0/1:122,0,188:17 0/1:90,0,27:5 0/1:58,0,0:3 0/1:78,0,48:6 0/1:169,0,255:38

10 7559507 . A C 999 . . GT:PL:DP 0/1:112,0,149:23 0/1:90,0,210:26 0/1:125,0,87:20 0/1:22,0,62:3 0/1:67,0,85:9 0/1:13,0,111:7 0/1:237,0,132:32

10 7577402 . A T 999 . . GT:PL:DP 0/1:131,0,119:36 0/1:145,0,58:34 0/1:86,0,130:27 0/1:54,0,50:19 0/1:34,0,21:16 0/1:50,0,21:17 0/1:111,0,63:105

10 7594136 . G T 999 . . GT:PL:DP 0/1:28,3,0:1 0/1:82,9,0:3 0/1:77,9,0:3 0/1:25,3,0:1 0/1:26,3,0:1 0/1:26,3,0:1 0/1:53,6,0:2

10 7714713 . A G 999 . . GT:PL:DP 0/1:152,0,204:26 0/1:55,0,141:19 0/1:119,0,158:18 0/1:38,0,33:4 0/1:119,12,0:4 0/1:21,0,35:5 0/1:101,0,187:22

10 7715308 . A G 999 . . GT:PL:DP 0/1:153,0,204:33 0/1:117,0,208:33 0/1:100,0,140:21 0/1:50,6,0:2 0/1:40,6,0:2 0/1:14,0,81:4 0/1:162,0,198:52

10 7743671 . T C 999 . . GT:PL:DP 0/1:247,0,255:195 0/1:209,0,241:168 0/1:231,0,255:197 0/1:115,0,201:52 0/1:9,0,28:10 0/1:158,0,106:58 0/1:171,0,255:160

10 7743681 . G A 999 . . GT:PL:DP 0/1:255,0,255:181 0/1:240,0,232:156 0/1:255,0,255:188 0/1:103,0,225:51 0/1:42,1,0:9 0/1:140,0,82:55 0/1:235,0,255:135

10 7855126 . T A 999 . . GT:PL:DP 0/1:145,0,78:55 0/1:77,0,237:63 0/1:183,0,197:76 0/1:34,0,66:15 0/1:39,0,26:5 0/1:24,0,21:11 0/1:162,0,190:90

10 7855146 . A C 999 . . GT:PL:DP 0/1:147,0,27:46 0/1:81,0,250:59 0/1:167,0,172:69 0/1:41,0,65:13 0/1:28,0,26:5 0/1:26,0,26:6 0/1:179,0,129:80

10 7855167 . T A 999 . . GT:PL:DP 0/1:125,0,15:41 0/1:22,0,197:53 0/1:78,0,128:60 0/1:7,0,93:15 0/1:37,0,21:7 0/1:20,0,22:9 0/1:84,0,222:76

10 7900989 . C T 999 . . GT:PL:DP 0/1:180,0,214:68 0/1:237,0,22:88 0/1:155,0,154:40 0/1:75,4,0:9 0/1:100,0,29:8 0/1:44,0,28:8 0/1:179,0,193:128

10 7987131 . G T 999 . . GT:PL:DP 0/1:209,0,92:24 0/1:147,0,244:41 0/1:33,0,215:29 0/1:20,0,21:2 0/1:14,0,105:6 0/1:21,0,52:4 0/1:205,0,125:32

10 8009338 . G A 999 . . GT:PL:DP 0/1:83,0,43:14 0/1:87,0,70:24 0/1:54,0,131:26 0/1:19,0,6:2 0/1:49,9,0:3 0/1:14,0,11:2 0/1:38,0,79:18

10 8065205 . A G 999 . . GT:PL:DP 0/1:218,0,255:36 0/1:183,0,255:37 0/1:255,0,206:46 0/1:84,0,25:4 0/1:56,6,0:2 0/1:12,0,92:6 0/1:221,0,255:44

10 8075983 . C T 999 . . GT:PL:DP 0/1:242,0,255:45 0/1:201,0,177:27 0/1:82,0,231:22 0/1:63,0,98:8 0/1:72,9,0:3 0/1:99,9,0:3 0/1:255,0,255:62

10 8134643 . T G 999 . . GT:PL:DP 0/1:218,0,236:30 0/1:174,0,255:28 0/1:199,0,247:28 0/1:11,0,140:6 0/1:67,0,46:5 0/1:56,6,0:2 0/1:255,0,255:58

10 8183959 . G C 999 . . GT:PL:DP 0/1:255,0,255:144 0/1:255,0,255:82 0/1:255,0,255:76 0/1:216,0,178:30 0/1:88,9,0:3 0/1:99,0,248:22 0/1:143,0,243:211

10 8184053 . G T 999 . . GT:PL:DP 0/1:255,0,255:148 0/1:190,0,255:96 0/1:255,0,255:74 0/1:227,0,98:23 0/1:22,0,33:2 0/1:160,0,118:18 0/1:255,0,237:200

10 8184054 . T C 999 . . GT:PL:DP 0/1:255,0,255:147 0/1:172,0,255:97 0/1:255,0,255:73 0/1:219,0,153:25 0/1:19,0,31:2 0/1:159,0,114:19 0/1:255,0,255:197

10 8184107 . A T 999 . . GT:PL:DP 0/1:255,0,255:107 0/1:167,0,236:74 0/1:248,0,255:59 0/1:162,0,125:19 0/1:45,0,48:6 0/1:168,0,16:16 0/1:255,0,154:173

10 8184507 . C T 999 . . GT:PL:DP 0/1:255,0,255:213 0/1:255,0,255:154 0/1:255,0,255:175 0/1:255,0,255:58 0/1:255,0,255:82 0/1:255,0,190:114 0/1:255,0,177:227

10 8184610 . A G 999 . . GT:PL:DP 0/1:255,0,255:172 0/1:255,0,255:161 0/1:255,0,255:172 0/1:255,0,250:57 0/1:255,0,255:110 0/1:255,0,25:106 0/1:255,0,216:144

10 8184620 . T C 999 . . GT:PL:DP 0/1:255,0,255:150 0/1:255,0,255:144 0/1:255,0,255:155 0/1:255,0,240:54 0/1:255,0,255:110 0/1:255,0,24:105 0/1:255,0,225:145

10 8184623 . G A 999 . . GT:PL:DP 0/1:255,0,255:148 0/1:255,0,255:138 0/1:255,0,245:147 0/1:255,0,239:54 0/1:255,0,248:109 0/1:255,0,21:104 0/1:255,0,226:146

10 8184688 . C T 999 . . GT:PL:DP 0/1:255,0,255:72 0/1:184,0,255:40 0/1:255,0,223:59 0/1:224,7,0:18 0/1:21,3,0:14 0/1:58,0,6:22 0/1:255,0,221:120

10 8184761 . A C 999 . . GT:PL:DP 0/1:240,0,255:74 0/1:165,0,184:33 0/1:221,0,77:30 0/1:65,0,93:12 0/1:198,0,132:34 0/1:161,0,55:14 0/1:251,0,255:121

10 8184874 . T G 999 . . GT:PL:DP 0/1:255,0,255:170 0/1:255,0,255:86 0/1:255,0,189:105 0/1:171,0,152:28 0/1:112,0,128:39 0/1:126,0,56:26 0/1:239,0,204:210

10 8184906 . A C 999 . . GT:PL:DP 0/1:255,0,255:170 0/1:250,0,255:96 0/1:255,0,191:112 0/1:136,0,131:20 0/1:120,0,90:37 0/1:82,0,2:19 0/1:215,0,194:204

10 8184985 . T C 999 . . GT:PL:DP 0/1:255,0,255:189 0/1:255,0,255:94 0/1:255,0,178:125 0/1:187,0,140:17 0/1:255,0,255:68 0/1:255,0,54:81 0/1:255,0,211:206

10 8426609 . C T 999 . . GT:PL:DP 0/1:143,0,86:16 0/1:191,0,223:35 0/1:181,0,156:21 0/1:61,6,0:2 0/1:22,0,49:3 0/1:56,3,0:3 0/1:210,0,194:33

10 8600716 . T G 999 . . GT:PL:DP 0/1:74,0,66:15 0/1:75,0,58:16 0/1:25,0,68:13 0/1:47,12,0:4 0/1:17,3,0:1 0/1:27,12,0:4 0/1:92,0,255:43

10 8600994 . T C 999 . . GT:PL:DP 0/1:132,0,174:103 0/1:109,0,210:102 0/1:116,0,148:96 0/1:117,0,218:128 0/1:35,0,149:48 0/1:56,0,201:59 0/1:96,0,238:124

10 8601003 . C G 999 . . GT:PL:DP 0/1:135,0,161:94 0/1:157,0,194:94 0/1:123,0,131:87 0/1:126,0,230:111 0/1:40,0,109:41 0/1:61,0,141:54 0/1:116,0,239:118

10 8601318 . T C 999 . . GT:PL:DP 0/1:71,0,83:44 0/1:85,0,11:23 0/1:121,1,0:42 0/1:17,0,36:7 0/1:31,6,0:2 0/1:28,6,0:3 0/1:170,0,161:87

10 8703254 . G A 999 . . GT:PL:DP 0/1:217,0,255:24 0/1:255,0,198:29 0/1:224,0,246:31 0/1:81,0,111:8 0/1:49,0,154:9 0/1:160,0,78:11 0/1:255,0,255:66

10 8775106 . G A 999 . . GT:PL:DP 0/1:255,0,255:203 0/1:21,0,198:205 0/1:95,0,255:198 0/1:102,0,255:45 0/1:49,0,248:33 0/1:9,0,206:53 0/1:40,0,238:209

10 8776530 . C A 999 . . GT:PL:DP 0/1:252,0,255:157 0/1:232,0,255:112 0/1:255,0,255:176 0/1:193,0,219:45 0/1:134,0,72:14 0/1:124,0,100:22 0/1:242,0,255:215

10 8776583 . C T 999 . . GT:PL:DP 0/1:255,0,255:182 0/1:255,0,255:138 0/1:255,0,255:198 0/1:239,0,243:52 0/1:202,0,181:30 0/1:221,0,170:47 0/1:255,0,255:235

10 8805609 . G A 999 . . GT:PL:DP 0/1:255,0,89:39 0/1:72,0,97:14 0/1:255,0,127:34 0/1:98,0,36:9 0/1:40,0,100:9 0/1:22,0,43:7 0/1:151,0,151:33

10 8805733 . T C 999 . . GT:PL:DP 0/1:233,0,213:62 0/1:219,0,33:51 0/1:189,0,224:55 0/1:31,0,118:7 0/1:139,0,105:21 0/1:214,0,24:19 0/1:252,0,151:56

10 8805758 . T A 999 . . GT:PL:DP 0/1:245,0,214:52 0/1:238,0,67:52 0/1:186,0,225:48 0/1:21,0,108:7 0/1:118,0,38:11 0/1:144,0,4:17 0/1:255,0,153:51

10 8806464 . G T 999 . . GT:PL:DP 0/1:255,0,255:119 0/1:185,0,255:76 0/1:255,0,255:148 0/1:249,0,146:26 0/1:53,0,105:15 0/1:24,0,213:22 0/1:255,0,255:93

10 8806542 . G A 999 . . GT:PL:DP 0/1:133,0,255:82 0/1:245,0,255:45 0/1:132,0,255:96 0/1:35,0,255:19 0/1:66,0,110:10 0/1:131,0,143:18 0/1:193,0,255:58

10 8806545 . C T 999 . . GT:PL:DP 0/1:119,0,255:82 0/1:228,0,255:45 0/1:118,0,255:95 0/1:24,0,255:22 0/1:47,0,113:9 0/1:109,0,161:15 0/1:185,0,255:58

10 8852755 . C G 999 . . GT:PL:DP 0/1:59,0,123:12 0/1:138,0,126:19 0/1:176,0,146:22 0/1:6,0,60:3 0/1:64,6,0:2 0/1:44,0,83:5 0/1:219,0,143:28

10 9184921 . C T 999 . . GT:PL:DP 0/1:117,0,255:61 0/1:78,0,255:54 0/1:188,0,255:52 0/1:43,0,155:10 0/1:65,0,126:9 0/1:49,0,202:14 0/1:103,0,255:68

10 9184993 . G A 999 . . GT:PL:DP 0/1:122,0,255:64 0/1:211,0,255:58 0/1:120,0,255:64 0/1:26,0,179:12 0/1:67,0,49:5 0/1:134,0,132:15 0/1:161,0,255:84

10 9185064 . G T 999 . . GT:PL:DP 0/1:89,0,255:58 0/1:192,0,255:51 0/1:55,0,255:45 0/1:17,0,134:9 0/1:6,0,102:6 0/1:100,0,156:16 0/1:78,0,255:63

10 9360930 . C G 999 . . GT:PL:DP 0/1:172,0,220:73 0/1:155,0,147:46 0/1:103,0,225:58 0/1:3,0,117:10 0/1:59,6,0:24 0/1:21,0,50:4 0/1:203,0,239:130

10 9361017 . T G 999 . . GT:PL:DP 0/1:182,0,232:57 0/1:185,0,203:55 0/1:160,0,181:58 0/1:71,0,87:12 0/1:20,0,42:3 0/1:43,6,0:2 0/1:206,0,212:102

10 9361023 . C A 999 . . GT:PL:DP 0/1:131,0,238:53 0/1:178,0,235:52 0/1:164,0,192:58 0/1:77,0,79:12 0/1:23,0,29:2 0/1:3,0,51:3 0/1:170,0,215:103

10 9376558 . G A 999 . . GT:PL:DP 0/1:255,0,166:26 0/1:164,0,255:29 0/1:194,0,255:28 0/1:78,0,50:5 0/1:97,9,0:3 0/1:23,0,23:2 0/1:255,0,254:51

10 9608969 . T G 999 . . GT:PL:DP 0/1:253,0,179:78 0/1:255,0,228:67 0/1:255,0,87:86 0/1:180,0,9:12 0/1:153,0,107:34 0/1:158,0,114:61 0/1:255,0,255:206

10 9649049 . G A 999 . . GT:PL:DP 0/1:70,0,161:18 0/1:76,0,141:23 0/1:118,0,220:25 0/1:16,0,47:3 0/1:47,0,24:3 0/1:32,3,0:1 0/1:85,0,146:20

10 9683091 . G A 999 . . GT:PL:DP 0/1:108,0,177:39 0/1:112,0,201:35 0/1:96,0,147:21 0/1:93,0,88:11 0/1:11,0,59:7 0/1:15,0,76:11 0/1:149,0,154:39

10 9683298 . A T 999 . . GT:PL:DP 0/1:235,0,49:42 0/1:186,0,11:19 0/1:170,0,55:26 0/1:113,0,31:12 0/1:38,0,116:14 0/1:31,0,42:5 0/1:174,0,112:58

10 9683356 . A G 999 . . GT:PL:DP 0/1:151,0,189:49 0/1:79,0,97:33 0/1:85,0,136:42 0/1:13,0,76:14 0/1:58,0,57:19 0/1:35,0,20:14 0/1:80,0,133:80

10 9683441 . C A 999 . . GT:PL:DP 0/1:209,0,198:44 0/1:161,0,217:33 0/1:131,0,228:38 0/1:85,0,65:11 0/1:152,0,117:19 0/1:79,0,144:12 0/1:241,0,158:90

10 9683665 . G A 999 . . GT:PL:DP 0/1:111,0,171:59 0/1:85,0,194:48 0/1:104,0,170:60 0/1:141,0,111:22 0/1:85,0,14:14 0/1:80,0,2:21 0/1:243,0,251:111

10 9683775 . C T 999 . . GT:PL:DP 0/1:179,0,255:83 0/1:91,0,222:63 0/1:148,0,239:74 0/1:139,0,161:22 0/1:48,0,105:12 0/1:127,0,38:16 0/1:25,0,209:135

10 9683937 . C G 999 . . GT:PL:DP 0/1:211,0,69:58 0/1:133,0,52:34 0/1:193,0,41:43 0/1:130,0,18:12 0/1:35,5,0:6 0/1:51,8,0:15 0/1:167,0,181:72

10 9686729 . A T 999 . . GT:PL:DP 0/1:146,0,193:42 0/1:88,0,205:33 0/1:117,0,247:43 0/1:89,0,39:7 0/1:19,0,57:4 0/1:37,0,54:4 0/1:194,0,255:74

10 9686730 . C A 999 . . GT:PL:DP 0/1:157,0,176:42 0/1:86,0,194:33 0/1:127,0,242:41 0/1:111,0,29:7 0/1:17,0,60:4 0/1:19,0,54:4 0/1:195,0,255:74

10 9727113 . A T 999 . . GT:PL:DP 0/1:221,0,255:224 0/1:191,0,255:231 0/1:255,0,255:239 0/1:255,0,255:89 0/1:245,0,255:64 0/1:85,0,255:59 0/1:34,0,255:221

10 9727152 . T C 999 . . GT:PL:DP 0/1:149,0,255:223 0/1:112,0,245:233 0/1:180,0,255:242 0/1:179,0,255:72 0/1:55,0,255:57 0/1:255,0,255:54 0/1:210,0,255:227

10 9811471 . G A 999 . . GT:PL:DP 0/1:170,0,198:96 0/1:169,0,129:96 0/1:149,0,191:83 0/1:64,0,132:21 0/1:60,5,0:8 0/1:55,0,36:28 0/1:182,0,255:161

10 9811520 . G T 999 . . GT:PL:DP 0/1:203,0,244:116 0/1:148,0,120:101 0/1:166,0,255:105 0/1:105,0,168:27 0/1:32,11,7:8 0/1:24,0,126:21 0/1:220,0,255:194

10 9821210 . C T 999 . . GT:PL:DP 0/1:80,0,255:90 0/1:199,0,255:92 0/1:145,0,255:93 0/1:34,0,137:17 0/1:33,0,36:21 0/1:14,0,41:21 0/1:149,0,255:139

10 9881258 . C T 999 . . GT:PL:DP 0/1:219,0,7:20 0/1:171,0,128:20 0/1:152,0,133:18 0/1:12,0,123:8 0/1:41,0,96:6 0/1:79,9,0:3 0/1:217,0,215:36

10 9986128 . G A 999 . . GT:PL:DP 0/1:157,0,153:55 0/1:144,0,201:69 0/1:142,0,127:34 0/1:59,0,28:3 0/1:130,0,13:11 0/1:63,6,0:2 0/1:187,0,223:71

10 10002989 . T C 999 . . GT:PL:DP 0/1:91,0,43:11 0/1:114,0,27:12 0/1:143,4,0:16 0/1:14,0,41:3 0/1:35,6,0:2 0/1:31,0,20:4 0/1:115,0,53:18

10 10018229 . G A 999 . . GT:PL:DP 0/1:255,0,255:275 0/1:255,0,255:275 0/1:255,0,255:279 0/1:255,0,255:137 0/1:235,0,190:227 0/1:189,0,212:252 0/1:255,0,255:276

10 10018231 . C T 999 . . GT:PL:DP 0/1:255,0,255:277 0/1:255,0,255:277 0/1:255,0,255:281 0/1:255,0,255:138 0/1:235,0,183:227 0/1:199,0,200:253 0/1:255,0,255:278

10 10018254 . G A 999 . . GT:PL:DP 0/1:109,0,255:300 0/1:255,0,255:298 0/1:224,0,255:304 0/1:255,0,255:163 0/1:141,0,196:224 0/1:42,0,159:252 0/1:255,0,255:301

10 10018338 . G T 999 . . GT:PL:DP 0/1:255,0,253:216 0/1:255,0,255:209 0/1:255,0,221:212 0/1:255,0,255:164 0/1:255,0,250:136 0/1:255,0,241:222 0/1:255,0,255:210

10 10018395 . A G 999 . . GT:PL:DP 0/1:255,0,245:221 0/1:255,0,255:220 0/1:255,0,195:229 0/1:255,0,255:153 0/1:255,0,255:181 0/1:255,0,222:175 0/1:255,0,255:228

10 10019890 . G A 999 . . GT:PL:DP 0/1:162,0,83:14 0/1:57,0,141:9 0/1:175,0,95:18 0/1:80,9,0:3 0/1:23,0,60:4 0/1:42,6,0:2 0/1:148,0,146:21

10 10056349 . A T 999 . . GT:PL:DP 0/1:255,0,255:188 0/1:242,0,235:181 0/1:176,0,255:187 0/1:138,0,160:49 0/1:120,0,14:10 0/1:85,0,70:13 0/1:247,0,255:179

10 10056350 . G C 999 . . GT:PL:DP 0/1:244,0,233:184 0/1:241,0,202:176 0/1:182,0,255:183 0/1:140,0,155:47 0/1:118,0,9:10 0/1:85,0,65:13 0/1:244,0,255:176

10 10062789 . T A 999 . . GT:PL:DP 0/1:255,0,235:136 0/1:255,0,248:165 0/1:239,0,241:203 0/1:183,0,73:38 0/1:5,0,190:46 0/1:130,0,208:36 0/1:255,0,203:240

10 10070743 . A G 999 . . GT:PL:DP 0/1:101,0,103:159 0/1:65,0,48:149 0/1:115,0,85:146 0/1:133,0,62:33 0/1:53,0,103:17 0/1:78,0,255:44 0/1:164,0,255:163

10 10104134 . A T 999 . . GT:PL:DP 0/1:39,0,54:6 0/1:40,0,43:6 0/1:18,0,49:3 0/1:18,0,37:3 0/1:13,0,1:3 0/1:15,6,0:2 0/1:109,0,58:17

10 10104136 . A T 999 . . GT:PL:DP 0/1:61,0,41:9 0/1:50,0,34:6 0/1:18,0,49:3 0/1:16,0,29:3 0/1:13,0,1:3 0/1:16,9,0:3 0/1:109,0,46:17

10 10104138 . A C 999 . . GT:PL:DP 0/1:65,0,41:8 0/1:78,0,28:5 0/1:78,0,44:5 0/1:21,0,27:2 0/1:13,0,1:3 0/1:16,9,0:3 0/1:113,0,44:14

10 10131515 . G A 999 . . GT:PL:DP 0/1:255,0,255:40 0/1:255,0,255:34 0/1:255,0,255:40 0/1:41,0,131:7 0/1:121,0,23:6 0/1:21,0,105:6 0/1:255,0,255:46

10 10131716 . A G 999 . . GT:PL:DP 0/1:133,0,162:43 0/1:210,0,159:39 0/1:151,0,107:38 0/1:12,0,110:8 0/1:43,0,34:9 0/1:8,0,54:3 0/1:184,0,167:54

10 10131719 . A G 999 . . GT:PL:DP 0/1:131,0,163:42 0/1:208,0,161:38 0/1:143,0,107:37 0/1:12,0,97:8 0/1:32,0,37:9 0/1:8,0,54:3 0/1:190,0,169:55

10 10230959 . A G 999 . . GT:PL:DP 0/1:47,0,180:21 0/1:93,0,148:29 0/1:123,0,65:17 0/1:9,3,0:1 0/1:51,6,0:2 0/1:35,6,0:2 0/1:15,0,220:32

10 10230960 . T C 999 . . GT:PL:DP 0/1:47,0,180:21 0/1:93,0,152:29 0/1:104,0,68:16 0/1:9,3,0:1 0/1:51,6,0:2 0/1:35,6,0:2 0/1:19,0,224:32

10 10245550 . A G 999 . . GT:PL:DP 0/1:196,0,209:92 0/1:148,0,210:81 0/1:121,0,181:58 0/1:18,0,72:9 0/1:47,0,184:17 0/1:13,0,185:21 0/1:255,0,255:129

10 10373720 . C T 999 . . GT:PL:DP 0/1:150,0,117:209 0/1:112,0,100:193 0/1:129,0,67:154 0/1:106,0,61:36 0/1:13,0,29:9 0/1:35,5,0:6 0/1:171,0,222:250

10 10373953 . A G 999 . . GT:PL:DP 0/1:255,0,255:220 0/1:255,0,255:216 0/1:255,0,255:180 0/1:204,0,255:56 0/1:12,0,105:18 0/1:38,0,31:21 0/1:24,0,255:212

10 10374531 . C A 999 . . GT:PL:DP 0/1:232,0,255:163 0/1:255,0,255:139 0/1:255,0,255:156 0/1:75,0,240:43 0/1:33,0,253:53 0/1:63,0,165:32 0/1:188,0,255:219

10 10374550 . G A 999 . . GT:PL:DP 0/1:215,0,255:139 0/1:255,0,255:136 0/1:255,0,255:139 0/1:71,0,244:37 0/1:8,0,225:42 0/1:40,0,135:23 0/1:185,0,255:217

10 10410318 . T G 999 . . GT:PL:DP 0/1:255,0,255:143 0/1:255,0,255:122 0/1:255,0,255:121 0/1:229,0,144:29 0/1:42,0,14:21 0/1:64,0,48:28 0/1:255,0,255:205

10 10432058 . T C 999 . . GT:PL:DP 0/1:255,0,58:102 0/1:231,0,255:121 0/1:255,0,250:78 0/1:164,0,150:14 0/1:42,0,137:17 0/1:183,0,41:16 0/1:201,0,255:109

10 10433895 . G A 999 . . GT:PL:DP 0/1:192,0,109:98 0/1:107,0,255:189 0/1:165,0,255:85 0/1:21,0,254:19 0/1:48,0,199:14 0/1:176,0,208:40 0/1:255,0,255:195

10 10433911 . A G 999 . . GT:PL:DP 0/1:157,0,32:104 0/1:119,0,255:195 0/1:175,0,255:88 0/1:44,0,233:19 0/1:99,0,190:17 0/1:196,0,255:46 0/1:255,0,255:200

10 10434055 . A T 999 . . GT:PL:DP 0/1:239,0,36:134 0/1:122,0,255:187 0/1:255,0,255:113 0/1:108,0,255:25 0/1:195,0,175:17 0/1:255,0,255:55 0/1:255,0,255:199

10 10436101 . A T 999 . . GT:PL:DP 0/1:255,0,128:109 0/1:203,0,33:148 0/1:255,0,164:96 0/1:191,0,214:38 0/1:159,0,56:23 0/1:132,0,33:16 0/1:255,0,250:149

10 10442013 . G A 999 . . GT:PL:DP 0/1:255,0,255:203 0/1:255,0,241:188 0/1:217,0,255:187 0/1:203,0,255:47 0/1:145,0,163:21 0/1:255,0,71:42 0/1:255,0,255:217

10 10442038 . T A 999 . . GT:PL:DP 0/1:255,0,255:166 0/1:255,0,202:118 0/1:255,0,255:142 0/1:252,0,255:38 0/1:134,0,210:20 0/1:255,0,106:41 0/1:255,0,255:198

10 10442093 . A C 999 . . GT:PL:DP 0/1:255,0,255:158 0/1:255,0,255:117 0/1:255,0,255:149 0/1:222,0,238:39 0/1:100,0,234:17 0/1:213,0,134:28 0/1:255,0,255:197

10 10442122 . T A 999 . . GT:PL:DP 0/1:255,0,255:135 0/1:232,0,255:106 0/1:236,0,255:124 0/1:225,0,232:38 0/1:112,0,248:19 0/1:178,0,230:33 0/1:255,0,255:201

10 10442152 . A G 999 . . GT:PL:DP 0/1:255,0,255:114 0/1:196,0,255:115 0/1:211,0,255:130 0/1:189,0,241:38 0/1:19,0,250:16 0/1:29,0,248:24 0/1:226,0,255:200

10 10442158 . C T 999 . . GT:PL:DP 0/1:255,0,255:106 0/1:192,0,255:115 0/1:192,0,255:132 0/1:173,0,255:39 0/1:11,0,255:17 0/1:25,0,253:24 0/1:215,0,255:193

10 10442254 . A G 999 . . GT:PL:DP 0/1:255,0,255:101 0/1:255,0,255:114 0/1:203,0,255:122 0/1:15,0,255:20 0/1:94,0,255:43 0/1:9,0,255:36 0/1:51,0,255:182

10 10442413 . G A 999 . . GT:PL:DP 0/1:255,0,255:116 0/1:255,0,255:105 0/1:255,0,255:110 0/1:234,0,217:28 0/1:103,0,229:31 0/1:211,0,255:51 0/1:255,0,255:146

10 10442425 . C T 999 . . GT:PL:DP 0/1:255,0,255:114 0/1:255,0,255:100 0/1:255,0,255:103 0/1:235,0,229:27 0/1:109,0,209:28 0/1:173,0,255:43 0/1:255,0,255:138

10 10442430 . A G 999 . . GT:PL:DP 0/1:255,0,255:113 0/1:255,0,255:89 0/1:255,0,255:94 0/1:236,0,218:27 0/1:124,0,204:28 0/1:162,0,255:42 0/1:255,0,255:135

10 10442468 . G A 999 . . GT:PL:DP 0/1:255,0,255:89 0/1:255,0,255:65 0/1:255,0,255:51 0/1:199,0,114:17 0/1:65,0,142:15 0/1:130,0,255:28 0/1:255,0,255:103

10 10442480 . C A 999 . . GT:PL:DP 0/1:255,0,255:70 0/1:255,0,255:53 0/1:254,0,255:43 0/1:188,0,117:16 0/1:60,0,152:15 0/1:53,0,176:20 0/1:255,0,255:89

10 10445169 . T G 999 . . GT:PL:DP 0/1:126,0,255:176 0/1:179,0,255:177 0/1:12,0,227:189 0/1:164,0,255:62 0/1:46,0,255:84 0/1:141,0,255:116 0/1:150,0,255:154

10 10445488 . C A 999 . . GT:PL:DP 0/1:255,0,255:219 0/1:255,0,255:202 0/1:255,0,255:212 0/1:255,0,255:96 0/1:243,0,255:94 0/1:255,0,248:78 0/1:255,0,255:200

10 10445574 . T A 999 . . GT:PL:DP 0/1:12,0,255:224 0/1:49,0,255:204 0/1:66,0,255:219 0/1:175,0,255:86 0/1:121,0,255:97 0/1:177,0,255:78 0/1:86,0,255:224

10 10445634 . A G 999 . . GT:PL:DP 0/1:244,0,255:230 0/1:255,0,255:205 0/1:203,0,255:211 0/1:66,0,255:71 0/1:6,0,255:76 0/1:23,0,255:68 0/1:72,0,255:220

10 10446060 . C T 999 . . GT:PL:DP 0/1:177,0,186:220 0/1:204,0,228:200 0/1:211,0,167:209 0/1:120,0,80:42 0/1:134,0,87:51 0/1:103,0,101:68 0/1:142,0,223:217

10 10592868 . C A 999 . . GT:PL:DP 0/1:123,0,255:42 0/1:255,0,143:73 0/1:155,0,211:31 0/1:85,0,27:7 0/1:15,2,0:6 0/1:105,4,0:21 0/1:255,0,247:75

10 10593003 . C T 999 . . GT:PL:DP 0/1:94,0,146:17 0/1:197,6,0:51 0/1:164,0,83:21 0/1:25,0,59:4 0/1:37,3,0:1 0/1:32,1,0:2 0/1:172,0,52:34

10 10683652 . T A 999 . . GT:PL:DP 0/1:206,0,105:45 0/1:185,0,163:73 0/1:215,0,153:47 0/1:19,0,57:4 0/1:79,0,84:11 0/1:15,0,67:4 0/1:196,0,198:115

10 10683887 . A G 999 . . GT:PL:DP 0/1:255,0,221:99 0/1:222,0,243:104 0/1:187,0,212:67 0/1:123,0,183:21 0/1:177,0,87:14 0/1:104,0,99:15 0/1:31,0,108:218

10 10683993 . T A 999 . . GT:PL:DP 0/1:255,0,255:62 0/1:255,0,255:116 0/1:187,0,255:117 0/1:115,0,207:21 0/1:21,0,100:8 0/1:150,0,161:17 0/1:146,0,193:216

10 10736177 . T G 999 . . GT:PL:DP 0/1:217,0,255:100 0/1:232,0,255:168 0/1:72,0,255:189 0/1:100,0,223:30 0/1:99,10,0:10 0/1:109,0,73:24 0/1:222,0,255:200

10 10870225 . T C 999 . . GT:PL:DP 0/1:25,0,247:21 0/1:78,0,249:22 0/1:23,0,255:26 0/1:20,0,84:4 0/1:31,0,27:2 0/1:25,0,75:4 0/1:152,0,255:50

10 10900666 . A T 999 . . GT:PL:DP 0/1:120,0,4:12 0/1:83,0,14:7 0/1:92,0,4:7 0/1:46,0,32:4 0/1:29,6,0:2 0/1:10,0,4:2 0/1:143,0,57:17

10 10900667 . T A 999 . . GT:PL:DP 0/1:113,0,5:12 0/1:83,0,14:7 0/1:92,0,13:7 0/1:46,0,42:4 0/1:29,6,0:2 0/1:10,0,4:2 0/1:145,0,52:16

10 10960821 . T C 999 . . GT:PL:DP 0/1:201,0,159:23 0/1:238,0,99:25 0/1:159,0,151:23 0/1:102,0,48:6 0/1:16,0,107:5 0/1:76,9,0:3 0/1:171,0,247:36

10 10996707 . G A 999 . . GT:PL:DP 0/1:209,0,192:21 0/1:98,0,112:11 0/1:205,0,197:20 0/1:48,0,105:7 0/1:22,0,74:4 0/1:22,0,96:4 0/1:197,0,255:36

10 11240236 . T A 999 . . GT:PL:DP 0/1:89,0,32:9 0/1:141,3,0:9 0/1:115,0,3:10 0/1:22,3,0:1 0/1:49,6,0:2 0/1:34,3,0:1 0/1:50,0,6:5

10 11240748 . C T 999 . . GT:PL:DP 0/1:255,0,48:42 0/1:255,0,44:48 0/1:237,0,134:55 0/1:98,8,0:5 0/1:31,3,0:1 0/1:150,6,0:6 0/1:255,0,98:36

10 11253542 . T C 999 . . GT:PL:DP 0/1:255,0,18:48 0/1:255,0,255:61 0/1:255,0,255:89 0/1:76,0,159:10 0/1:224,0,130:16 0/1:59,0,255:20 0/1:255,0,255:128

10 11253596 . T A 999 . . GT:PL:DP 0/1:255,0,58:53 0/1:255,0,255:52 0/1:255,0,255:97 0/1:87,0,87:6 0/1:226,0,143:16 0/1:132,0,255:23 0/1:255,0,255:138

10 11253678 . T A 999 . . GT:PL:DP 0/1:255,0,196:50 0/1:255,0,191:37 0/1:255,0,255:91 0/1:141,0,13:7 0/1:142,0,120:13 0/1:45,0,255:29 0/1:255,0,255:134

10 11253682 . G A 999 . . GT:PL:DP 0/1:255,0,196:47 0/1:255,0,177:36 0/1:255,0,255:87 0/1:148,0,13:7 0/1:142,0,116:13 0/1:49,0,255:32 0/1:255,0,255:135

10 11253932 . C T 999 . . GT:PL:DP 0/1:255,0,255:85 0/1:255,0,255:70 0/1:255,0,255:97 0/1:234,0,202:20 0/1:84,0,252:18 0/1:161,0,255:34 0/1:255,0,255:126

10 11254055 . C T 999 . . GT:PL:DP 0/1:255,0,255:101 0/1:255,0,255:74 0/1:255,0,255:93 0/1:172,0,255:23 0/1:114,0,255:29 0/1:50,0,255:36 0/1:232,0,255:132

10 11254076 . A C 999 . . GT:PL:DP 0/1:255,0,255:105 0/1:255,0,255:80 0/1:255,0,255:95 0/1:184,0,255:25 0/1:71,0,255:22 0/1:75,0,255:39 0/1:177,0,255:136

10 11254160 . A G 999 . . GT:PL:DP 0/1:240,0,255:104 0/1:255,0,255:100 0/1:255,0,255:105 0/1:194,0,216:19 0/1:70,0,255:17 0/1:126,0,255:41 0/1:255,0,255:137

10 11254383 . C T 999 . . GT:PL:DP 0/1:255,0,255:146 0/1:239,0,255:109 0/1:255,0,255:130 0/1:148,0,226:22 0/1:8,0,255:27 0/1:116,0,255:49 0/1:230,0,255:154

10 11268589 . A G 999 . . GT:PL:DP 0/1:255,0,255:144 0/1:215,0,255:124 0/1:255,0,255:138 0/1:213,0,221:48 0/1:61,0,113:13 0/1:43,0,127:7 0/1:255,0,255:191

10 11268833 . G C 999 . . GT:PL:DP 0/1:225,0,157:84 0/1:151,0,213:61 0/1:207,0,201:95 0/1:194,0,98:30 0/1:45,0,81:8 0/1:110,0,159:18 0/1:243,0,255:157

10 11281557 . C T 999 . . GT:PL:DP 0/1:255,0,242:62 0/1:255,0,162:83 0/1:227,0,152:50 0/1:140,0,79:11 0/1:101,0,36:11 0/1:111,0,3:5 0/1:255,0,244:90

10 11281944 . C G 999 . . GT:PL:DP 0/1:255,0,203:64 0/1:245,0,217:73 0/1:255,0,242:115 0/1:64,0,121:14 0/1:102,0,64:8 0/1:187,0,9:16 0/1:255,0,213:114

10 11281948 . G T 999 . . GT:PL:DP 0/1:255,0,193:60 0/1:245,0,215:69 0/1:255,0,239:110 0/1:57,0,144:15 0/1:123,0,62:9 0/1:213,0,7:17 0/1:255,0,206:111

10 11345146 . A G 999 . . GT:PL:DP 0/1:27,0,211:21 0/1:63,0,35:8 0/1:63,0,97:15 0/1:15,0,42:4 0/1:39,0,66:7 0/1:101,0,45:16 0/1:125,0,135:19

10 11379901 . T C 999 . . GT:PL:DP 0/1:255,0,242:227 0/1:255,0,255:221 0/1:255,0,221:211 0/1:255,0,227:196 0/1:255,0,255:123 0/1:255,0,234:119 0/1:255,0,255:233

10 11379916 . G A 999 . . GT:PL:DP 0/1:255,0,190:215 0/1:255,0,224:207 0/1:252,0,157:198 0/1:255,0,159:182 0/1:255,0,240:95 0/1:255,0,191:128 0/1:255,0,255:218

10 11419157 . T G 999 . . GT:PL:DP 0/1:199,0,245:46 0/1:156,0,44:17 0/1:81,0,255:34 0/1:12,0,141:7 0/1:53,0,137:10 0/1:26,0,150:11 0/1:157,0,255:48

10 11574945 . A C 999 . . GT:PL:DP 0/1:169,0,108:34 0/1:86,0,73:25 0/1:100,0,189:47 0/1:21,0,12:3 0/1:33,9,0:3 0/1:78,6,0:5 0/1:87,0,122:47

10 11596915 . T A 999 . . GT:PL:DP 0/1:22,0,42:5 0/1:77,0,35:11 0/1:88,0,88:11 0/1:55,9,0:3 0/1:81,0,85:11 0/1:39,0,101:10 0/1:74,0,158:20

10 11700792 . G C 999 . . GT:PL:DP 0/1:149,0,255:35 0/1:198,0,232:70 0/1:99,0,255:40 0/1:10,0,143:8 0/1:82,0,5:9 0/1:128,0,234:61 0/1:170,0,233:88

10 11700950 . G C 999 . . GT:PL:DP 0/1:88,0,58:16 0/1:107,0,111:28 0/1:72,0,82:16 0/1:17,0,18:4 0/1:90,0,25:7 0/1:41,0,50:7 0/1:98,0,132:31

10 11830459 . G A 999 . . GT:PL:DP 0/1:255,0,157:45 0/1:245,0,138:32 0/1:193,0,73:29 0/1:173,0,35:10 0/1:75,0,123:10 0/1:110,0,163:16 0/1:255,0,248:61

10 11830476 . G A 999 . . GT:PL:DP 0/1:159,0,255:40 0/1:42,0,233:27 0/1:177,0,169:30 0/1:62,0,58:8 0/1:122,0,106:11 0/1:22,0,181:14 0/1:157,0,255:58

10 11830535 . G T 999 . . GT:PL:DP 0/1:221,0,181:37 0/1:185,0,92:30 0/1:105,0,170:35 0/1:40,0,63:8 0/1:15,0,71:4 0/1:80,0,27:8 0/1:231,0,178:49

10 11844789 . T C 999 . . GT:PL:DP 0/1:255,0,255:192 0/1:255,0,255:163 0/1:225,0,222:176 0/1:179,0,209:23 0/1:96,0,171:19 0/1:144,0,155:32 0/1:255,0,255:221

10 11844801 . T C 999 . . GT:PL:DP 0/1:255,0,255:193 0/1:255,0,255:153 0/1:230,0,230:175 0/1:178,0,212:25 0/1:93,0,160:20 0/1:136,0,154:34 0/1:255,0,255:207

10 12000682 . G A 999 . . GT:PL:DP 0/1:87,9,0:3 0/1:227,0,37:13 0/1:172,0,13:8 0/1:48,6,0:2 0/1:9,3,0:1 0/1:64,6,0:2 0/1:190,0,27:11

10 12052236 . C T 999 . . GT:PL:DP 0/1:179,0,97:18 0/1:100,0,161:21 0/1:165,0,77:20 0/1:51,0,89:5 0/1:49,0,17:3 0/1:19,0,44:4 0/1:168,0,232:37

10 12077751 . C G 999 . . GT:PL:DP 0/1:116,0,129:24 0/1:151,0,89:24 0/1:120,0,133:28 0/1:37,0,27:5 0/1:33,0,22:3 0/1:64,0,19:4 0/1:104,0,93:23

10 12077787 . T A 999 . . GT:PL:DP 0/1:96,0,116:26 0/1:35,0,150:25 0/1:80,0,121:26 0/1:21,0,39:5 0/1:79,0,16:6 0/1:6,0,68:6 0/1:68,0,110:24

10 12144886 . G A 999 . . GT:PL:DP 0/1:255,0,238:173 0/1:255,0,255:163 0/1:255,0,255:155 0/1:219,0,140:48 0/1:68,0,9:8 0/1:173,0,119:30 0/1:255,0,255:153

10 12145050 . T C 999 . . GT:PL:DP 0/1:166,0,255:185 0/1:168,0,255:200 0/1:140,0,255:200 0/1:62,0,253:52 0/1:33,10,0:13 0/1:31,0,59:15 0/1:66,0,255:153

10 12176265 . A C 999 . . GT:PL:DP 0/1:255,0,255:139 0/1:255,0,243:82 0/1:147,16,255:80 0/1:176,60,153:27 0/1:19,0,255:31 0/1:186,0,255:97 0/1:255,0,255:174

10 12273262 . C A 999 . . GT:PL:DP 0/1:17,0,194:91 0/1:148,0,223:98 0/1:93,0,219:107 0/1:31,0,105:32 0/1:54,0,216:47 0/1:123,0,205:45 0/1:30,0,216:212

10 12288958 . A T 999 . . GT:PL:DP 0/1:223,0,255:39 0/1:255,0,255:44 0/1:254,0,253:37 0/1:53,6,0:2 0/1:29,0,28:2 0/1:40,6,0:2 0/1:255,0,255:48

10 12390448 . G A 999 . . GT:PL:DP 0/1:255,0,244:245 0/1:229,0,182:245 0/1:199,0,197:236 0/1:255,0,250:158 0/1:146,0,250:131 0/1:216,0,255:110 0/1:220,0,155:248

10 12390474 . A T 999 . . GT:PL:DP 0/1:255,0,246:247 0/1:239,0,227:238 0/1:193,0,211:233 0/1:255,0,246:146 0/1:126,0,255:161 0/1:255,0,255:117 0/1:232,0,195:259

10 12390564 . A G 999 . . GT:PL:DP 0/1:255,0,196:229 0/1:255,0,232:223 0/1:255,0,251:219 0/1:255,0,213:167 0/1:249,0,255:186 0/1:235,0,255:121 0/1:255,0,204:238

10 12521708 . C T 999 . . GT:PL:DP 0/1:74,0,255:62 0/1:208,0,255:61 0/1:83,0,255:68 0/1:70,0,100:8 0/1:13,0,52:12 0/1:101,0,103:34 0/1:32,0,255:106

10 12576686 . G A 999 . . GT:PL:DP 0/1:118,0,143:18 0/1:126,0,106:19 0/1:132,0,160:32 0/1:28,0,31:3 0/1:90,0,122:10 0/1:18,0,80:4 0/1:54,0,165:27

10 12583895 . A G 999 . . GT:PL:DP 0/1:117,0,129:18 0/1:226,0,109:27 0/1:170,0,216:40 0/1:45,0,16:4 0/1:51,6,0:2 0/1:40,0,42:5 0/1:158,0,163:34

10 12600774 . G A 999 . . GT:PL:DP 0/1:114,0,75:20 0/1:81,0,123:15 0/1:83,0,124:19 0/1:16,3,0:1 0/1:6,0,9:3 0/1:18,0,1:2 0/1:59,0,98:15

10 12600775 . T A 999 . . GT:PL:DP 0/1:104,0,75:20 0/1:81,0,114:15 0/1:78,0,124:19 0/1:16,3,0:1 0/1:6,0,9:3 0/1:36,16,16:3 0/1:61,0,94:14

10 12600776 . T G 999 . . GT:PL:DP 0/1:114,0,75:20 0/1:63,0,128:15 0/1:83,0,124:19 0/1:16,3,0:1 0/1:6,0,9:3 0/1:44,1,0:3 0/1:61,0,94:14

10 12600777 . A C 999 . . GT:PL:DP 0/1:151,0,70:22 0/1:70,0,130:16 0/1:86,0,110:18 0/1:16,3,0:1 0/1:6,0,9:3 0/1:44,1,0:3 0/1:61,0,94:14

10 12600778 . A T 999 . . GT:PL:DP 0/1:133,0,70:22 0/1:110,0,86:15 0/1:86,0,101:18 0/1:16,3,0:1 0/1:6,0,9:3 0/1:44,1,0:3 0/1:61,0,94:14

10 12600779 . T A 999 . . GT:PL:DP 0/1:169,0,65:23 0/1:157,0,56:17 0/1:86,0,101:18 0/1:16,3,0:1 0/1:6,0,1:3 0/1:44,1,0:3 0/1:61,0,86:14

10 12662167 . A G 999 . . GT:PL:DP 0/1:104,0,255:229 0/1:255,0,255:203 0/1:234,0,255:221 0/1:255,0,255:47 0/1:73,0,162:68 0/1:33,0,103:57 0/1:61,0,235:225

10 12729168 . T G 999 . . GT:PL:DP 0/1:123,0,217:67 0/1:167,0,124:59 0/1:160,0,121:74 0/1:81,0,60:13 0/1:13,0,24:7 0/1:10,0,86:9 0/1:171,0,167:74

10 12729201 . T A 999 . . GT:PL:DP 0/1:166,0,204:69 0/1:185,0,105:65 0/1:120,0,191:77 0/1:84,0,67:14 0/1:46,0,6:6 0/1:37,0,25:7 0/1:164,0,161:86

10 12729299 . T C 999 . . GT:PL:DP 0/1:189,0,150:44 0/1:148,0,61:28 0/1:204,0,34:43 0/1:34,0,82:8 0/1:111,0,5:10 0/1:48,0,4:4 0/1:207,0,121:67

10 12729511 . A T 999 . . GT:PL:DP 0/1:255,0,255:208 0/1:255,0,255:189 0/1:255,0,255:213 0/1:255,0,207:44 0/1:177,0,208:68 0/1:49,0,206:95 0/1:192,0,255:230

10 12729539 . A C 999 . . GT:PL:DP 0/1:255,0,255:188 0/1:255,0,255:177 0/1:255,0,255:182 0/1:255,0,255:46 0/1:145,0,206:43 0/1:17,0,232:66 0/1:220,0,255:193
[truncated: 859,364 more chars]
