# Supplementary material for: Accurate identification of Helicoverpa armigera–Helicoverpa zea hybrids using genome admixture analysis: implications for genomic surveillance
Source: Front Insect Sci. 2024 Feb 23;4:1339143. doi: 10.3389/finsc.2024.1339143 (PMC10926370; doi:10.3389/finsc.2024.1339143)
Supplement: Supplementary file 2 [file Table_1.pdf]

Supplementary Table 1 Sequencing statistics.

Fastq files corresponding to this table can be found in the SRA under BioProject ID: PRJNA973566 or SRA submission number SUB13362524. The latter four genomes were sequenced in two separate lanes. After filtration and repair, reads from the two lanes were merged before mapping.

Sample name	Sample code	Number of reads	Number of filtered reads	Average length of filtered reads (bp)	Q20 bases of filtered reads (%)	Q30 bases of filtered reads (%)
PRh	RAPiD-Genomics_F061_UPR_134801_P001_WA01_i5-505_i7-59_S1442_L008_R1_001.fastq	68237942	68215872	149	98.6	96.0
	RAPiD-Genomics_F061_UPR_134801_P001_WA01_i5-505_i7-59_S1442_L008_R2_001.fastq	68237942	68160626	149	96.4	92.0
MAh	RAPiD-Genomics_F061_UPR_134801_P001_WA02_i5-505_i7-27_S1443_L008_R1_001.fastq	69981718	69481299	149	98.6	96.2
	RAPiD-Genomics_F061_UPR_134801_P001_WA02_i5-505_i7-27_S1443_L008_R2_001.fastq	69981718	69462248	148	95.1	89.2
HaM	RAPiD-Genomics_F071_UPR_134802_P001_WA01_i5-508_i7-59_S2885_L004_R1_001.fastq	81978423	81952805	148	97.5	93.4
	(RAPiD-Genomics_F071_UPR_134802_P001_WA01_i5-508_i7-59_S2885_L004_R2_001.fastq)	81978423	81950055	147	95.6	90.4
HzF	RAPiD-Genomics_F071_UPR_134802_P001_WA02_i5-508_i7-27_S2886_L004_R1_001.fastq	74909547	74854750	148	97.6	93.6
	RAPiD-Genomics_F071_UPR_134802_P001_WA02_i5-508_i7-27_S2886_L004_R2_001.fastq	74909547	74856157	147	95.8	90.7
HzCol	RAPiD-Genomics_F131_UPR_134803_P001_WA01_i5-512_i7-97_S184_L002_R1_001.fastq.gz	40950840	40909180	148	98.4	95.1
	RAPiD-Genomics_F131_UPR_134803_P001_WA01_i5-512_i7-97_S184_L002_R2_001.fastq.gz	41688962	41645905	148	98.5	95.1
	RAPiD-Genomics_F134_UPR_134803_P001_WA01_i5-512_i7-97_S5_L001_R1_001.fastq.gz	9003282	9002312	149	96.4	90.4
	RAPiD-Genomics_F134_UPR_134803_P001_WA01_i5-512_i7-97_S5_L001_R2_001.fastq.gz	9003282	8999277	149	95.2	88.1

HzIll	RAPiD-Genomics_F131_UPR_134803_P001_WB01_i5-512_i7-109_S185_L002_R1_001.fastq.gz	41688962	41645904	148	98.5	95.1
	RAPiD-Genomics_F131_UPR_134803_P001_WB01_i5-512_i7-109_S185_L002_R2_001.fastq.gz	41688962	41670503	148	98.0	93.9
	RAPiD-Genomics_F134_UPR_134803_P001_WB01_i5-512_i7-109_S6_L001_R1_001.fastq.gz	8180764	8179779	149	96.4	90.5
	RAPiD-Genomics_F134_UPR_134803_P001_WB01_i5-512_i7-109_S6_L001_R2_001.fastq.gz	8180764	8177487	149	95.4	88.6
HzMaine	RAPiD-Genomics_F131_UPR_134803_P001_WC01_i5-512_i7-121_S186_L002_R1_001.fastq.gz	41936744	41894121	148	98.5	95.1
	RAPiD-Genomics_F131_UPR_134803_P001_WC01_i5-512_i7-121_S186_L002_R2_001.fastq.gz	41936744	41916752	148	98.1	94.0
	RAPiD-Genomics_F134_UPR_134803_P001_WC01_i5-512_i7-121_S7_L001_R1_001.fastq.gz	8823812	8822863	149	96.4	90.5
	RAPiD-Genomics_F134_UPR_134803_P001_WC01_i5-512_i7-121_S7_L001_R2_001.fastq.gz	8823812	8819941	149	95.4	88.6
HzNC	RAPiD-Genomics_F131_UPR_134803_P001_WD01_i5-512_i7-133_S187_L002_R1_001.fastq.gz	43364690	43319834	149	98.4	95.1
	RAPiD-Genomics_F131_UPR_134803_P001_WD01_i5-512_i7-133_S187_L002_R2_001.fastq.gz	43364690	43341100	149	98.0	93.9
	RAPiD-Genomics_F134_UPR_134803_P001_WD01_i5-512_i7-133_S8_L001_R1_001.fastq.gz	8677566	8676286	150	96.4	90.3
	RAPiD-Genomics_F134_UPR_134803_P001_WD01_i5-512_i7-133_S8_L001_R2_001.fastq.gz	8677566	8673245	150	95.3	88.4
